# Next Generation Copper Mediators for the Efficient Production of ^18^F‐Labeled Aromatics

**DOI:** 10.1002/chem.202202965

**Published:** 2022-11-17

**Authors:** Chris Hoffmann, Niklas Kolks, Daniel Smets, Alexander Haseloer, Benedikt Gröner, Elizaveta A. Urusova, Heike Endepols, Felix Neumaier, Uwe Ruschewitz, Axel Klein, Bernd Neumaier, Boris D. Zlatopolskiy

**Affiliations:** ^1^ Faculty of Medicine and University Hospital Cologne Institute of Radiochemistry and Experimental Molecular Imaging University of Cologne Kerpener Straße 62 50937 Cologne Germany; ^2^ Institute of Neuroscience and Medicine Nuclear Chemistry (INM-5) Forschungszentrum Jülich GmbH Wilhelm-Johnen-Straße 52428 Jülich Germany; ^3^ Faculty of Mathematics and Natural Sciences Department of Chemistry Institute of Inorganic Chemistry University of Cologne Greinstr. 6 50939 Cologne Germany; ^4^ Faculty of Medicine and University Hospital Cologne Department of Nuclear Medicine University of Cologne Kerpener Straße 62 50937 Cologne Germany

**Keywords:** copper, fluorine-18, positron emission tomography, radiochemistry, radiopharmaceuticals

## Abstract

Cu‐mediated radiofluorination is a versatile tool for the preparation of ^18^F‐labeled (hetero)aromatics. In this work, we systematically evaluated a series of complexes and identified several generally applicable mediators for highly efficient radiofluorination of aryl boronic and stannyl substrates. Utilization of these mediators in *n*BuOH/DMI or DMI significantly improved ^18^F‐labeling yields despite use of lower precursor amounts. Impressively, application of 2.5 μmol aryl boronic acids was sufficient to achieve ^18^F‐labeling yields of up to 75 %. The practicality of the novel mediators was demonstrated by efficient production of five PET‐tracers and transfer of the method to an automated radiosynthesis module. In addition, (*S*)‐3‐[^18^F]FPhe and 6‐[^18^F]FDOPA were prepared in activity yields of 23±1 % and 30±3 % using only 2.5 μmol of the corresponding boronic acid or trimethylstannyl precursor.

## Graphical abstract

**Evaluation of a series of Cu(II) complexes** revealed several highly efficient mediators for radiofluorination of aryl boronic and stannyl substrates. After optimization of the reaction conditions, their application enabled preparation of seven PET‐tracers in improved radiochemical yields and/or using significantly lower precursor loadings.

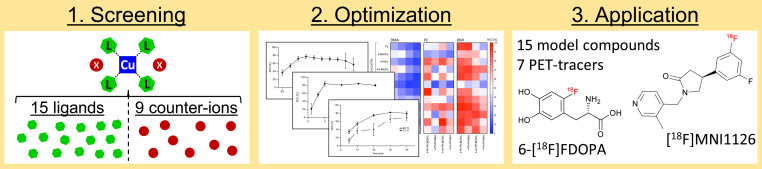

## Introduction

Positron emission tomography (PET) is a non‐invasive imaging technique that employs probes labeled with β^+^‐emitting radionuclides to visualize biochemical processes for research or diagnostic purposes. The most widely used PET radionuclide is fluorine‐18, which is readily accessible in the form of [^18^F]fluoride ([^18^F]F^−^) from [^18^O]H_2_O via the ^18^O(p,n)^18^F nuclear reaction. Fluorine‐18 has advantageous decay properties (high β^+^‐emission probability, relatively low β^+^ energy, suitably long half‐life of 110 min) compared to other common short‐lived PET isotopes like ^11^C, ^13^N, ^15^O or ^68^Ga.^[1]^ However, direct application of [^18^F]F^−^ in [^18^O]H_2_O for radiofluorination is usually not feasible due to its high degree and strength of hydration.^[2]^ As such, time consuming azeotropic drying steps and addition of bases and phase transfer catalysts are usually required to obtain highly nucleophilic [^18^F]F^−^ that can be further used for S_n_2, S_n_Ar or transition metal‐catalyzed reactions.^[3]^ Owing to radioactive decay and inevitable heating‐induced adsorption of [^18^F]F^−^ to the reactor walls, azeotropic drying and other pre‐processing steps are invariably associated with radioactivity losses. However, these steps and the use of additives can be omitted, for example, by applying the “minimalist”^[4]^ or related^[5]^ protocols for S_n_2, S_n_Ar or Cu‐mediated radiofluorination.

Recently introduced approaches for Cu‐mediated production of ^18^F‐labeled (hetero)arenes from different substrates,^[6]^ including readily available aryl boronic acids,^[7]^ pinacol boronates^[8]^ and trialkylstannanes,^[9]^ have granted access to emerging or established but otherwise hardly accessible radiotracers. Whereas the original procedures were rather inefficient and not well suited for radiotracer production on a preparative scale, several approaches to circumvent these limitations have since been proposed,[^5b^, ^10^] which significantly contributed to the dramatic growth of PET imaging in the last 5–7 years. Main drawback of these procedures in their current form is that relatively large amounts of labeling precursor and Cu(Py)_4_(OTf)_2_ (≥20 μmol of each) are often required to achieve high and reproducible radiochemical yields (RCYs),^[11]^ especially when radiosyntheses are performed in remotely controlled synthesis units. This not only increases production costs, but in many cases also complicates purification of the resulting PET‐tracers. Surprisingly, to date, no significant efforts have been made to find more efficient radiolabeling mediators, which could help to eliminate this bottleneck.

**Scheme 1:**
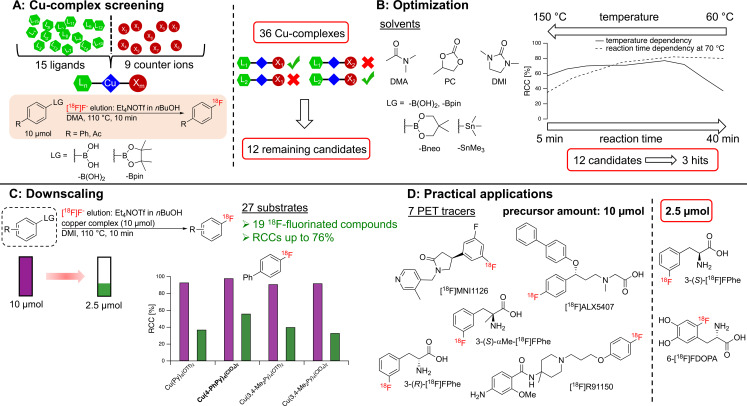
Workflow of the present study on different Cu(II) complexes for efficient radiofluorination of aryl boronic and stannyl substrates. A) During an initial screening of a total of 36 Cu(II) complexes with different ligands and counter ions, B) the 12 most promising candidates were identified and used for subsequent optimization studies. C) Following downscaling experiments with various model compounds, D) three of the candidate complexes were used to prepare different PET‐tracers.

In this study, we first prepared a series of Cu(II) complexes and evaluated their efficiencies as mediators for radiofluorination of various model substrates bearing ‐B(OH)_2_ or ‐Bpin groups (Scheme 1A). The most promising candidates were selected and used in subsequent experiments to optimize further reaction parameters like solvent, time and temperature (Scheme 1B). In addition, the developed radiofluorination protocol was adapted to radiolabeling of aryl trialkylstannanes (Scheme 1B) and to the use of low precursor amounts (Scheme 1C). Finally, we exemplified the suitability of the optimized protocol for the manual and automated preparation of several PET‐tracers (Scheme 1D).

## Results and Discussion

In order to assess the influence of different ligands on radiofluorination, we prepared a series of Cu(II) complexes with fifteen N‐heterocycles and nine counter ions. For comparison, Co(II) perchlorate and Ni(II) triflate pyridine complexes were also synthesized. For three copper complexes, including the literature known Cu(Py)_4_(OTf)_2_ (with known crystal structure)^[12]^ as well as Cu(3,4‐Me_2_Py)_4_(OTf)_2_ and Cu(4‐MeOPy)_4_(ClO_4_)_2_, the crystal structures were determined (see Supporting Information for more details).

**Scheme 2:**
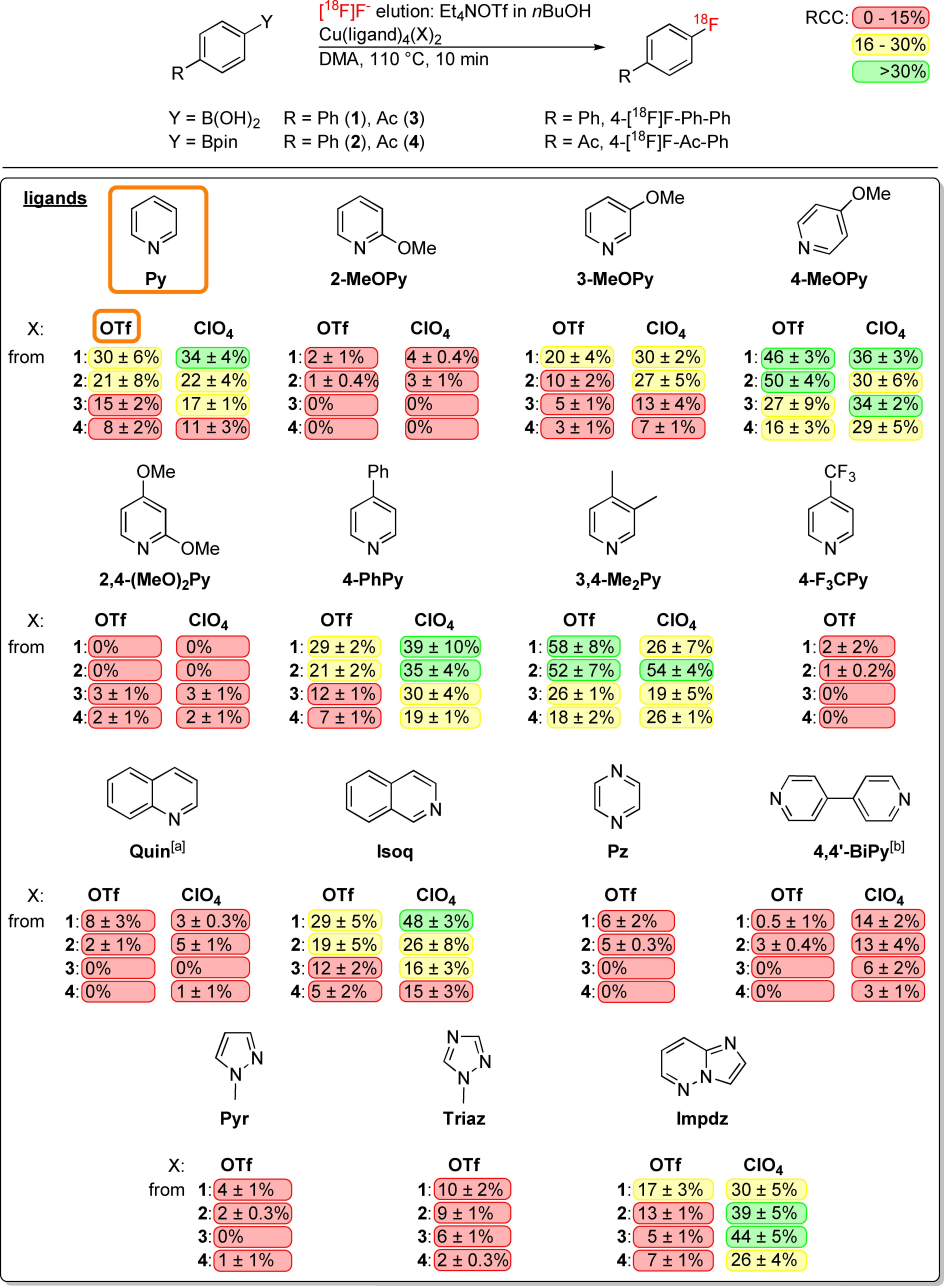
Radiofluorination of aryl boronic acids and pinacol boronates using different mediators in *n*BuOH/DMA. Conditions: i) elution of [^18^F]F^−^ (10–50 MBq) with Et_4_NOTf (1 mg, 4 μmol) in nBuOH (400 μL) into a solution of substrates **1**–**4** (10 μmol, 1 eq.) and Cu(ligand)_4_(X)_2_ (10 μmol, 1 eq.) in DMA (800 μL); ii) 110 °C for 10 min under air; iii) addition of H_2_O (1 mL). Radiochemical conversions (RCCs) are provided in the following format: mean RCC±standard deviation (%). All experiments were carried out at least in triplicate. Conventional Cu(Py)_4_(OTf)_2_ is highlighted with an orange frame, while RCCs are color‐coded in red (0–15 %), yellow (16–30 %) or green (>30 %) as indicated in the upper right corner. Py (pyridine), 2‐MeOPy (2‐methoxypyridine), 3‐MeOPy (3‐methoxypyridine), 4‐MeOPy (4‐methoxypyridine), 2,4‐(MeO)_2_Py (2,4‐dimethoxypyridine), 4,4′‐BiPy (4,4′‐bipyridine), 4‐PhPy (4‐phenylpyridine), 3,4‐Me_2_Py (3,4‐lutidine), 4‐F_3_CPy (4‐trifluoromethylpyridine), Pz (pyrazine), Triaz (N‐methyl‐1,2,4‐triazole), Pyr (N‐methylpyrazole), Quin (quinoline), Isoq (isoquinoline), Impdz (imidazo(1,2‐b)pyridazine).^[a]^ Cu(Quin)_3_(MeOH)X_2_;^[b]^ Cu(4,4′‐BiPy)_2_X_2_.

For our initial optimization studies, we used sterically unhindered, moderately electron‐rich 4‐biphenyl‐ and CH acidic electron‐poor 4‐acetylphenyl‐boronic acids and pinacol boronates as “simple” and “difficult” model radiolabeling precursors, respectively (Scheme 2).

**Figure 1:**
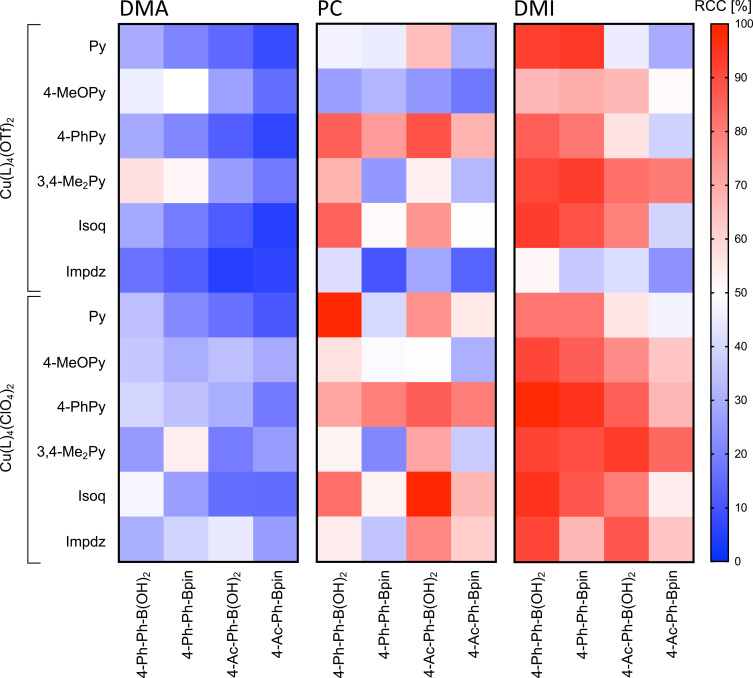
Dependency of radiochemical conversions (RCCs) on radiolabeling substrate and Cu mediator in different reaction solvents. Reaction conditions: [^18^F]F^−^ (10–50 MBq), radiolabeling substrate (10 μmol), Cu(L)_4_X_2_ (10 μmol) and Et_4_NOTf (1 mg, 4 μmol) in *n*BuOH/reaction solvent 1 : 2 (1.2 mL) at 110 °C for 10 min, followed by addition of H_2_O (2 mL). RCCs were determined by HPLC as described in the Supporting Information. All experiments were performed at least in triplicate. The color bar represents RCC (%). For full statistics including main effects and factor interactions, see Tables S18–S21 and S27–S34.

**Figure 2:**
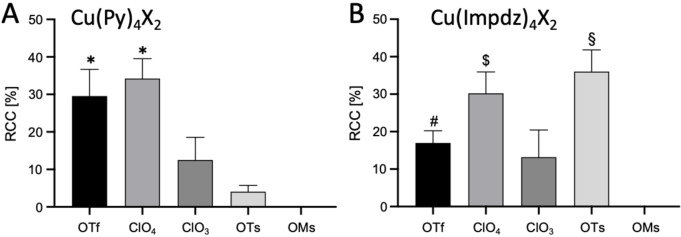
Radiochemical conversions (RCCs) for labeling of 4‐Ph‐Ph‐B(OH)_2_ using A) Cu(Py)_4_X_2_ and B) Cu(Impdz)_4_X_2_ in *n*BuOH/DMA. Reaction conditions: [^18^F]F^−^ (10–50 MBq), 4‐biphenyl boronic acid (10 μmol), Cu(L)_4_X_2_ (10 μmol) and Et_4_NOTf (1 mg, 4 μmol) in *n*BuOH/DMA 1 : 2 (1.2 mL) at 110 °C for 10 min, followed by addition of H_2_O (2 mL). RCCs were determined by HPLC as described in the Supporting Information. All experiments were performed in triplicate. Statistics: *: p<0.05 for comparison with ClO_3_, OTs and OMs; #: p<0.05 for comparison with OMs; $: p<0.05 for comparison with ClO_3_ and OMs; §: p<0.05 for comparison with OTf, ClO_3_ and OMs. For full statistics including main effects and factor interactions, see Tables S22 and S23.

Since copper complexes are prone to decomposition under basic conditions,^[4e]^ non‐basic tetraethylammonium triflate (Et_4_NOTf) was selected for [^18^F]F^−^ elution. Radiolabeling was carried out according to the alcohol‐enhanced protocol^[5b]^ as follows. [^18^F]F^−^ trapped on an anion exchange resin was eluted with Et_4_NOTf (1 mg, 4 μmol) in *n*BuOH (400 μL) directly into a solution of the copper complex and precursor (10 μmol of each) in DMA (800 μmol). The reaction mixture was heated at 110 °C for 10 min under air, diluted with H_2_O (2 mL), and radiochemical conversions (RCCs)^[11]^ were determined by HPLC. The results of the screening experiments were statistically evaluated using GraphPad Prism software. While highly reproducible [^18^F]F^−^ elution efficiencies of 90–95 % were observed, the RCCs varied markedly depending on the applied Cu mediator (Figure 1). The use of Ni or Co complexes as well as Cu(II) complexes with Br^−^, Cl^−^, OAc^−^, OMs^−^ or SO_4_^2−^ counter ions did not result in the formation of radiolabeled products (data not shown). Copper(II) complexes formed by pyridines containing electron donating methyl, phenyl and methoxy substituents in the *p*‐ or *p*‐ and *m*‐positions, isoquinoline (Isoq) or imidazo[1,2‐b]pyridazine (Impdz)^[13]^ as monodentate ligands with triflate or perchlorate counter ions were the most suitable mediators for radiofluorination (Scheme 2, Figures 1 and S13, Tables S18–S22). Thus, ^18^F‐labeling of biphenyl substrates and 4‐Ac‐Ph‐B(OH)_2_ was significantly more efficient if Cu(4‐MeOPy)_4_(OTf)_2_ or Cu(3,4‐Me_2_Py)_4_(OTf)_2_ were used instead of Cu(Py)_4_(OTf)_2_. In the case of 4‐Ac‐Ph‐Bpin on the other hand, differences in the RCCs obtained with different mediators did not reach statistical significance. For all tested Cu complexes, 4‐phenyl‐substituted substrates afforded higher RCCs than 4‐acetyl‐substituted substrates. In the majority of cases, B(OH)_2_ was a slightly better leaving group than Bpin. If Cu(4‐MeOPy)_4_(OTf)_2_ was used as the mediator, RCCs for 4‐Ac‐Ph‐B(OH)_2_ were significantly higher than for 4‐Ac‐Ph‐Bpin. Among the perchlorate complexes, Cu(Impdz)_4_(ClO_4_)_2_ furnished significantly higher RCCs with 4‐Ph‐Ph‐Bpin, 4‐Ac‐Ph‐B(OH)_2_ and 4‐Ac‐Ph‐Bpin precursors as compared to Cu(Py)_4_(OTf)_2_. Copper perchlorate complexes with 4‐methoxypyridine and 3,4‐lutidine ligands were significantly more efficient for radiofluorination of 4‐acetyl‐substituted substrates and aryl boronic acids, respectively. Cu(Isoq)_4_(ClO_4_)_2_ was a significantly better mediator for radiolabeling of 4‐Ph‐Ph‐B(OH)_2_ than Cu(Py)_4_(OTf)_2_, and Cu(Impdz)_4_(OTs)_2_ was at least as effective as the corresponding triflate and perchlorate complexes (Figure 2, Table S23). Noteworthy, among all tested Cu complexes, only Cu(Impdz)_4_(ClO_4_)_2_ enabled the preparation of 4‐[^18^F]F‐Ph‐Ph and 4‐[^18^F]F‐Ac‐Ph from the respective trimethylstannyl precursors in reasonable RCCs (20±4 % and 23±2 %, respectively, Table S24).

Although application of the alternative mediators significantly improved the radiolabeling yields in *n*BuOH/DMA, they remained moderate and did not exceed 58 %. Consequently, the influence of aprotic reaction solvents other than DMA was studied.

Based on an initial solvent screening, propylene carbonate (PC) and 1,3‐dimethyl‐2‐imidazolidinone (DMI) were selected for further evaluation in the following experiments (Figures 1 and S22). For a total of seven precursor/Cu mediator combinations, application of *n*BuOH/PC gave significantly higher labeling yields when compared to those obtained using DMA (Figures S21 and S22, symbol #), whereas in *n*BuOH/DMA no significantly higher ^18^F‐incorporation was observed for any precursor/Cu mediator combination. In particular, application of Cu complexes with 4‐PhPy ligands in *n*BuOH/PC afforded RCCs of 70–86 % for all four model substrates. For the boronic acid substrates, 4‐Ph‐Ph‐ and 4‐Ac‐Ph‐B(OH)_2_, almost quantitative (>98 %) RCCs in *n*BuOH/PC were obtained with Cu(Py)_4_(ClO_4_)_2_ and Cu(Isoq)_4_(ClO_4_)_2_, respectively.

For reactions performed with DMI as reaction solvent, significantly higher RCCs compared to the corresponding reactions with DMA were observed for eight combinations of Cu complex and radiofluorination substrate (Figures S21 and S22, symbol §), while DMA showed no significant advantage over DMI for any of the remaining combinations examined. In particular, in *n*BuOH/DMI, both 3,4‐lutidine complexes allowed radiolabeling of all four model substrates in RCCs ≥80 %. Perchlorate complexes with 4‐MeOPy, 4‐PhPy and Isoq ligands were also efficient mediators for radiofluorination of all model substrates (except for 4‐Ac‐Ph‐Bpin) in DMI, affording labeling efficacies of >75 %. Conventional Cu(Py)_4_(OTf)_2_ in DMI enabled highly efficient preparation of electron‐rich 4‐[^18^F]fluorobiphenyl but not electron‐poor 4‐[^18^F]fluoroacetophenone (RCCs>90 % vs. ≤45 %, respectively), while Cu(Impdz)_4_(ClO_4_)_2_ was an efficient mediator for radiofluorination of both model aryl boronic acids (RCCs >85 %).

Generally, under the conditions examined (aprotic reaction solvent, radiolabeling mediator), 4‐biphenyl precursors often provided significantly higher RCCs than 4‐acetylphenyl substrates (Figure S22, symbols # and § over black and dark grey bars). In the majority of cases, boronic acid substrates and, particularly, 4‐Ac‐Ph‐B(OH)_2_ were more efficient radiolabeling precursors than the corresponding pinacol boronates. Among all tested solvent/precursor combinations, comparison with Cu(Py)_4_(OTf)_2_ showed that triflate complexes were significantly better mediators in 16 cases and significantly worse mediators in 11 cases, while perchlorate complexes were significantly better in 31 cases and significantly worse in two cases (Figure S22 and Tables S29, S31 and S33). Nearly quantitative (>95 %) RCCs were observed in five cases: for radiolabeling of 4‐Ph‐Ph‐B(OH)_2_ with Cu(4‐PhPy)_4_(ClO_4_)_2_ or Cu(Isoq)_4_(ClO_4_)_2_ in DMI, for radiolabeling of 4‐Ph‐Ph‐Bpin with Cu(4‐PhPy)_4_(ClO_4_)_2_ in DMI, as well as for radiolabeling of 4‐Ph‐Ph‐B(OH)_2_ with Cu(Py)_4_(ClO_4_)_2_ or Cu(Isoq)_4_(ClO_4_)_2_ in PC. Notably, in all cases except for 4‐Ph‐Ph‐B(OH)_2_ in *n*BuOH/PC, Cu(Impdz)_4_(ClO_4_)_2_ afforded significantly higher RCCs than the known mediator Cu(Impdz)_4_(OTf)_2_ ^[13]^ (Figure S23, Table S35). Compared to Cu(Py)_4_(OTf)_2_, Cu(4‐PhPy)_4_(ClO_4_)_2_ and Cu(Isoq)_4_(ClO_4_)_2_ delivered significantly higher RCCs in eight and six cases, respectively, whereas Cu(Impdz)_4_(ClO_4_)_2_ afforded significantly higher RCCs in six cases and significantly lower RCCs in one case (Figure S22, Tables S29, S31 and S33).

Direct comparison of PC and DMI as reaction co‐solvents demonstrated that four substrate/Cu complex combinations provided significantly higher RCCs in *n*BuOH/DMI (Figures S21 and S22, symbol $), while only one combination gave significantly higher RCCs in *n*BuOH/PC. Since these observations pointed to DMI as an advantageous reaction solvent in comparison to PC, the former was chosen for further studies. In *n*BuOH/DMI, the highest mean RCCs for four tested substrates were observed with Cu(3,4‐Me_2_Py)_4_(OTf)_2_, Cu(3,4‐Me_2_Py)_4_(ClO_4_)_2_ and Cu(4‐PhPy)_4_(ClO_4_)_2_ (87±7 %, 90±5 % and 87±14 %, respectively). Consequently, these complexes were selected for all further optimization studies.

In the next step, the influence of reaction time and temperature on labeling efficacy was evaluated. Using Cu(4‐PhPy)_4_(ClO_4_)_2_ as the Cu mediator, 4‐Ph‐Ph‐B(OH)_2_ was radiofluorinated in RCCs of 65–80 % over a broad range of reaction temperatures and times (5–20 min at 80–140 °C; Figure S24A). At reaction temperatures of ≥140 °C, an increase in the variability of ^18^F‐incorporation was observed. Reaction times of less than 5 min at 110 °C led to a drop of labeling efficacy (down to 57±4 % and 20±13 % for reaction times of three and one min, respectively; Figure S24B), while RCCs of 54±2 % and 37±7 % were still observed after 10 min at lower temperatures of 70 and 60 °C, respectively. A further increase of the incubation time at these reaction temperatures to 30 min led to RCCs of 82±1 % and 66±8 %, respectively (Figure S24C).

Having established optimized radiofluorination conditions for boronic acids and pinacol boronates, we next extended the procedure to labeling of neopentyl glycol boronates (Bneo) and trimethylstannanes, using 4‐Ph‐Ph‐Bneo, 4‐Ac‐Ph‐Bneo, 4‐Ph‐Ph‐SnMe_3_ and 4‐Ac‐Ph‐SnMe_3_ as model substrates (Table 1). Radiolabeling of both Bneo precursors with Cu(4‐PhPy)_4_(ClO_4_)_2_ as mediator furnished the corresponding ^18^F‐fluorinated compounds in RCCs of 84±1 % and 59±18 % for the 4‐Ph‐ and 4‐Ac‐substituted substrates, respectively. 4‐Phenyl‐ and 4‐acetylphenyltrimethylstannane were radiolabeled in moderate RCCs of up to 29 % and 44 %, respectively. Notably, conventional radiolabeling conditions [10 μmol stannane and Cu(Py)_4_(OTf)_2_ in *n*BuOH/DMA, 10 min at 110 °C] afforded RCCs of only 2–14 %. In our previous work,^[14]^ we already observed that, even though Cu‐mediated radiofluorination of aryl stannanes tolerated alcohols as co‐solvents, no increase of the RCCs was typically observed when using alcohol‐containing media. Applying Cu(4‐PhPy)_4_(ClO_4_)_2_ as the mediator in pure DMI, electron‐rich 4‐Ph‐Ph‐SnMe_3_ was radiolabeled in significantly improved RCCs of 38±2 % (vs. 26±4 % in DMI/*n*BuOH; p=0.0552). Exclusion of *n*BuOH, however, did not improve the RCCs of electron‐deficient 4‐[^18^F]fluoroacetophenone (44±4 % vs. 45±4 %). In the next step, we evaluated the influence of reaction atmosphere (air vs. argon) on the radiolabeling efficiency (see Ref. [15] for a relevant discussion). Use of an argon atmosphere significantly increased RCCs for ^18^F‐fluorination of 4‐Ph‐Ph‐SnMe_3_ to 57±4 % (p=0.0091), while the labeling efficacy of 4‐Ac‐Ph‐SnMe_3_ (47±6 %) remained essentially unaffected. Finally, reduction of the reaction temperature to 90 °C enabled ^18^F‐fluorination of 4‐Ph‐ and 4‐Ac‐Ph‐SnMe_3_ in RCCs of 60±3 and 63±10 %, respectively.

**Table 1:** Radiofluorination of stannyl precursors.

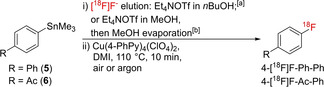				
Entry	Precursor	Elution	Conditions	RCC [%]
1	**5**	*n*BuOH^[a]^	air	20±8
2			argon	22±6
3		MeOH^[b]^	air	38±2
4			argon	57±3
5			argon^[c]^	60±3
6	**6**	*n*BuOH^[a]^	air	44±3
7			argon	33±11
8		MeOH^[b]^	air	45±3
9			argon	47±6
10			argon^[c]^	63±10

Conditions: i) [a] [^18^F]F^−^ (10‐50 MBq) was eluted with a solution of Et_4_NOTf (1 mg, 4 μmol) in *n*BuOH (400 μL) into a solution of **5** or **6** (10 μmol, 1 eq.) and Cu(4‐PhPy)_4_(ClO_4_)_2_ (10 μmol, 1 eq.) in DMI (800 μL) or [b] [^18^F]F^−^ (10–50 MBq) was eluted with a solution of Et_4_NOTf (1 mg, 4 μmol) in MeOH (400 μL), MeOH was evaporated at 60 °C under reduced pressure and in a stream of air or argon within 3 min, and the residue was taken up into a solution of **5** or **6** (10 μmol, 1 eq.) and Cu(4‐PhPy)_4_(ClO_4_)_2_ (10 μmol, 1 eq.) in DMI (800 μL); ii) 110 °C for 10 min and then cooling and addition of H_2_O (1 mL). [c] same as [b] but ii) 90 °C for 10 min. Radiochemical conversions (RCCs) were determined by HPLC. All experiments were carried out at least in triplicate.

To demonstrate the practical suitability of the novel Cu mediators, we next applied them for the preparation of several PET‐tracers: [^18^F]R91150,^[16]^ [^18^F]ALX5407,^[17]^ [^18^F]MNI1126,^[18]^ 3‐(*S*)‐ and (*R*)‐[^18^F]FPhes,^[19]^ and (*S*)‐αMe‐3‐[^18^F]FPhe.^[20]^

**Scheme 3:**
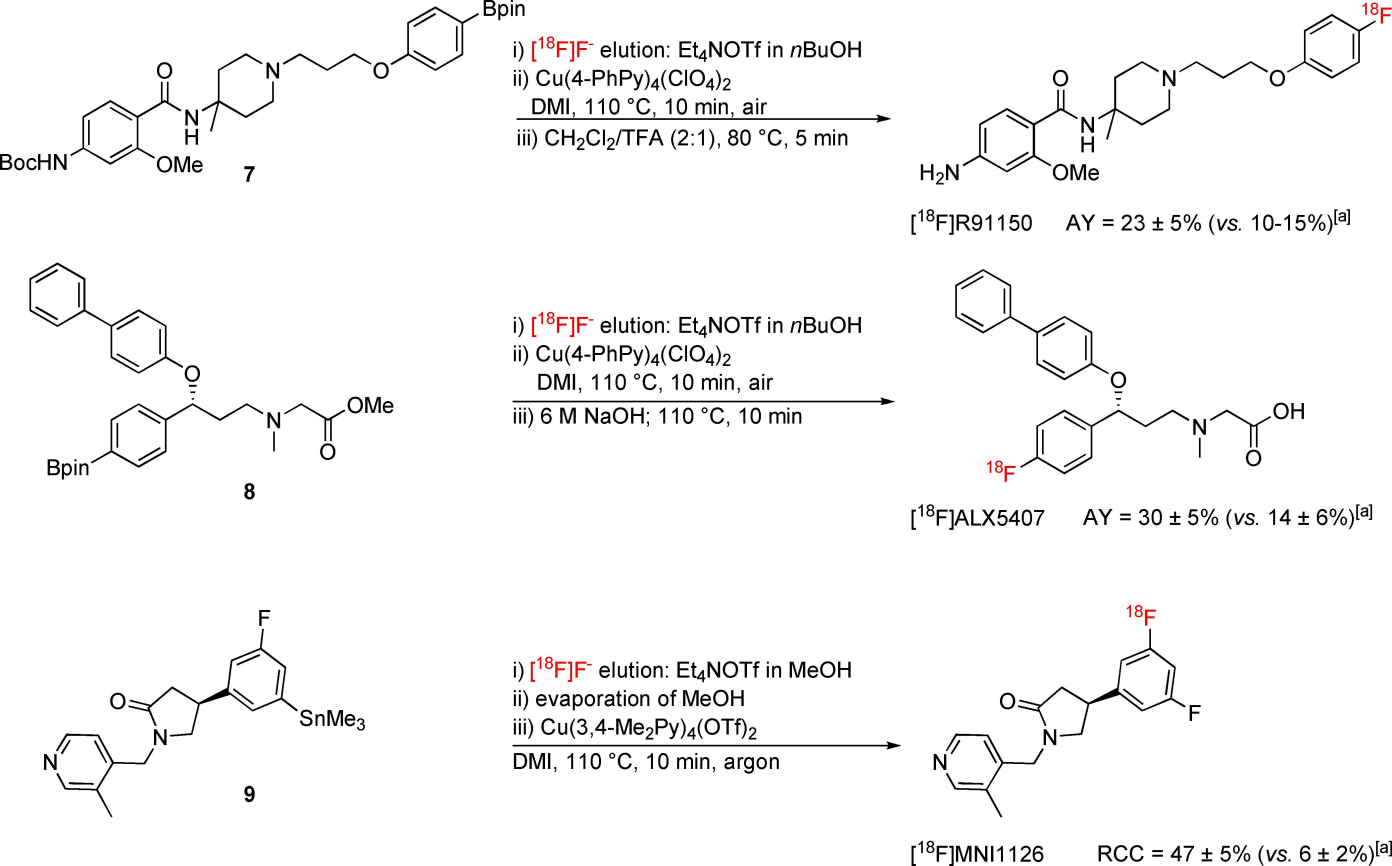
Production of the PET‐tracers [^18^F]R91150, [^18^F]ALX5407 and [^18^F]MNI1126. [a] AY or RCC of radiosynthesis if the ^18^F‐labeling step was performed under standard conditions [Cu(Py)_4_(OTf)_2_ in nBuOH/DMA]. All radiosyntheses were carried out in triplicate. AY – activity yield; RCC – radiochemical conversion.

Radiosynthesis of [^18^F]R91150, a promising 5‐HT_2A_‐selective PET‐tracer,^[16b]^ from the corresponding Bpin precursor **7** was performed according to the developed protocol using Cu(4‐PhPy)_4_(ClO_4_)_2_ as the mediator. After subsequent deprotection and HPLC purification, the tracer was obtained in activity yields (AYs)^[11]^ of 23±5 % (Scheme 3), as compared to AYs of 10–15 % obtained under standard conditions [Cu(Py)_4_(OTf)_2_ in DMA/*n*BuOH]. Application of the method for preparation of [^18^F]ALX5407, a glycine transporter type 1 (GlyT_1_) radioligand,^[17]^ from the appropriate Bpin precursor **8** using Cu(4‐PhPy)_4_(ClO_4_)_2_ improved AYs (30±5 % vs. 14±6 %), allowed for a reduction of precursor and mediator amounts from 30 to 10 μmol, and thus simplified preparative HPLC purification of the radiolabeled product (Scheme 3).

[^18^F]MNI1126, a specific PET‐probe for the ubiquitous synaptic vesicle glycoprotein 2A (SV2A),^[18]^ was most efficiently prepared from the corresponding stannyl precursor **9**. In this case, application of Cu(3,4‐Me_2_Py)_4_(OTf)_2_ as radiolabeling mediator and DMI as reaction solvent furnished the tracer in RCCs of 47±5 % (Scheme 3), as compared to RCCs of 6±2 % under standard conditions [Cu(Py)_4_(OTf)_2_ in DMA].

**Scheme 4:**
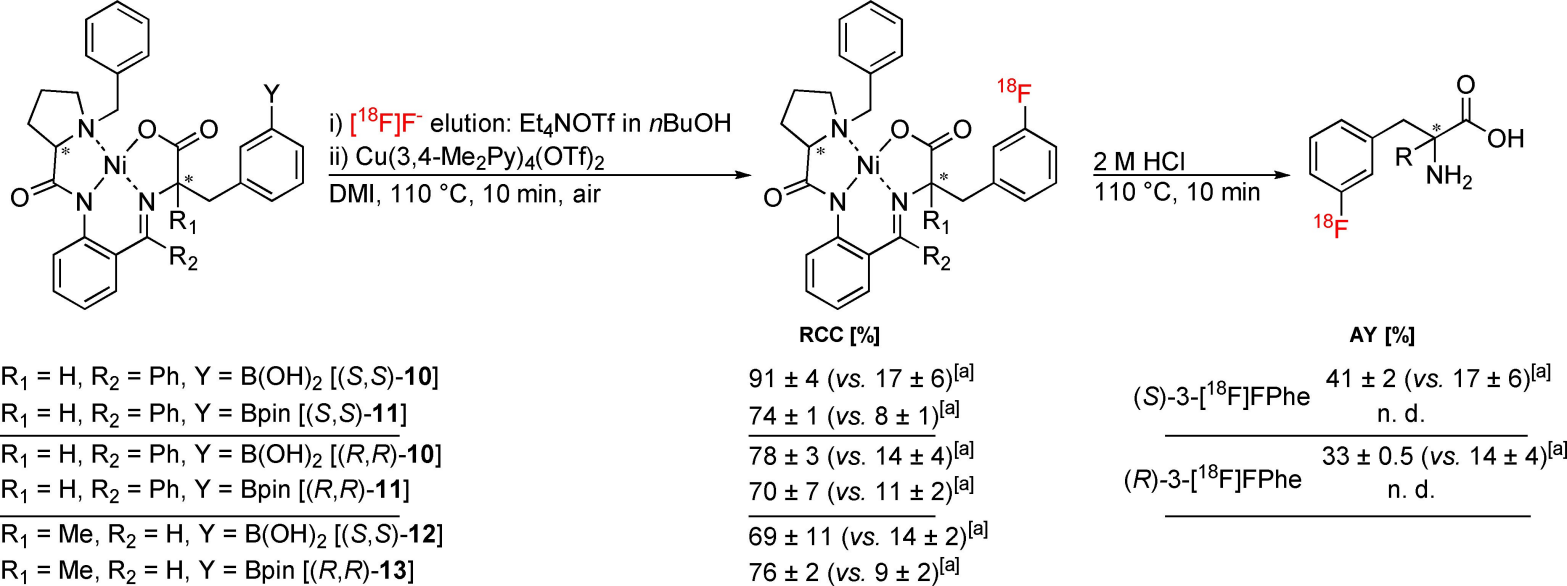
Preparation of 3‐[^18^F]FPhes and protected αMe‐3‐[^18^F]FPhes using the novel radiolabeling protocol. [a] AY or RCC of radiosynthesis if the ^18^F‐labeling step was performed under standard conditions [Cu(Py)_4_(OTf)_2_ in *n*BuOH/DMA]. AY – activity yield; RCC – radiochemical conversion.

^18^F‐Labeled fluorophenylalanines ([^18^F]FPhes) and α‐methyl‐fluorophenylalanines (αMe‐FPhes) are promising PET probes for the visualization of increased amino acid uptake associated with increased protein synthesis rates in cerebral and peripheral tumors.[^19^, ^21^] We recently published a procedure for preparation of these and other radiolabeled aromatic amino acids via alcohol‐enhanced Cu‐mediated radiofluorination of Bpin‐substituted chiral complexes using Ni/Cu‐BPB/BPA templates as double protecting groups.^[20]^ Application of 30 μmol Bpin precursor and 60 μmol Cu(Py)_4_(OTf)_2_ in *n*BuOH/DMA delivered the corresponding ^18^F‐labeled complexes in good to excellent RCCs, but downscaling of the precursor/Cu mediator amounts led to a sharp decrease of the conversions. This problem could be circumvented by elution of [^18^F]F^−^ from an anion exchange resin with Et_4_NHCO_3_ in MeOH, followed by evaporation of MeOH and addition of the radiolabeling precursor and Cu(Py)_4_(OTf)_2_ in *n*BuOH/DMA. However, complete omission of all evaporation steps would further reduce the synthesis time and simplify transfer of the method to an automated synthesis module. With this in mind, we applied the procedure for radiolabeling of the corresponding Bpin‐ and B(OH)_2_‐substituted Ni(II)‐BPX complexes (Scheme 4). The latter were prepared by transesterification of the appropriate Bpin boronates with MeB(OH)_2_.^[22]^ Using 10 μmol precursor and Cu(3,4‐Me_2_Py)_4_(OTf)_2_ as the Cu mediator, (*S*,*S*)‐ and (*R*,*R*)‐Ni‐BPB‐3‐[^18^F]FPhes as well as (*S*,*S*)‐Ni‐BPA‐αMe‐3‐[^18^F]FPhe were prepared in RCCs of 69–91 %. Subsequent hydrolysis, HPLC isolation and formulation furnished (*S*)‐ and (*R*)‐3‐[^18^F]FPhe from the respective boronic acid precursors as solutions ready for application in AYs of 43±3 (*n*=3) and 33±1 % (*n*=3), respectively. Molar activity amounted to 538 GBq/μmol for 1.8 GBq (*S*)‐3‐[^18^F]FPhe (from 4 GBq [^18^F]F^−^).

The developed protocol was also successfully implemented into a remotely controlled synthesis unit (AllInOne, Trasis), enabling the automated preparation of (*S*)‐3‐[^18^F]FPhe in AYs of 18±2 % (*n*=3) within 70 min.

**Scheme 5:**
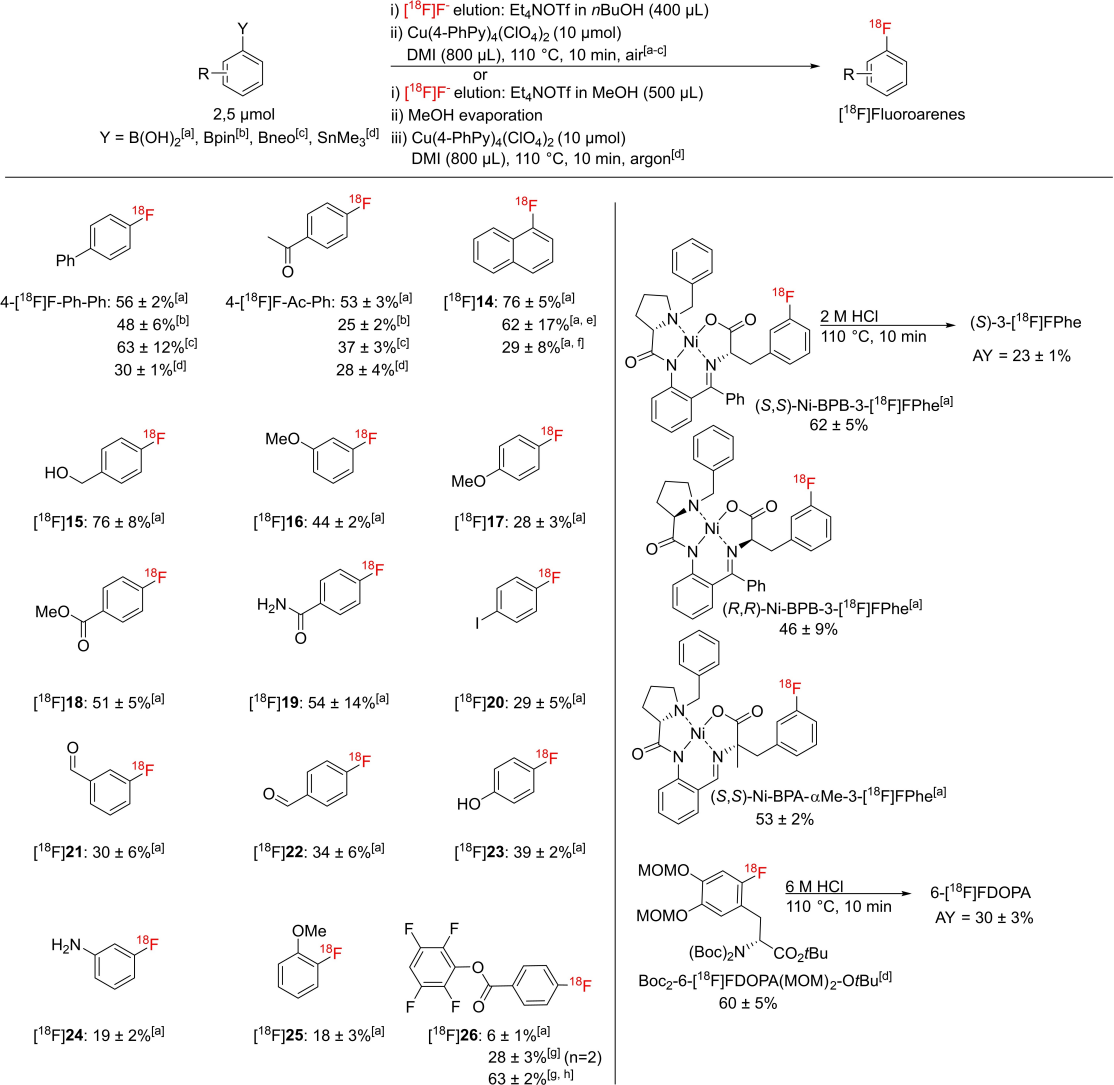
Substrate scope of the developed protocol using 2.5 μmol substrate loading. Radiolabeled products were prepared from the respective [a] aryl boronic acid, [b] pinacol boronate, [c] neopentyl glycol boronate or [d] trimethyl stannyl precursors. [e] Prepared from 1 μmol precursor. [f] Prepared from 0.5 μmol precursor. [g] Radiosyntheses were performed in pure DMI and elution of [^18^F]F^−^ with Et_4_NOTf in MeOH followed by evaporation of MeOH at 60 °C in a stream of argon. [h] Prepared from 10 μmol precursor. If not otherwise stated, mean RCCs±standard deviations are provided and all experiments were carried out at least in triplicate. AY – activity yield; RCC – radiochemical conversion.

The improved performance of the procedure raised the question whether even lower precursor loadings could be efficiently applied. To address this, we first evaluated radiolabeling of 2.5 μmol 4‐Ph‐Ph‐B(OH)_2_ in 1.2 mL *n*BuOH/DMI (1 : 2) using Et_4_NOTf (1 mg, 4 μmol) for [^18^F]F^−^ elution and 10 μmol Cu(Py)_4_(OTf)_2_, Cu(3,4‐Me_2_Py)_4_(OTf)_2_, Cu(3,4‐Me_2_Py)_4_(ClO_4_)_2_ or Cu(4‐PhPy)_4_(ClO_4_)_2_ as labeling mediator at 110 °C for 10 min (Scheme 5, Figure S60, Tables S50 and S51). Under these conditions, Cu(4‐PhPy)_4_(ClO_4_)_2_ was significantly more efficient than the other Cu complexes examined and afforded 4‐[^18^F]F‐Ph‐Ph in satisfactory RCCs of 56±2 %, so that it was selected for further experiments. Next, 2.5 μmol scale radiolabeling reactions with different 4‐Ph‐ and 4‐Ac‐substituted boronic compounds and stannanes were evaluated (Scheme 5, Tables S52 and S53). Radiolabeling of boronic substrates was carried out in *n*BuOH/DMI (1 : 2; 1.2 mL) and of stannyl precursors in DMI (0.8 mL). Whereas RCCs for both B(OH)_2_ substrates and 4‐Ph‐Ph‐Bneo were comparable (53–63 %), they were lower for the other precursors examined (25–48 %).

Next, the procedure was applied for 2.5 μmol scale radiolabeling of fifteen additional aryl boronic acids. Thus, electron‐rich and electron‐poor ^18^F‐fluorinated aromatics including unprotected 4‐[^18^F]fluoro‐aniline and ‐phenol were synthesized in RCCs of 18–76 % (Scheme 5). Noteworthy, 1.0 and 0.5 μmol of 1‐naphthyl boronic acid were sufficient to obtain 1‐[^18^F]fluoronaphthalene in RCCs of 62±17 % and 29±8 %, respectively. The sensitive radiofluorinated active ester [^18^F]**26** ^[4b]^ was also successfully prepared in RCCs of 28±3 % (in pure DMI; 63±2 % RCCs from 10 μmol precursor; 6±1 % RCCs in DMI/*n*BuOH). Strikingly, 2.5 μmol of Ni‐BPB‐3‐B(OH)_2_Phes as well as (*S*,*S*)‐Ni‐BPA‐αMe‐3‐B(OH)_2_Phe were sufficient to achieve RCCs of 46–62 %. Exemplarily, hydrolysis of (*S*,*S*)‐Ni‐BPB‐3‐[^18^F]FPhe followed by HPLC purification furnished (*S*)‐3‐[^18^F]FPhe in 23±1 % AYs.

Finally, we utilized the procedure for a 2.5 μmol scale preparation of 6‐[^18^F]fluoro‐3,4‐dihydroxyphenylalanine (6‐[^18^F]FDOPA; Scheme 5). 6‐[^18^F]FDOPA is widely used for measuring the integrity and function of the nigrostriatal dopaminergic system, for example, in Parkinson's disease^[23]^ as well as for the detection and staging of neuroendocrine tumors.^[24]^ Using conventional Cu(Py)_4_(OTf)_2_, 6‐[^18^F]FDOPA was previously prepared from 5 μmol of Boc_2_‐6‐Bpin‐DOPA(MOM)_2_‐O*t*Bu in AYs of 6±1 %.^[25]^ Radiofluorination of 2.5 μmol of Boc_2_‐6‐Bpin‐DOPA(MOM)_2_‐OMe precursor with Cu(4‐PhPy)_4_(ClO_4_)_2_ as mediator furnished protected 6‐[^18^F]FDOPA in <20 % RCC. In contrast, for the corresponding stannyl precursor, Boc_2_‐6‐Me_3_Sn‐DOPA(MOM)_2_‐OMe, higher RCCs of 60±5 % were observed. Interestingly, in this case, the reduction of the precursor amount from 10 to 2.5 μmol did not lead to a decrease of the RCCs (58±4 vs. 60±5 %). Subsequent deprotection and HPLC isolation delivered the desired tracer in good AYs of 30±3 %.

In summary, based on the results of the present study, the following general recommendations can be made for Cu‐mediated radiofluorination of aromatic boronyl and stannyl substrates:

*n*BuOH/DMI is the solvent of choice for radiolabeling of boronyl substrates while pure DMI is well suited for radiolabeling of stannyl substrates;
Boronic acids are frequently better radiofluorination substrates then the corresponding BPin esters;
For ^18^F‐fluorinations performed with 10 μmol of the radiolabeling precursor, Cu(4‐PhPy)_4_(ClO_4_)_2_, Cu(3,4‐Me_2_Py)_4_(OTf)_2_ and Cu(3,4‐Me_2_Py)_4_(ClO_4_)_2_ are the most efficient mediators;
At 2.5 μmol substrate loading, Cu(4‐PhPy)_4_(ClO_4_)_2_ is the preferred mediator;
Radiofluorination of boronyl substrates should be carried out under air; while stannyl precursor are preferably radiofluorinated under argon;
Thermolabile substrates can be efficiently radiolabeled at 70–90 °C.

## Conclusion

In conclusion, our screening study led to the discovery of several highly efficient mediators for Cu‐mediated radiofluorination like Cu(4‐PhPy)_4_(ClO_4_)_2_, Cu(3,4‐Me_2_Py)_4_(OTf)_2_ and Cu(3,4‐Me_2_Py)_4_(ClO_4_)_2_. Especially in DMI or *n*BuOH/DMI as reaction media, these copper complexes enabled highly efficient ^18^F‐labeling of different boronic and stannyl substrates. The optimized protocol worked well at reaction temperatures in the range of 60–90 °C, indicating its suitability for radiofluorination of thermolabile substrates. The practicality of the method was demonstrated by preparation of several PET‐tracers, including 6‐[^18^F]FDOPA, in improved radiochemical yields and/or using significantly lower precursor loadings. Furthermore, the procedure was successfully implemented into a remote‐controlled synthesis unit, highlighting its applicability for GMP‐compliant production of clinically relevant PET‐tracers.

## Experimental Section

**Chemistry**: Detailed information on the synthesis of metal complexes and radiolabeling precursors as well as the corresponding analytical data (^1^H, ^13^C, LR‐MS‐ESI, HR‐MS‐ESI, elemental analysis, IR and crystallographic data) are provided in the Supporting Information.

**Radiochemistry**: Detailed descriptions of radiolabeling procedures, optimization studies, statistical evaluation and PET‐tracer syntheses with representative HPLC analytic and quality controls are provided in the Supporting Information.

**Processing of fluoride‐18**: Aqueous [^18^F]F^−^ was loaded onto a QMA cartridge (preconditioned with 1 mL H_2_O) from the female side to the male side. The cartridge was washed (from the male side) with anhydrous MeOH (1 mL) to remove residual H_2_O and dried (from the female side) with air (2×10 mL). [^18^F]F^−^ was eluted (from the female to the male side) with a solution of Et_4_NOTf in *n*BuOH or MeOH.

**Optimized procedure for radiofluorination of boranyl precursors**: [^18^F]F^−^ (500 μL, 20–5000 MBq) was loaded onto a QMA cartridge and eluted with a solution of Et_4_NOTf (1 mg, 3.6 μmol) in *n*BuOH (400 μL) into a solution of the respective Cu complex and precursor (10 μmol of each or 10 μmol of mediator and 2.5 μmol of boranyl substrate) in DMI (800 μL). The reaction mixture was heated at 110 °C for 10 min in atmospheric or synthetic air, cooled to ambient temperature and diluted with H_2_O (2 mL). RCCs were determined by radio‐HPLC. PET‐tracers were isolated by preparative HPLC and formulated as ready‐to‐use solutions. At high atmospheric humidity (e. g., during the midsummer), a significant drop of the RCCs was sometimes observed, presumably owing to the hygroscopicity of Et_4_NOTf. In such cases, [^18^F]F^−^ should preferably be eluted with a solution of Et_4_NOTf in MeOH. MeOH was removed at 60 °C for 2–3 min under reduced pressure in a stream of argon and the residue was taken up into a solution of the respective Cu mediator and precursor (10 μmol of each if not otherwise noted) in a mixture of the corresponding solvent and *n*BuOH (1200 μL; 2 : 1).

**Optimized procedure for radiofluorination of stannyl precursors**: [^18^F]F^−^ was loaded onto a QMA carbonate cartridge and eluted with Et_4_NOTf (1 mg, 3.6 μmol) in MeOH (500 μL) into a V–Vial as described above, followed by evaporation of MeOH at 60 °C under reduced pressure in a stream of argon. The V‐Vial was filled with argon, sealed with a silicon septum and a solution of the appropriate precursor and copper complex (10 μmol of each if not otherwise noted) in DMI (800 μL) was added via a cannula through the septum. The reaction mixture was heated at 90 or 110 °C for 10 min, cooled to ambient temperature and diluted with H_2_O (2 mL). RCCs were determined by radio‐HPLC. PET‐tracers were isolated by preparative HPLC and formulated as ready‐to‐use solutions.

**Crystal data**: Deposition Numbers 2202867 [for Cu(Py)_4_(OTf)_2_], 2202868 [for Cu(4‐MeOPy)_4_(ClO_4_)_2_] and 2202858 [for Cu(3,4‐Me_2_Py)_4_(OTf)_2_] contain the supplementary crystallographic data for this paper. These data are provided free of charge by the joint Cambridge Crystallographic Data Centre and Fachinformationszentrum Karlsruhe Access Structures service.

## Conflict of interest

The authors declare no conflict of interest.

1

## Supporting information

As a service to our authors and readers, this journal provides supporting information supplied by the authors. Such materials are peer reviewed and may be re‐organized for online delivery, but are not copy‐edited or typeset. Technical support issues arising from supporting information (other than missing files) should be addressed to the authors.

Supporting Information

## Data Availability

The data that support the findings of this study are available from the corresponding author upon reasonable request.
