# Supplementary material for: Next Generation Copper Mediators for the Efficient Production of 18F‐Labeled Aromatics
Source: Chemistry. 2022 Nov 17;29(2):e202202965. doi: 10.1002/chem.202202965 (PMC10100267; doi:10.1002/chem.202202965)
Supplement: Supplementary file 1 — Supporting Information [file CHEM-29-0-s001.pdf]

Chemistry–A European Journal

Supporting Information

Next Generation Copper Mediators for the Efficient Production of ^{18}F-Labeled Aromatics

Chris Hoffmann, Niklas Kolks, Daniel Smets, Alexander Haseloer, Benedikt Gröner, Elizaveta A. Urusova, Heike Endepols, Felix Neumaier, Uwe Ruschewitz, Axel Klein, Bernd Neumaier,* and Boris D. Zlatopolskiy

1 Materials and methods 3
1.1 General 3
1.2 Nuclear magnetic resonance (NMR) spectroscopy 3
1.3 Mass spectrometry (MS) 3
1.4 Infrared spectroscopy (IR) 3
1.5 Elemental analysis 4
1.6 Column Chromatography. 4
1.7 Thin layer chromatography (TLC) 4
2 Chemistry 4
2.1 Preparation of Ni(II), Co(II) and Cu(II) complexes – General Procedure 1 (GP1) 4
2.2 Crystallography 14
2.3 Preparation of aryl trimethylstannanes – General Procedure 2 (GP2) 29
2.4 Preparation of aryl neopentyl glycol boronates – General Procedure 3 (GP3) 32
2.5 Preparation of $(Boc)_2$-6-BpinDOPA$(MOM)_2$-OMe (S4) 35
2.6 Preparation of $(Boc)_2$-6-$SnMe_3$DOPA$(MOM)_2$-OMe (S6) 43
2.7 Preparation of $B(OH)_2$ substituted Ni(II)-BPX complexes – General Procedure 4 (GP4) 45
3 Radiochemistry 50
3.1 General conditions 50
3.2 Analytical HPLC 50
3.3 Preparative HPLC 53
3.4 Processing of fluoride-18 53
3.5 Statistical analysis 54
3.6 General procedures for radiolabeling 54
3.7 Optimization studies 56
3.8 Radiolabeling of model stannyl precursors 5 and 6 99
3.9 Radiosynthesis of $[^{18}F]$R91150 107
3.10 Radiosynthesis of $[^{18}F]$ALX5407 109
3.11 Radiosynthesis of $[^{18}F]$MNI1126 111
3.12 Preparation of 3-$[^{18}F]$FPhes and (*S*)-αMe-3-$[^{18}F]$FPhe – General Procedure (GP8) 113
3.13 Automated radiosynthesis of 3-(*S*)-$[^{18}F]$FPhe in an AllInOne synthesis module (Trasis) .. 120
3.14 Radiolabeling at 2.5 µmol precursor loading. Initial experiments 123
3.15 Preparation of 6-$[^{18}F]$FDOPA 140
4 References 142

1 Materials and methods

1.1 General

Unless otherwise stated, all reagents and solvents were purchased from Sigma-Aldrich (Steinheim, Germany), Acros (Fisher Scientific GmbH, Nidderrau, Germany), Alfa Aesar [Thermo Fisher (Kandel) GmbH, Kandel, Germany], BLDPharm (Kaiserslautern, Germany) or Key Organics (Camelford, UK), and used without further purification. Unless otherwise stated, all reactions were carried out with magnetic stirring and, if air or moisture sensitive substrates and/or reagents were handled, in flame-dried glassware under argon. Organic extracts were dried over anhydrous Na_2SO_4 or $MgSO_4$. Solutions were concentrated under reduced pressure (1–900 mbar) at 40–50 °C using a rotary evaporator. Quinoline for the synthesis of copper complexes was purified by vacuum distillation over Na_2SO_4 and stored under argon. Compounds **7**,[1] **8**,[2] **9**,[3] (*S*,*S*)- and (*R*,*R*)-**11**[4] and (*S*,*S*)-**13**,[4] as well as Boc-DOPA-OMe,[5] were prepared according to the literature.

1.2 Nuclear magnetic resonance (NMR) spectroscopy

Proton and carbon nuclear magnetic resonance (^{1}H and ^{13}C NMR) spectra were recorded on a Bruker Avance Neo (400 MHz) spectrometer. Chemical shifts are reported in parts per million (ppm) relative to residual peaks of deuterated solvents. The observed signal multiplicities are characterized as follows: s = singlet, d = doublet, t = triplet, m = multiplet and br = broad. Coupling constants (*J*) are reported in hertz (Hz).

1.3 Mass spectrometry (MS)

Low resolution mass spectra (LR-MS) were measured with an MSQ PlusTM mass spectrometer (Thermo Electron Corporation, San Jose, USA).

High resolution mass spectra (HR-MS) were measured with an LTQ Orbitrap XL (Thermo Fischer Scientific Inc., Bremen, Germany).

1.4 Infrared spectroscopy (IR)

Infrared spectra were recorded as the neat compound using PerkinElmer UATR Two [PerkinElmer LAS (Germany) GmbH, Rodgau, Germany]. Absorptions were reported as wavenumbers (cm^{-1}).

1.5 Elemental analysis

Elemental analyses of the copper complexes were performed by HEKAtech GmbH (Wegberg, Germany).

1.6 Column Chromatography.

Merck silica gel, grade 60, 230–400 mesh, (Merck KGaA, Darmstadt, Germany) was used for column chromatography. Bpin and Me_3Sn derivatives were chromatographed on Sigma-Aldrich silica gel 60 Å, 230–400 mesh, with ca. 0.1% Ca (Sigma-Aldrich, Taufkirchen, Germany). For automated flash chromatography, a Grace Reveleris iES flash chromatography system equipped with RevealX detector, allowing for multisignal (UV/ELSD) collection (Büchi Labortechnik GmbH, Essen, Germany), and Büchi FlashPure (40 µm SiO_2) or Büchi FlashPure Select C_{18} (30 µm, spherical) cartridges (Büchi Labortechnik GmbH, Essen, Germany) were employed. Solvent proportions are indicated in a volume/volume ratio.

1.7 Thin layer chromatography (TLC)

Thin layer chromatography (TLC) was performed using precoated sheets, 0.25 mm Sil G/UV254 from Merck KGaA (Darmstadt, Germany). The chromatograms were visualized under UV light (λ = 254 nm) and/or using either phosphomolybdic acid or $KMnO_4$ stain solutions.

2 Chemistry

2.1 Preparation of Ni(II), Co(II) and Cu(II) complexes – General Procedure 1 (GP1)

The respective Cu, Ni or Co salt (1 eq.) was dissolved in hot *i*PrOH (0.2 M) and *N*-heteroarene (6 eq.) in isopropanol (2 M) was slowly added to the resulting solution. The reaction mixture was allowed to cool to ambient temperature and stirred for another 1 h. The products typically precipitated spontaneously. If no spontaneous precipitation occurred, Et_2O was added until the product had precipitated completely. The solid was filtered off and dried under reduced pressure.

$Co(Py)_4(ClO_4)_2·3H_2O$[6]

The title compound was prepared according to GP1 from $Co(ClO_4)_2·6H_2O$ (1.0 g, 2.8 mmol, 1 eq.) and pyridine (1.4 mL, 1.3 g, 17 mmol, 6 eq.). The product was obtained as a light red

solid (0.83 g, 1.3 mmol, 47%). IR (ATR): ν = 3391, 1643, 1604, 1489, 1445, 1220, 1126, 1088, 1066, 1042, 1009, 987, 953, 930, 885, 755, 703, 655, 623, 461. Elemental analysis calcd (%) for $C_{20}H_{26}CoCl_2N_4O_{11}$: C 38.23, H 4.17, N 8.92; found C 38.79, H 4.32, N 9.11.

$Ni(Py)_4(OTf)_2{\cdot}H_2O$[6]

Anhydrous $Ni(OTf)_2$ (50 mg, 140 µmol, 1 eq.) was suspended in MeOH/H_2O (20 mL, 1:4) and heated gently until the solution became clear. The solvent was removed, and the residue was taken up in 2,2-dimethoxypropane (2 mL). A solution of pyridine (100 µL, 98 mg, 1.2 mmol, 86 eq.) in 2,2-dimethoxypropane (1 mL) was added and a light blue solid precipitated. The solvent was decanted, and the solid was washed with hexane and dried under high vacuum. The title compound was obtained as light blue solid (72 mg, 104 µmol, 74%). IR (ATR): ν = 1605, 1447, 1309, 1233, 1224, 1217, 1180, 1161, 1070, 1033, 1013, 764, 758, 710, 701, 653, 631, 581, 568, 511. Elemental analysis calcd (%) for $C_{22}H_{22}NiF_6N_4O_7S_2$: C 38.23, H 3.21, N 8.11; found C 37.92, H 3.00, N 7.95.

$Cu(Py)_4(OTf)_2$[7]

The title compound was prepared according to GP1 from $Cu(OTf)_2$ (0.50 g, 1.4 mmol, 1 eq.) and pyridine (0.68 mL, 0.67 g, 8.4 mmol, 6 eq.). The product was obtained as a blue solid (0.83 g, 1.2 mmol, 86%). Crystals for structural analysis were obtained by slow evaporation of a diluted solution of $Cu(Py)_4(OTf)_2$ in MeOH at ambient temperature. IR (ATR): ν = 1609, 1489, 1453, 1447, 1290, 1241, 1225, 1156, 1087, 1070, 1031, 1019, 989, 956, 759, 699, 654, 633, 572, 516. Elemental analysis calcd (%) for $C_{22}H_{20}CuF_6N_4O_6S_2$: C 38.97, H 2.97, N 8.26; found C 38.80, H 3.12, N 8.21.

$Cu(Py)_4(ClO_4)_2$[8]

The title compound was prepared according to GP1 from $Cu(ClO_4)_2{\cdot}6H_2O$ (2.0 g, 5.5 mmol, 1 eq.) and pyridine (2.7 mL, 2.65 g, 33 mmol, 6 eq.). The product was obtained as a light violet solid (3.2 g, 4.6 mmol, 85%). IR (ATR): ν = 1609, 1491, 1447, 1238, 1221, 1162, 1109, 1070, 1052, 1043, 1016, 1006, 985, 957, 932, 882, 757, 693, 640, 620. Elemental analysis calcd (%) for $C_{20}H_{20}CuCl_2N_4O_8$: C 41.50, H 3.48, N 9.68; found C 40.80, H 3.45, N 9.49.

$Cu(Py)_2Cl_2$[9]

The title compound was prepared according to GP1 from $CuCl_2$ (1.0 g, 5.9 mmol, 1 eq.) and pyridine (2.8 mL, 2.85 g, 35 mmol, 6 eq.). The product was obtained as a blue solid (1.6 g,

3.5 mmol, 93%). IR (ATR): ν = 3223, 3067, 3042, 3028, 3007, 1606, 1491, 1449, 1366, 1241, 1220, 1153, 1080, 1065, 1044, 1018, 873, 760, 687, 644. Elemental analysis calcd (%) for $C_{10}H_{10}CuCl_2N_2$: C 41.04, H 3.44, N 9.57; found C 40.47, H 3.24, N 9.34.

$Cu(Py)_2Br_2$[10]

The title compound was prepared according to GP1 from $CuBr_2$ (1.0 g, 4.5 mmol, 1 eq.) and pyridine (2.2 mL, 2.17 g, 27 mmol, 6 eq.). The product was obtained as a green solid (216 mg, 0.4 mmol, 13%). IR (ATR): ν = 3113, 3067, 3044, 3027, 3005, 1605, 1490, 1447, 1364, 1220, 1153, 1079, 1043, 1017, 945, 868, 755, 686, 651, 643. Elemental analysis calcd (%) for $C_{10}H_{10}CuBr_2N_2$: C 31.48, H 2.64, N 7.34; found C 30.57, H 2.54, N 7.04.

$Cu(Py)_4(ClO_3)_2$

$Cu(ClO_3)_2$[11] was prepared as follows. $CuSO_4$ (300 mg, 1.2 mmol, 1 eq.) was suspended in MeOH/H_2O (2:1, 30 mL) and heated under reflux until a clear blue solution was obtained. A solution of $Ba(ClO_3)_2$ (380 mg, 1.2 mmol, 1 eq.) in H_2O (3 mL) was added dropwise to the hot reaction mixture. After cooling to room temperature, the precipitated white solid was filtered off, washed with MeOH and the filtrate was concentrated under reduced pressure to afford hydrated $Cu(ClO_3)_2$ as a green solid. $Cu(ClO_3)_2$ was taken up in MeOH (20 mL) and pyridine (0.6 mL, 0.59 g, 7.2 mmol, 6 eq.) was added slowly at 80 °C. Thereafter, the reaction mixture was cooled to room temperature, the volume was reduced by half under reduced pressure and the reaction mixture was placed into the fridge for 16 h. The resulting precipitate was filtered off to afford the title compound as a violet solid (133 mg, 0.2 mmol, 20%). IR (ATR): ν = 3101, 3048, 3030, 1604, 1491, 1449, 1223, 1071, 1043, 994, 953, 902, 819, 777, 760, 707, 697, 640, 600, 472. Elemental analysis calcd (%) for $C_{20}H_{20}CuCl_2N_4O_6$: C 43.93, H 3.69, N 10.25; found C 43.85, H 3.69, N 10.12.

$Cu(Py)_4(OTs)_2{\cdot}H_2O$

The title compound was prepared according to GP1 from $Cu(OTs)_2$ (0.50 g, 1.2 mmol, 1 eq.) and pyridine (0.6 mL, 0.57 g, 7.2 mmol, 6 eq.). The product was obtained as a light blue solid (0.76 g, 1.1 mmol, 86%). IR (ATR): ν = 1607, 1450, 1232, 1220, 1172, 1162, 1150, 1117, 1071, 1045, 1030, 1006, 818, 767, 697, 679, 651, 642, 562, 550. Elemental analysis calcd (%) for $C_{34}H_{34}CuN_4O_7S_2$: C 55.16, H 4.90, N 7.57; found C 55.62, H 4.75, N 7.35.

Cu(Py)$_4$(OMs)$_2$

The title compound was prepared according to GP1 from Cu(OMs)$_2$·2H$_2$O (1.0 g, 3.5 mmol, 1 eq.) and pyridine (1.7 mL, 1.6 g, 21 mmol, 6 eq.). The product was obtained as a blue solid (1.7 g, 3.0 mmol, 86%). IR (ATR): ν = 1606, 1490, 1448, 1230, 1220, 1206, 1181, 1153, 1145, 1081, 1072, 1042, 1016, 766, 760, 699, 689, 639, 551, 526. Elemental analysis calcd (%) for $C_{22}H_{26}CuN_4O_6S_2$: C 46.35, H 4.60, N 9.83; found C 46.98, H 4.44, N 10.27.

[Cu(Py)$_3$SO$_4$)]$_2$[12]

Anhydrous pyridine (12.0 mL, 11.8 g, 149 mmol, 12 eq.) was added to anhydrous $CuSO_4$ (2.00 g, 12.5 mmol, 1 eq.) and the resulting blue suspension was vigorously stirred at 100 ºC for 16 h. *i*PrOH (30 mL) was added to the reaction mixture and stirring was continued at 70 ºC for 1 h. The resulting suspension was cooled to ambient temperature and filtered. The filter cake was washed with *i*PrOH (80 mL) followed by Et$_2$O (80 mL) and dried to afford the title compound as a blue solid (5.04 g, 12.7 mmol, >99%). IR (ATR): ν = 1449, 1130, 1100, 1083, 1071, 1054, 1041, 1021, 1014, 990, 789, 777, 765, 756, 713, 698, 639, 618, 603, 559. Elemental analysis calcd (%) for $C_{15}H_{15}CuN_3O_4S$: C 45.39, H 3.81, N 10.59; found C 45.90, H 4.33, N 10.62.

[Cu(Py)(OAc)$_2$]$_2$[13,14]

Anhydrous pyridine (8.0 mL, 7.9 g, 99 mmol, 9 eq.) was added to anhydrous Cu(OAc)$_2$ [2.0 g, 11 mmol, 1 eq.; prepared by drying of Cu(OAc)$_2$·H$_2$O at 140 ºC for 4 h] and the resulting blue suspension was vigorously stirred at 70 ºC for 1 h. *i*PrOH (20 mL) was added to the reaction mixture and stirring was continued for 4 h. The resulting green suspension was cooled to ambient temperature and filtered. The filter cake was washed with *i*PrOH (80 mL) followed by Et$_2$O (80 mL) and dried to afford the title compound as a green solid (2.54 g, 9.7 mmol ,88%). IR (ATR): ν = 1611, 1597, 1573, 1484, 1445, 1424, 1361, 1350, 1236, 1217, 1153, 1082, 1070, 1049, 1037, 1008, 764, 704, 681, 627. Elemental analysis calcd (%) for $C_9H_{11}CuNO_4$: C 41.46, H 4.25, N 5.37; found C 41.41, H 4.29, N 5.32.

Cu(4-PhPy)$_4$(OTf)$_2$[15]

The title compound was prepared according to GP1 from Cu(OTf)$_2$ (1.9 g, 5.4 mmol, 1 eq.) and 4-phenylpyridine (5.0 g, 32 mmol, 6 eq.). The product was obtained as a blue solid (5.2 g, 5.3 mmol, 98%). IR (ATR): ν = 1615, 1421, 1289, 1256, 1241, 1228, 1156, 1072, 1030, 1014, 844, 767, 735, 699, 636, 625, 574, 565, 516, 491. Elemental analysis calcd (%) for $C_{46}H_{36}CuF_6N_4O_6S_2$: C 56.24, H 3.69, N 5.70; found C 55.8, H 3.73, N 5.67.

Cu(4-PhPy)$_4$(ClO$_4$)$_2$

The title compound was prepared according to GP1 from Cu(ClO$_4$)$_2$·6H$_2$O (1.0 g, 2.7 mmol, 1 eq.) and 4-phenylpyridine (2.5 g, 16 mmol, 6 eq.). The product was obtained as a violet solid (2.5 g, 2.5 mmol, 93%). IR (ATR): ν = 1615, 1487, 1423, 1225, 1104, 1072, 1045, 1013, 952, 927, 840, 765, 731, 698, 693, 620, 564, 496, 489, 472. Elemental analysis calcd (%) for $C_{44}H_{36}CuCl_2N_4O_8$: C 59.83, H 4.11, N 6.34; found C 59.4, H 4.53, N 5.96.

Cu(2-MeOPy)$_2$(OTf)$_2$

The title compound was prepared according to GP1 from Cu(OTf)$_2$ (1.0 g, 2.8 mmol, 1 eq.) and 2-methoxypyridine (1.8 mL, 1.87 g, 17 mmol, 6 eq.). The product was obtained as a violet solid (1.9 g, 2.4 mmol, 84%). IR (ATR): ν = 3118, 1609, 1579, 1486, 1436, 1306, 1263, 1226, 1160, 1120, 1063, 1050, 1029, 1007, 783, 758, 652, 635, 572, 517. Elemental analysis calcd (%) for $C_{26}H_{28}CuF_6N_4O_{10}S_2$: C 39.12, H 3.54, N 7.02; found C 38.64, H 3.53, N 6.89.

Cu(2-MeOPy)$_2$(ClO$_4$)$_2$·2H$_2$O

The title compound was prepared according to GP1 from Cu(ClO$_4$)$_2$·6H$_2$O (1.0 g, 2.8 mmol, 1 eq.) and 2-methoxypyridine (1.8 mL, 1.87 g, 17 mmol, 6 eq.). The product was obtained as a violet solid (1.8 g, 2.3 mmol, 84%). IR (ATR): ν = 3117, 1609, 1577, 1485, 1431, 1303, 1289, 1267, 1166, 1078, 1028, 1005, 933, 878, 785, 652, 621, 572, 524, 498. Elemental analysis calcd (%) for $C_{24}H_{32}CuCl_2N_4O_{14}$: C 39.22, H 4.39, N 7.62; found C 39.52, H 4.31, N 7.34.

Cu(3-MeOPy)$_4$(OTf)$_2$

The title compound was prepared according to GP1 from Cu(OTf)$_2$ (1.0 g, 2.8 mmol, 1 eq.) and 3-methoxypyridine (1.8 mL, 1.95 g, 17 mmol, 6 eq.). The product was obtained as a violet solid (2.1 g, 2.6 mmol, 92%). IR (ATR): ν = 1580, 1494, 1432, 1282, 1240, 1227, 1197, 1187, 1160, 1112, 1060, 1028, 1012, 823, 807, 700, 634, 574, 562, 516. Elemental analysis calcd (%) for $C_{26}H_{28}CuF_6N_4O_{10}S_2$: C 39.12, H 3.54, N 7.02; found C 38.90, H 3.78, N 7.04.

Cu(3-MeOPy)$_4$(ClO$_4$)$_2$

The title compound was prepared according to GP1 from Cu(ClO$_4$)$_2$·6H$_2$O (1.0 g, 2.8 mmol, 1 eq.) and 3-methoxypyridine (1.8 mL, 1.95 g, 17 mmol, 6 eq.). The product was obtained as a violet solid (1.9 g, 2.4 mmol, 85%). IR (ATR): ν = 1575, 1496, 1487, 1430, 1290, 1248, 1197,

1103, 1067, 1058, 1046, 1032, 1010, 930, 909, 808, 700, 649, 620, 577. Elemental analysis calcd (%) for $C_{24}H_{28}CuCl_2N_4O_{12}$: C 41.24, H 4.04, N 8.02; found C 40.50, H 4.11, N 7.93.

Cu(4-MeOPy)$_4$(OTf)$_2$

The title compound was prepared according to GP1 from Cu(OTf)$_2$ (0.6 g, 1.7 mmol, 1 eq.) and 4-methoxypyridine (1.0 mL, 1.08 g, 10 mmol, 6 eq.). The product was obtained as a violet solid (1.3 g, 1.6 mmol, 94%). Crystals for the structural analysis were obtained by slow evaporation of a diluted solution of the complex in MeOH at ambient temperature. IR (ATR): ν = 1616, 1566, 1515, 1441, 1305, 1287, 1241, 1223, 1210, 1154, 1059, 1027, 1005, 836, 828, 812, 635, 572, 541, 516. Elemental analysis calcd (%) for $C_{26}H_{28}CuF_6N_4O_{10}S_2$: C 39.12, H 3.54, N 7.02; found C 38.90, H 3.67, N 7.01.

Cu(4-MeOPy)$_4$(ClO$_4$)$_2$

The title compound was prepared according to GP1 from Cu(ClO$_4$)$_2$·6 H$_2$O (0.6 g, 1.7 mmol, 1 eq.) and 4-methoxypyridine (1.0 mL, 1.08 g, 10 mmol, 6 eq.). The product was obtained as a violet solid (1.1 g, 1.6 mmol, 94%). IR (ATR): ν = 1617, 1567, 1517, 1504, 1441, 1306, 1210, 1111, 1100, 1070, 1057, 1052, 1030, 1010, 984, 838, 832, 813, 620, 539. Elemental analysis calcd (%) for $C_{24}H_{28}CuCl_2N_4O_{12}$: C 41.24, H 4.04, N 8.02; found C 40.90, H 4.02, N 8.03.

Cu[2,4-(MeO)$_2$Py]$_4$(OTf)$_2$

The title compound was prepared according to GP1 from Cu(OTf)$_2$ (1.0 g, 2.8 mmol, 1 eq.) and 2,4-dimethoxypyridine (2.2 mL, 2.34 g, 17 mmol, 6 eq.). The product was obtained as a blue solid (2.1 g, 2.6 mmol, 92%). IR (ATR): ν = 1615, 1575, 1484, 1439, 1341, 1258, 1219, 1186, 1162, 1116, 1044, 1026, 1006, 941, 831, 816, 636, 573, 517, 467. Elemental analysis calcd (%) for $C_{30}H_{36}CuF_6N_4O_{14}S_2$: C 39.24, H 3.95, N 6.10; found C 38.74, H 3.90, N 5.96.

Cu[2,4-(MeO)$_2$Py]$_4$(ClO$_4$)$_2$

The title compound was prepared according to GP1 from Cu(ClO$_4$)$_2$·6H$_2$O (1.0 g, 2.8 mmol, 1 eq.) and 2,4-dimethoxypyridine (2.2 mL, 2.34 g, 17 mmol, 6 eq.). The product was obtained as blue solid (1.9 g, 2.4 mmol, 85%). IR (ATR): ν = 1615, 1575, 1504, 1484, 1461, 1440, 1418, 1340, 1271, 1220, 1184, 1079, 1043, 1028, 1007, 941, 828, 664, 622, 464. Elemental analysis calcd (%) for $C_{28}H_{36}CuCl_2N_4O_{16}$: C 41.06, H 4.43, N 6.84; found C 41.03, H 4.78, N 6.78.

Cu(3,4-Me$_2$Py)$_4$(OTf)$_2$

The title compound was prepared according to GP1 from Cu(OTf)$_2$ (1.0 g, 2.8 mmol, 1 eq.) and 3,4-lutidine (1.8 mL, 1.72 g, 17 mmol, 6 eq.). The product was obtained as a violet solid (2.1 g, 2.6 mmol, 94%). Crystals for structural analysis were obtained by slow cooling of a solution of 10 mg of the title compound in 10 mL hot *i*PrOH to ambient temperature. IR (ATR): ν = 1615, 1501, 1451, 1290, 1241, 1223, 1207, 1155, 1086, 1032, 873, 843, 756, 722, 637, 617, 572, 542, 529, 516. Elemental analysis calcd (%) for $C_{30}H_{36}CuF_6N_4O_6S_2$: C 45.59, H 4.59, N 7.09; found C 45.10, H 4.81, N 7.08.

Cu(3,4-Me$_2$Py)$_4$(ClO$_4$)$_2$

The title compound was prepared according to GP1 from Cu(ClO$_4$)$_2$·6H$_2$O (1.0 g, 2.8 mmol, 1 eq.) and 3,4-lutidine (1.8 mL, 1.7 g, 17 mmol, 6 eq.). The product was obtained as a violet solid (1.8 g, 2.6 mmol, 91%). IR (ATR): ν = 2980, 1615, 1502, 1449, 1424, 1383, 1207, 1176, 1111, 1048, 1012, 979, 931, 874, 847, 835, 721, 621, 543, 529. Elemental analysis calcd (%) for $C_{28}H_{36}CuCl_2N_4O_8$: C 48.67, H 5.25, N 8.11; found C 48.3, H 5.34, N 8.09.

Cu(4,4'-BiPy)$_2$(OTf)$_2$·H$_2$O

The title compound was prepared according to GP1 from Cu(OTf)$_2$ (1.0 g, 2.8 mmol, 1 eq.) and 4,4'-bipyridine (2.7 g, 17 mmol, 6 eq.). The product was obtained as a light blue solid (1.1 g, 1.6 mmol, 57%). IR (ATR): ν = 1615, 1539, 1493, 1421, 1290, 1241, 1225, 1152, 1073, 1031, 1018, 818, 760, 730, 675, 635, 608, 574, 517, 488. Elemental analysis calcd (%) for $C_{22}H_{18}CuF_6N_4O_7S_2$: C 38.18, H 2.62, N 8.10; found C 37.81, H 2.92, N 7.87.

Cu(4,4'-BiPy)$_2$(ClO$_4$)$_2$·*i*PrOH

The title compound was prepared according to GP1 from Cu(ClO$_4$)$_2$·6H$_2$O (1.0 g, 2.7 mmol, 1 eq.) and 4,4'-bipyridine (2.5 g, 16 mmol, 6 eq.). The product was obtained as a light blue solid (2.1 g, >99%). IR (ATR): ν = 1612, 1600, 1419, 1408, 1076, 1064, 1019, 1004, 992, 833, 828, 813, 802, 729, 642, 623, 611, 571, 516, 476. Elemental analysis calcd (%) for $C_{23}H_{24}CuCl_2N_4O_9$: C 43.51, H 3.81, N 8.82; found C 44.58, H 3.25, N 10.27.

Cu(Pz)$_4$(OTf)$_2$·2H$_2$O[7]

The title compound was prepared according to GP1 from Cu(OTf)$_2$ (1.9 g, 5.4 mmol, 1 eq.) and pyrazine (2.6 g, 32 mmol, 6 eq.). The product was obtained as a light blue solid (3.7 g, 5.4 mmol, 95%). IR (ATR): ν = 1418, 1283, 1247, 1225, 1175, 1149, 1125, 1084, 1055, 1033,

1027, 805, 760, 749, 701, 633, 573, 515, 494, 455. Elemental analysis calcd (%) for $C_{18}H_{20}CuF_6N_8O_8S_2$: C 30.11, H 2.81, N 15.61; found C 29.52, H 2.45, N 14.69.

$Cu(4\text{-}F_3CPy)_4(OTf)_2{\cdot}2H_2O$

The title compound was prepared according to GP1 from $Cu(OTf)_2$ (2.9 g, 8.0 mmol, 1 eq.) and 4-(trifluoromethyl)pyridine (7.0 g, 48 mmol, 6 eq.). The product was obtained as a light blue solid (7.1 g, 7.4 mmol, 90%). IR (ATR): ν = 1425, 1326, 1298, 1241, 1215, 1168, 1137, 1108, 1089, 1061, 1029, 841, 759, 678, 665, 637, 610, 574, 517, 502. Elemental analysis calcd (%) for $C_{26}H_{20}CuF_{18}N_4O_8S_2$: C 31.67, H 2.04, N 5.68; found C 31.33, H 1.65, N 5.52.

$Cu(Quin)_3(OTf)_2{\cdot}MeOH$

$Cu(OTf)_2$ (500 mg, 1.4 mmol, 1 eq.) was suspended in degassed anhydrous MeOH (14 mL) and heated gently until the solution became clear. A solution of freshly distilled quinoline (1 mL, 1.1 g, 8.4 mmol, 6 eq.) in degassed anhydrous MeOH (17 mL) was added slowly at room temperature and the green solution placed in the freezer at –20 °C for 2 h. The solvent was decanted, and the precipitated solid was washed with hexane and dried over high vacuum. The title compound was obtained as dark green solid (425 mg, 0.54 mmol, 42%). IR (ATR): ν = 1510, 1289, 1251, 1235, 1224, 1166, 1146, 1133, 1057, 1032, 822, 808, 780, 756, 736, 635, 572, 556, 515, 489. Elemental analysis calcd (%) for $C_{30}H_{25}CuF_6N_3O_7S_2$: C 46.13, H 3.23, N 5.38; found C 46.90, H 3.40, N 5.55.

$Cu(Quin)_3(ClO_4)_2{\cdot}MeOH$

$Cu(ClO_4)_2{\cdot}6H_2O$ (500 mg, 1.3 mmol, 1 eq.) was suspended in degassed anhydrous MeOH (14 mL) and heated gently until the solution became clear. A solution of freshly distilled quinoline (0.9 mL, 1.0 g, 7.8 mmol, 6 eq.) in degassed anhydrous MeOH (17 mL) was added slowly at room temperature and the green solution placed in the freezer at –20 °C for 2 h. The solvent was decanted, and the precipitated solid was washed with hexane and dried over high vacuum. The title compound was obtained as dark green solid (355 mg, 0.52 mmol, 40%). IR (ATR): ν = 1509, 1312, 1095, 1083, 1067, 1047, 960, 824, 808, 788, 779, 746, 738, 640, 621, 559, 531, 500, 488, 467. Elemental analysis calcd (%) for $C_{28}H_{25}CuCl_2N_3O_9$: C 49.31, H 3.70, N 6.16; found C 49.04, H 3.82, N 6.10.

$Cu(Isoq)_4(OTf)_2$[16]

The title compound was prepared according to GP1 from $Cu(OTf)_2$ (1.0 g, 2.8 mmol, 1 eq.) and isoquinoline (2.2 g, 17 mmol, 6 eq.). The product was obtained as a light blue solid (2.5 g,

2.8 mmol, 99%). IR (ATR): ν = 1634, 1389, 1291, 1239, 1222, 1183, 1158, 1149, 1045, 1029, 1017, 962, 873, 830, 748, 632, 573, 516, 484, 471. Elemental analysis calcd (%) for $C_{38}H_{28}CuF_6N_4O_6S_2$: C 51.96, H 3.21, N 6.38; found C 51.80, H 3.61, N 6.25.

Cu(Isoq)$_4$(ClO$_4$)$_2$

The title compound was prepared according to GP1 from $Cu(ClO_4)_2{\cdot}6H_2O$ (1.0 g, 2.8 mmol, 1 eq.) and isoquinoline (2.2 g, 17 mmol, 6 eq.). The product was obtained as a light blue solid (2.2 g, 2.4 mmol, 98%). IR (ATR): ν = 1633, 1389, 1281, 1112, 1055, 1044, 1037, 1016, 998, 962, 931, 872, 835, 827, 745, 639, 620, 542, 484, 470. Elemental analysis calcd (%) for $C_{36}H_{28}CuCl_2N_4O_8$: C 55.50, H 3.62, N 7.19; found C 54.70, H 3.69, N 7.16.

Cu(Pyr)$_4$(OTf)$_2$[17]

The title compound was prepared according to GP1 from $Cu(OTf)_2$ (1.0 g, 2.8 mmol, 1 eq.) and *N*-methylpyrazole (1.4 mL, 1.38 g, 17 mmol, 6 eq.). The product was obtained as a blue solid (0.9 g, 1.3 mmol, 47%). IR (ATR): ν = 1526, 1434, 1413, 1274, 1241, 1223, 1175, 1152, 1114, 1083, 1056, 1028, 1000, 788, 766, 678, 633, 607, 573, 516. Elemental analysis calcd (%) for $C_{18}H_{24}CuF_6N_8O_6S_2$: C 31.33, H 3.51, N 16.24; found C 30.68, H 3.48, N 15.75.

Cu(Triaz)$_4$(OTf)$_2$

The title compound was prepared according to GP1 from $Cu(OTf)_2$ (1.0 g, 2.8 mmol, 1 eq.) and 1-methyl-1,2,4-triazole (1.3 mL, 1.4 g, 17 mmol, 6 eq.). The product was obtained as a light blue solid (1.7 g, 2.4 mmol, 85%). IR (ATR): ν = 1543, 1295, 1280, 1248, 1227, 1171, 1157, 1129, 1077, 1034, 994, 905, 883, 853, 691, 681, 672, 634, 573, 517. Elemental analysis calcd (%) for $C_{14}H_{20}CuF_6N_{12}O_6S_2$: C 24.23, H 2.90, N 24.22; found C 24.00, H 2.91, N 23.85.

Cu(Impdz)$_4$(OTf)$_2$[18]

The title compound was prepared according to GP1 from $Cu(OTf)_2$ (1.9 g, 5.4 mmol, 1 eq.) and imidazo(1,2-b)pyridazine (3.8 g, 32 mmol, 6 eq.). The product was obtained as a blue solid (4.4 g, 5.2 mmol, 96%). IR (ATR): ν = 1541, 1503, 1373, 1352, 1307, 1280, 1239, 1220, 1149, 1069, 1026, 878, 804, 754, 731, 632, 582, 572, 514, 457. Elemental analysis calcd (%) for $C_{26}H_{20}CuF_6N_{12}O_6S_2$: C 37.26, H 2.41, N 20.05; found C 36.80, H 2.36, N 19.80.

$Cu(Impdz)_4(ClO_4)_2{\cdot}H_2O$

The title compound was prepared according to GP1 from $Cu(ClO_4)_2{\cdot}6H_2O$ (2.0 g, 5.5 mmol, 1 eq.) and imidazo(1,2-b)pyridazine (3.9 g, 33 mmol, 6 eq.). The product was obtained as a light violet solid (4.4 g, >99%). IR (ATR): ν = 1541, 1504, 1372, 1348, 1301, 1270, 1151, 1088, 1068, 1023, 991, 806, 784, 758, 744, 637, 621, 583, 573, 459. Elemental analysis calcd (%) for $C_{24}H_{22}CuCl_2N_{12}O_9$: C 38.08, H 2.93, N 22.21; found C 38.10, H 3.06, N 22.20.

$Cu(Impdz)_4(ClO_3)_2{\cdot}2H_2O$

$Cu(ClO_3)_2$[11] was prepared as follows. $CuSO_4$ (1.2 g, 7.4 mmol, 1 eq.) was suspended in $MeOH/H_2O$ (2:1, 186 mL) and heated under reflux until a clear blue solution was obtained. A solution of $Ba(ClO_3)_2$ (2.4 g, 7.4 mmol, 1 eq.) in H_2O (19 mL) was added dropwise to the hot reaction mixture. After cooling to room temperature, the precipitated white solid was filtered off, washed with MeOH and the filtrate was concentrated under reduced pressure to afford $Cu(ClO_3)_2$ as a green solid. $Cu(ClO_3)_2$ was taken up in MeOH (124 mL) and imidazo(1,2-b)pyridazine (5.3 g, 44 mmol, 6 eq.) was added slowly at 80 °C. Thereafter, the reaction mixture was cooled to room temperature, the volume was reduced by half under reduced pressure and the reaction mixture was placed into the fridge for 16 h. The resulting precipitate was filtered off to afford the title compound as a blue solid (1.9 g, 2.7 mmol, 36%). IR (ATR): ν = 1620, 1537, 1500, 1372, 1350, 1303, 1270, 1149, 956, 927, 913, 793, 756, 731, 636, 604, 581, 573, 471, 458. Elemental analysis calcd (%) for $C_{24}H_{24}CuCl_2N_{12}O_8$: C 38.80, H 3.26, N 22.62; found C 39.12, H 3.27, N 22.33.

$Cu(Impdz)_4(OTs)_2{\cdot}2H_2O$

The title compound was prepared according to GP1 from $Cu(OTs)_2$ (1.1 g, 2.7 mmol, 1 eq.) and imidazo(1,2-b)pyridazine (1.9 g, 16 mmol, 6 eq.). The product was obtained as a dark turquoise solid (1.7 g, 1.9 mmol, 69%). IR (ATR): ν = 1539, 1371, 1349, 1303, 1230, 1186, 1174, 1151, 1119, 1035, 1010, 808, 798, 753, 717, 679, 634, 566, 549, 456. Elemental analysis calcd (%) for $C_{38}H_{38}CuN_{12}O_8S_2$: C 49.69, H 4.17, N 18.30; found C 50.03, H 4.11, N 17.91.

$[Cu(Impdz)_3(OMs)_2]_2$

The title compound was prepared according to GP1 from $Cu(OMs)_2$ (1.7 g, 6.8 mmol, 1 eq.) and imidazo(1,2-b)pyridazine (4.9 g, 41 mmol, 6 eq.). The product was obtained as a turquoise solid (4.3 g, 5.8 mmol, 85%). IR (ATR): ν = 1349, 1303, 1206, 1186, 1149, 1137, 1040, 808,

800, 793, 772, 757, 749, 727, 572, 550, 528, 516, 467, 455. Elemental analysis calcd (%) for $C_{20}H_{21}CuN_9O_6S_2$: C 39.31, H 3.46, N 20.63; found C 39.35, H 3.48, N 21.32.

2.2 Crystallography

Crystallographic tables for $Cu(Py)_4(OTf)_2$

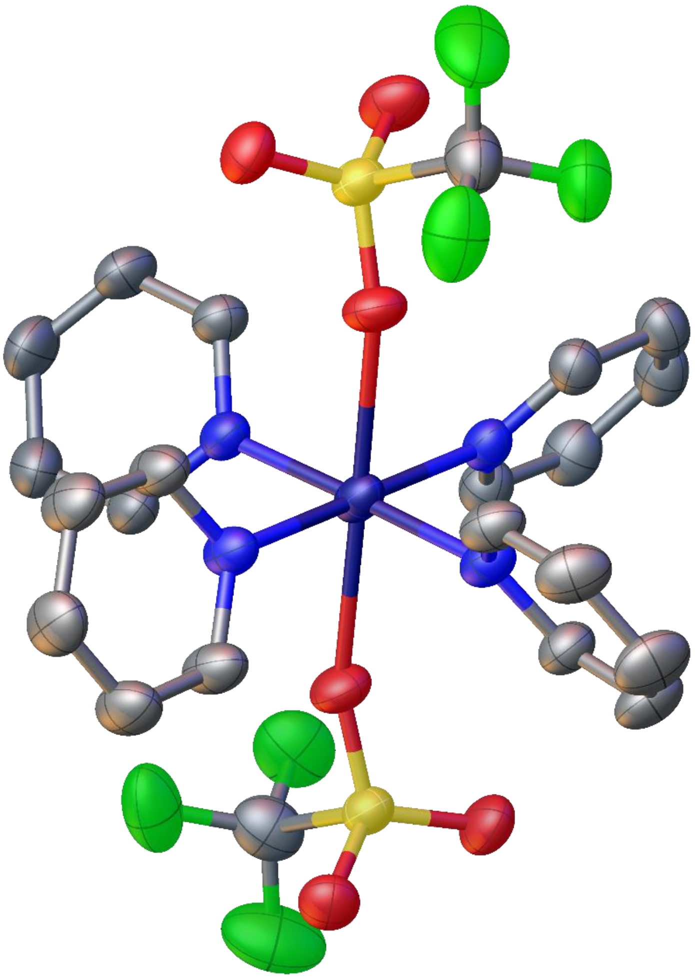

Figure S1: Crystal structure of $Cu(Py)_4(OTf)_2$.

Table S1: Crystal data and structure refinement for $Cu(Py)_4(OTf)_2$.

Identification code	$Cu(Py)_4(OTf)_2$
CCDC number:	2202867
Empirical formula	$C_{11}H_{10}Cu_{0.5}F_3N_2O_3S$
Formula weight	339.048
Temperature/K	293(2)
Crystal system	orthorhombic
Space group	Pbcn
a/Å	10.461(5)
b/Å	16.152(5)

c/Å	16.556(5)
α/°	90.000(5)
β/°	90.000(5)
γ/°	90.000(5)
Volume/$Å^3$	2797.4(18)
Z	8
ρ_{calc}g/cm^3	1.610
μ/mm^{-1}	1.014
F(000)	1375.3
Radiation	Mo Kα (λ = 0.71073)
2Θ range for data collection/°	4.64 to 53.6
Index ranges	-13 ≤ h ≤ 13, -20 ≤ k ≤ 20, -20 ≤ l ≤ 20
Reflections collected	39891
Independent reflections	2978 [R_{int} = 0.0796, R_{sigma} = 0.0298]
Data/restraints/parameters	2978/0/189
Goodness-of-fit on F^2	1.042
Final R indexes [I>=2σ (I)]	R_1 = 0.0352, wR_2 = 0.0878
Final R indexes [all data]	R_1 = 0.0552, wR_2 = 0.0979
Largest diff. peak/hole / e $Å^{-3}$	0.49/-0.44

Table S2: Fractional atomic coordinates (×10^4) and equivalent isotropic displacement parameters ($Å^2$×10^3) for $Cu(Py)_4(OTf)_2$. U_{eq} is defined as 1/3 of the trace of the orthogonalized U_{IJ} tensor.

**Atom**	***x***	***y***	***z***	**U(eq)**
C1	2478(2)	789.7(15)	8237.0(16)	41.5(5)
C2	1491(2)	851.1(19)	8778.2(19)	54.0(7)
C3	1670(3)	1297(2)	9476(2)	60.2(8)
C4	2835(3)	1662.0(18)	9613.2(19)	55.1(7)
C5	3779(2)	1583.5(15)	9041.6(17)	45.0(6)
C6	6032(3)	2819.0(16)	7730.9(19)	49.7(7)
C7	6064(3)	3667.2(17)	7749(2)	55.1(7)
C8	5000	4099(2)	7500	54.7(10)
C9	4764(2)	-564.0(14)	6818.4(17)	41.5(6)
C10	4750(3)	-1415.4(16)	6802.6(19)	48.8(7)
C11	5000	-1847(2)	7500	49.8(9)
C12	7242(3)	1013.5(18)	10020.3(19)	57.6(7)
N1	3618.1(18)	1153.4(11)	8354.1(13)	37.9(4)
N2	5000	2390.1(16)	7500	40.1(7)
N3	5000	-134.5(16)	7500	36.2(6)
O1	6530.8(16)	1141.7(11)	8564.3(12)	46.6(4)
O2	8498.3(17)	1823.2(11)	8947.9(12)	47.2(4)
O3	8414.7(18)	324.5(11)	8827.5(14)	53.9(5)
F1	6533(2)	338.4(11)	10147.6(12)	71.4(5)
F2	6516(2)	1668.0(12)	10219.8(12)	77.1(6)
F3	8223(2)	993.3(15)	10521.1(13)	91.0(7)

Atom	*x*	*y*	*z*	U(eq)
S1	7748.1(5)	1083.6(3)	8974.8(4)	36.84(16)
Cu1	5000	1128.3(2)	7500	36.47(14)

Table S3: Anisotropic displacement parameters ($Å^2×10^3$) for $Cu(Py)_4(OTf)_2$. The anisotropic displacement factor exponent takes the form:
$-2\pi^2[h^2a^{*2}U_{11}+2hka^*b^*U_{12}+…]$.

Atom	U_{11}	U_{22}	U_{33}	U_{12}	U_{13}	U_{23}
C1	31.0(11)	42.1(12)	51.4(15)	-3.0(9)	0.1(11)	-3.0(11)
C2	30.1(13)	66.6(17)	65.2(19)	-2.5(12)	2.9(12)	-8.5(14)
C3	40.2(14)	77(2)	63.8(19)	6.9(13)	12.2(14)	-16.5(16)
C4	44.7(15)	56.9(16)	63.6(19)	9.7(12)	-1.1(14)	-18.3(14)
C5	33.1(12)	40.5(12)	61.5(17)	4.0(10)	-3.3(12)	-10.8(12)
C6	36.1(13)	37.0(12)	76(2)	-2.2(10)	-9.8(13)	4.0(12)
C7	43.2(15)	39.3(13)	83(2)	-7.2(11)	-6.8(15)	-3.0(13)
C8	54(2)	29.5(16)	80(3)	-0	0(2)	0
C9	38.7(13)	37.8(12)	47.9(15)	-0.7(9)	4.5(11)	-2.4(10)
C10	50.1(16)	40.4(13)	55.8(17)	-3.6(11)	7.0(13)	-8.8(11)
C11	50(2)	31.9(16)	67(3)	-0	13(2)	0
C12	65.6(19)	53.3(17)	53.9(17)	-12.9(14)	-0.5(15)	4.8(13)
N1	27.8(9)	33.0(10)	53.0(12)	1.2(7)	-0.1(9)	-4.6(8)
N2	32.1(14)	28.8(13)	59.5(19)	-0	-4.4(14)	0
N3	29.2(13)	29.8(12)	49.4(16)	-0	4.9(13)	0
O1	30.3(9)	51.6(10)	58.0(11)	4.6(7)	-8.1(8)	-0.2(8)
O2	40.8(10)	42.9(10)	57.9(11)	-9.4(7)	3.7(9)	-1.7(8)
O3	37.9(10)	42.7(10)	81.1(15)	9.0(7)	0.5(10)	-8.2(9)
F1	79.5(13)	62.8(11)	71.8(13)	-19.3(9)	11.0(10)	15.4(9)
F2	94.6(15)	64.8(11)	71.9(12)	-10.2(10)	35.4(11)	-16.4(9)
F3	104.5(17)	110.2(17)	58.2(12)	-32.5(14)	-28.0(12)	17.5(11)
S1	28.3(3)	35.1(3)	47.1(3)	0.5(2)	-1.1(2)	-1.6(2)
Cu1	25.8(2)	27.8(2)	55.8(3)	-0	2.27(17)	0

Table S4: Bond lengths for $Cu(Py)_4(OTf)_2$.

Atom	Atom	Length/Å	Atom	Atom	Length/Å
C1	C2	1.371(4)	C12	F1	1.336(3)
C1	N1	1.343(3)	C12	F2	1.343(4)
C2	C3	1.374(4)	C12	F3	1.320(4)
C3	C4	1.373(4)	C12	S1	1.813(3)
C4	C5	1.373(4)	N1	Cu1[1]	2.023(2)
C5	N1	1.344(3)	N2	Cu1	2.038(3)
C6	C7	1.371(4)	N3	Cu1	2.040(3)
C6	N2	1.338(3)	O1	S1	1.4465(18)
C7	C8	1.377(3)	O1	Cu1	2.3811(19)

Atom	Atom	Length/Å	Atom	Atom	Length/Å
C9	C10	1.376(3)	O2	S1	1.4300(18)
C9	N3[1]	1.347(3)	O3	S1	1.4314(18)
C10	C11[1]	1.374(4)			

[1]1-X,+Y,3/2-Z

Table S5: Bond angles for $Cu(Py)_4(OTf)_2$.

Atom	Atom	Atom	Angle/°	Atom	Atom	Atom	Angle/°
N1	C1	C2	122.9(2)	Cu1	N3	C9[1]	120.99(14)
C3	C2	C1	119.0(3)	Cu1	N3	C9	120.99(14)
C4	C3	C2	119.0(3)	Cu1	O1	S1	159.91(12)
C5	C4	C3	119.0(3)	O1	S1	C12	101.29(14)
N1	C5	C4	122.8(2)	O2	S1	C12	104.00(13)
N2	C6	C7	122.9(3)	O2	S1	O1	114.48(11)
C8	C7	C6	118.7(3)	O3	S1	C12	104.55(14)
C7[1]	C8	C7	119.1(3)	O3	S1	O1	113.85(11)
N3[1]	C9	C10	122.2(3)	O3	S1	O2	116.29(11)
C11[1]	C10	C9	119.3(3)	N1	Cu1	N1[1]	177.70(11)
C10	C11	C10[1]	119.0(3)	N2	Cu1	N1[1]	88.85(5)
F2	C12	F1	106.8(3)	N2	Cu1	N1	88.85(5)
F3	C12	F1	108.2(2)	N3	Cu1	N1[1]	91.15(5)
F3	C12	F2	107.7(3)	N3	Cu1	N1	91.15(5)
S1	C12	F1	111.3(2)	N3	Cu1	N2	180.0
S1	C12	F2	110.5(2)	O1	Cu1	N1	87.89(7)
S1	C12	F3	112.0(2)	O1[1]	Cu1	N1[1]	87.89(7)
C5	N1	C1	117.3(2)	O1	Cu1	N1[1]	92.09(7)
Cu1[1]	N1	C1	121.66(17)	O1[1]	Cu1	N1	92.09(7)
Cu1[1]	N1	C5	120.88(16)	O1[1]	Cu1	N2	89.48(4)
C6[1]	N2	C6	117.6(3)	O1	Cu1	N2	89.48(4)
Cu1	N2	C6	121.18(15)	O1[1]	Cu1	N3	90.52(4)
Cu1	N2	C6[1]	121.18(15)	O1	Cu1	N3	90.52(4)
C9	N3	C9[1]	118.0(3)	O1[1]	Cu1	O1	178.96(9)

[1]1-X,+Y,3/2-Z

Crystallographic tables for Cu(4-MeOPy)$_4$(ClO$_4$)$_2$

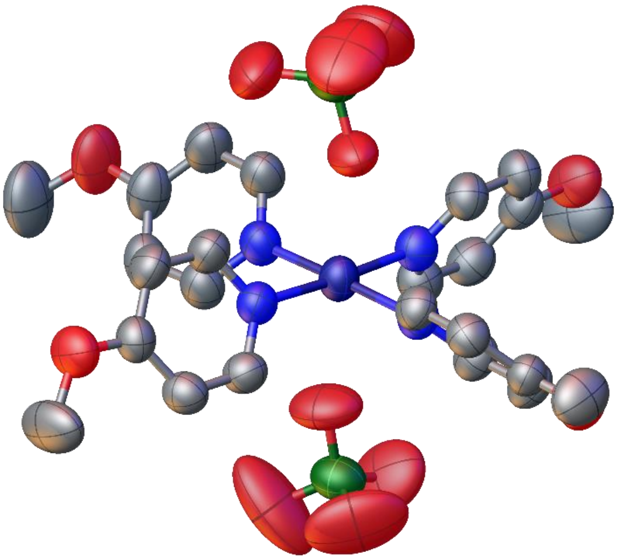

Figure S2: Crystal structure of Cu(4-MeOPy)$_4$(ClO$_4$)$_2$.

Table S6: Crystal data and structure refinement for Cu(4-MeOPy)$_4$(ClO$_4$)$_2$.

Identification code	Cu(4-OMePy)$_4$(ClO$_4$)$_2$
CCDC number:	2202868
Empirical formula	$C_{24}H_{28}N_4O_{12}Cl_2Cu$
Formula weight	698.960
Temperature/K	293(2)
Crystal system	orthorhombic
Space group	Pbca
a/Å	10.849(5)
b/Å	15.715(5)
c/Å	36.036(5)
α/°	90.000(5)
β/°	90.000(5)
γ/°	90.000(5)
Volume/Å^3	6144(4)
Z	8
ρ_{calc}g/cm^3	1.511
μ/mm^{-1}	0.950
F(000)	2878.8
Radiation	Mo Kα (λ = 0.71073)
2Θ range for data collection/°	4.38 to 49.3
Index ranges	$-12 \leq h \leq 11$, $-18 \leq k \leq 18$, $-42 \leq l \leq 42$
Reflections collected	97722
Independent reflections	5169 [R_{int} = 0.0883, R_{sigma} = 0.0280]
Data/restraints/parameters	5169/0/389
Goodness-of-fit on F^2	1.032
Final R indexes [I>=2σ (I)]	R_1 = 0.0509, wR_2 = 0.1418

Final R indexes [all data] R_1 = 0.0791, wR_2 = 0.1615
Largest diff. peak/hole / e Å^{-3} 0.67/-0.49

Table S7: Fractional atomic coordinates (×10^4) and equivalent isotropic displacement parameters (Å^2×10^3) for Cu(4-MeOPy)$_4$(ClO$_4$)$_2$. U_{eq} is defined as 1/3 of the trace of the orthogonalized U_{IJ} tensor.

**Atom**	***x***	***y***	***z***	**U(eq)**
C1	9245(4)	744(3)	873.5(12)	78.8(12)
C2	8759(4)	373(3)	558.9(14)	84.2(13)
C3	8879(5)	784(4)	230.4(13)	89.5(14)
C4	9488(5)	1550(4)	222.9(13)	95.1(15)
C5	9947(4)	1882(3)	545.1(13)	79.8(12)
C6	7621(7)	-216(5)	-94(2)	144(3)
C7	12766(4)	1524(3)	988.3(12)	76.9(11)
C8	13889(4)	1590(3)	817.9(12)	79.5(12)
C9	14351(4)	2386(3)	752.6(12)	74.9(11)
C10	13689(4)	3092(3)	869.2(13)	79.8(12)
C11	12580(4)	2964(3)	1039.5(12)	72.8(11)
C12	16159(6)	1854(5)	450(2)	140(3)
C13	12231(4)	2331(3)	1939.0(12)	73.7(11)
C14	12705(4)	2646(3)	2258.8(13)	75.1(11)
C15	12022(4)	3195(3)	2474.0(11)	65.8(10)
C16	10857(4)	3416(3)	2349.6(12)	67.7(10)
C17	10450(4)	3081(3)	2022.9(12)	67.6(10)
C18	11853(6)	4037(4)	3019.0(15)	106.9(18)
C19	7734(4)	2243(3)	1388.0(12)	76.2(12)
C20	6563(4)	2101(3)	1505.2(13)	79.1(12)
C21	6353(4)	1573(3)	1802.8(12)	69.0(10)
C22	7356(4)	1238(3)	1983.6(12)	71.1(11)
C23	8523(4)	1417(3)	1852.0(11)	69.3(10)
C24	4933(6)	784(5)	2162(2)	138(3)
N1	9829(3)	1492(2)	871.6(9)	70.7(9)
N2	12096(3)	2193(2)	1098.6(9)	67.9(8)
N3	11103(3)	2542(2)	1812.7(9)	65.5(8)
N4	8726(3)	1906(2)	1556.7(9)	66.6(8)
O1	148(7)	4416(6)	668(2)	227(4)
O2	-189(3)	3567(2)	1157.5(13)	111.6(13)
O3	-1704(5)	3741(3)	718.3(18)	168(2)
O4	-1173(7)	4872(4)	1100(2)	210(3)
O5	983(5)	581(3)	1583.1(13)	134.6(16)
O6	904(8)	-836(4)	1662(3)	244(4)
O7	1392(8)	-2(5)	2133.9(13)	219(4)
O8	2697(4)	-214(4)	1638.0(19)	178(2)
O9	8402(4)	519(3)	-96.0(10)	130.2(15)

Atom	*x*	*y*	*z*	U(eq)
O10	15444(3)	2560(3)	587.2(10)	100.9(11)
O11	12550(3)	3475(2)	2787.4(8)	82.8(8)
O12	5170(3)	1442(2)	1900.0(10)	93.2(10)
Cl1	-716.6(14)	4157.8(9)	913.0(5)	107.9(5)
Cl2	1511.9(12)	-101.4(7)	1762.2(4)	86.4(4)
Cu01	10444.0(5)	2042.4(3)	1341.48(13)	68.0(2)

Table S8: Anisotropic displacement parameters ($Å^2 \times 10^3$) for $Cu(4\text{-}MeOPy)_4(ClO_4)_2$. The anisotropic displacement factor exponent takes the form: $-2\pi^2[h^2a^{*2}U_{11}+2hka^*b^*U_{12}+\ldots]$.

Atom	U_{11}	U_{22}	U_{33}	U_{12}	U_{13}	U_{23}
C1	74(3)	90(3)	73(3)	-14(2)	5(2)	5(2)
C2	73(3)	93(3)	87(3)	-13(2)	7(2)	-18(3)
C3	73(3)	128(4)	68(3)	-12(3)	2(2)	-19(3)
C4	94(3)	129(4)	63(3)	-23(3)	-6(2)	9(3)
C5	75(3)	95(3)	70(3)	-14(2)	2(2)	6(2)
C6	137(6)	175(7)	119(5)	-48(5)	-10(4)	-53(5)
C7	78(3)	76(3)	76(3)	-2(2)	3(2)	-5(2)
C8	73(3)	90(3)	76(3)	13(2)	0(2)	-4(2)
C9	57(2)	101(3)	66(2)	7(2)	-0.5(19)	7(2)
C10	64(3)	88(3)	88(3)	-9(2)	2(2)	7(2)
C11	62(2)	77(3)	80(3)	0(2)	0(2)	-4(2)
C12	94(4)	181(7)	144(6)	45(5)	50(4)	25(5)
C13	59(2)	93(3)	70(2)	7(2)	1(2)	-2(2)
C14	58(2)	93(3)	74(3)	11(2)	-5(2)	2(2)
C15	61(2)	73(2)	63(2)	-5(2)	-4.4(18)	4.8(19)
C16	64(2)	65(2)	74(2)	2.9(19)	4(2)	-2(2)
C17	53(2)	75(3)	74(2)	5(2)	-3.3(19)	2(2)
C18	130(5)	91(3)	100(4)	15(3)	-18(3)	-28(3)
C19	67(3)	90(3)	72(3)	-7(2)	-8(2)	11(2)
C20	58(3)	97(3)	82(3)	-2(2)	-12(2)	11(2)
C21	57(2)	75(3)	75(2)	-7(2)	1.2(19)	-2(2)
C22	67(3)	72(3)	74(2)	2(2)	8(2)	9(2)
C23	66(3)	76(3)	66(2)	4(2)	-1.7(19)	8(2)
C24	80(4)	167(6)	167(6)	-2(4)	34(4)	75(5)
N1	66(2)	81(2)	65(2)	-12.8(18)	1.4(16)	4.4(17)
N2	64(2)	74(2)	66(2)	-2.0(17)	2.5(16)	-3.6(16)
N3	51.5(18)	77(2)	67.4(19)	0.6(16)	2.2(15)	4.3(17)
N4	62.3(19)	72(2)	65.8(19)	-4.1(16)	-5.0(16)	2.4(16)
O1	216(7)	266(8)	198(6)	-56(6)	28(5)	117(6)
O2	88(2)	92(2)	155(3)	0.8(19)	-26(2)	42(2)
O3	139(4)	139(4)	225(6)	-17(3)	-88(4)	50(4)
O4	220(7)	130(4)	278(9)	78(4)	-38(6)	-24(5)

Atom	U_{11}	U_{22}	U_{33}	U_{12}	U_{13}	U_{23}
O5	143(4)	115(3)	146(4)	49(3)	8(3)	47(3)
O6	281(9)	134(5)	316(10)	-91(5)	130(8)	-82(6)
O7	311(9)	272(8)	75(3)	100(7)	32(4)	20(4)
O8	96(3)	241(6)	197(6)	63(4)	25(3)	14(5)
O9	128(3)	183(4)	80(2)	-50(3)	-7(2)	-30(2)
O10	63.5(19)	139(3)	100(2)	10(2)	17.6(18)	22(2)
O11	82(2)	91(2)	75.1(18)	1.2(16)	-13.7(16)	-7.4(16)
O12	62.6(19)	111(2)	106(2)	-2.7(17)	13.3(17)	16.3(19)
Cl1	89.7(9)	88.9(9)	144.9(13)	1.7(7)	-8.2(9)	37.5(9)
Cl2	90.2(8)	75.4(7)	93.7(8)	13.5(6)	14.5(6)	11.3(6)
Cu01	58.5(3)	82.3(4)	63.2(3)	-7.7(2)	0.1(2)	0.1(2)

Table S9: Bond lengths for $Cu(4\text{-}MeOPy)_4(ClO_4)_2$.

Atom	Atom	Length/Å	Atom	Atom	Length/Å
C1	C2	1.379(6)	C17	N3	1.338(5)
C1	N1	1.335(6)	C18	O11	1.432(6)
C2	C3	1.355(7)	C19	C20	1.357(6)
C3	C4	1.374(8)	C19	N4	1.345(5)
C3	O9	1.351(6)	C20	C21	1.375(6)
C4	C5	1.367(7)	C21	C22	1.373(6)
C5	N1	1.333(6)	C21	O12	1.347(5)
C6	O9	1.433(7)	C22	C23	1.381(6)
C7	C8	1.369(6)	C23	N4	1.331(5)
C7	N2	1.339(5)	C24	O12	1.423(7)
C8	C9	1.368(7)	N1	Cu01	2.015(3)
C9	C10	1.387(7)	N2	Cu01	2.009(3)
C9	O10	1.355(5)	N3	Cu01	2.003(3)
C10	C11	1.366(6)	N4	Cu01	2.030(3)
C11	N2	1.337(5)	O1	Cl1	1.351(6)
C12	O10	1.442(7)	O2	Cl1	1.403(4)
C13	C14	1.356(6)	O3	Cl1	1.438(5)
C13	N3	1.348(5)	O4	Cl1	1.401(6)
C14	C15	1.377(6)	O5	Cl2	1.377(4)
C15	C16	1.385(6)	O6	Cl2	1.378(6)
C15	O11	1.341(5)	O7	Cl2	1.355(5)
C16	C17	1.363(6)	O8	Cl2	1.373(5)

Table S10: Bond angles for $Cu(4\text{-}MeOPy)_4(ClO_4)_2$.

Atom	Atom	Atom	Angle/°	Atom	Atom	Atom	Angle/°
N1	C1	C2	123.3(4)	C11	N2	C7	116.9(4)
C3	C2	C1	118.7(5)	Cu01	N2	C7	121.4(3)
C4	C3	C2	118.8(4)	Cu01	N2	C11	121.8(3)
O9	C3	C2	125.3(5)	C17	N3	C13	116.4(4)
O9	C3	C4	115.9(5)	Cu01	N3	C13	120.9(3)
C5	C4	C3	119.5(5)	Cu01	N3	C17	122.6(3)
N1	C5	C4	122.6(5)	C23	N4	C19	117.1(4)
N2	C7	C8	123.8(4)	Cu01	N4	C19	121.4(3)
C9	C8	C7	118.2(4)	Cu01	N4	C23	121.3(3)
C10	C9	C8	119.4(4)	C6	O9	C3	118.0(5)
O10	C9	C8	125.5(4)	C12	O10	C9	117.8(5)
O10	C9	C10	115.1(4)	C18	O11	C15	117.8(4)
C11	C10	C9	118.3(4)	C24	O12	C21	117.1(4)
N2	C11	C10	123.4(4)	O2	Cl1	O1	109.1(4)
N3	C13	C14	122.8(4)	O3	Cl1	O1	109.6(5)
C15	C14	C13	120.2(4)	O3	Cl1	O2	108.0(3)
C16	C15	C14	117.8(4)	O4	Cl1	O1	108.6(5)
O11	C15	C14	116.7(4)	O4	Cl1	O2	111.9(4)
O11	C15	C16	125.5(4)	O4	Cl1	O3	109.7(4)
C17	C16	C15	118.5(4)	O6	Cl2	O5	109.3(5)
N3	C17	C16	124.2(4)	O7	Cl2	O5	109.5(4)
N4	C19	C20	122.9(4)	O7	Cl2	O6	108.1(5)
C21	C20	C19	119.8(4)	O8	Cl2	O5	109.7(4)
C22	C21	C20	118.0(4)	O8	Cl2	O6	104.8(4)
O12	C21	C20	117.0(4)	O8	Cl2	O7	115.3(5)
O12	C21	C22	124.9(4)	N2	Cu01	N1	88.85(14)
C23	C22	C21	119.0(4)	N3	Cu01	N1	177.38(15)
N4	C23	C22	123.0(4)	N3	Cu01	N2	90.28(13)
C5	N1	C1	117.1(4)	N4	Cu01	N1	88.39(13)
Cu01	N1	C1	122.1(3)	N4	Cu01	N2	176.52(13)
Cu01	N1	C5	120.8(3)	N4	Cu01	N3	92.57(13)

Crystallographic tables for Cu(3,4-Me$_2$Py)$_4$(OTf)$_2$

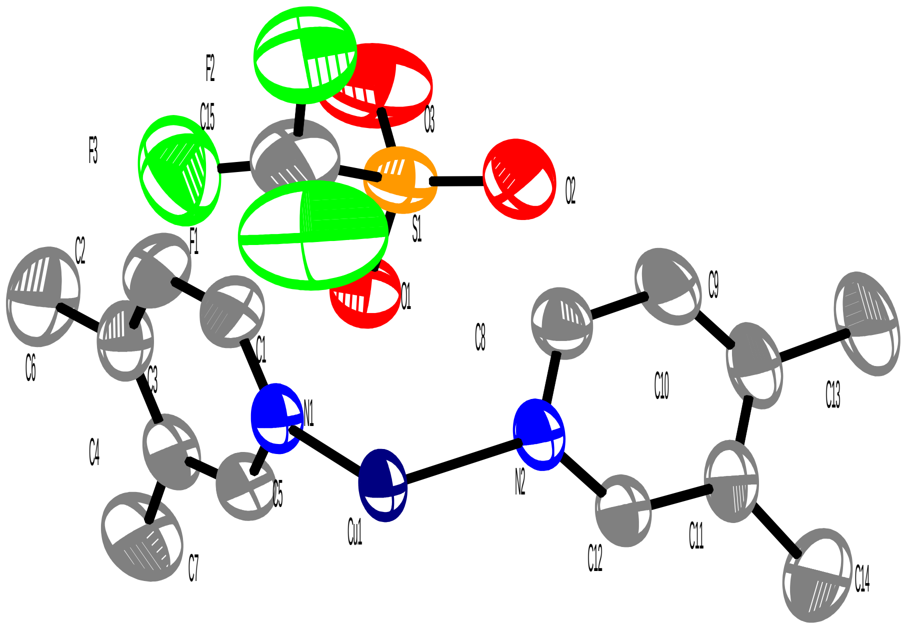

Figure S3: Asymmetric unit of Cu(3,4-Me$_2$Py)$_4$(OTf)$_2$ and naming scheme.

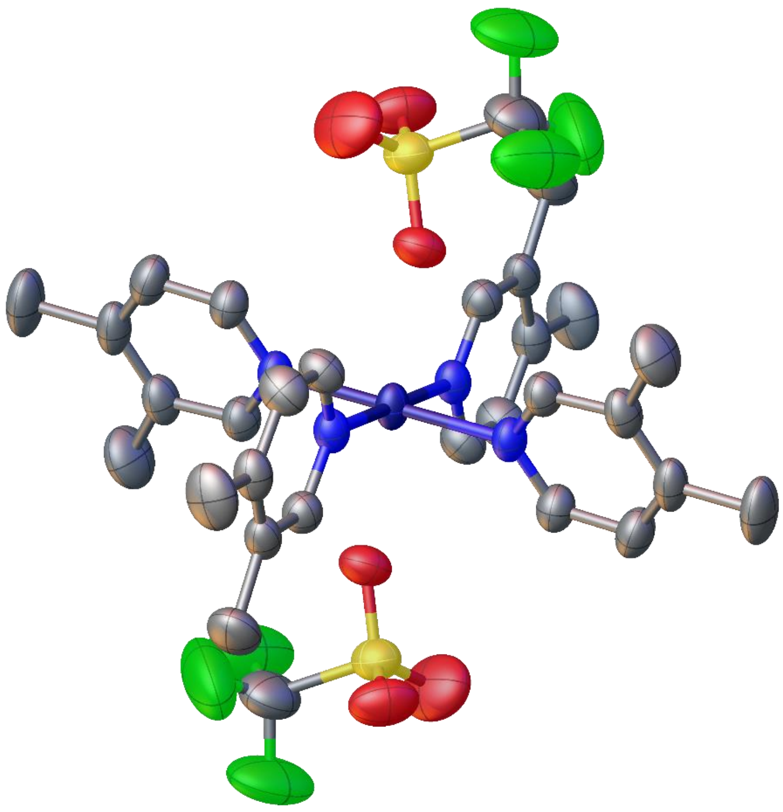

Figure S4: Crystal structure of Cu(3,4-Me$_2$Py)$_4$(OTf)$_2$.

Table S11: Crystal data and structure refinement for $Cu(3,4\text{-}Me_2Py)_4(OTf)_2$.

Identification code	$Cu(3,4\text{-}Me_2Py)_4(OTf)_2$
CCDC number	2202858
Empirical formula	$C_{30}H_{36}N_4O_6F_6S_2Cu$
Formula weight	790.31
Temperature/K	100.0(2)
Crystal system	monoclinic
Space group	$P2_1/n$
a/Å	10.1196(6)
b/Å	10.4414(5)
c/Å	17.5274(10)
α/°	90
β/°	106.280(2)
γ/°	90
Volume/Å^3	1777.73(17)
Z	2
ρ_{calc}g/cm^3	1.4766
μ/mm^{-1}	0.809
F(000)	815.9
Crystal size/mm^3	0.827 × 0.452 × 0.433
Radiation	Mo Kα (λ = 0.71073)
2Θ range for data collection/°	5.74 to 67.7
Index ranges	$-15 \leq h \leq 15$, $-16 \leq k \leq 14$, $-27 \leq l \leq 27$
Reflections collected	64608
Independent reflections	7126 [R_{int} = 0.0335, R_{sigma} = 0.0152]
Data/restraints/parameters	7126/0/227
Goodness-of-fit on F^2	1.023
Final R indexes [I>=2σ (I)]	R_1 = 0.0439, wR_2 = 0.1382
Final R indexes [all data]	R_1 = 0.0519, wR_2 = 0.1453
Largest diff. peak/hole / e Å^{-3}	0.60/-0.51

Table S12: Fractional atomic coordinates (×10[4]) and equivalent isotropic displacement parameters (Å^2×10^3) for Cu(3,4-Me_2Py)$_4$(OTf)$_2$. U_{eq} is defined as 1/3 of the trace of the orthogonalized U_{IJ} tensor.

**Atom**	***x***	***y***	***z***	**U(eq)**
Cu1	5000	5000	5000	40.12(8)
S1	4702.4(5)	4082.0(5)	2769.2(3)	56.44(11)
N1	3005.9(11)	5614.3(10)	4678.9(7)	40.0(2)
N2	5628.7(12)	6825.7(10)	4916.4(8)	41.3(2)
O1	4832.8(16)	4717.0(14)	3517.7(8)	58.6(3)
O2	5590(2)	2998.2(16)	2836.4(10)	84.4(5)
C12	5271.9(16)	7463.1(13)	4228.2(9)	45.6(3)
C1	2224.4(16)	5710.8(16)	3927.9(9)	49.6(3)
C5	2438.3(15)	5995.8(13)	5246.6(9)	44.7(3)
C2	898.5(18)	6180.0(19)	3727.9(11)	56.7(4)
C11	5686.2(16)	8709.4(13)	4132.6(11)	50.1(3)
C3	315.6(15)	6568.4(14)	4317.7(11)	49.9(3)
C9	6848.2(18)	8684.6(15)	5512.2(12)	57.2(4)
C10	6495.6(17)	9338.5(13)	4795.7(12)	53.4(4)
C8	6416.5(16)	7439.0(14)	5551.4(10)	49.4(3)
C4	1114.0(15)	6470.3(12)	5099.4(10)	46.2(3)
O3	3322(2)	3918(3)	2281.3(15)	116.0(8)
F2	5272(4)	4873(2)	1491.9(11)	136.7(11)
F3	4804(4)	6352.9(19)	2198.0(12)	152.0(11)
C14	5242(3)	9312(2)	3321.0(15)	78.6(6)
F1	6715(3)	5431(4)	2582.1(18)	168.4(13)
C13	7026(3)	10677.2(17)	4759.7(19)	81.4(7)
C7	607(3)	6884(2)	5788.2(15)	74.5(6)
C6	-1125(2)	7082(2)	4105.1(18)	78.1(6)
C15	5438(4)	5242(3)	2235.9(15)	88.9(8)

Table S13: Anisotropic displacement parameters ($Å^2 \times 10^3$) for $Cu(3,4\text{-}Me_2Py)_4(OTf)_2$. The anisotropic displacement factor exponent takes the form: $-2\pi^2[h^2a^{*2}U_{11}+2hka^*b^*U_{12}+\ldots]$.

Atom	U_{11}	U_{22}	U_{33}	U_{12}	U_{13}	U_{23}
Cu1	35.19(11)	27.06(11)	59.92(16)	0.15(6)	16.29(9)	4.48(7)
S1	55.9(2)	63.2(2)	50.0(2)	-0.11(17)	14.42(16)	-9.19(16)
N1	37.7(5)	32.0(4)	52.9(6)	0.9(3)	17.0(4)	3.7(4)
N2	41.1(5)	29.1(4)	56.7(6)	-1.7(3)	18.9(4)	1.9(4)
O1	71.6(8)	60.6(6)	50.3(6)	6.7(6)	28.0(6)	-2.7(5)
O2	112.8(14)	68.2(9)	70.0(9)	25.3(9)	22.2(9)	-10.5(7)
C12	49.1(7)	35.1(5)	55.5(7)	-1.7(5)	19.4(6)	2.7(5)
C1	46.8(7)	54.5(7)	50.7(7)	7.5(6)	19.1(6)	5.4(6)
C5	44.8(6)	41.0(6)	50.2(7)	-0.8(5)	16.2(5)	-1.6(5)
C2	48.2(7)	64.3(9)	56.6(8)	9.6(7)	13.1(6)	9.9(7)
C11	51.5(7)	35.3(6)	71.0(9)	1.9(5)	29.6(7)	9.9(6)
C3	39.4(6)	40.3(6)	73.3(9)	3.9(5)	21.2(6)	8.3(6)
C9	53.2(8)	42.5(7)	76.6(11)	-11.6(6)	19.6(7)	-8.8(7)
C10	46.7(7)	31.5(5)	90.1(12)	-4.2(5)	32.6(7)	0.4(6)
C8	47.5(7)	41.1(6)	59.7(8)	-5.8(5)	15.0(6)	0.7(5)
C4	47.6(6)	34.6(5)	64.2(8)	-2.3(5)	28.5(6)	-2.8(5)
O3	63.4(10)	158(2)	112.0(17)	-12.9(12)	-0.1(10)	-32.5(15)
F2	234(3)	133.1(18)	62.0(9)	21.0(15)	72.4(15)	8.1(8)
F3	298(4)	78.5(11)	98.4(14)	21.0(17)	86.4(18)	14.9(10)
C14	91.9(16)	63.6(11)	85.4(15)	-0.1(10)	33.2(12)	28.8(10)
F1	142(2)	241(3)	137(2)	-94(2)	62.7(19)	1(2)
C13	75.5(13)	37.5(7)	140(2)	-15.3(8)	44.6(14)	1.9(10)
C7	79.3(14)	73.4(12)	87.2(14)	3.2(10)	50.4(12)	-13.4(10)
C6	46.1(8)	78.7(14)	113.5(19)	17.3(9)	28.9(10)	19.6(13)
C15	125(3)	94.1(16)	57.8(12)	-4.6(17)	41.5(15)	2.5(11)

[1]1-X,1-Y,1-Z

Table S14: Bond lengths for Cu(3,4-Me_2Py)$_4$(OTf)$_2$.

Atom	Atom	Length/Å	Atom	Atom	Length/Å
Cu1	N1[1]	2.0405(11)	C5	C4	1.384(2)
Cu1	N1	2.0405(11)	C2	C3	1.387(3)
Cu1	N2[1]	2.0279(10)	C11	C10	1.386(3)
Cu1	N2	2.0279(10)	C11	C14	1.504(3)
S1	O1	1.4426(13)	C3	C4	1.386(2)
S1	O2	1.4289(16)	C3	C6	1.499(2)
S1	O3	1.4288(18)	C9	C10	1.385(3)
S1	C15	1.813(3)	C9	C8	1.380(2)
N1	C1	1.3359(19)	C10	C13	1.505(2)
N1	C5	1.3412(18)	C4	C7	1.502(2)
N2	C12	1.3356(19)	F2	C15	1.324(3)
N2	C8	1.337(2)	F3	C15	1.318(4)
C12	C11	1.3916(19)	F1	C15	1.279(4)
C1	C2	1.378(2)			

[1]1-X,1-Y,1-Z

Table S15: Bond angles for Cu(3,4-Me_2Py)$_4$(OTf)$_2$.

Atom	Atom	Atom	Angle/°	Atom	Atom	Atom	Angle/°
N1[1]	Cu1	N1	180.0	C3	C2	C1	120.11(16)
N2[1]	Cu1	N1[1]	89.46(4)	C10	C11	C12	118.00(15)
N2[1]	Cu1	N1	90.54(4)	C14	C11	C12	119.00(17)
N2	Cu1	N1[1]	90.54(4)	C14	C11	C10	123.00(15)
N2	Cu1	N1	89.46(4)	C4	C3	C2	117.62(14)
N2[1]	Cu1	N2	180.0	C6	C3	C2	120.44(18)
O2	S1	O1	113.20(9)	C6	C3	C4	121.95(18)
O3	S1	O1	115.11(13)	C8	C9	C10	120.32(16)
O3	S1	O2	116.15(14)	C9	C10	C11	117.92(13)
C15	S1	O1	102.46(11)	C13	C10	C11	122.63(18)
C15	S1	O2	103.42(15)	C13	C10	C9	119.44(18)
C15	S1	O3	104.20(18)	C9	C8	N2	122.24(16)
C1	N1	Cu1	124.19(10)	C3	C4	C5	118.47(13)
C5	N1	Cu1	119.00(10)	C7	C4	C5	118.98(17)
C5	N1	C1	116.76(12)	C7	C4	C3	122.54(16)
C12	N2	Cu1	121.52(10)	F2	C15	S1	110.9(2)
C8	N2	Cu1	121.07(10)	F3	C15	S1	110.5(2)
C8	N2	C12	117.41(12)	F3	C15	F2	106.3(3)
C11	C12	N2	124.09(14)	F1	C15	S1	111.4(2)
C2	C1	N1	122.86(15)	F1	C15	F2	110.2(3)
C4	C5	N1	124.18(14)	F1	C15	F3	107.5(3)

[1]1-X,1-Y,1-Z

In all three crystal structures, Cu(II) is coordinated by four *N*-donor systems in a planar fashion (Figs. S1–4). The coordination sphere surrounding the Cu(II) ion can best be described as an elongated octahedron, which is most distorted for the pyridine structure and symmetrical in the case of Cu(3,4-lutidine)$_4$(OTf)$_2$ due to the characteristics of the crystal refinement.

The crystal structures of the pyridine and 3,4-lutidine complexes exhibit similar Cu−N bond distances, yet the axial triflate coligand is 3.7% and 9.6% further away from the metal center, leading to a more distorted coordination sphere. The 4-methoxy substitution at the pyridine ring leads to a small (about 2%) shortening of the Cu−N bond distance in comparison to the complex with the unsubstituted pyridine. The tilt of the pyridine rings out of the Cu(II) coordination plane remains similar when comparing the pyridine and 4-methoxy pyridine ligands, while the lutidine ligands are almost perpendicular to the plane.

2.3 Preparation of aryl trimethylstannanes – General Procedure 2 (GP2)

Sn_2Me_6 (1.5 eq.) was added slowly at ambient temperature to a solution of the respective aryl iodide or bromide (1 eq.) and $Pd(PPh_3)_4$ (0.2 eq) in a suspension of LiCl (5 eq.) in anhydrous toluene. The reaction mixture was stirred at 110 °C until TLC indicated complete conversion of the aryl halogenide (typically 4 h). After cooling to ambient temperature, the mixture was diluted with EtOAc and filtered through a plug of Celite®. All volatiles were removed under reduced pressure and the residue was purified by flash column chromatography to afford the desired aryl trimethyl stannane.

4-Ph-Ph-SnMe$_3$ (5)[19]

The title compound was obtained as a colorless solid (382 mg, 1.20 mmol, 67%) according to GP2 from 4-iodobiphenyl (500 mg, 1.8 mmol, 1 eq.) and Sn_2Me_6 (0.60 mL, 0.95 g, 2.7 mmol, 1.5 eq.) using $Pd(PPh_3)_4$ (0.42 g, 0.36 mmol, 0.2 eq) and LiCl (0.38 g, 9 mmol, 5 eq) in anhydrous toluene (25 mL). The crude product was purified by flash chromatography (petrol ether, R_f = 0.49). ^{1}H NMR (400 MHz, $CDCl_3$): δ = 7.67 – 7.54 (m, 6H), 7.48 – 7.44 (m, 2H), 7.39 – 7.34 (m, 1H), 0.34 (s, 9H). ^{13}C NMR (100 MHz, $CDCl_3$): δ = 141.4, 141.3, 141.2, 136.4, 128.9, 127.4, 127.3, 126.3, –9.4.

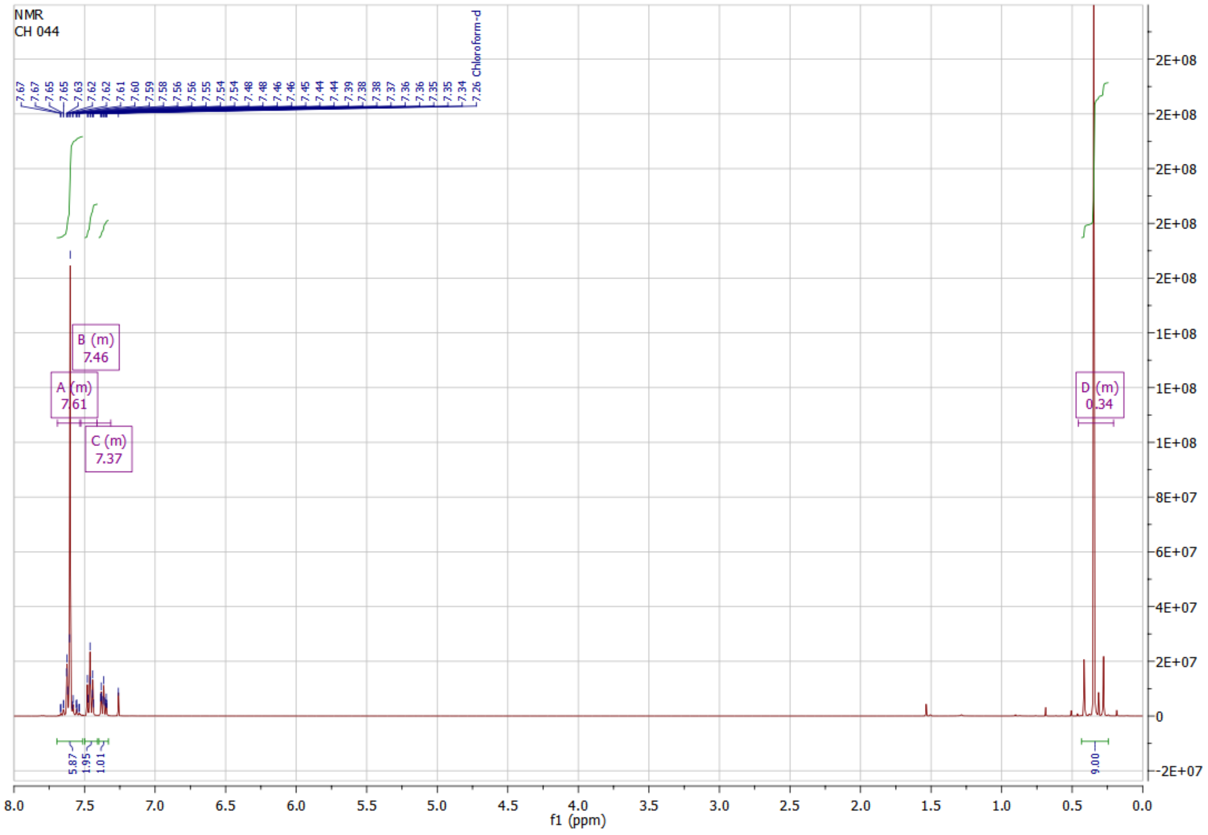

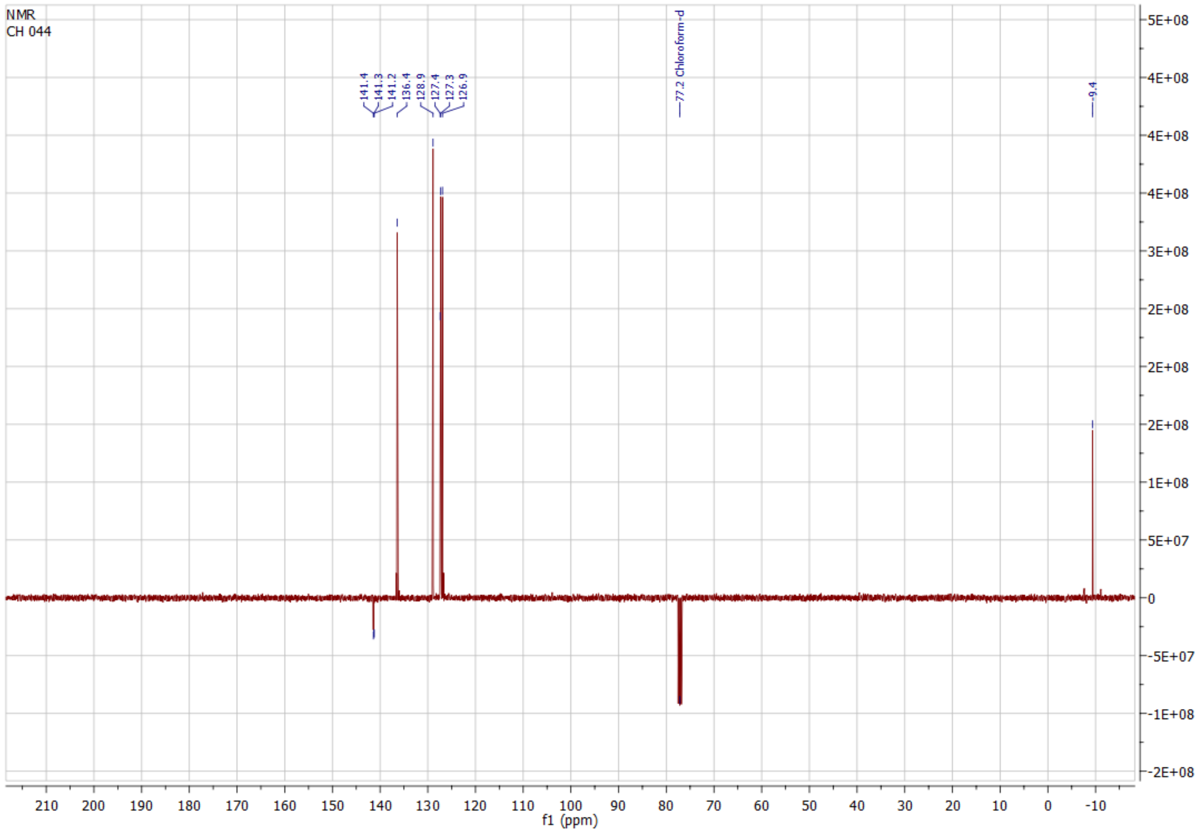

4-Ac-Ph-SnMe$_3$ (6)[20]

The title compound was obtained as a colorless oil (1.0 g, 3.6 mmol, 89%) according to GP2 from 4-iodoacetophenone (1.0 g, 4.1 mmol, 1 eq.) and Sn_2Me_6 (1.3 mL, 2.1 g, 6.1 mmol, 1.5 eq.) using $Pd(PPh_3)_4$ (0.94 g, 0.82 mmol, 0.2 eq) and LiCl (0.87 g, 21 mmol, 5 eq) in anhydrous toluene (30 mL). The crude product was purified by flash chromatography (petrol ether/EtOAc = 9:1, R_f = 0.48). ^{1}H NMR (400 MHz, $CDCl_3$): δ = 7.89 (d, J = 8.19 Hz, 2H), 7.61(d, J = 8.10 Hz, 2H), 2.59 (s, 3H), 0.33 (s, 9H). ^{13}C NMR (100 MHz, $CDCl_3$): δ = 198.6, 150.3, 136.9, 136.1, 127.4, 26.7, –9.4.

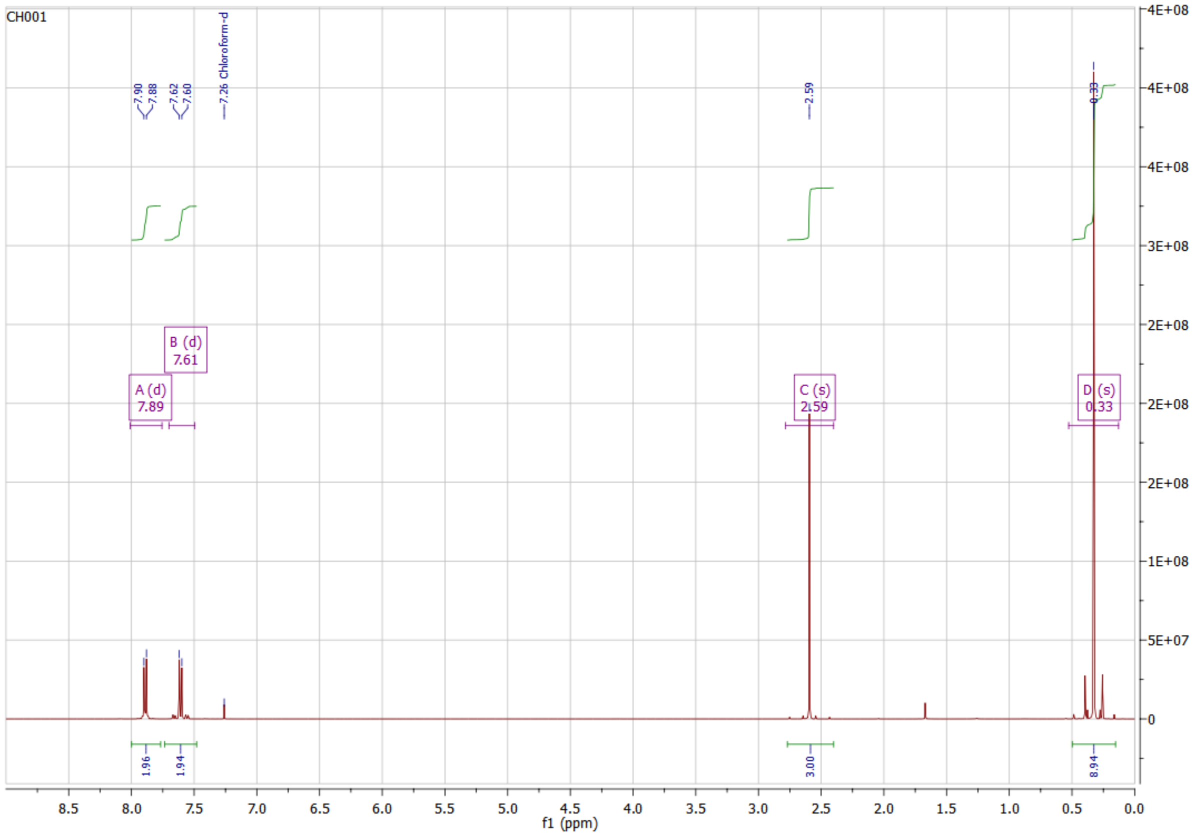
CH001
7.90
7.88
7.62
7.60
7.26 Chloroform-d
2.59
0.33
A (d)
7.89
B (d)
7.61
C (s)
2.59
D (s)
0.33
1.96
1.94
3.00
8.94
f1 (ppm)

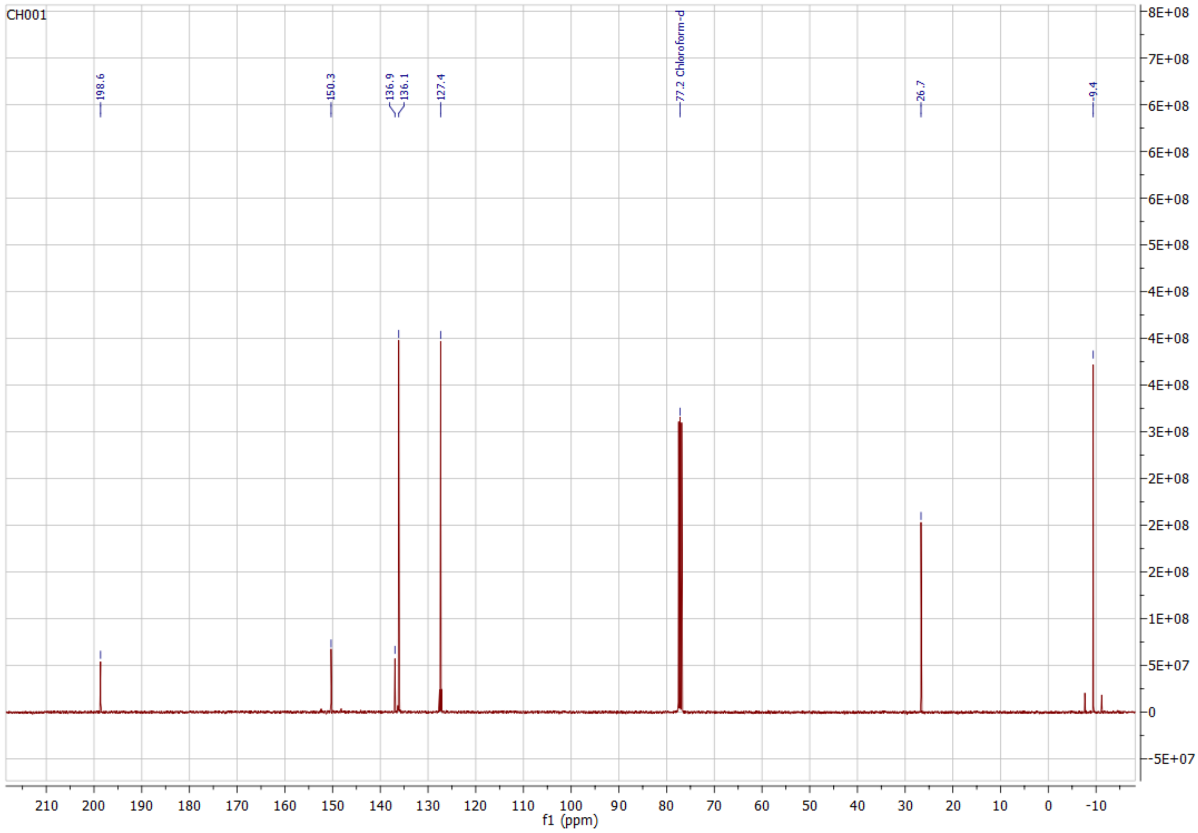
CH001
198.6
150.3
136.9
136.1
127.4
77.2 Chloroform-d
26.7
-9.4
f1 (ppm)

2.4 Preparation of aryl neopentyl glycol boronates – General Procedure 3 (GP3)

Anhydrous $MgSO_4$ (3–4 eq.; dried at 300 ºC for 16 h and cooled to ambient temperature under Ar before use) was added to a solution of the respective aryl boronic acid (1 eq.) and 2,2-dimethyl-1,3-propandiol (1 eq.) in anhydrous Et_2O (0.3 M) and the reaction mixture was vigorously stirred for 16 h. Thereafter, the mixture was filtered through a pad of silica gel (0.1% Ca) and concentrated under reduced pressure. The residue was dried at 1 mbar and 50 ºC and purified by recrystallization from hexane to afford the desired aryl boronate.

4-Ph-Ph-Bneo[21]

The title compound was obtained as a colorless solid (1.0 g, 3.8 mmol, 74%) according to GP3 from 4-Ph-Ph-$B(OH)_2$ (1.0 g, 5.1 mmol, 1 eq.) and 2,2-dimethyl-1,3-propandiol (0.53 g, 5.1 mmol, 1 eq.) using $MgSO_4$ (2.2 g, 18 mmol, 3.6 eq). R_f = 0.50 (hexane/EtOAc = 6:1). ^{1}H NMR (400 MHz, $CDCl_3$): δ = 7.93 – 7.87 (m, 2H), 7.68 – 7.59 (m, 4H), 7.49 – 7.42 (m, 2H), 7.40 – 7.33 (m, 1H), 3.81 (s, 4H), 1.05 (s, 6H). ^{13}C NMR(101 MHz, $CDCl_3$): δ = 143.4, 141.4, 134.5, 128.9, 127.5, 127.4, 126.5, 72.5, 32.1, 22.1. *C*-Bneo was not observed.

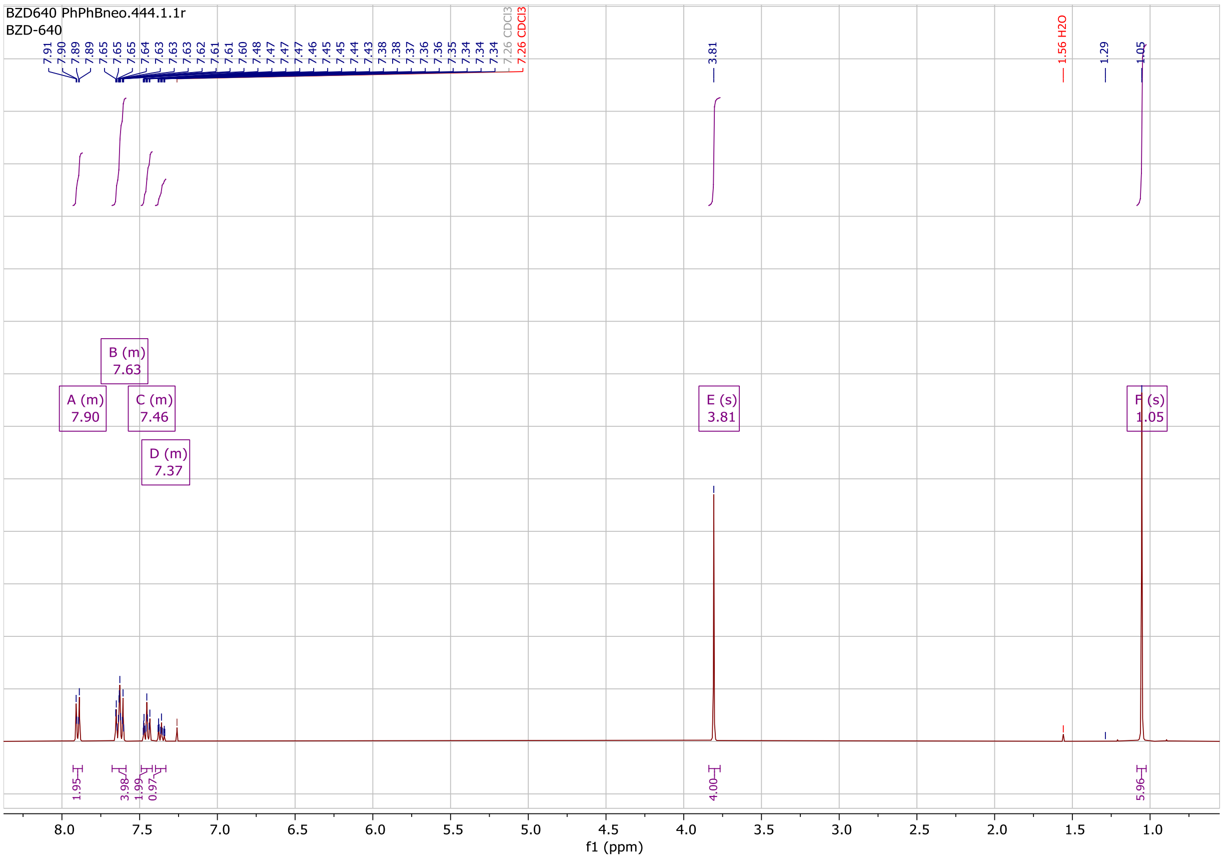
BZD640 PhPhBneo.444.1.1r
BZD-640
A (m) 7.90
B (m) 7.63
C (m) 7.46
D (m) 7.37
E (s) 3.81
F (s) 1.05
f1 (ppm)

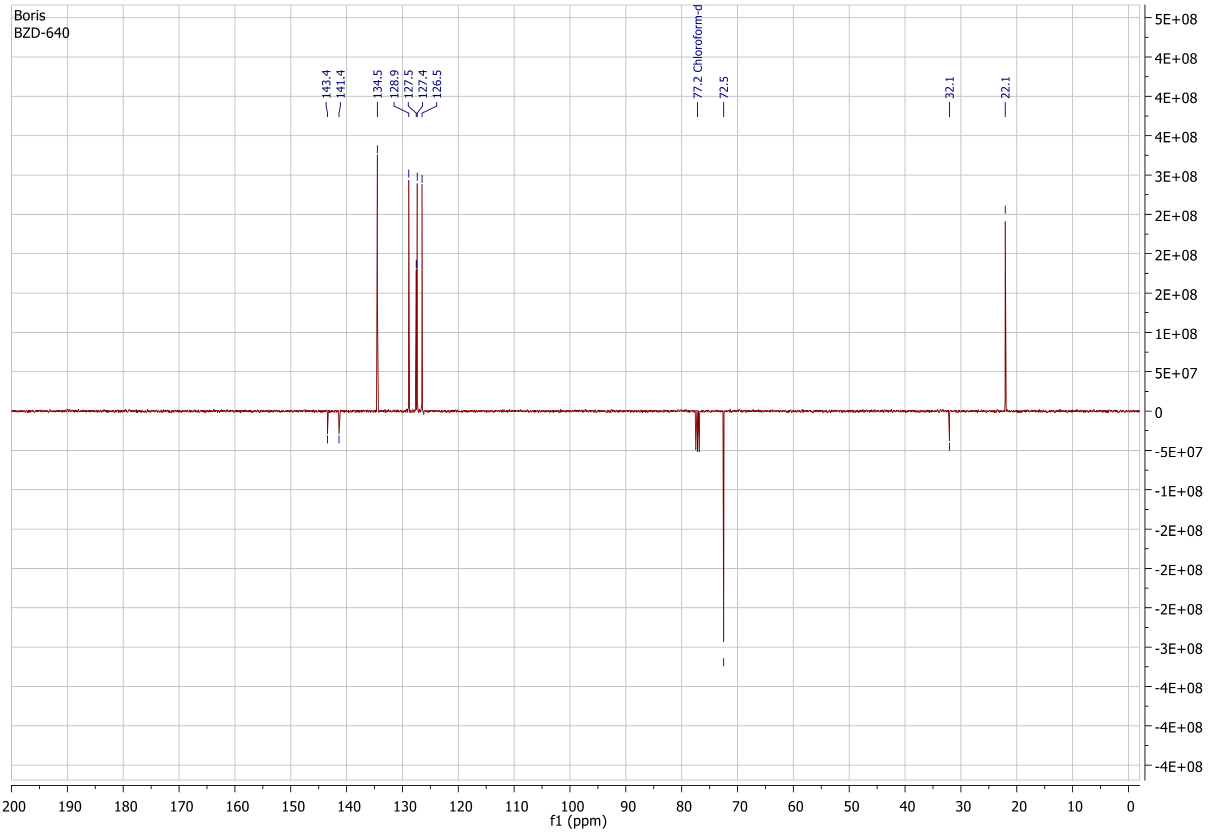
Boris
BZD-640
143.4
141.4
134.5
128.9
127.5
127.4
126.5
77.2 Chloroform-d
72.5
32.1
22.1
f1 (ppm)

4-Ac-Ph-Bneo[22]

The title compound was obtained as a colorless solid (1.0 g, 4.4 mmol, 72%) according to GP3 from 4-Ac-Ph-B(OH)$_2$ (1.0 g, 6.1 mmol, 1 eq.) and 2,2-dimethyl-1,3-propandiol (0.64 g, 6.1 mmol) using $MgSO_4$ (2.3 g, 19 mmol, 3.1 eq.). R_f = 0.28 (hexane/EtOAc = 6:1). ^{1}H NMR (400 MHz, $CDCl_3$): δ = 7.96 – 7.84 (m, 4H), 3.78 (s, 4H), 2.61 (s, 3H), 1.03 (s, 6H). ^{13}C NMR (101 MHz, $CDCl_3$): δ = 198.7, 138.7, 134.1, 127.3, 72.5, 32.0, 26.9, 22.0. *C*-Bneo was not observed. LR-MS: m/z $[M+H]^+$ calculated for $[C_{13}H_{17}BO_3+H]^+$ = 233.13, found: 233.23.

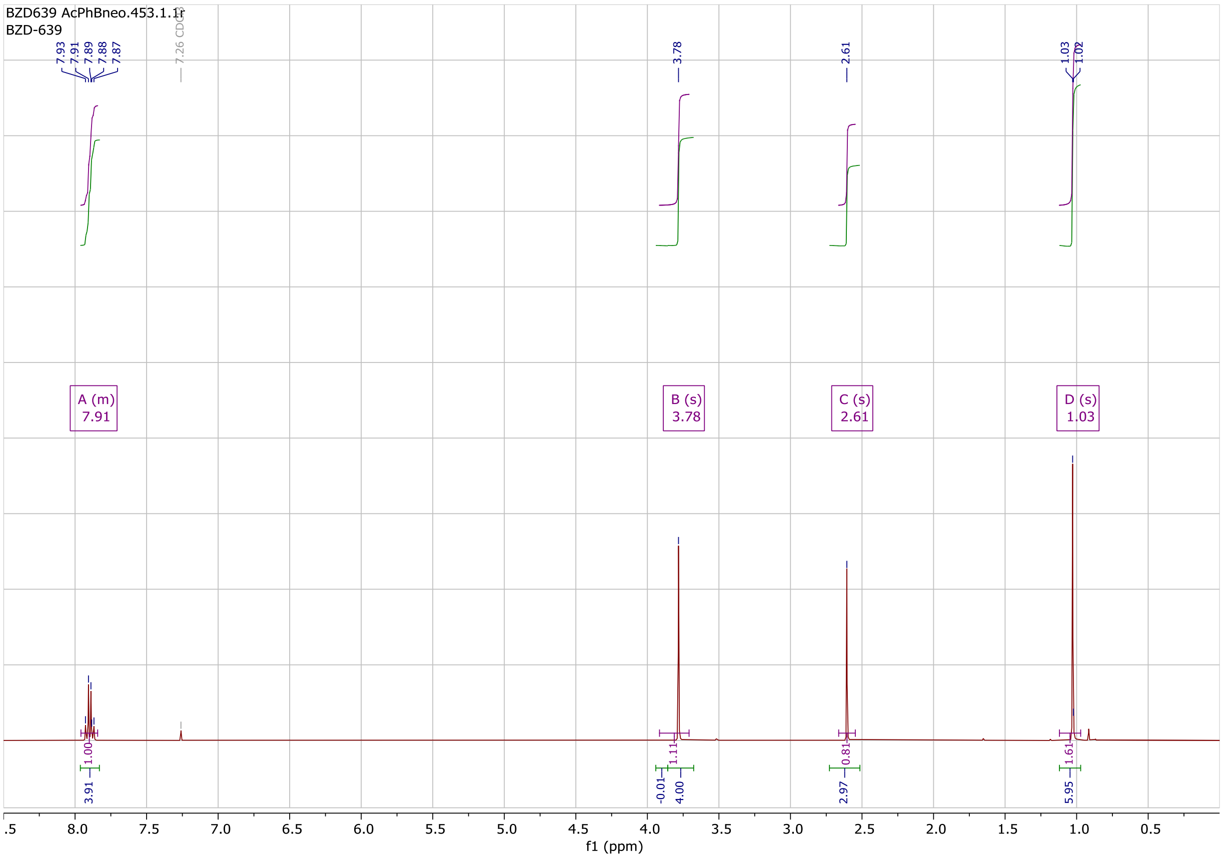

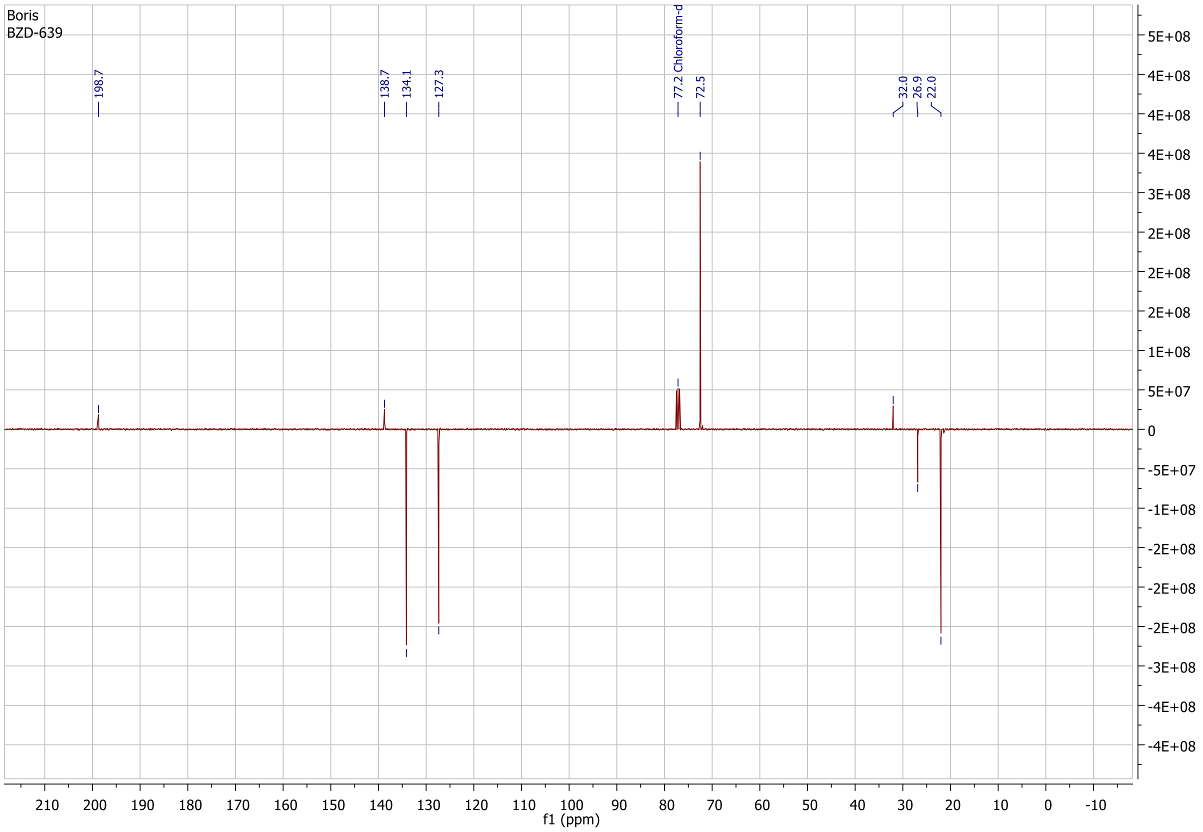

2.5 Preparation of (Boc)$_2$-6-BpinDOPA(MOM)$_2$-OMe (S4)

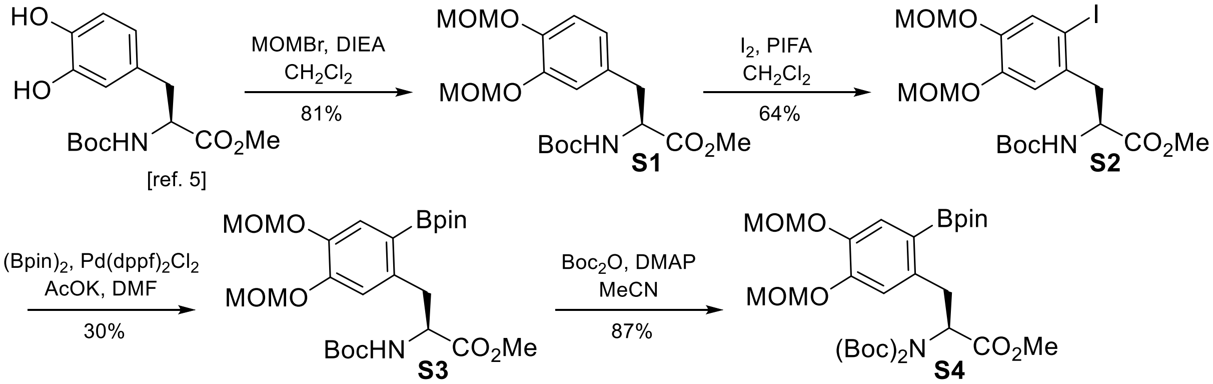

Scheme S1: Preparation of (Boc)$_2$-6-BpinDOPA(MOM)$_2$-OMe (**S4**). (Bpin)$_2$ – bis-(pinacolato)-diborane; DMAP – (4-dimethylamino)pyridine; MOMBr – methoxymethyl bromide; Pd(dppf)$_2$Cl$_2$ – [1,1'-bis-(diphenylphosphino)-ferrocene]-dichloropalladium; PIFA – [bis(trifluoroacetoxy)iodo]benzene.

Boc-DOPA(MOM)$_2$-OMe (S1)

MOMBr (13.8 mL, 21.9 g, 175 mmol, 3.5 eq.) was added dropwise to a solution of Boc-DOPA-OMe (15.7 g, 50.0 mmol, 1 eq.) and DIEA (30.5 mL, 22.6 g, 175 mmol, 3.5 eq.) in CH_2Cl_2 (120 mL) and the reaction mixture was stirred for 2 h. Thereafter, additional DIEA (15.3 mL, 11.3 g, 87.5 mmol, 1.75 eq.) followed by MOMBr (6.40 mL, 10.9 g, 87.4 mmol, 1.75 eq.) was added and the mixture was stirred for 16 h. Afterwards, all volatiles were removed under reduced pressure and the residue was taken up in Et_2O/H_2O (200 mL of each). The organic layer was separated, successively washed with 1 M $NaHSO_4$ (3×50 mL), H_2O (50 mL), 10% $NaHCO_3$ (3×50 mL), H_2O (3×50 mL), and brine (2×30 mL), dried, filtered and concentrated under reduced pressure. The resulting crude product was purified by column chromatography (EtOAc:hexane 1:1.5; R_f=0.41) to afford the title compound as a colorless oil (16.3 g, 40.8 mmol, 82%). ^{1}H NMR (400 MHz, $CDCl_3$) δ = 7.10 – 7.02 (m, 1H), 6.91 (d, J = 1.9 Hz, 1H), 6.70 (dd, *J* = 8.2, 1.7 Hz, 1H), 5.22 – 5.16 (m, 4H), 5.01 – 4.91 (m, *J* = 7.6 Hz, 1H), 4.62 – 4.48 (m, 1H), 3.73 (s, 3H), 3.50 (s, 6H), 1.42 (s, 9H). ^{13}C NMR (101 MHz, $CDCl_3$): δ = 172.4, 155.2, 147.4, 146.4, 130.3, 123.4, 117.8, 116.8, 95.6 (×2), 80.0, 56.3, 56.2, 54.5, 52.3, 37.8, 28.4. LR-MS: m/z $[M+H]^+$ calculated for $[C_{19}H_{29}NO_8+H]^+$ = 400.2, found: 399.98.

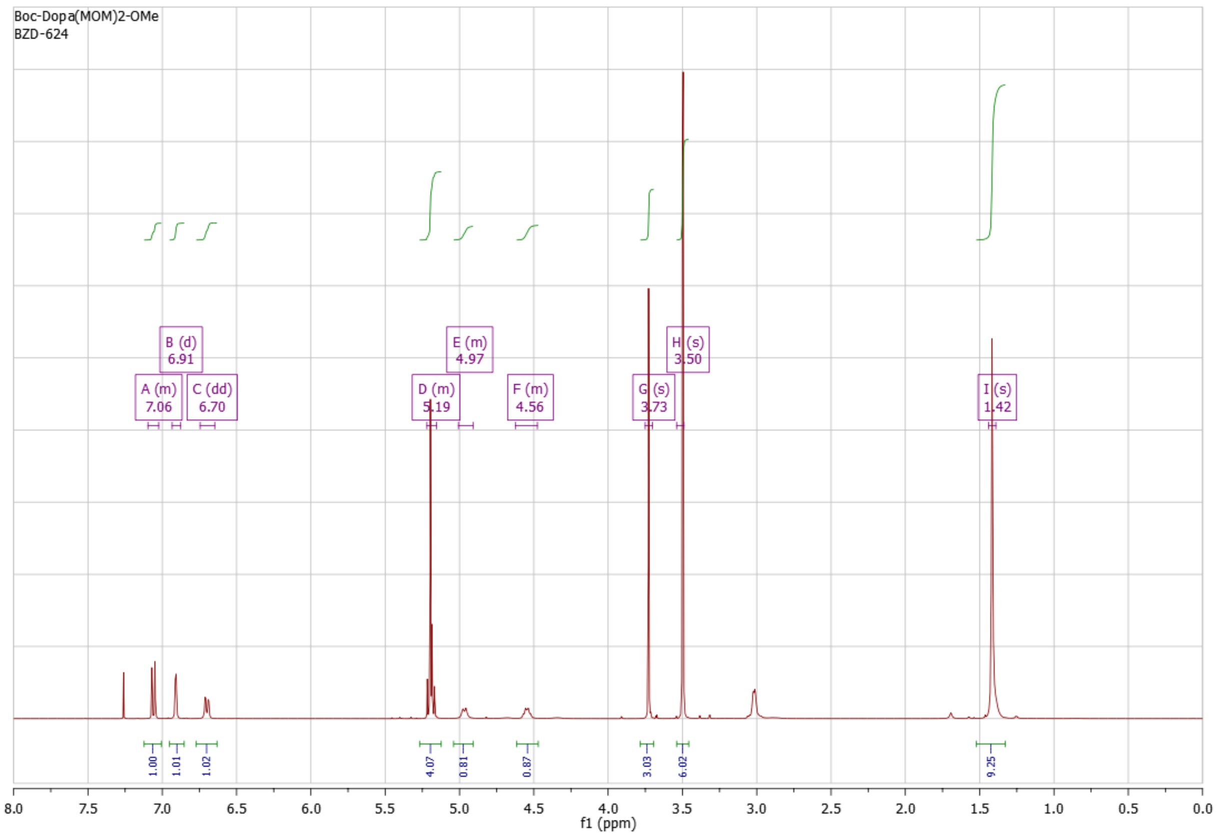

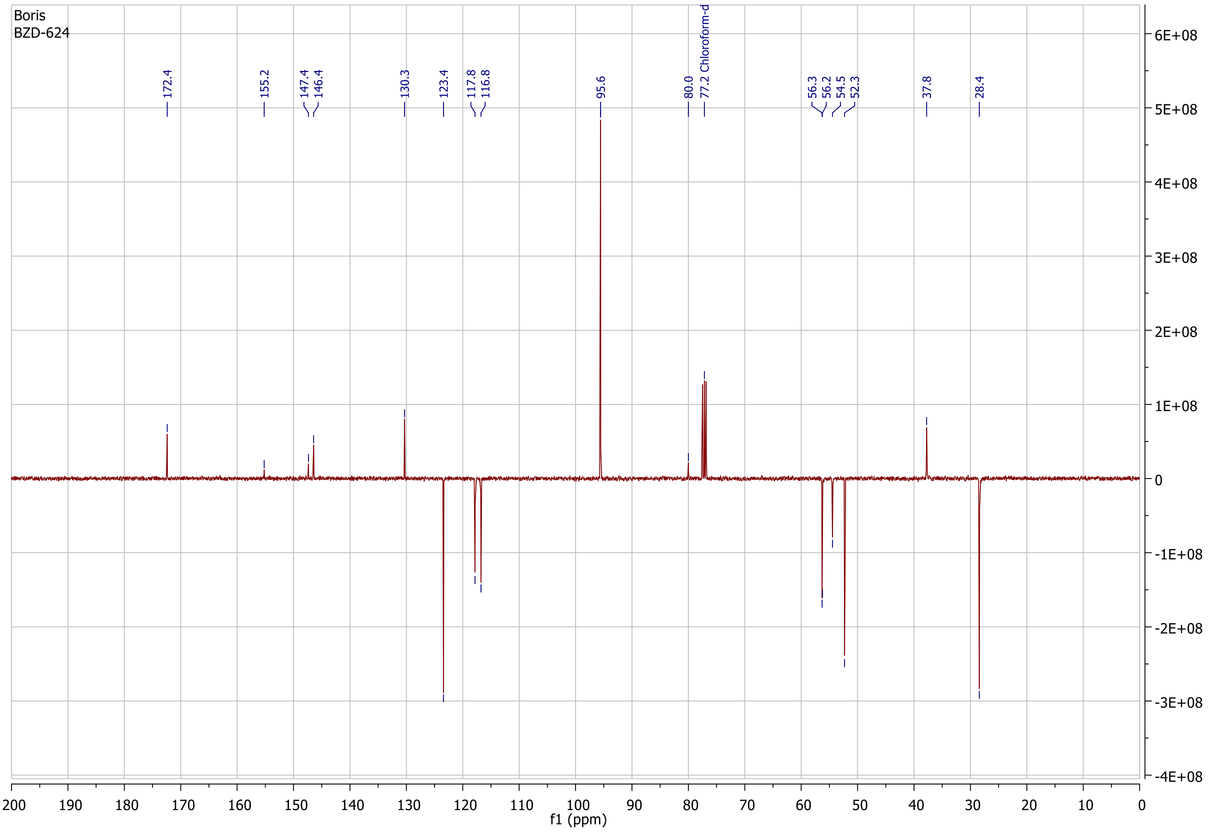

Boc-6-IDOPA(MOM)$_2$-OMe (S2)

Iodine (8.63 g, 34.0 mmol, 1 eq.) was added to an ice-cold solution of **S1** (13.6 g, 34.0 mmol, 1 eq.) in anhydrous CH_2Cl_2 (160 mL) and the mixture was stirred until the iodine was completely dissolved. Thereafter, PIFA (17.5 g, 40.7 mmol, 1.2 eq.) was added and the reaction mixture was stirred for 15 min. Afterwards, the cooling bath was removed, and the mixture was stirred for 90 min. A saturated solution of $Na_2S_2O_3$ (100 mL) was added and stirring continued for 10 min. The organic layer was separated, washed with 10% $NaHCO_3$ (3×50 mL) and brine (2×30 mL), dried, filtered and concentrated under reduced pressure. The residue was purified by column chromatography (EtOAc:hexane 1:1; R_f=0.57) followed by recrystallization from Et_2O/hexane to afford the title compound as a colorless solid (8.5 g, 16.2 mmol, 48%). The mother liquor was concentrated under reduced pressure and the residue was purified by column chromatography followed by recrystallization from Et_2O/hexane to afford a second crop of the title compound (2.2 g, overall 56%). Noteworthy, **S1** and **S2** were inseparable from each other on silica gel TLC plates or columns. ^{1}H NMR (400 MHz, $CDCl_3$; mixture of two rotamers) δ = 7.53 (s, 1H), 6.98 (s, 1H), 5.18 (s, 4H), 5.08 – 4.95 (m, 1H), 4.70 – 4.42 (m, 1H), 3.75 (s, 3H), 3.51 (s, 3H), 3.49 (s, 3H), 3.21 (dd, J = 14.0, 5.7 Hz, 1H), 3.10 – 2.70 (m, 1H), 1.47 – 1.19 (m, 9H). ^{13}C NMR (101 MHz, $CDCl_3$) δ 172.4, 155.1, 147.6, 146.9, 133.5, 127.0, 118.1, 95.6, 95.6, 91.2, 80.0, 56.5, 56.4, 53.9, 52.5, 42.5, 28.4. LR-MS: m/z $[M+Na]^+$ calculated for

$[C_{19}H_{28}INO_8+Na]^+$ = 548.08, found: 547.92 $[M+H]^+$; calculated for $[C_{19}H_{28}INO_8+H]^+$ = 526.1, found: 525.92.

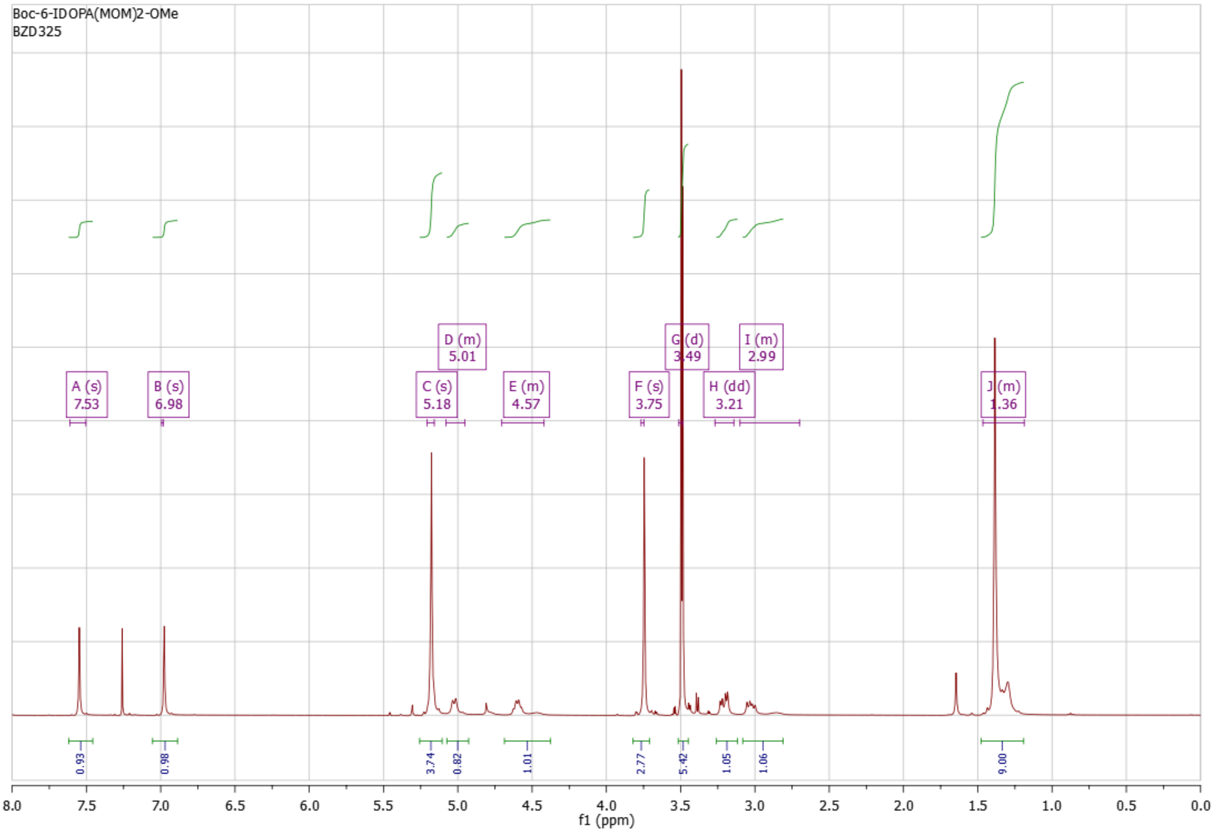

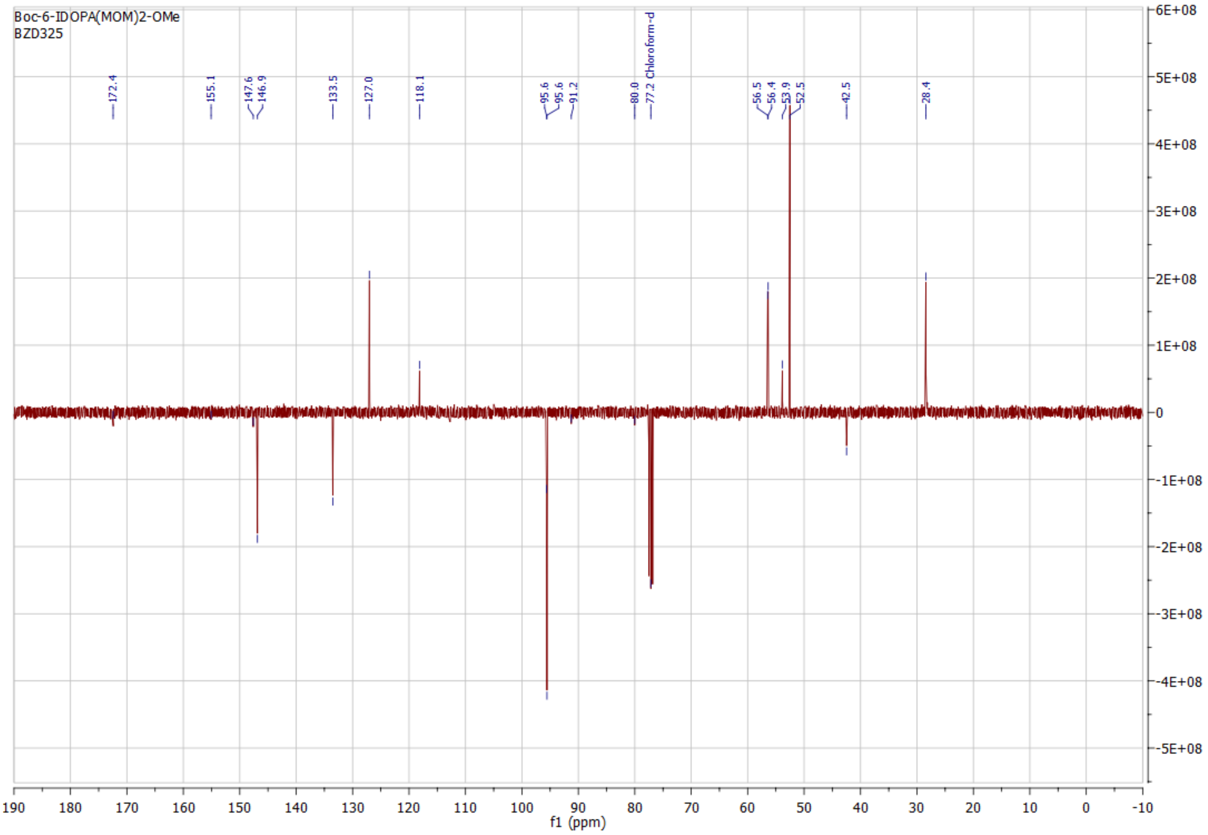

Boc-6-BpinDOPA(MOM)$_2$-OMe (S3)

A suspension of AcOK (1.68 g, 17.1mmol, 3 eq.) in a solution of **S2** (3.00 g, 5.71 mmol, 1 eq.), Pd(dppf)Cl_2 (0.37 g, 0.46 mmol, 0.08 eq.) and $(Bpin)_2$ (5.08 g, 10.0 mmol, 1.75 eq.) in anhydrous DMF (12 mL) was placed into an oil bath preheated to 65 °C and the reaction mixture was stirred for 4 days. All volatiles were removed under reduced pressure, the residue was dried at 1 mbar and 60 °C, taken up in EtOAc/hexane = 1:6, and the resulting suspension was purified by column chromatography on silica gel (EtOAc:hexane 1:1; R_f=0.57) followed by column chromatography on C_{18} phase (dry loading; 20% → 100% MeCN) to afford the title compound (0.9 g, 30%; contained ca. 20 mol.% MeCN according to its ^{1}H-NMR spectrum) as a colorless oil. Noteworthy, **S1**, **S2** and **S3** were inseparable from each other on normal phase silica gel TLC plates or columns. ^{1}H NMR (400 MHz, $CDCl_3$; mixture of two rotamers) δ = 7.51 (s, 1H), 7.01 (s, 1H), 5.98 (d, *J* = 7.9 Hz, 1H), 5.34 – 5.18 (m, 4H), 4.32 (ddd, *J* = 10.4, 8.1, 4.2 Hz, 1H), 3.75 (s, 3H), 3.52 (s, 3H), 3.50 (s, 3H), 3.30 – 3.08 (m, 2H), 1.36 (s, 9H), 1.35 (s, 6H), 1.34 (s, 6H). ^{13}C NMR (101 MHz, $CDCl_3$): δ = 173.4, 155.9, 150.1, 145.3, 139.2, 124.0, 117.8, 95.6, 95.1, 84.2, 79.4, 56.5, 56.4, 52.2, 36.6, 28.4, 25.1, 24.8. *C*-Bpin was not observed. LR-MS: m/z $[M+Na]^+$ calculated for $[C_{25}H_{40}BNO_{10}+Na]^+$ = 548.26, found: 548.16; $[M+H]^+$ calculated for $[C_{25}H_{41}BNO_{10}+H]^+$ = 526.28, found: 526.1.

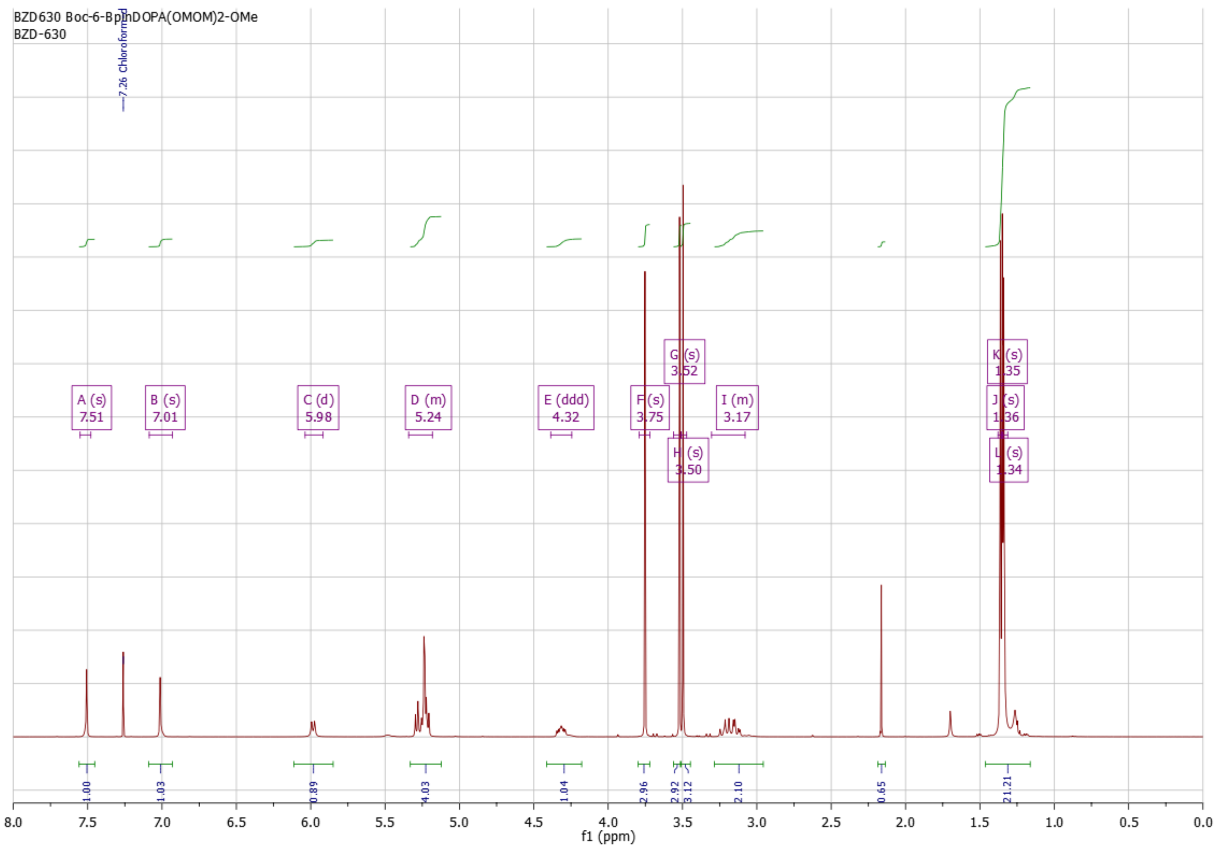
BZD630 Boc-6-BpinDOPA(OMOM)2-OMe
BZD-630
7.26 Chloroform-d
A (s) 7.51
B (s) 7.01
C (d) 5.98
D (m) 5.24
E (ddd) 4.32
F (s) 3.75
G (s) 3.52
H (s) 3.50
I (m) 3.17
K (s) 1.35
J (s) 1.36
L (s) 1.34
1.00
1.03
0.89
4.03
1.04
2.96
2.92
3.12
2.10
0.65
21.21
f1 (ppm)

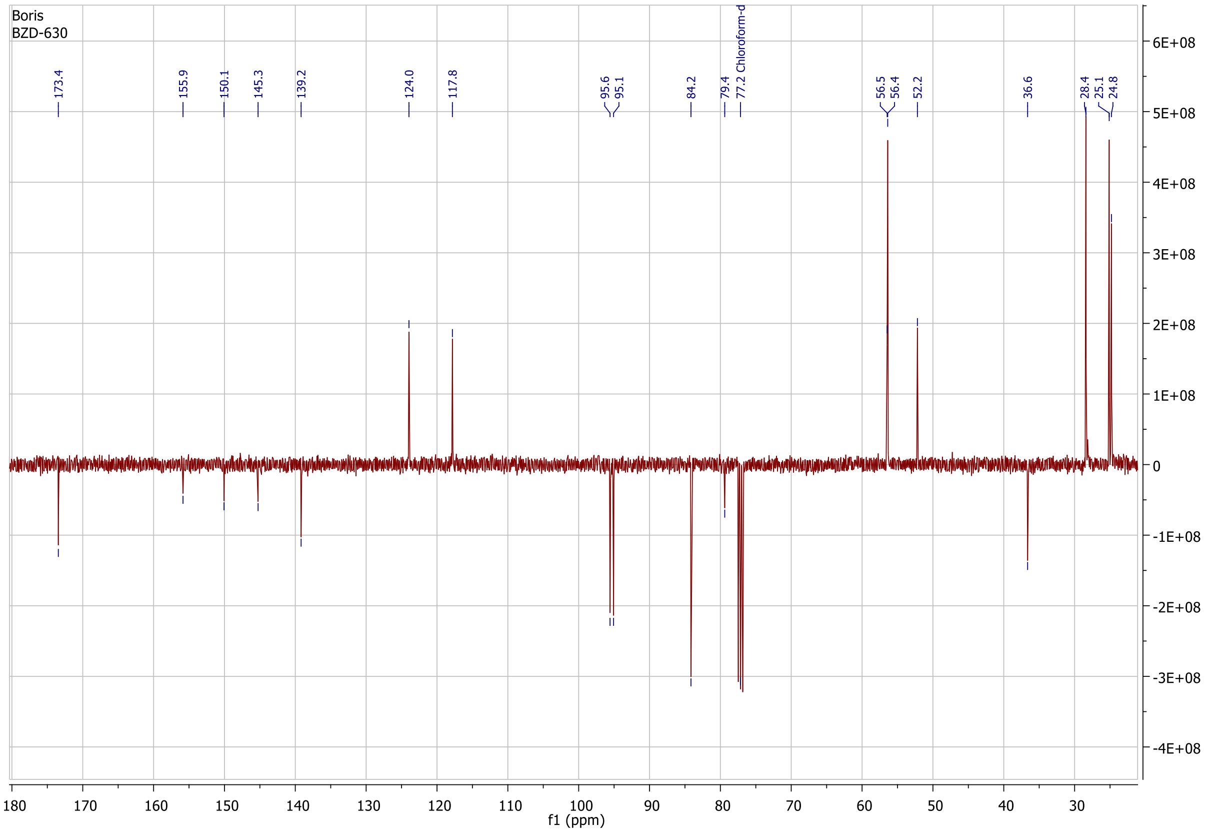
Boris
BZD-630
173.4
155.9
150.1
145.3
139.2
124.0
117.8
95.6
95.1
84.2
79.4
77.2 Chloroform-d
56.5
56.4
52.2
36.6
28.4
25.1
24.8
f1 (ppm)

(Boc)$_2$-6-BpinDOPA(MOM)$_2$-OMe (S4)

DMAP (30 mg, 0.25 mmol, 0.1 eq.) was added to a solution of **S3** (1.2 g, 2.3 mmol, 1 eq.) and Boc$_2$O (2.2 g, 10 mmol, 4.3 eq.) in anhydrous MeCN (7 mL) and the reaction mixture was manually stirred until the DMAP had dissolved. Thereafter, the mixture was left to stay at ambient temperature for 4 days. All volatiles were removed under reduced pressure and the residue was purified by column chromatography (EtOAc/hexane = 1:3, R_f = 0.35) followed by low-temperature recrystallization from pentane (–25 ºC) to afford the title compound as a colorless solid (1.2 g, 1.9 mmol, 83%). ^{1}H NMR (400 MHz, $CDCl_3$) δ = 7.49 (s, 1H), 6.87 (s, 1H), 5.28 (dd, *J* = 11.4, 3.9 Hz, 1H), 5.24 (dd, *J* = 6.5, 3.8 Hz, 2H), 5.16 (dd, *J* = 12.9, 6.5 Hz, 2H), 3.97 (dd, *J* = 13.5, 3.9 Hz, 1H), 3.75 (s, 3H), 3.49 (s, 3H), 3.46 (s, 3H), 3.04 (dd, *J* = 13.5, 11.4 Hz, 1H), 1.33 (s, 18H), 1.31 (s, 6H), 1.29 (s, 6H). ^{13}C NMR (101 MHz, $CDCl_3$): δ = 171.1, 151.7, 149.7, 144.9, 140.1, 124.3, 119.1, 95.5, 95.3, 83.6, 82.6, 60.1, 56.3, 56.1, 52.1, 35.5, 27.9, 25.1, 24.9. *C*-Bpin was not observed. LR-MS: m/z $[M+Na]^+$ calculated for $[C_{30}H_{48}BNO_{12}+Na]^+$ = 648.33, found: 648.38; $[M+H]^+$ calculated for $[C_{30}H_{48}BNO_{12}+H]^+$ = 626.34, found: 626.38. HR-MS: m/z $[M+Na]^+$ calculated for $[C_{30}H_{48}BNO_{12}+Na]^+$ = 648.31618, found: 648.31687.

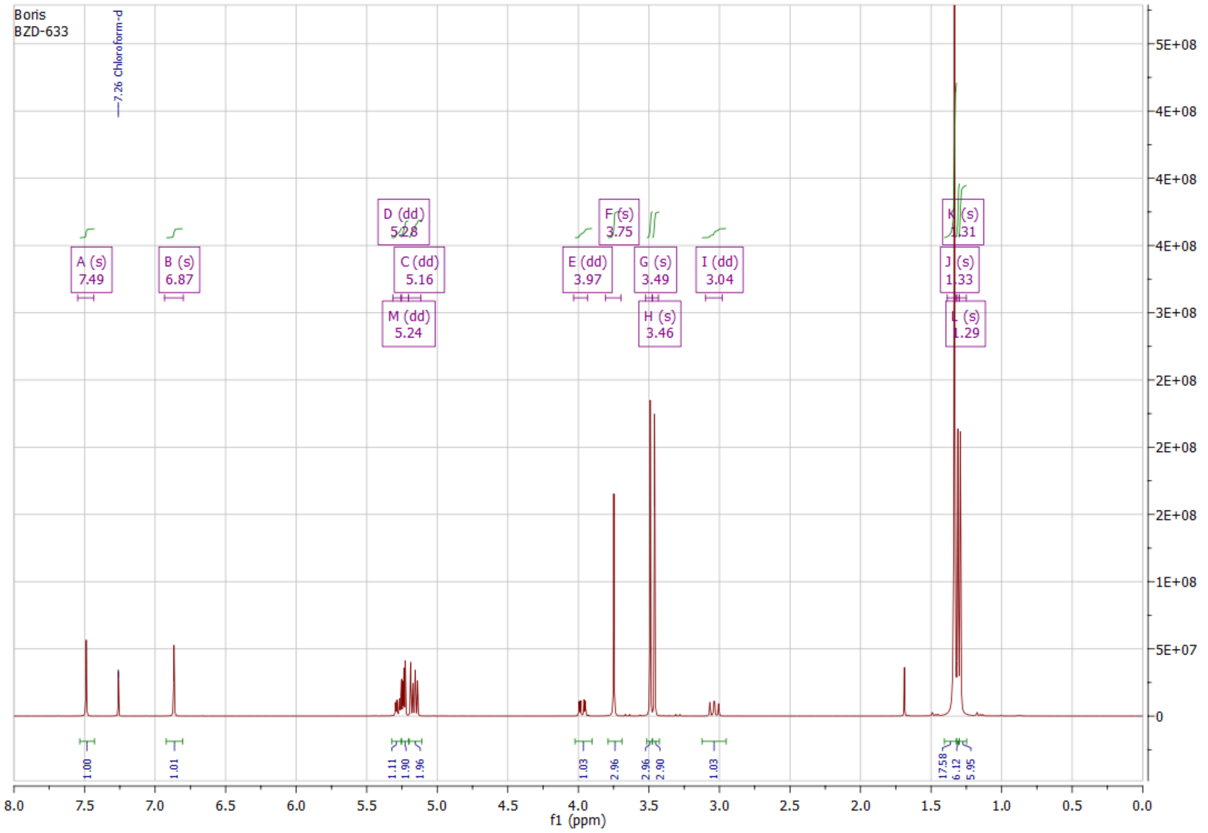
Boris
BZD-633
7.26 Chloroform-d
A (s) 7.49
B (s) 6.87
D (dd) 5.28
C (dd) 5.16
M (dd) 5.24
E (dd) 3.97
F (s) 3.75
G (s) 3.49
H (s) 3.46
I (dd) 3.04
K (s) 1.31
J (s) 1.33
L (s) 1.29
1.00
1.01
1.11
1.90
1.96
1.03
2.96
2.96
2.90
1.03
17.58
6.12
5.95
f1 (ppm)

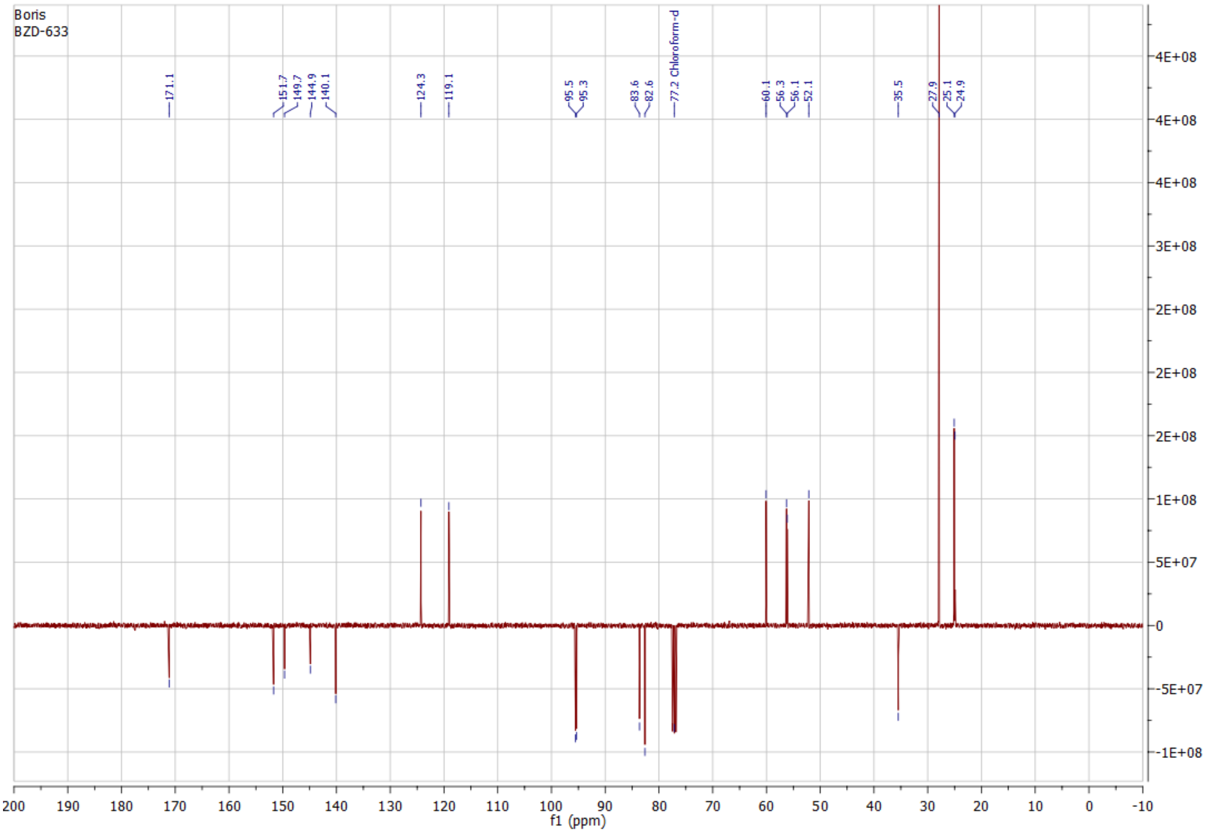
Boris
BZD-633
171.1
151.7
149.7
144.9
140.1
124.3
119.1
95.5
95.3
83.6
82.6
77.2 Chloroform-d
60.1
56.3
56.1
52.1
35.5
27.9
25.1
24.9
f1 (ppm)

2.6 Preparation of $(Boc)_2$-6-$SnMe_3$DOPA$(MOM)_2$-OMe (S6)

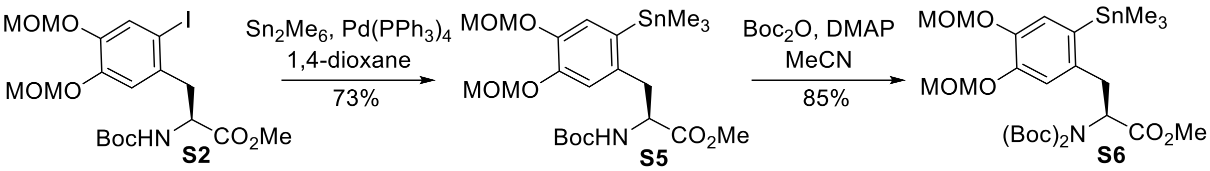

Scheme S2: Preparation of $(Boc)_2$-6-$SnMe_3$DOPA$(MOM)_2$-OMe (**S6**).

Boc-6-$SnMe_3$DOPA$(MOM)_2$-OMe (S5)

A solution of **S2** (2.3 g, 4.3 mmol, 1 eq.), Sn_2Me_6 (3.5 g, 11 mmol, 2.6 eq.) and $Pd(PPh_3)_4$ (0.50 g, 0.43 mmol, 0.1 eq.) in anhydrous 1,4-dioxane (10 mL) was stirred at 70 ºC for 24 h and thereafter at 90 ºC for 3 h. The reaction mixture was concentrated under reduced pressure and the residue was dried at 1 mbar and 60 ºC for 2 h. Thereafter, the crude product was purified by column chromatography (EtOAc:hexane = 1:3, R_f = 0.26) to afford the title compound (1.8 g, 73%) as a yellow oil, which was directly used for the next step without further purification and characterization.

$(Boc)_2$-6-$SnMe_3$DOPA$(MOM)_2$-OMe (S6)

DMAP (38 mg, 0.31 mmol, 0.1 eq.) was added to a solution of **S5** (1.8 g, 3.1 mmol, 1 eq.) and Boc_2O (2.2 g, 9.4 mmol, 3 eq.) in anhydrous MeCN (8 mL) and the reaction mixture was manually stirred until DMAP had dissolved. Thereafter, the mixture was left to stay at ambient temperature for 4 days. All volatiles were removed under reduced pressure and the residue was purified by column chromatography (EtOAc/hexane = 1:3, R_f = 0.54) to afford the title compound (1.7 g, 2.6 mmol, 84%) as a yellow oil. ^{1}H NMR (400 MHz, $CDCl_3$) δ = 7.15 (s, 1H), 6.93 (s, 1H), 5.26 (d, J = 6.6 Hz, 1H), 5.21 – 5.11 (m, 3H), 4.94 (td, J = 7.8, 4.9 Hz, 1H), 3.75 (s, 3H), 3.49 (s, 3H), 3.47 (s, 3H), 3.32 – 3.25 (m, 2H), 1.37 (s, 18H), 0.31 (s, 9H). ^{13}C NMR (101 MHz, $CDCl_3$): δ = 170.8, 151.7, 147.9, 145.4, 139.1, 135.3, 124.7, 118.2, 95.8, 95.4, 83.1, 60.0, 56.2, 56.1, 52.4, 38.1, 27.9, –8.1. HR-MS: m/z $[M+H]^+$ calculated for $[C_{27}H_{45}SnNO_{10}+H]^+$ = 664.21382, found: 664.21345. Correct isotopic pattern.

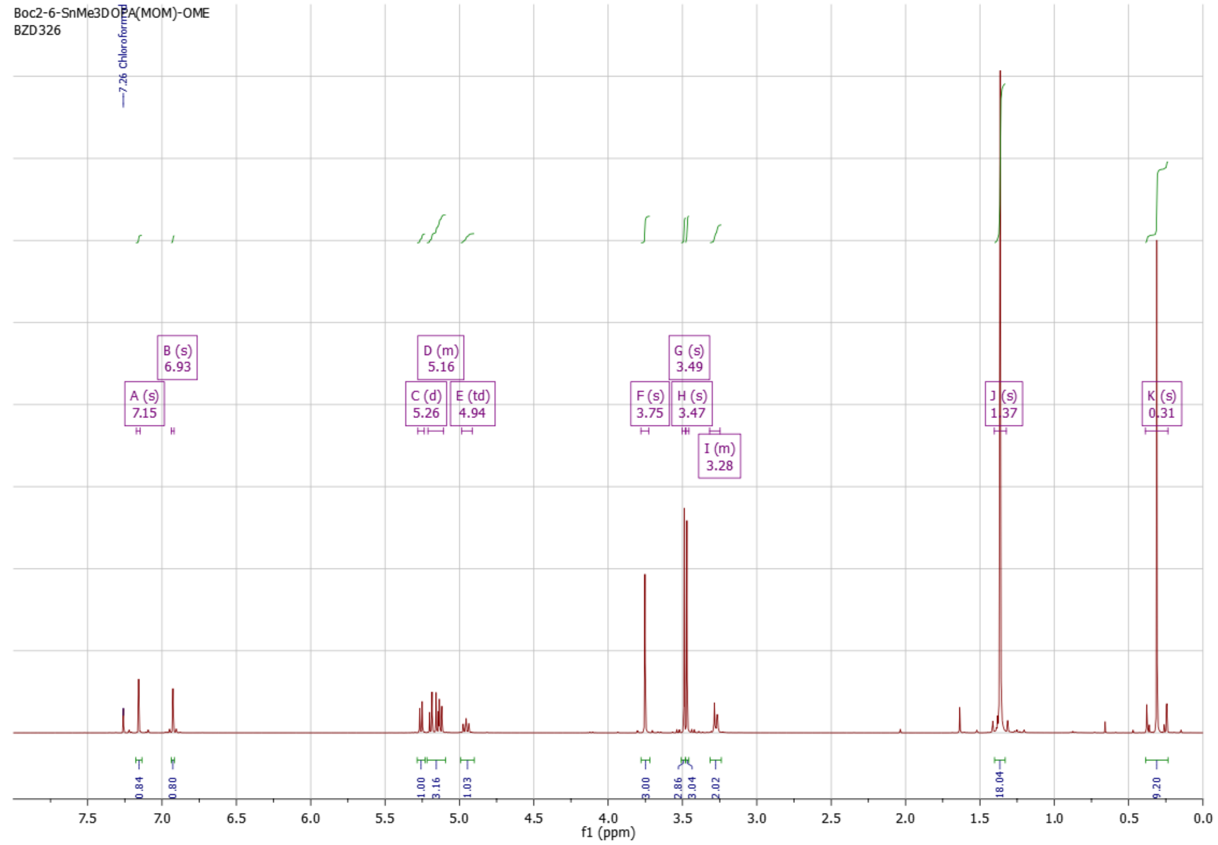
Boc2-6-SnMe3DOPA(MOM)-OME
BZD326
7.26 Chloroform
A (s) 7.15
B (s) 6.93
C (d) 5.26
D (m) 5.16
E (td) 4.94
F (s) 3.75
G (s) 3.49
H (s) 3.47
I (m) 3.28
J (s) 1.37
K (s) 0.31
0.84
0.80
1.00
3.16
1.03
3.00
2.86
3.04
2.02
18.04
9.20
f1 (ppm)

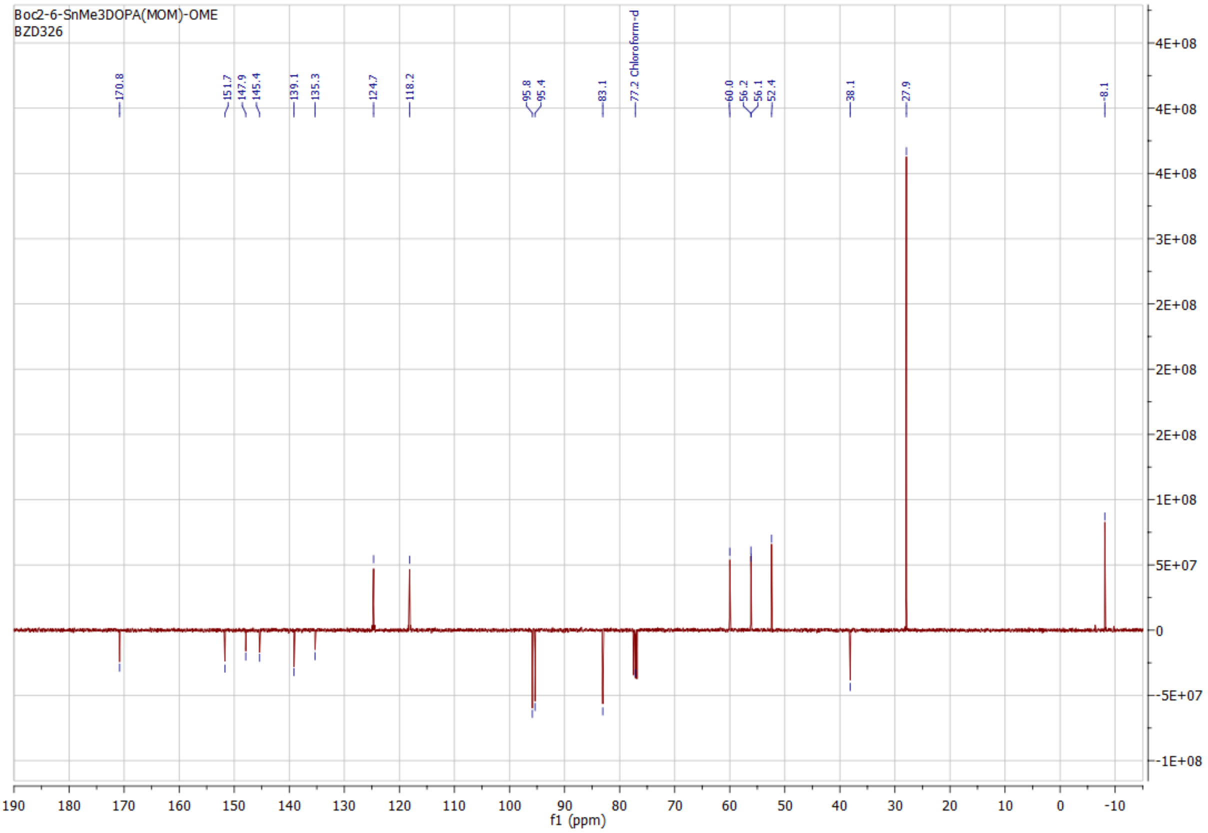
Boc2-6-SnMe3DOPA(MOM)-OME
BZD326
170.8
151.7
147.9
145.4
139.1
135.3
124.7
118.2
95.8
95.4
83.1
77.2 Chloroform-d
60.0
56.2
56.1
52.4
38.1
27.9
-8.1
f1 (ppm)

2.7 Preparation of $B(OH)_2$ substituted Ni(II)-BPX complexes – General Procedure 4 (GP4)

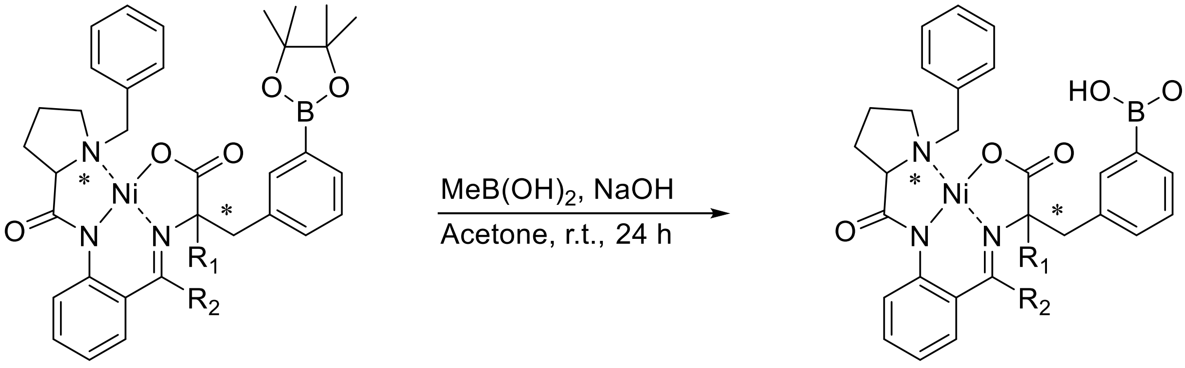

R$_1$ = H, R$_2$ = Ph [(*S*,*S*)-**11**]
R$_1$ = H, R$_2$ = Ph [(*R*,*R*)-**11**]
R$_1$ = Me, R$_2$ = H [(*R*,*R*)-**13**]

R$_1$ = H, R$_2$ = Ph [(*S*,*S*)-**10**]
R$_1$ = H, R$_2$ = Ph [(*R*,*R*)-**10**]
R$_1$ = Me, R$_2$ = H [(*S*,*S*)-**12**]

Scheme 3: Preparation of $B(OH)_2$ substituted Ni(II)-BPX complexes.

$B(OH)_2$-substituted Ni(II)-BPX complexes were prepared according to the literature.[23] 0.1 M NaOH (0.2 eq.) was added to a solution of the respective BPin substituted Ni-BPX complex (0.6 M, 1 eq.) and methylboronic acid (10 eq.) in acetone and the reaction mixture was stirred for 24 h. Afterwards, the mixture was neutralized by addition of 0.1 M HCl (0.15 eq.) and all volatiles were removed under reduced pressure. The crude product was purified by reversed phase flash column chromatography (MeCN/H_2O).

(*S*,*S*)-Ni-BPB-3-B(OH)$_2$Phe [(*S*,*S*)-10]

The title compound was obtained as a red solid (337 mg, 0.53 mmol, 85%) according to GP4 from (*S*,*S*)-Ni-BPB-3-BPinPhe[4] (450 mg, 0.63 mmol). ^{1}H NMR (400 MHz, THF-d_8) δ = 8.40 (dd, *J* = 8.8, 1.0 Hz, 1H), 8.14 – 8.04 (m, 2H), 7.86 (d, *J* = 7.4 Hz, 1H), 7.69 (s, 1H), 7.62 – 7.42 (m, 4H), 7.37 – 7.20 (m, 6H), 7.10 (ddd, *J* = 7.4, 2.8, 1.5 Hz, 2H), 7.02 (ddd, *J* = 8.7, 6.8, 1.8 Hz, 1H), 6.65 (dd, *J* = 8.3, 1.7 Hz, 1H), 6.56 (ddd, *J* = 8.2, 6.8, 1.2 Hz, 1H), 4.13 (dd, *J* = 11.0, 6.6 Hz, 2H), 3.42 (d, *J* = 12.5 Hz, 1H), 3.23 (t, *J* = 8.3 Hz, 1H), 3.00 (ddd, *J* = 17.5, 11.4, 4.2 Hz, 2H), 2.79 (dd, *J* = 13.5, 5.6 Hz, 1H), 2.20 – 2.13 (m, 3H), 2.02 – 1.92 (m, 1H), 1.58 – 1.49 (m, 1H). ^{13}C NMR (101 MHz, THF-d_8) δ = 181.1, 178.0, 171.8, 145.2, 137.2, 136.2 (×2), 135.4, 134.4, 134.1, 133.4, 132.7, 132.4, 130.4, 130.0, 129.8, 129.4 (×2), 129.3, 128.5 (×2), 127.1, 124.5, 120.3, 72.6, 71.1, 63.9, 58.5, 40.4, 31.7, 24.2. HR-MS: m/z $[M+H]^+$ calculated for $[C_{34}H_{31}BN_3NiO_5+H]^+$ = 631.18976, found: 631.19052 $[M+H^+]$. Correct isotopic pattern.

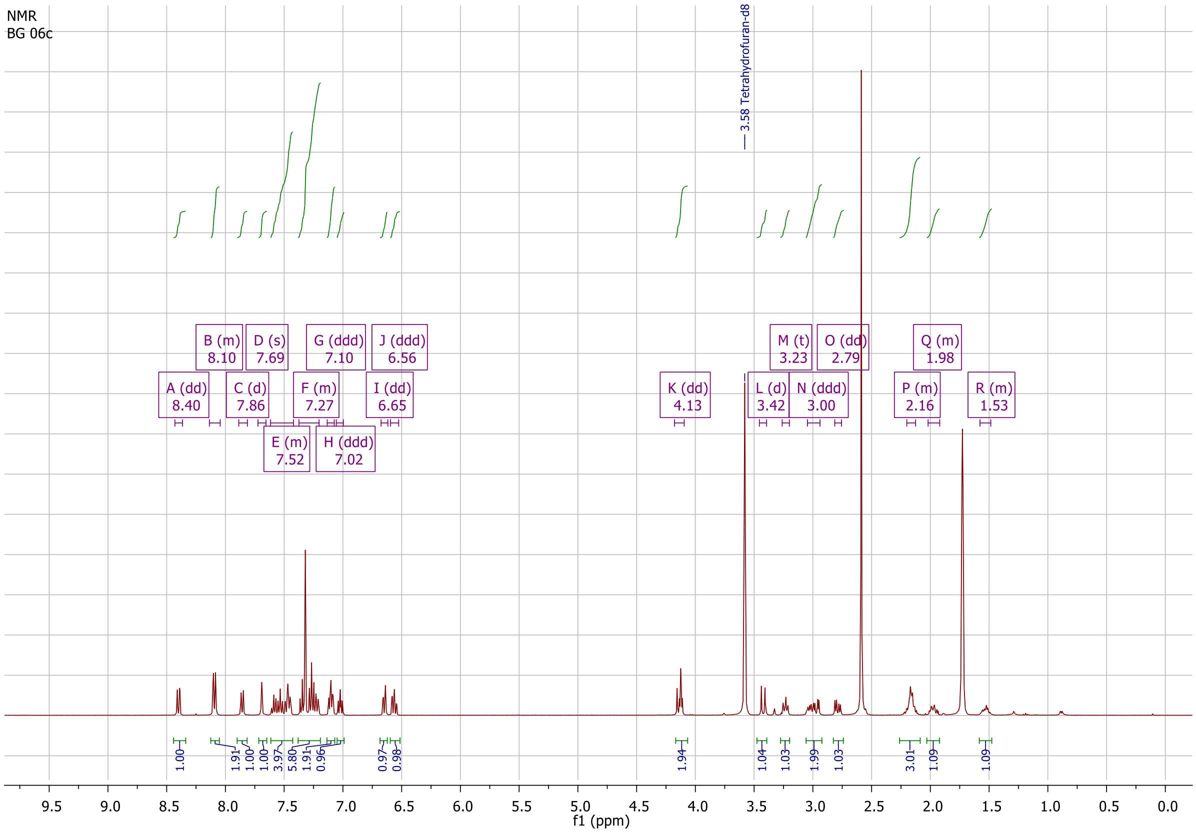
NMR
BG 06c
3.58 Tetrahydrofuran-d8
A (dd) 8.40
B (m) 8.10
C (d) 7.86
D (s) 7.69
E (m) 7.52
F (m) 7.27
G (ddd) 7.10
H (ddd) 7.02
I (dd) 6.65
J (ddd) 6.56
K (dd) 4.13
L (d) 3.42
M (t) 3.23
N (ddd) 3.00
O (dd) 2.79
P (m) 2.16
Q (m) 1.98
R (m) 1.53
f1 (ppm)

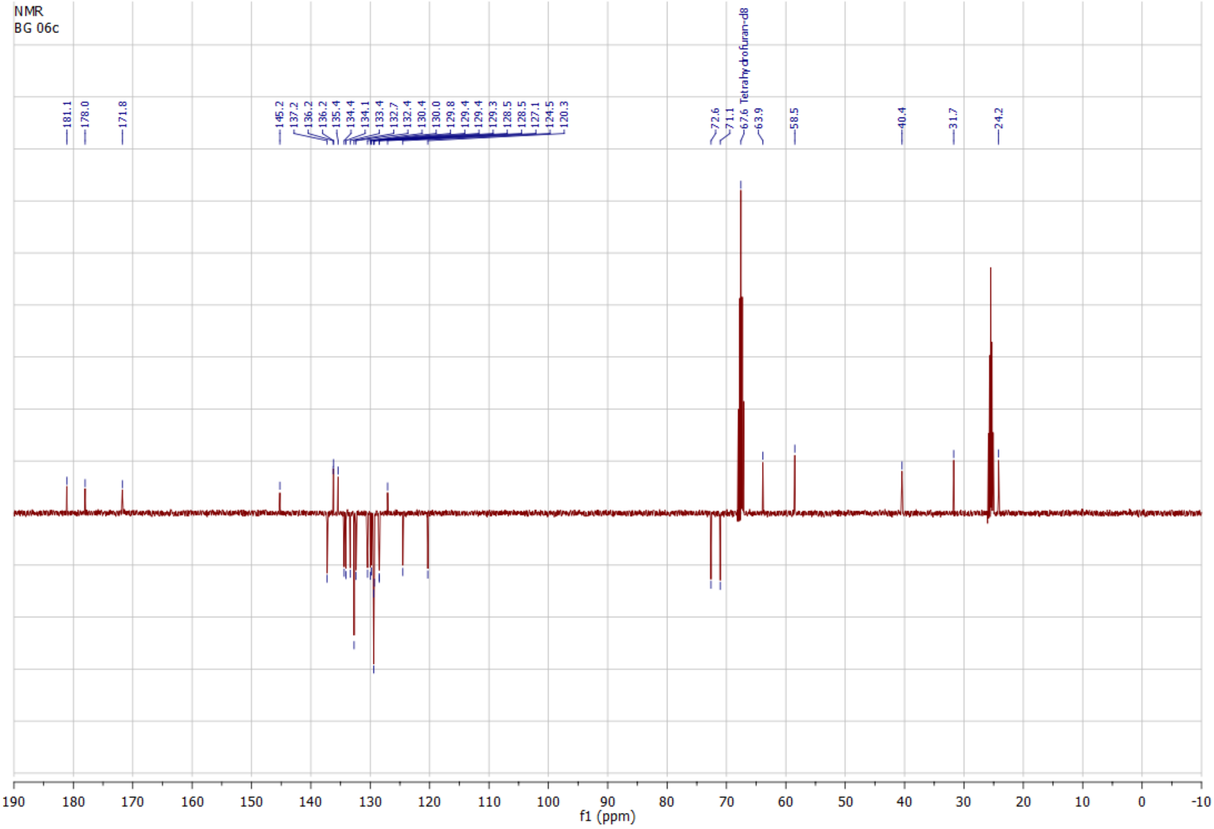
NMR
BG 06c
181.1
178.0
171.8
145.2
137.2
136.2
135.4
134.4
134.1
133.4
132.7
132.4
130.4
130.0
129.8
129.4
129.4
129.3
128.5
127.1
124.5
120.3
72.6
71.1
67.6 Tetrahydrofuran-d8
63.9
58.5
40.4
31.7
24.2
f1 (ppm)

(*R*,*R*)-Ni-BPB-3-B(OH)$_2$Phe [(*R*,*R*)-10]

The title compound was obtained as a red solid (357 mg, 0.63 mmol, 90%) according to GP4 from (*R*,*R*)-Ni-BPB-3-BPinPhe[4] (450 mg, 0.63 mmol). ^{1}H NMR (400 MHz, THF-d_8) δ = 8.40 (dd, *J* = 8.8, 0.9 Hz, 1H), 8.12 – 8.06 (m, 2H), 7.85 (t, *J* = 9.5 Hz, 1H), 7.68 (d, *J* = 12.9 Hz, 1H), 7.61 – 7.43 (m, 4H), 7.38 – 7.18 (m, 6H), 7.14 – 7.08 (m, 2H), 7.02 (ddd, *J* = 8.7, 6.8, 1.8 Hz, 1H), 6.65 (dd, *J* = 8.3, 1.7 Hz, 1H), 6.56 (ddd, *J* = 8.2, 6.8, 1.2 Hz, 1H), 4.17 – 4.09 (m, 2H), 3.42 (d, *J* = 12.5 Hz, 1H), 3.22 (d, *J* = 7.2 Hz, 1H), 3.06 – 2.94 (m, 2H), 2.79 (dd, *J* = 13.5, 5.6 Hz, 1H), 2.24 – 2.11 (m, 3H), 1.98 (ddd, *J* = 16.4, 11.1, 5.7 Hz, 1H), 1.53 (td, *J* = 11.0, 5.3 Hz, 1H). ^{13}C NMR (101 MHz, THF-d_8) δ = 181.1, 178.1, 171.8, 145.2, 137.3, 136.2 (×2), 135.4, 134.4, 134.1, 133.4, 132.7, 132.4, 130.5, 130.0, 129.8, 129.7, 129.4, 129.3, 128.5 (×2), 127.1, 124.5, 120.3, 72.6, 71.1, 63.9, 58.5, 40.4, 31.7, 24.2. HR-MS: m/z [M+H]$^+$ calculated for [$C_{34}H_{31}BN_3NiO_5$+H]$^+$ = 631.18976, found: 631.19045 [M+H$^+$]. Correct isotopic pattern.

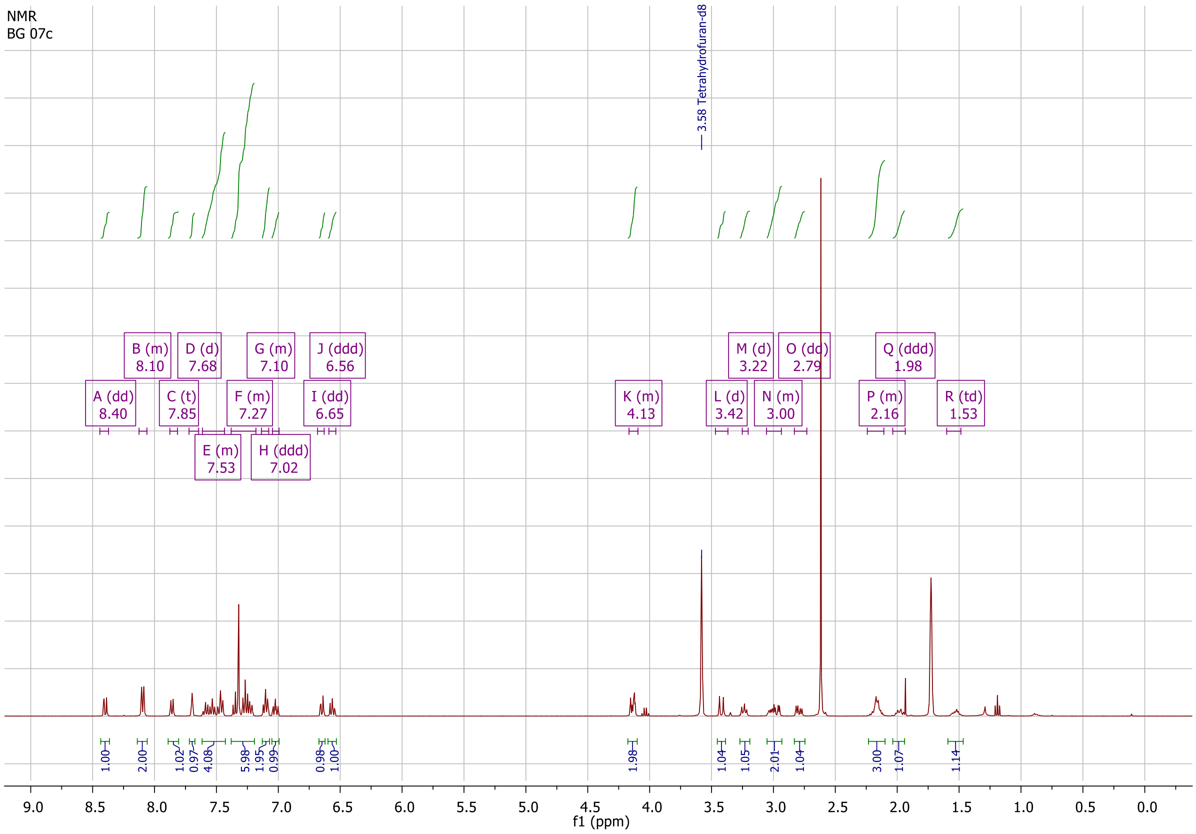

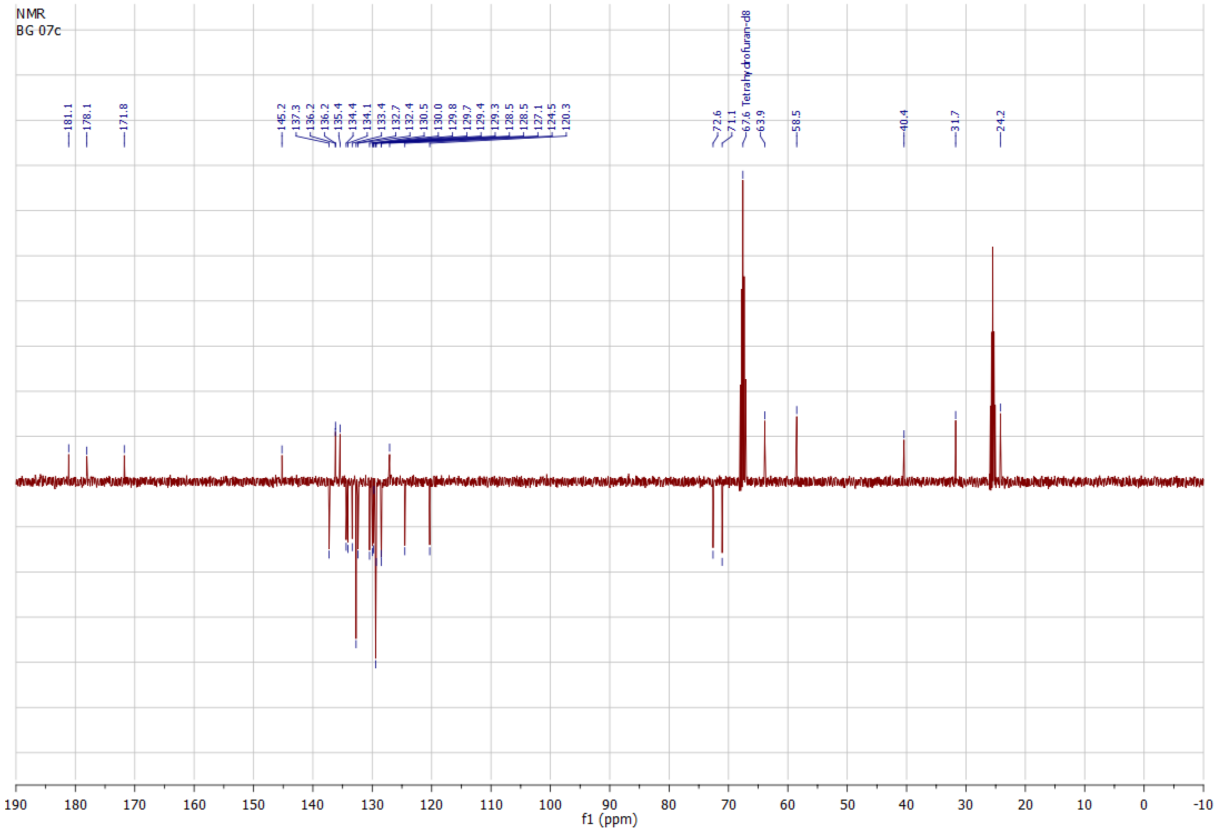

Synthesis of (*S*,*S*)-Ni-BPA-α-methyl-3-B(OH)$_2$Phe [(*S*,*S*)-12]

The title compound was obtained as a red solid (370 mg, 0.65 mmol, 83%) according to GP4 from (*S*,*S*)-Ni-BPA-α-methyl-3-BPinPhe[4] (510 mg, 0.78 mmol). ^{1}H NMR (400 MHz, $CDCl_3$) δ = 8.48 (d, *J* = 8.6 Hz, 1H), 8.03 – 7.90 (m, 3H), 7.72 (d, *J* = 19.9 Hz, 1H), 7.62 – 7.54 (m, 1H), 7.47 – 7.16 (m, 7H), 7.14 (t, *J* = 8.2 Hz, 1H), 7.01 – 6.84 (m, 1H), 4.20 – 4.07 (m, 2H), 3.48 (d, *J* = 12.7 Hz, 1H), 3.35 (d, *J* = 13.4 Hz, 1H), 3.23 (dd, *J* = 9.4, 6.6 Hz, 1H), 2.98 (dd, *J* = 10.1, 5.8 Hz, 1H), 2.86 (d, *J* = 13.5 Hz, 1H), 2.29 – 1.99 (m, 2H), 1.92 – 1.78 (m, 1H), 1.68 – 1.56 (m, 4H), 1.50 – 1.40 (m, 1H), 1.31 – 1.21 (m, 3H), 0.89 (ddt, *J* = 46.7, 43.1, 19.6 Hz, 1H). ^{13}C NMR (101 MHz, $CDCl_3$) δ = 182.2, 181.8, 161.4, 142.8, 136.3, 134.4, 134.1, 133.9, 133.7, 133.3, 132.9, 131.8, 129.1, 128.9, 128.1, 124.0, 123.5, 121.6, 74.9, 70.4, 63.2, 60.5, 60.0, 57.4, 48.3, 30.9, 29.8, 25.4, 23.2, 21.2, 14.3 (×2). HR-MS: m/z $[M+H]^+$ calculated for $[C_{29}H_{29}BN_3NiO_5+H]^+$ = 569.17411, found: 569.17416. Correct isotopic pattern.

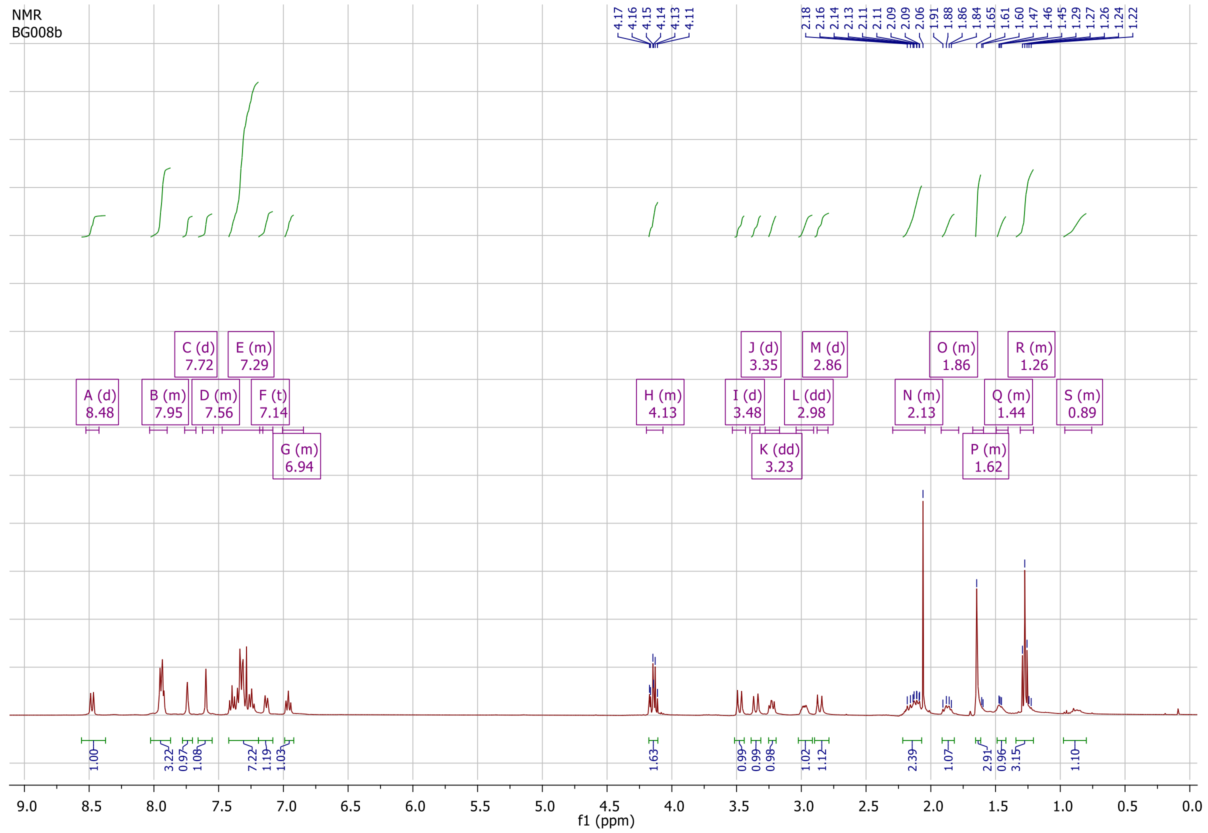
NMR
BG008b
A (d) 8.48
B (m) 7.95
C (d) 7.72
D (m) 7.56
E (m) 7.29
F (t) 7.14
G (m) 6.94
H (m) 4.13
I (d) 3.48
J (d) 3.35
K (dd) 3.23
L (dd) 2.98
M (d) 2.86
N (m) 2.13
O (m) 1.86
P (m) 1.62
Q (m) 1.44
R (m) 1.26
S (m) 0.89
f1 (ppm)

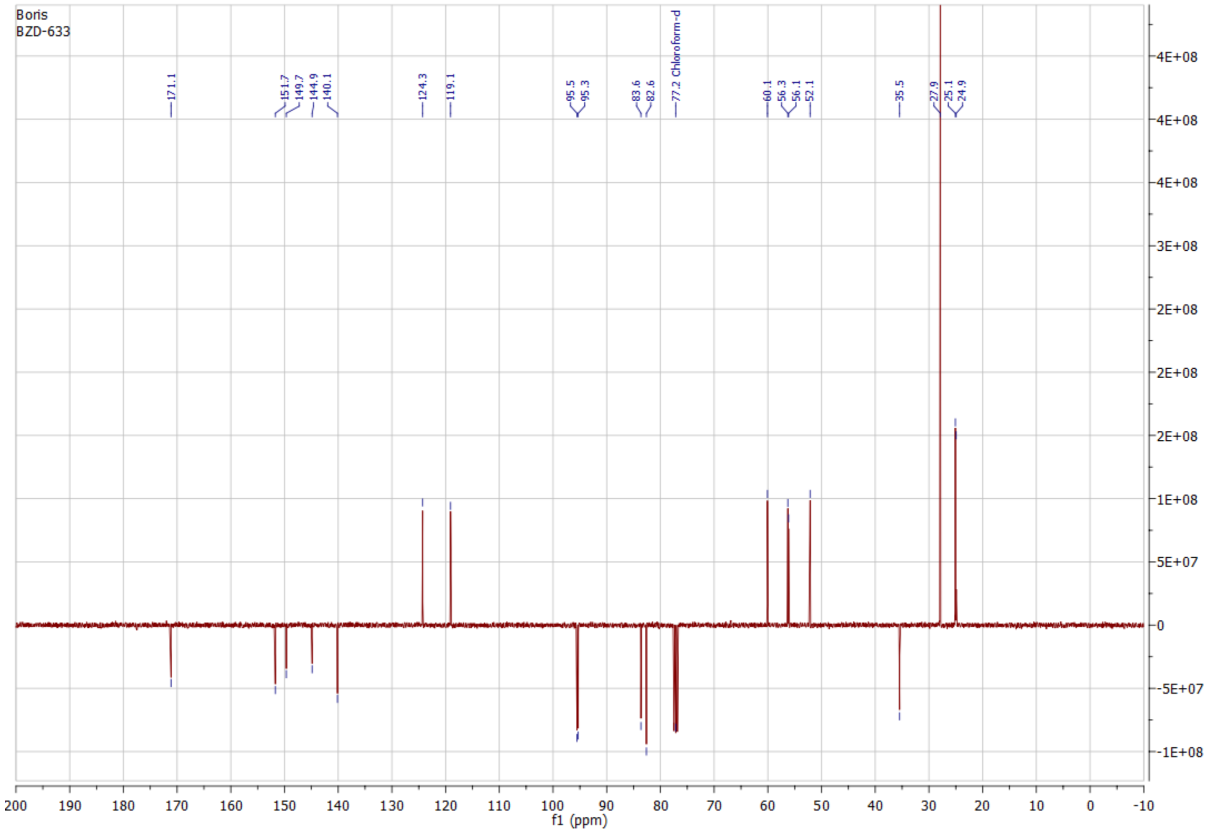
Boris
BZD-633
171.1
151.7
149.7
144.9
140.1
124.3
119.1
95.5
95.3
83.6
82.6
77.2 Chloroform-d
60.1
56.3
56.1
52.1
35.5
27.9
25.1
24.9
f1 (ppm)

3 Radiochemistry

3.1 General conditions

[^{18}F]Fluoride ([^{18}F]F^{-}) was produced via the $^{18}O(p,n)^{18}F$ nuclear reaction by bombardment of enriched [^{18}O]H_2O with 16.5 MeV protons using a BC1710 cyclotron (The Japan Steel Works Ltd., Shinagawa, Japan) at the INM-5 (Forschungszentrum Jülich). All radiosyntheses were carried out in 5 mL Wheaton V-Vials equipped with PTFE wing coated stir bars. Anhydrous solvents (DMA, DMI, PC, *n*BuOH and MeOH, dried over molecular sieves) were purchased from Sigma-Aldrich (Steinheim, Germany) or Acros (Fisher Scientific GmbH, Nidderrau, Germany). Anion exchange resins (Sep-Pak Accell Plus QMA carbonate plus light cartridges 40 mg sorbent per cartridge) and solid phase extraction Sep-Pak C18 and HLB short cartridges (360 mg per cartridge) were obtained from Waters GmbH (Eschborn, Germany). The polymeric-based StrataX cartridge (30 mg) was obtained from Phenomenex (Aschaffenburg, Deutschland).

3.2 Analytical HPLC

Analytical radio-HPLC was performed on a Dionex Ultimate® 3000 HPLC systems (Thermo Fisher Scientific GmbH, Dreieich, Germany) or a HPLC system (Knauer Wissenschaftliche Geräte GmbH, Berlin, Germany) with Azura P 6.1L pump and Azura UVD 2.1S UV/Vis detector. For monitoring absorbance at 254 nm and radioactivity, the UV/Vis detector was coupled in series with a Berthold NaI detector, giving a time of delay of 0.1–0.3 min between the corresponding responses, depending on the flow rate. RCCs were determined by radio-HPLC after dilution of the reaction mixture with H_2O (2 mL) or 20% MeCN (2 mL), through comparison of the peak areas for the radiolabeled product and a post-column injection of the reaction mixture.[24] The identity of radiolabeled products was confirmed by co-injection of the corresponding non-radiolabeled reference compound. Activity yields (AY) were determined by comparing the initial activity on the QMA cartridge and the activity of the radiolabeled product.

HPLC conditions for radiolabeled model compounds: column: Chromolith SpeedROD®, 130 Å, 1.6 µm, 4.6×50 mm (Merck Millipore, Darmstadt, Germany) flow rate: 1.5 mL/min, injection loop: 20 µL.

Radiolabeled product	Mobile phase	t_R [min]
[^{18}F]F-Ph-Ph	50% MeCN	3.5
[^{18}F]F-Ac-Ph	20% MeCN	3.7
[^{18}F]**14**	40% MeCN	5.5
[^{18}F]**15**	10% MeCN	3.5
[^{18}F]**16**	30% MeCN	4.7
[^{18}F]**17**	30% MeCN	4.0
[^{18}F]**18**	30% MeCN	3.5
[^{18}F]**19**	10% MeCN	2.1
[^{18}F]**20**	40% MeCN	4.7
[^{18}F]**21**	20% MeCN	3.6
[^{18}F]**22**	20% MeCN	3.2
[^{18}F]**23**	10% MeCN	4.5
[^{18}F]**24**	10% MeCN	3.9
[^{18}F]**25**	30% MeCN	3.2
[^{18}F]**26**	50% MeCN	4.4

HPLC conditions for radiolabeled tracer

[^{18}F]7: Column: Synergi Hydro-RP, 4 µm, 4.6×250 mm (Phenomenex, Aschaffenburg, Germany); eluent: 60% MeCN (0.1% TFA); flow rate: 1.5 mL/min; injection loop: 20 µL. t_R = 3.1 min.

[^{18}F]ALX5407: Column: Synergi Hydro-RP, 4 µm, 4.6×250 mm (Phenomenex, Aschaffenburg, Germany); eluent: 45% MeCN (0.1% TFA); flow rate: 1.5 mL/min; injection loop: 20 µL. t_R = 8.3 min.

[^{18}F]MNI1126: Column: Chromolith SpeedROD®, 4.6×50 mm (Merck Millipore, Darmstadt, Germany); eluent: 25% MeCN; flow rate: 1.5 mL/min; injection loop: 20 µL. t_R = 5.6 min.

(*S*,*S*)-**Ni-BPB-3-[^{18}F]FPhe**: Column: Synergi Hydro-RP, 4 µm, 4.6×250 mm (Phenomenex, Aschaffenburg, Germany); eluent: 75% MeCN; flow rate: 1.5 mL/min; injection loop: 20 µL. t_R = 5.0 min.

(*R*,*R*)-**Ni-BPB-3-[^{18}F]FPhe**: Column: Synergi Hydro-RP, 4 µm, 4.6×250 mm (Phenomenex, Aschaffenburg, Germany); eluent: 75% MeCN; flow rate: 1.5 mL/min; injection loop: 20 µL. t_R = 5.0 min.

(*S*,*S*)-**Ni-BPA-αMe-[^{18}F]FPhe**: Column: Synergi Hydro-RP, 4 µm, 4.6×250 mm (Phenomenex, Aschaffenburg, Germany); eluent: 75% MeCN; flow rate: 1.5 mL/min; injection loop: 20 µL. t_R = 3.8 min.

6-[^{18}F]FDOPA: Column: Synergi Hydro-RP, 4 µm, 4.6×250 mm (Phenomenex, Aschaffenburg, Germany); eluent: 60% MeCN; flow rate: 1.5 mL/min; injection loop: 20 µL. t_R = 10.9 min.

<u>HPLC conditions for quality control</u>

[^{18}F]R91150: Column: Synergi Hydro-RP, 4 µm, 4.6×250 mm (Phenomenex, Aschaffenburg, Germany); eluent: 30% MeCN (0.1% TFA); flow rate: 1.0 mL/min; injection loop: 20 µL. t_R = 6.4 min

[^{18}F]ALX5407: Column, Kinetex EVO C18, 5 µm, 4.6×250 mm (Phenomenex, Aschaffenburg, Germany); eluent, 35% MeCN (0.1% TFA); flow rate, 1.5 mL/min; injection loop: 20 µL. t_R = 12.0 min.

3-(*S*)-[^{18}F]FPhe: Column: Synergi Hydro-RP, 4 µm, 4.6×100 mm (Phenomenex, Aschaffenburg, Germany); eluent: 5% EtOH (0.1% H_3PO_4); flow rate: 1.0 mL/min; injection loop: 20 µL. t_R = 9.8 min.

3-(*R*)-[^{18}F]FPhe: Column: Synergi Hydro-RP, 4 µm, 4.6×100 mm (Phenomenex, Aschaffenburg, Germany); eluent: 10% EtOH (0.1% H_3PO_4); flow rate: 1.0 mL/min; injection loop: 20 µL. t_R = 7.8 min.

6-[^{18}F]FDOPA: Column: Synergi Hydro-RP, 4 µm, 4.6×250 mm (Phenomenex, Aschaffenburg, Germany); eluent: 1% EtOH (0.1% H_3PO_4); flow rate: 1.0 mL/min; injection loop: 20 µL. t_R = 7.1 min

3.3 Preparative HPLC

The HPLC system used for purification of crude PET-tracers comprised a Merck Hitachi L-6000 pump, a Knauer K-2500 detector, a Rheodyne 6-way valve and a Geiger-Müller counter.

[^{18}F]R91150: Column: Hydro-RP, 10 µm, 10×250 mm (Phenomenex, Aschaffenburg); eluent: 30% MeCN (0.1% TFA); flow rate: 7.1 mL/min; t_R= 7.5–9.0 min.

[^{18}F]ALX5407: Column: Gemini C18 110A, 5 µm, 10×250 mm (Phenomenex, Aschaffenburg, Germany); eluent: 35% MeCN (0.1% AcOH); flow rate: 7.4 mL/min. t_R = 20.5–22.5 min.

3-(*S*)-[^{18}F]FPhe: Column: Hydro-RP, 10 µm, 10×250 mm (Phenomenex, Aschaffenburg); eluent: 5% EtOH (0.1% H_3PO_4); flow rate: 7.4 mL/min; t_R= 8.0–9.2 min.

3-(*R*)-[^{18}F]FPhe: The same as for 3-(*S*)-[^{18}F]FPhe.

3-(*S*)-αMe-[^{18}F]FPhe: Column: Hydro-RP, 10 µm, 10×250 mm (Phenomenex, Aschaffenburg); eluent: 5% EtOH (0.1% H_3PO_4); flow rate: 7.4 mL/min. t_R = 17.1 min.

6-[^{18}F]FDOPA: Column: Hydro-RP, 10 µm, 10×250 mm (Phenomenex, Aschaffenburg); eluent, 1% EtOH (0.1% H_3PO_4); flow rate: 7.1 mL/min. t_R = 9–11 min.

3.4 Processing of fluoride-18

Aqueous [^{18}F]F^- was loaded onto a QMA cartridge (preconditioned with 1 mL H_2O) from the female to the male side. The cartridge was washed (from the male side) with anhydrous MeOH (1 mL) to remove residual H_2O and dried (from the female side) with air (2×10 mL). [^{18}F]F^- was eluted (from the female to the male side) with a solution of Et_4NOTf in *n*BuOH or MeOH.

3.5 Statistical analysis

All statistical analyses were performed with GraphPad Prism 8.4.3 for macOS, using one-way and two-way ANOVAs followed by appropriate Dunnett's, Tukey's or Sidak's multiple comparison tests. Statistical significance was defined as a *p*-value of less than 0.05.

3.6 General procedures for radiolabeling

"Alcohol enhanced" Cu-mediated radiofluorination of boranyl precursors – General Procedure 5 (GP5)

$[^{18}F]F^{-}$ (500 µL, 20–5000 MBq) was loaded onto a QMA cartridge and eluted with a solution of Et_4NOTf (1 mg, 3.6 µmol) in *n*BuOH (400 µL) into a solution of the respective Cu complex and precursor (10 µmol of each if not otherwise noted) in the corresponding solvent (800 µL). The reaction mixture was heated at 110 °C for 10 min under atmospheric or synthetic air, cooled to ambient temperature and diluted with H_2O (2 mL). RCCs were determined by radio-HPLC as described above. At high atmospheric humidity (e.g., during the midsummer), a significant drop of the RCCs was sometimes observed, presumably owing to the hygroscopicity of Et_4NOTf. In such cases $[^{18}F]F^{-}$ should preferably be eluted with a solution of Et_4NOTf in MeOH, as described above. MeOH was removed at 60 °C for 2–3 min under reduced pressure in a stream of argon and the residue was taken up into a solution of the respective Cu complex and precursor (10 µmol of each if not otherwise noted) in a mixture of the corresponding solvent and *n*BuOH (1200 µL; 2:1).

"Alcohol enhanced" Cu-mediated radiofluorination of stannyl precursors – General Procedure 6 (GP6)

Air conditions (GP6-A): $[^{18}F]F^{-}$ was loaded onto a QMA carbonate cartridge and eluted with Et_4NOTf (1 mg, 3.6 µmol) in *n*BuOH (400 µL) as described above directly into a solution of the corresponding stannyl precursor and copper(II) complex (10 µmol of each if not otherwise noted) in DMI (800 µL). The reaction mixture was heated at 110 °C for 10 min. RCCs were determined by radio-HPLC as described above.

Argon conditions (GP6-B): $[^{18}F]F^{-}$ was loaded onto a QMA carbonate cartridge. The V-Vial was evacuated using an oil pump, filled with argon and sealed with a silicone septum. Thereafter, $[^{18}F]F^{-}$ was eluted with Et_4NOTf (1 mg. 3.6 µmol) in *n*BuOH (400 µL) as described above with a cannula through the septum into the V-Vial, the cartridge was purged with argon and a solution of the corresponding precursor and copper(II) complex (10 µmol of each if not

otherwise noted) in DMI (800 µL) was added via a cannula. The reaction mixture was heated at 110 °C for 10 min. RCCs were determined by radio-HPLC as described above.

Cu-mediated radiofluorination of stannyl precursors in pure DMI – General Procedure 7 (GP7)

Air conditions (GP7-A): $[^{18}F]F^{-}$ was loaded onto a QMA carbonate cartridge and eluted with Et_4NOTf (1 mg, 3.6 µmol) in MeOH (500 µL) as described above. MeOH was evaporated at 60 °C for 2–3 min under reduced pressure in a stream of argon. The V-Vial was opened and a solution of the respective precursor and copper(II) complex (10 µmol of each if not otherwise noted) in DMI (800 µL) was added. The reaction mixture was heated at 110 °C for 10 min. RCCs were determined by radio-HPLC as described above.

Argon conditions (GP7-B): $[^{18}F]F^{-}$ was loaded onto a QMA carbonate cartridge and eluted with Et_4NOTf (1 mg, 3.6 µmol) in MeOH (500 µL) as described above, followed by evaporation of MeOH at 60 °C under reduced pressure in a stream of argon. The V-Vial was filled with argon, sealed with a silicon septum and a solution of the appropriate precursor and copper(II) complex (10 µmol of each if not otherwise noted) in DMI (800 µL) was added via a cannula through the septum. The reaction mixture was heated at 90 or 110 °C for 10 min. RCCs were determined by radio HPLC as described above.

3.7 Optimization studies

Elution efficiency

The dependency of [^{18}F]F^{-} elution efficiency and RCC on the amount of Et_4NOTf used was assessed according to GP5 with **1** and $Cu(Py)_4(OTf)_2$ in *n*BuOH/DMA (n = 3). RCCs were determined by radio-HPLC as described above.

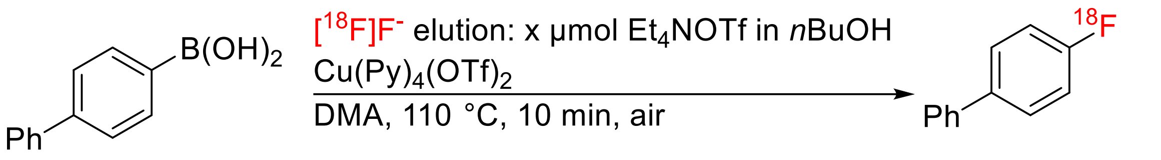

1 4-[^{18}F]F-Ph-Ph

Table S16: Dependency of [^{18}F]F^{-} recovery and RCCs on the amount of Et_4NOTf.

Entry	m [mg]	x [μmol]	Elution Efficiency [%]	RCC [%]
1	7.7	28	98 ± 0.4	28 ± 3
2	3.5	13	97 ± 0.3	35 ± 3
3	1.0	3.6	95 ± 1	38 ± 2
4	0.1	0.36	53 ± 7	19 ± 1

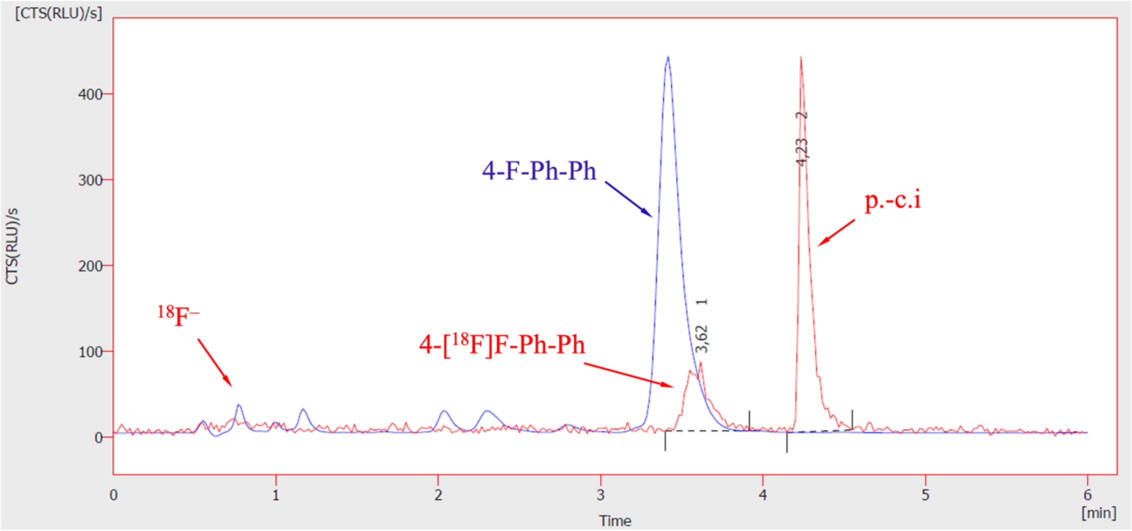

Result Table (Uncal - Data\alc-enhanced_393_19_01_2022_[1 DMA]_biphenyl_b(OH)2 - HERM)

	Reten. Time [min]	Area [CTS(RLU)/s.s]	Height [CTS(RLU)/s]	Area [%]
1	3,617	799,000	81,000	27,4
2	4,233	2117,000	438,375	72,6
	Total	2916,000	519,375	100,0

Figure S5: HPLC traces of crude 4-[^{18}F]F-Ph-Ph (spiked with 4-F-Ph-Ph) prepared in *n*BuOH/DMA from **1** using $Cu(Py)_4(OTf)_2$ as mediator and 1 mg Et_4NOTf for the elution of [^{18}F]F^{-}. Blue trace: UV, λ = 254 nm; red trace: radioactivity. Abbreviation: p.-c.i – post-column injection.

Screening of nickel(II), cobalt(II) and copper(II) complexes

The screening of nickel(II), cobalt(II) and copper(II) complexes was performed according to GP5 with aryl boronates **1 – 4** and stannanes **5** and **6** in *n*BuOH/DMA (n=3). RCCs were determined by radio-HPLC as described above. Representative radio-HPLC chromatograms of radiolabeled products are shown in Figure **S6–S12** and Figure **S14–S18**.

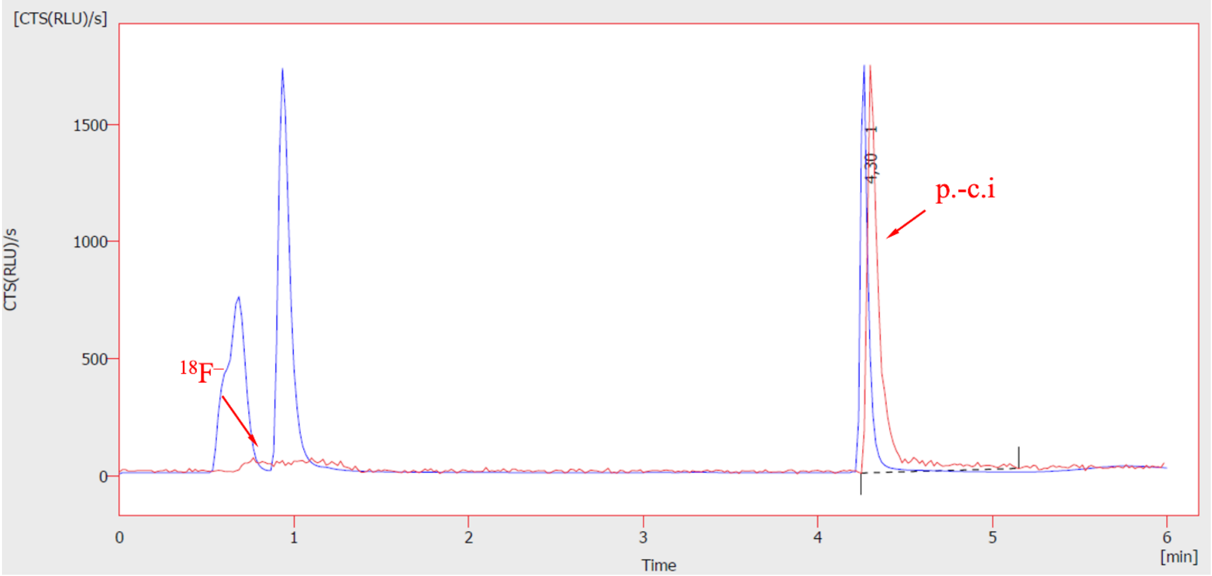

Result Table (Uncal - Data\alc-enhanced_855_01_08_2022_[31 DMA]biphenyl-B(OH)2 - HERM)

	Reten. Time [min]	Area [CTS(RLU)/s.s]	Height [CTS(RLU)/s]	Area [%]
1	4,300	8598,000	1741,944	100,0
	Total	8598,000	1741,944	100,0

Figure S6: HPLC traces of radiolabeling of **1** in *n*BuOH/DMA using $Ni(Py)_4(OTf)_2 \cdot H_2O$ as mediator and 1 mg Et_4NOTf for the elution of [^{18}F]F$^-$. Blue trace: UV, λ = 254 nm; red trace: radioactivity. Abbreviation: p.-c.i – post-column injection.

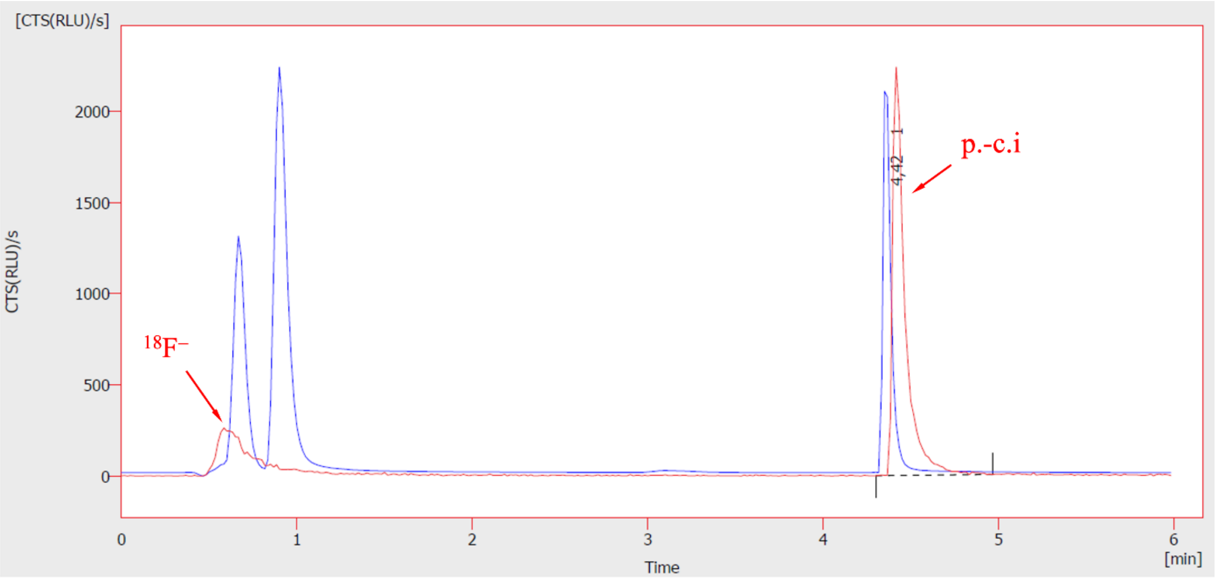

Result Table (Uncal - Data|alc-enhanced_916_20_09_2022_[32 DMA]biphenyl-B(OH)2 - HERM)

	Reten. Time [min]	Area [CTS(RLU)/s.s]	Height [CTS(RLU)/s]	Area [%]
1	4,417	11166,000	2239,775	100,0
	Total	11166,000	2239,775	100,0

Figure S7: HPLC traces of radiolabeling of **1** in *n*BuOH/DMA using $Co(Py)_4(ClO_4)_2{\cdot}3H_2O$ as mediator and 1 mg Et_4NOTf for the elution of $[^{18}F]F^{-}$. Blue trace: UV, λ = 254 nm; red trace: radioactivity. Abbreviation: p.-c.i – post-column injection.

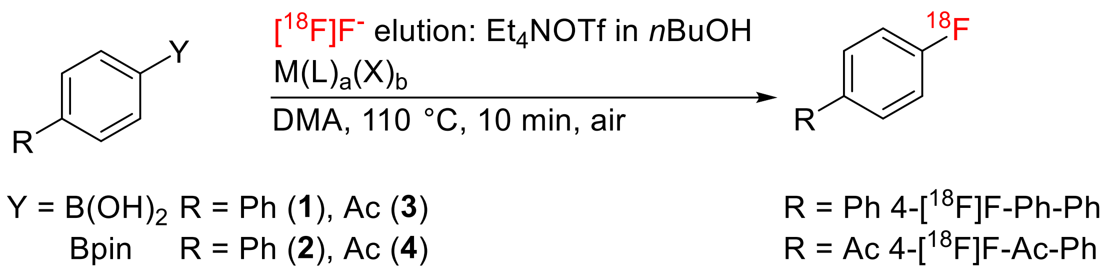

Table S17: Dependency of RCCs in *n*BuOH/DMA on the applied nickel, cobalt or copper mediator.

				RCC [%]			
**Entry**	**M**	**L**	**X**	**1**	**2**	**3**	**4**
1	Ni	Py	OTf	0	–	–	–
2	Co	Py	ClO_4	0	–	–	–
3	Cu	Py	OTf	30 ± 6	21 ± 8	15 ± 2	8 ± 2
4			ClO_4	34 ± 4	22 ± 4	17 ± 1	11 ± 3
5			ClO_3	13 ± 5	–	–	–
6			OTs	4 ± 1	3 ± 2	2 ± 0.4	–
7			OMs	0	–	–	–
8			Cl	0	–	–	–
9			SO_4	0	–	–	–
10			OAc	0	–	–	–
11			Br	0	–	–	–
12		2-MeOPy	OTf	2 ± 1	1 ± 0.4	0	0
13			ClO_4	4 ± 1	3 ± 1	0	0
14		3-MeOPy	OTf	20 ± 4	10 ± 2	5 ± 2	3 ± 1
15			ClO_4	30 ± 2	27 ± 5	13 ± 4	7 ± 1
16		4-MeOPy	OTf	46 ± 3	50 ± 4	27 ± 9	16 ± 3
17			ClO_4	36 ± 3	30 ± 6	34 ± 2	29 ± 5
18		2,4-$(MeO)_2$Py	OTf	0	0	3 ± 1	2 ± 1
19			ClO_4	0	0	3 ± 1	2 ± 1
20		4-PhPy	OTf	29 ± 2	21 ± 2	12 ± 1	7 ± 1
21			ClO_4	39 ± 10	35 ± 4	30 ± 4	19 ± 1
22		3,4-Me_2Py	OTf	58 ± 8	52 ± 7	26 ± 1	18 ± 2
23			ClO_4	26 ± 7	54 ± 4	19 ± 5	26 ± 1
24		4-F_3Cpy	OTf	2 ± 2	1 ± 0.2	0	0
25		Pz	OTf	6 ± 2	5 ± 0.3	0	0
26		4,4'-BiPy	OTf	1 ± 1	3 ± 0.4	0	0
27			ClO_4	14 ± 2	13 ± 4	6 ± 2	3 ± 1
28		Triaz	OTf	10 ± 2	9 ± 1	6 ± 1	2 ± 0.3
29		Pyr	OTf	4 ± 1	2 ± 0.3	0	1 ± 1
30		Quin	OTf	8 ± 3	2 ± 1	0	0

Entry	M	L	X	RCC [%] 1	2	3	4
31			ClO_4	3 ± 1	5 ± 1	0	1 ± 1
32		Isoq	OTf	29 ± 5	19 ± 5	12 ± 2	5 ± 2
33			ClO_4	48 ± 3	26 ± 8	16 ± 3	15 ± 3
34		Impdz	OTf	17 ± 3	13 ± 1	5 ± 1	7 ± 1
35			ClO_4	30 ± 5	39 ± 5	44 ± 5	26 ± 4
36			ClO_3	13 ± 6	–	–	–
37			OMs	0	0	0	0
38			OTs	36 ± 5	5 ± 3	1 ± 1	4 ± 1

Conditions: i) elution of $[^{18}F]F^-$ (10–50 MBq) with Et_4NOTf (1 mg, 4 µmol) in *n*BuOH (400 µL) into a solution of substrates **1**–**4** (10 µmol, 1 eq.) and $M(L)_a(X)_b$ (10 µmol, 1 eq.) in DMA (800 µL); ii) 110 °C for 10 min under air; iii) addition of H_2O (1 mL). Radiochemical conversions (RCCs) were determined by HPLC as described in the supporting information and are provided in the following format: mean RCC ± standard deviation (%). All experiments were carried out at least in triplicate. Py – pyridine, 2-MeOPy – 2-methoxypyridine, 3-MeOPy – 3-methoxypyridine, 4-MeOPy – 4-methoxypyridine, 2,4-$(MeO)_2Py$ – 2,4-dimethoxypyridine, 4,4'-BiPy – 4,4'-bipyridine, 4-PhPy – 4-phenylpyridine, 3,4-Me_2Py – 3,4-lutidine, 4-F_3CPy – 4-trifluoromethylpyridine, Pz – pyrazine, Triaz – *N*-methyl-1,2,4-triazole, Pyr – *N*-methylpyrazole, Quin – quinoline, Isoq – isoquinoline, Impdz – imidazo(1,2-b)pyridazine.

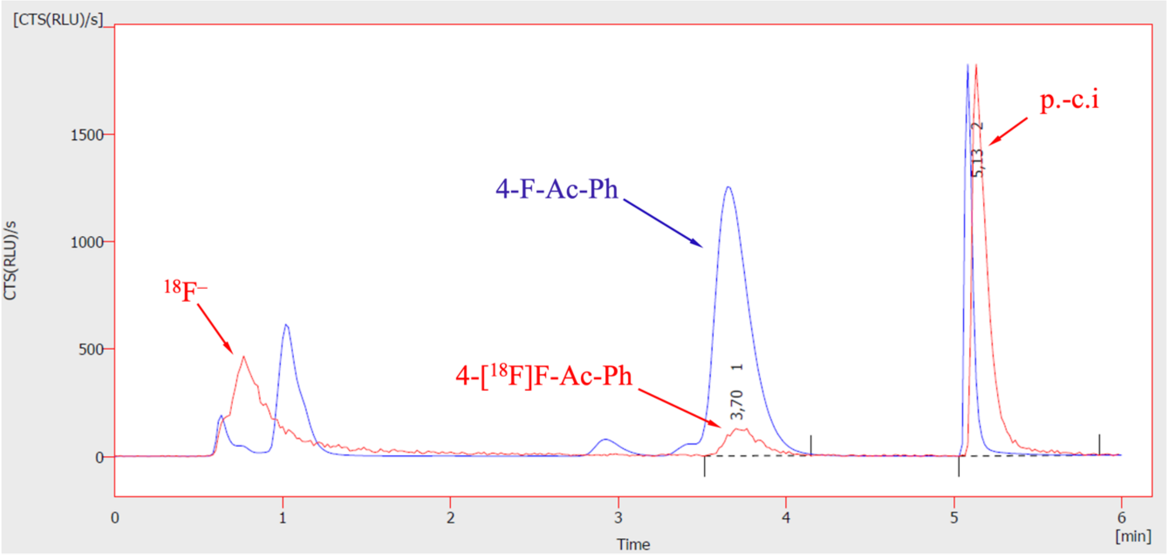

Result Table (Uncal - Data|alc-enhanced_824_28_06_2022_[1 DMA]4-acetylphenyl_B(OH)2_spike - HERM)

	Reten. Time [min]	Area [CTS(RLU)/s.s]	Height [CTS(RLU)/s]	Area [%]
1	3,700	1816,000	126,711	13,3
2	5,133	11853,000	1823,520	86,7
	Total	13669,000	1950,231	100,0

Figure S8: HPLC traces of crude 4-[^{18}F]F-Ac-Ph (spiked with 4-F-Ac-Ph) prepared in *n*BuOH/DMA from **3** using $Cu(Py)_4(OTf)_2$ as mediator. Blue trace: UV, λ = 254 nm; red trace: radioactivity. Abbreviation: p.-c.i – post-column injection.

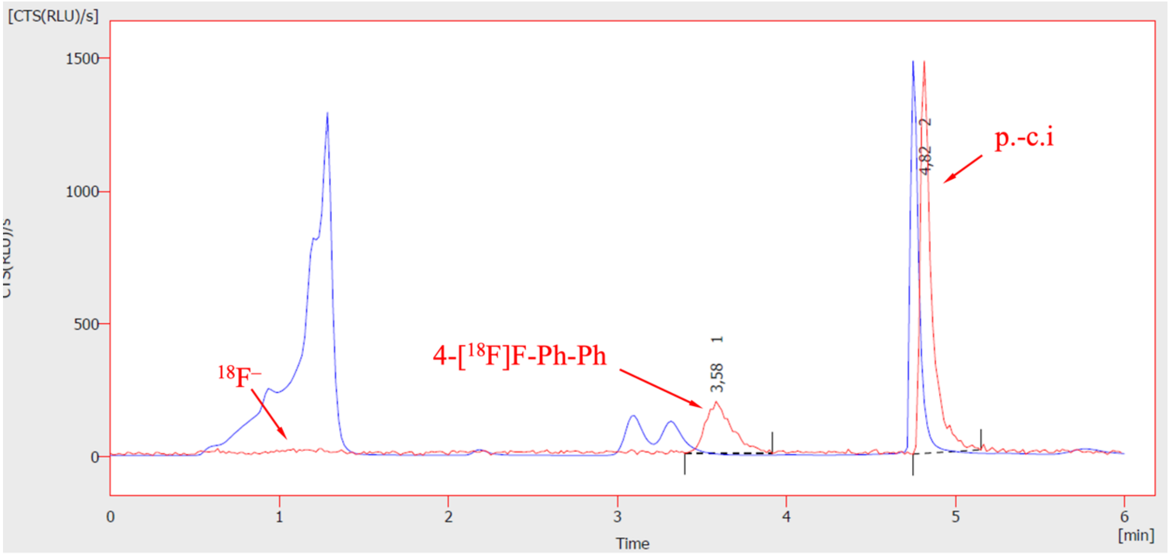

Result Table (Uncal - Data\alc-enhanced_400_19_01_2022_[3 DMA]_biphenyl_b(OH)2 - HERM)

	Reten. Time [min]	Area [CTS(RLU)/s.s]	Height [CTS(RLU)/s]	Area [%]
1	3,583	2122,000	196,000	23,5
2	4,817	6916,000	1478,333	76,5
	Total	9038,000	1674,333	100,0

Figure S9: HPLC traces of crude 4-[^{18}F]F-Ph-Ph prepared in *n*BuOH/DMA from **1** using Cu(4-PhPy)$_4$(OTf)$_2$ as mediator. Blue trace: UV, λ = 254 nm; red: radioactivity. Abbreviation: p.-c.i – post-column injection.

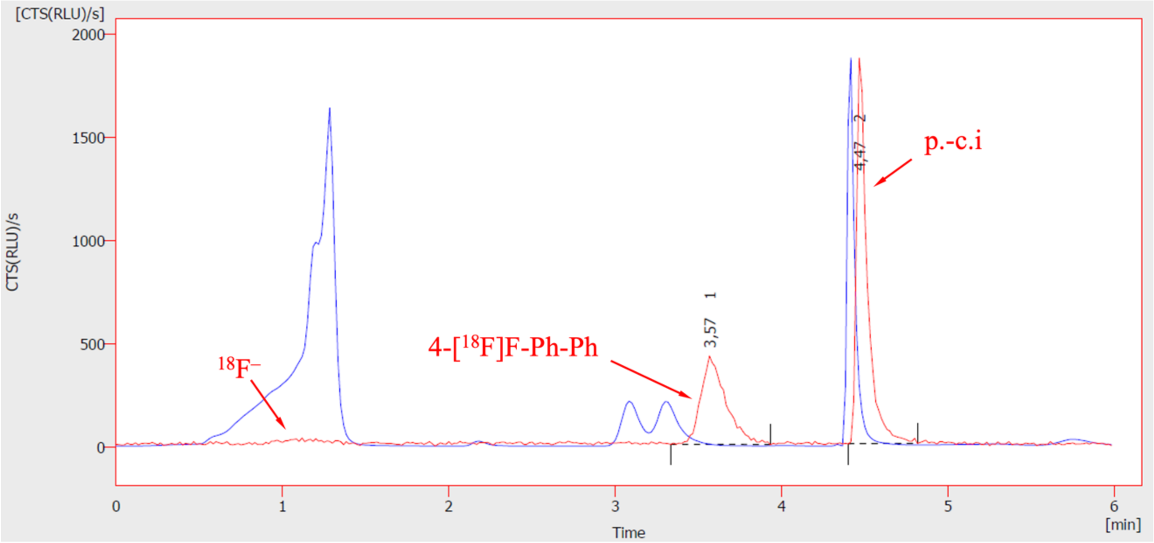

Result Table (Uncal - Data\alc-enhanced_403_19_01_2022_[4 DMA]_biphenyl_b(OH)2 - HERM)

	Reten. Time [min]	Area [CTS(RLU)/s.s]	Height [CTS(RLU)/s]	Area [%]
1	3,567	4500,000	428,389	34,6
2	4,467	8494,000	1866,680	65,4
	Total	12994,000	2295,069	100,0

Figure S10: HPLC traces of crude 4-[^{18}F]F-Ph-Ph prepared in *n*BuOH//DMA from **1** using Cu(4-PhPy)$_4$(ClO$_4$)$_2$ as mediator. Blue trace: UV, λ = 254 nm; red trace: radioactivity. Abbreviation: p.-c.i – post-column injection.

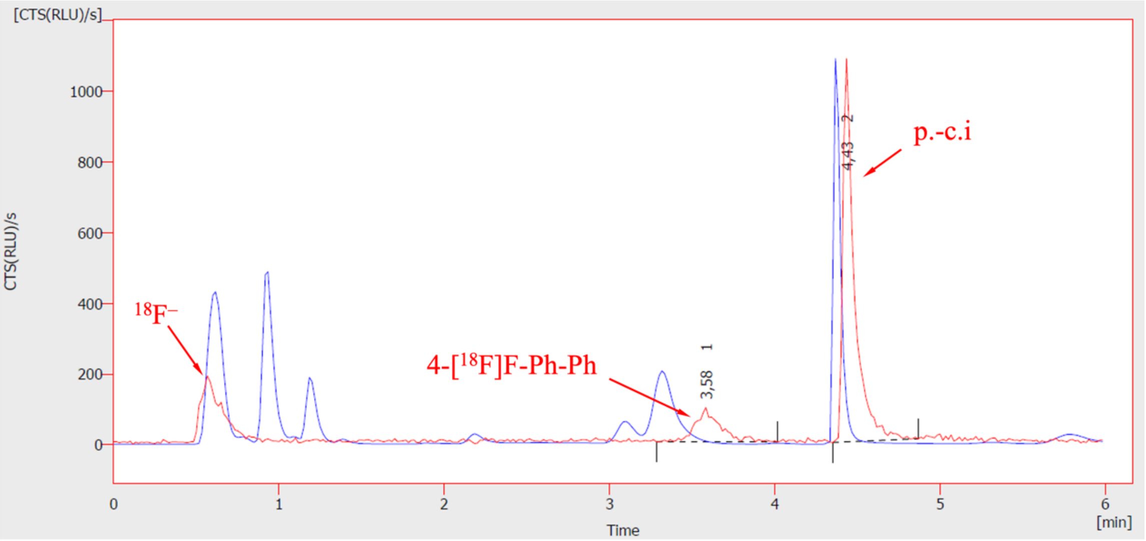

Result Table (Uncal - Data|alc-enhanced_405_19_01_2022_[17 DMA]_biphenyl_b(OH)2 - HERM)

	Reten. Time [min]	Area [CTS(RLU)/s.s]	Height [CTS(RLU)/s]	Area [%]
1	3,583	1027,000	97,409	16,2
2	4,433	5318,000	1085,387	83,8
	Total	6345,000	1182,796	100,0

Figure S11: HPLC traces of crude 4-[^{18}F]F-Ph-Ph prepared in *n*BuOH/DMA from **1** using Cu(Impdz)$_4$(OTf)$_2$ as mediator. Blue trace: UV, λ = 254 nm; red trace: radioactivity. Blue: UV chromatogram, λ = 254 nm; red: radio-chromatogram. Abbreviation: p.-c.i – post-column injection.

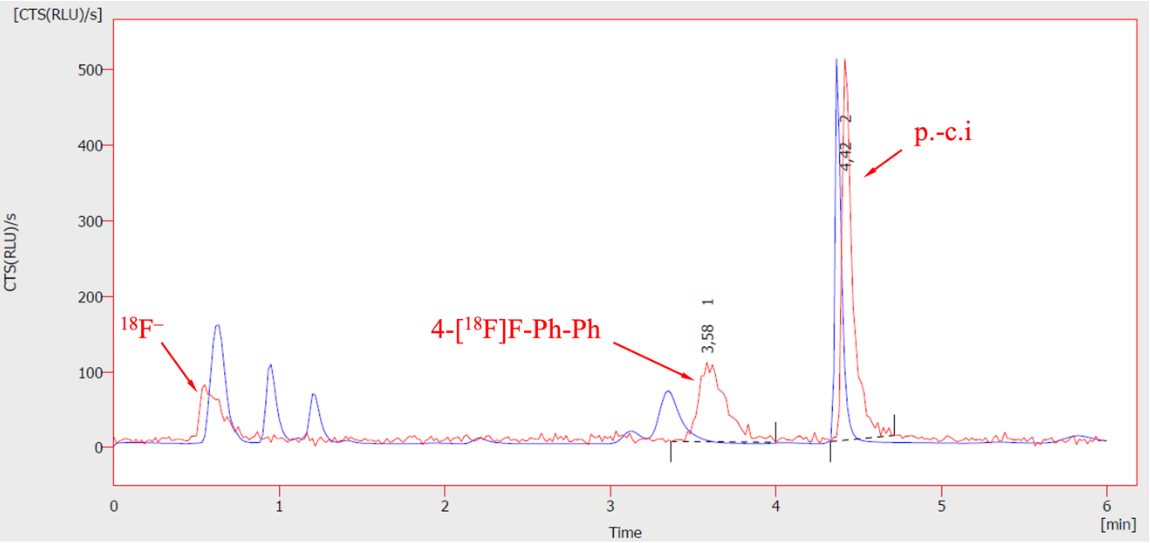

Result Table (Uncal - Data|alc-enhanced_410_19_01_2022_[18 DMA]_biphenyl_b(OH)2 - HERM)

	Reten. Time [min]	Area [CTS(RLU)/s.s]	Height [CTS(RLU)/s]	Area [%]
1	3,583	1225,000	105,342	34,9
2	4,417	2287,000	505,261	65,1
	Total	3512,000	610,603	100,0

Figure S12: HPLC traces of crude 4-[^{18}F]F-Ph-Ph prepared in *n*BuOH/DMA from **1** using Cu(Impdz)$_4$(ClO$_4$)$_2$ as mediator. Blue trace: UV, λ = 254 nm; red trace: radioactivity. Abbreviation: p.-c.i – post-column injection.

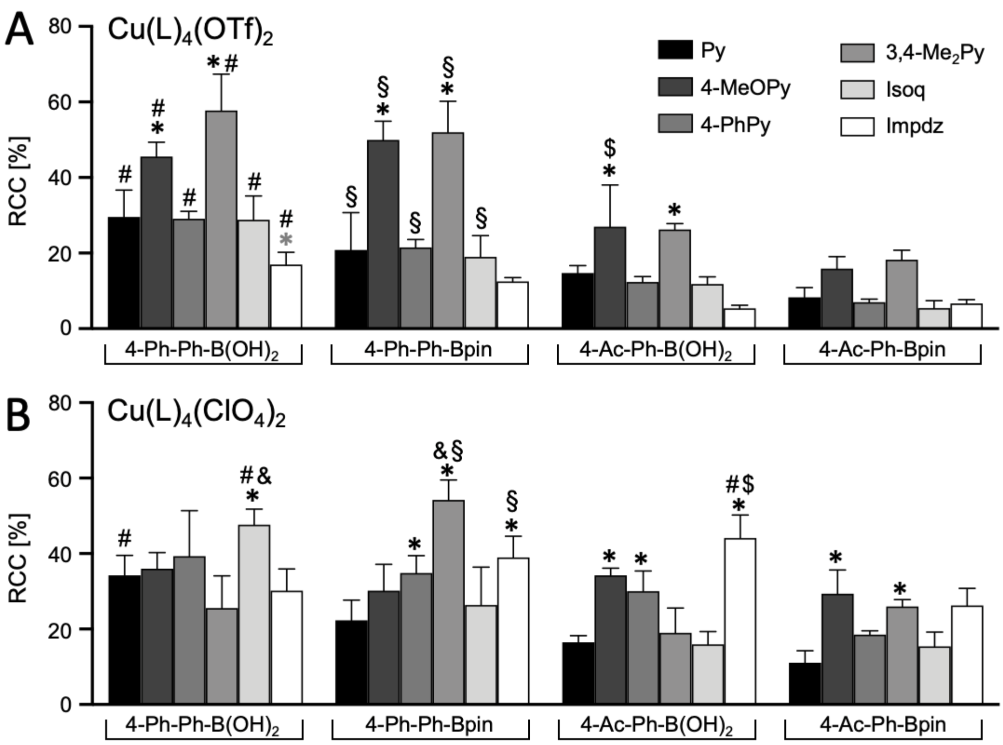

Figure S13: Dependency of RCCs on the precursor and applied Cu complex in *n*BuOH/DMA. Significant differences are indicated by the following symbols: *: $p<0.05$ for comparison of the different ligands to Py; &: $p<0.05$ for 4-Ph-Ph-B(OH)$_2$ vs. 4-Ph-Ph-Bpin; #: $p<0.05$ for 4-Ph-Ph-B(OH)$_2$ vs. 4-Ac-Ph-B(OH)$_2$; §: $p<0.05$ for 4-Ph-Ph-Bpin vs. 4-Ac-Ph-Bpin; $: $p<0.05$ for 4-Ac-Ph-B(OH)$_2$ vs. 4-Ac-Ph-Bpin (two-way ANOVA).

Statistical data corresponding to Figure S13:

Table S18: RCCs (mean values ± standard deviation in %) for radiosyntheses with different Cu mediators, Cu(L)$_4$(OTf)$_2$, in *n*BuOH/DMA. 2-way ANOVA: Main effect of factor "**ligand**": $F(5,48)=60.23$, $p<0.0001$. Dunnett's multiple comparisons test with Cu(Py)$_4$(OTf)$_2$ as control with n=3 per group.

	4-Ph-Ph-B(OH)$_2$	4-Ph-Ph-Bpin	4-Ac-Ph-B(OH)$_2$	4-Ac-Ph-Bpin
Py	29.55 ± 7.14	20.82 ± 9.91	14.75 ± 1.93	8.29 ± 2.55
4-MeOPy	45.53 ± 3.81<br>p=0.0014*	49.96 ± 4.93<br>p<0.0001*	27.01 ± 11.05<br>p=0.0187*	15.88 ± 3.17<br>p=0.2415
4-PhPy	29.09 ± 1.98<br>p=0.9999	21.48 ± 2.11<br>p=0.9997	12.37 ± 1.42<br>p=0.9669	7.00 ± 0.85<br>p=0.9979
3,4-Me$_2$Py	57.76 ± 9.63<br>p<0.0001*	51.96 ± 8.26<br>p<0.0001*	26.23 ± 1.57<br>p=0.0305*	18.24 ± 2.52<br>p=0.0744
Isoq	28.80 ± 6.35<br>p=0.9997	19.00 ± 5.63<br>p=0.9896	11.80 ± 1.91<br>p=0.9240	5.43 ± 1.96<br>p=0.9329
Impdz	16.98 ± 3.27<br>p=0.0153*	12.51 ± 1.02<br>p=0.1729	5.37 ± 0.80<br>p=0.1012	6.70 ± 0.96<br>p=0.9944

green: significantly higher RCCs compared to Cu(Py)$_4$(OTf)$_2$; red: significantly lower RCCs compared to Cu(Py)$_4$(OTf)$_2$.

Table S19: RCCs (mean values ± standard deviation in %) for radiosyntheses with different Cu mediators, $Cu(L)_4(OTf)_2$, in *n*BuOH/DMA. Main effect of factor "**precursor**": $F(3,48)=91.01$, $p<0.0001$. Tukey's multiple comparisons test, n=3 per group.

	4-Ph-Ph-$B(OH)_2$ vs. 4-Ph-Ph-Bpin	4-Ph-Ph-$B(OH)_2$ vs. 4-Ac-Ph-$B(OH)_2$	4-Ph-Ph-Bpin vs. 4-Ac-Ph-Bpin	4-Ac-Ph-$B(OH)_2$ vs. 4-Ac-Ph-Bpin
Py	29.55 ± 7.14<br>20.82 ± 9.91<br>p=0.1582	29.55 ± 7.14<br>14.75 ± 1.93<br>p=0.0039[#]	20.82 ± 9.91<br>8.29 ± 2.55<br>p=0.0183[§]	14.75 ± 1.93<br>8.29 ± 2.55<br>p=0.4007
4-MeOPy	45.53 ± 3.81<br>49.96 ± 4.93<br>p=0.7029	45.53 ± 3.81<br>27.01 ± 11.05<br>p=0.0002[#]	49.96 ± 4.93<br>15.88 ± 3.17<br>p<0.0001[§]	27.01 ± 11.05<br>15.88 ± 3.17<br>p=0.0438[$]
4-PhPy	29.09 ± 1.98<br>21.48 ± 2.11<br>p=0.2594	29.09 ± 1.98<br>12.37 ± 1.42<br>p=0.0009[#]	21.48 ± 2.11<br>7.00 ± 0.85<br>p=0.0049[§]	12.37 ± 1.42<br>7.00 ± 0.85<br>p=0.5612
3,4-Me_2Py	57.76 ± 9.63<br>51.96 ± 8.26<br>p=0.4959	57.76 ± 9.63<br>26.23 ± 1.57<br>p<0.0001[#]	51.96 ± 8.26<br>18.24 ± 2.52<br>p<0.0001[§]	26.23 ± 1.57<br>18.24 ± 2.52<br>p=0.2209
Isoq	28.80 ± 6.35<br>19.00 ± 5.63<br>p=0.0922	28.80 ± 6.35<br>11.80 ± 1.91<br>p=0.0008[#]	19.00 ± 5.63<br>5.43 ± 1.96<br>p=0.0093[§]	11.80 ± 1.91<br>5.43 ± 1.96<br>p=0.4141
Impdz	16.98 ± 3.27<br>12.51 ± 1.02<br>p=0.6956	16.98 ± 3.27<br>5.37 ± 0.80<br>p=0.0327[#]	12.51 ± 1.02<br>6.70 ± 0.96<br>p=0.4949	5.37 ± 0.80<br>6.70 ± 0.96<br>p=0.9879

red: significantly lower RCCs for Bpin compared to $B(OH)_2$ precursors; green: significantly higher RCCs for 4-Ph-Ph compared to 4-Ac-Ph precursors.

Table S20: RCCs (mean values ± standard deviation in %) for radiosyntheses with different Cu mediators, $Cu(L)_4(ClO_4)_2$, in *n*BuOH/DMA. 2-way ANOVA: Main effect of factor "**ligand**": $F(5,48)=9.02$, $p<0.0001$. Dunnett's multiple comparisons test with $Cu(Py)_4(ClO_4)_2$ as control with n=3 per group.

	4-Ph-Ph-B(OH)$_2$	4-Ph-Ph-Bpin	4-Ac-Ph-B(OH)$_2$	4-Ac-Ph-Bpin
Py	34.21 ± 5.32	22.33 ± 5.31	16.51 ± 1.73	11.08 ± 3.17
4-MeOPy	36.03 ± 4.24<br>p=0.9944	30.17 ± 6.96<br>p=0.3291	34.23 ± 1.92<br>p=0.0020*	29.33 ± 6.35<br>p=0.0014*
4-PhPy	39.32 ± 12.07<br>p=0.7135	34.88 ± 4.59<br>p=0.0414*	30.04 ± 5.39<br>p=0.0244*	18.55 ± 1.09<br>p=0.3736
3,4-Me$_2$Py	25.57 ± 8.53<br>p=0.2446	54.26 ± 5.24<br>p<0.0001*	19.00 ± 6.56<br>p=0.9772	25.97 ± 1.82<br>p=0.0114*
Isoq	47.62 ± 4.19<br>p=0.0262*	26.37 ± 10.07<br>p=0.8575	15.97 ± 3.35<br>p=0.9999	15.43 ± 3.77<br>p=0.8187
Impdz	30.21 ± 5.73<br>p=0.8613	39.00 ± 5.60<br>p=0.0039*	44.15 ± 6.10<br>p<0.0001*	26.24 ± 4.58<br>p=0.0097*

green: significantly higher RCCs compared to $Cu(Py)_4(ClO_4)_2$; red: significantly lower RCC compared to $Cu(Py)_4(ClO_4)_2$.

Table S21: RCCs (mean values ± standard deviation in %) for radiosyntheses with different Cu mediators, $Cu(L)_4(ClO_4)_2$, in *n*BuOH/DMA. 2-way ANOVA: Main effect of factor "**precursor**": $F(3,48)=25.45$, $p<0.0001$. Tukey's multiple comparisons test, n=3 per group.

	4-Ph-Ph-B(OH)$_2$ vs. 4-Ph-Ph-Bpin	4-Ph-Ph-B(OH)$_2$ vs. 4-Ac-Ph-B(OH)$_2$	4-Ph-Ph-Bpin vs. 4-Ac-Ph-Bpin	4-Ac-Ph-B(OH)$_2$ vs. 4-Ac-Ph-Bpin
Py	34.21 ± 5.32<br>22.33 ± 5.31<br>p=0.0669	34.21 ± 5.32<br>16.51 ± 1.73<br>p=0.0024#	22.33 ± 5.31<br>11.08 ± 3.17<br>p=0.0904	16.51 ± 1.73<br>11.08 ± 3.17<br>p=0.6556
4-MeOPy	36.03 ± 4.24<br>30.17 ± 6.96<br>p=0.5972	36.03 ± 4.24<br>34.23 ± 1.92<br>p=0.9805	30.17 ± 6.96<br>29.33 ± 6.35<br>p=0.9980	34.23 ± 1.92<br>29.33 ± 6.35<br>p=0.7235
4-PhPy	39.32 ± 12.07<br>34.88 ± 4.59<br>p=0.7797	39.32 ± 12.07<br>30.04 ± 5.39<br>p=0.2094	34.88 ± 4.59<br>18.55 ± 1.09<br>p=0.0056§	30.04 ± 5.39<br>18.55 ± 1.09<br>p=0.0807
3,4-Me_2Py	25.57 ± 8.53<br>54.26 ± 5.24<br>p<0.0001&	25.57 ± 8.53<br>19.00 ± 6.56<br>p=0.5045	54.26 ± 5.24<br>25.97 ± 1.82<br>p<0.0001§	19.00 ± 6.56<br>25.97 ± 1.82<br>p=0.4531
Isoq	47.62 ± 4.19<br>26.37 ± 10.07<br>p=0.0002&	47.62 ± 4.19<br>15.97 ± 3.35<br>p<0.0001#	26.37 ± 10.07<br>15.43 ± 3.77<br>p=0.1046	15.97 ± 3.35<br>15.43 ± 3.77<br>p=0.9995
Impdz	30.21 ± 5.73<br>39.00 ± 5.60<br>p=0.2517	30.21 ± 5.73<br>44.15 ± 6.10<br>p=0.0229#	39.00 ± 5.60<br>26.24 ± 4.58<br>p=0.0430§	44.15 ± 6.10<br>26.24 ± 4.58<br>p=0.0021$

red: significantly lower RCCs for Bpin compared to B(OH)$_2$ precursors; blue: significantly higher RCCs for Bpin compared to B(OH)$_2$ precursors; green: significantly higher RCCs for 4-Ph-Ph compared to 4-Ac-Ph substituted precursors; pink: significantly lower RCCs for 4-Ph-Ph compared to 4-Ac-Ph precursors.

Statistical data corresponding to Figure 2 in the main article:

Table S22: RCCs (mean values ± standard deviation in %) for radiolabeling of 4-Ph-Ph-B(OH)$_2$ using different Cu(Py)$_4$X$_2$ complexes in *n*BuOH/DMA. 1-way ANOVA: Main effect of factor "**counter ion**": F(4,10)=29.30, p<0.0001. Tukey's multiple comparisons test, n=3 per group.

	RCC (%)	comparison	
Cu(Py)$_4$(OTf)$_2$	29.55 ± 7.14	vs. Cu(Py)$_4$(ClO$_4$)$_2$	p=0.7659
		vs. Cu(Py)$_4$(ClO$_3$)$_2$	p=0.0108*
		vs. Cu(Py)$_4$(OTs)$_2$	p=0.0006*
		vs. Cu(Py)$_4$(OMs)$_2$	p=0.0002*
Cu(Py)$_4$(ClO$_4$)$_2$	34.21 ± 5.32	vs. Cu(Py)$_4$(ClO$_3$)$_2$	p=0.0020*
		vs. Cu(Py)$_4$(OTs)$_2$	p=0.0001*
		vs. Cu(Py)$_4$(OMs)$_2$	p<0.0001*
Cu(Py)$_4$(ClO$_3$)$_2$	12.50 ± 6.05	vs. Cu(Py)$_4$(OTs)$_2$	p=0.2824
		vs. Cu(Py)$_4$(OMs)$_2$	p=0.0628
Cu(Py)$_4$(OTs)$_2$	4.06 ± 1.65	vs. Cu(Py)$_4$(OMs)$_2$	p=0.8402
Cu(Py)$_4$(OMs)$_2$	0.00 ± 0.00		

Table S23: RCCs (mean values ± standard deviation in %) for radiolabeling of 4-Ph-Ph-B(OH)$_2$ in *n*BuOH/DMA using different Cu(Impdz)$_4$X$_2$ complexes as mediators. 1-way ANOVA: Main effect of factor "**counter ion**": F(4,10)=23.64, p<0.0001. Tukey's multiple comparisons test, n=3 per group.

	RCC (%)	comparison	
Cu(Impdz)$_4$(OTf)$_2$	16.98 ± 3.27	vs. Cu(Impdz)$_4$(ClO$_4$)$_2$	p=0.0586
		vs. Cu(Impdz)$_4$(ClO$_3$)$_2$	p=0.8865
		vs. Cu(Impdz)$_4$(OTs)$_2$	p=0.0069*
		vs. Cu(Impdz)$_4$(OMs)$_2$	p=0.0145*
Cu(Impdz)$_4$(ClO$_4$)$_2$	30.21 ± 5.73	vs. Cu(Impdz)$_4$(ClO$_3$)$_2$	p=0.0144*
		vs. Cu(Impdz)$_4$(OTs)$_2$	p=0.6407
		vs. Cu(Impdz)$_4$(OMs)$_2$	p=0.0002*
Cu(Impdz)$_4$(ClO$_3$)$_2$	13.21 ± 7.21	vs. Cu(Impdz)$_4$(OTs)$_2$	p=0.0019*
		vs. Cu(Impdz)$_4$(OMs)$_2$	p=0.0590
Cu(Impdz)$_4$(OTs)$_2$	36.02 ± 5.80	vs. Cu(Impdz)$_4$(OMs)$_2$	p<0.0001*
Cu(Impdz)$_4$(OMs)$_2$	0.00 ± 0.00		

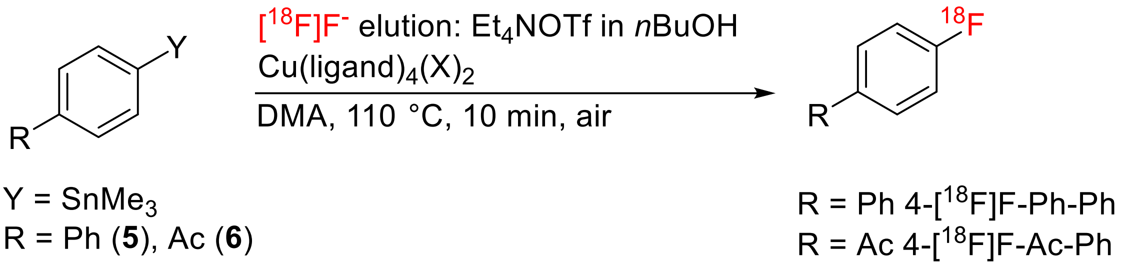

Table S24: Dependency of RCCs for radiolabeling of 4-Ph- and 4-Ac-Ph-$SnMe_3$ in *n*BuOH/DMA on the applied copper mediators.

			RCC [%]	
**Entry**	**Ligand**	**X**	**5**	**6**
1	Py	OTf	14 ± 2	2 ± 1
2		ClO_4	10 ± 2	3 ± 1
3	3-MeOPy	OTf	4 ± 1	5 ± 1
4		ClO_4	5 ± 1	7 ± 2
5	4-MeOPy	OTf	4 ± 2	13 ± 6
6		ClO_4	5 ± 2	12 ± 2
7	4-PhPy	OTf	9 ± 1	3 ± 0.3
8		ClO_4	14 ± 5	9 ± 1
9	3,4-Me_2Py	OTf	10 ± 4	11 ± 2
10		ClO_4	8 ± 3	5 ± 1
11	Isoq	OTf	7 ± 2	7 ± 1
12		ClO_4	20 ± 4	9 ± 2
13	Impdz	OTf	7 ± 3	13 ± 3
14		ClO_4	20 ± 4	23 ± 2

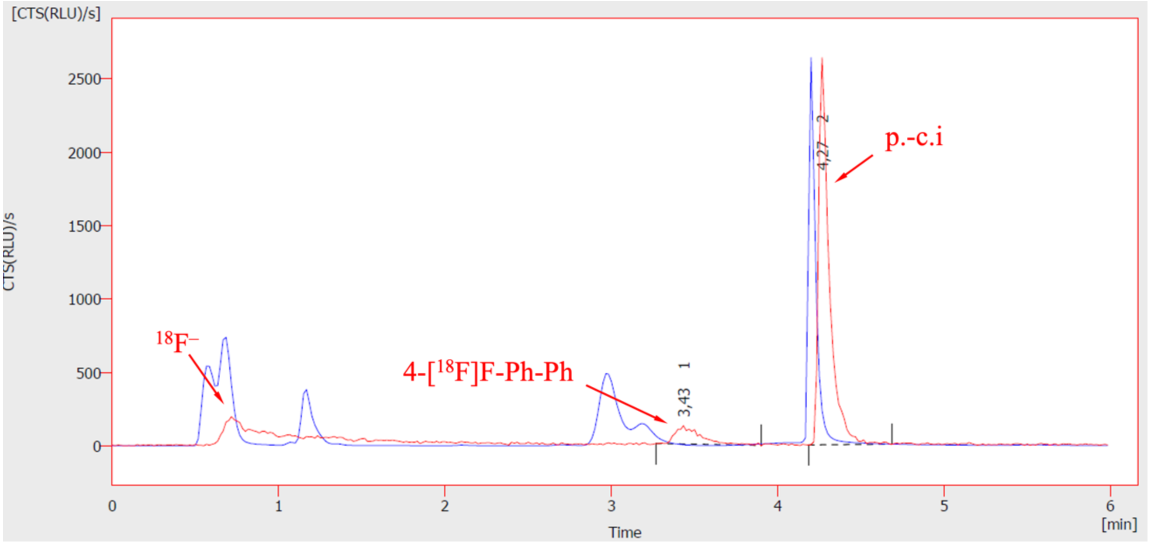

Result Table (Uncal - Data|alc-enhanced_637_29_03_2022_[1 DMA]biphenyl-SnMe3 - HERM)

	Reten. Time [min]	Area [CTS(RLU)/s.s]	Height [CTS(RLU)/s]	Area [%]
1	3,433	1339,000	125,421	10,2
2	4,267	11813,000	2636,000	89,8
	Total	13152,000	2761,421	100,0

Figure S14: HPLC traces of crude 4-[^{18}F]F-Ph-Ph prepared in *n*BuOH/DMA from **5** using $Cu(Py)_4(OTf)_2$ as mediator. Blue trace: UV, λ = 254 nm; red trace: radioactivity. Abbreviation: p.-c.i – post-column injection.

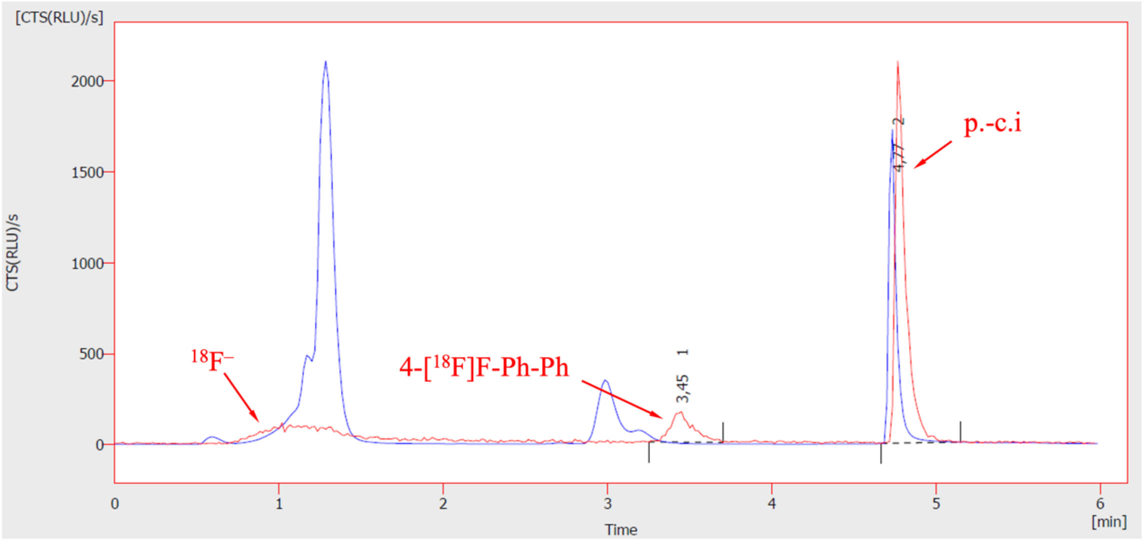

Result Table (Uncal - Data|alc-enhanced_640_29_03_2022_[4 DMA]biphenyl-SnMe3 - HERM)

	Reten. Time [min]	Area [CTS(RLU)/s.s]	Height [CTS(RLU)/s]	Area [%]
1	3,450	1599,000	166,889	14,6
2	4,767	9387,500	2104,552	85,4
	Total	10986,500	2271,441	100,0

Figure S15: HPLC traces of crude 4-[^{18}F]F-Ph-Ph prepared in *n*BuOH/DMA from **5** using $Cu(4\text{-}PhPy)_4(ClO_4)_2$ as mediator. Blue trace: UV, λ = 254 nm; red trace: radioactivity. Abbreviation: p.-c.i – post-column injection.

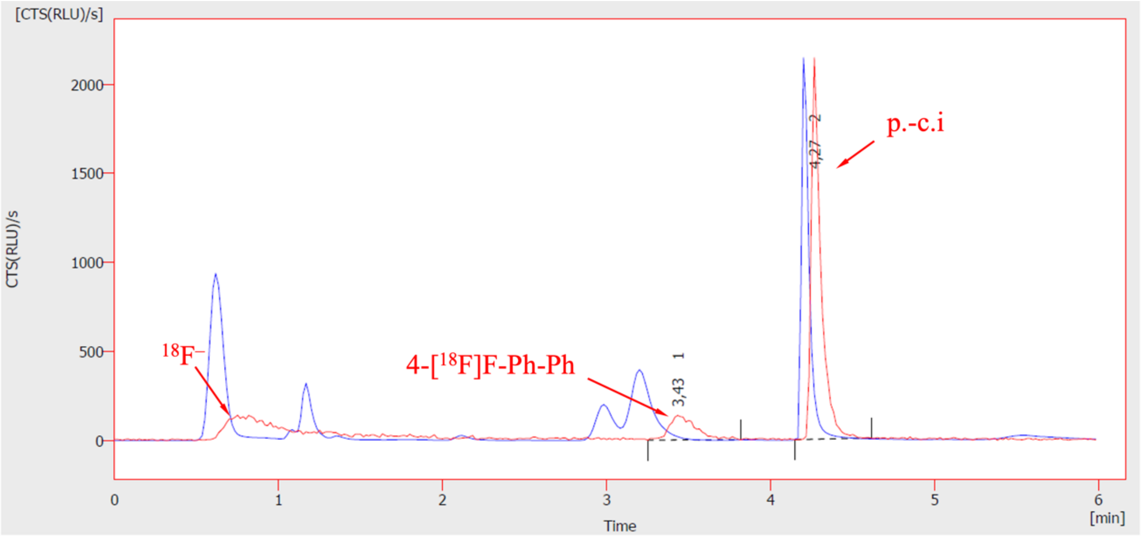

Result Table (Uncal - Data|alc-enhanced_642_29_03_2022_[18 DMA]biphenyl-SnMe3 - HERM)

	Reten. Time [min]	Area [CTS(RLU)/s.s]	Height [CTS(RLU)/s]	Area [%]
1	3,433	1614,000	139,029	15,5
2	4,267	8798,000	2141,500	84,5
	Total	10412,000	2280,529	100,0

Figure S16: HPLC traces of crude 4-[18F]F-Ph-Ph prepared in *n*BuOH/DMA from **5** using Cu(Impdz)$_4$(ClO$_4$) as mediator. Blue trace: UV, λ = 254 nm; red trace: radioactivity. Abbreviation: p.-c.i – post-column injection.

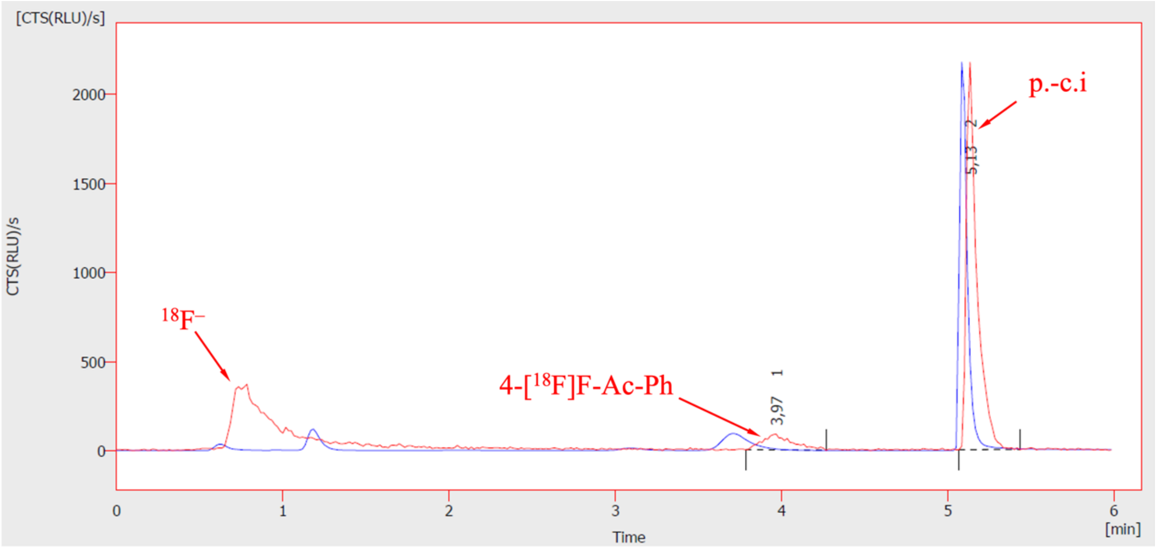

Result Table (Uncal - Data|alc-enhanced_644_29_03_2022_[4 DMA]4-acetylphenyl-SnMe3 - HERM)

	Reten. Time [min]	Area [CTS(RLU)/s.s]	Height [CTS(RLU)/s]	Area [%]
1	3,967	1098,500	89,138	10,1
2	5,133	9748,000	2175,636	89,9
	Total	10846,500	2264,774	100,0

Figure S17: HPLC traces of crude 4-[18F]F-Ac-Ph prepared in *n*BuOH/DMA from **6** using Cu(4-PhPy)$_4$(ClO$_4$)$_2$ as mediator. Blue trace: UV, λ = 254 nm; red trace: radioactivity. Abbreviation: p.-c.i – post-column injection.

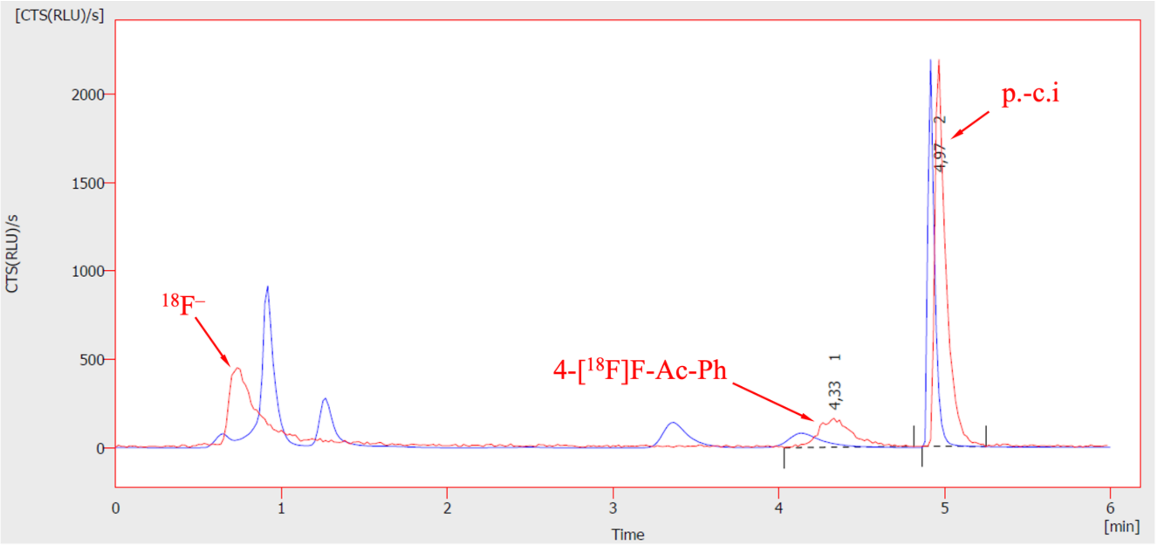

Result Table (Uncal - Data|alc-enhanced_657_30_03_2022_[18 DMA]4-acetylphenyl-SnMe3 - HERM)

	Reten. Time [min]	Area [CTS(RLU)/s.s]	Height [CTS(RLU)/s]	Area [%]
1	4,333	2391,500	164,319	19,1
2	4,967	10157,500	2187,261	80,9
	Total	12549,000	2351,580	100,0

Figure S18: HPLC traces of crude 4-[^{18}F]F-Ac-Ph prepared in *n*BuOH/DMA from **6** using Cu(Impdz)$_4$(ClO$_4$)$_2$ as mediator. Blue trace: UV, λ = 254 nm; red trace: radioactivity. Abbreviation: p.-c.i – post-column injection.

Screening of aprotic co-solvents

Radiosyntheses were performed according to GP5 with different copper(II) complexes and **1**–**4** in PC or DMI (n=3). RCCs were determined by radio-HPLC as described above. Representative radio-HPLC chromatograms of radiolabeled products are shown in Figure **S19** and Figure **S20**.

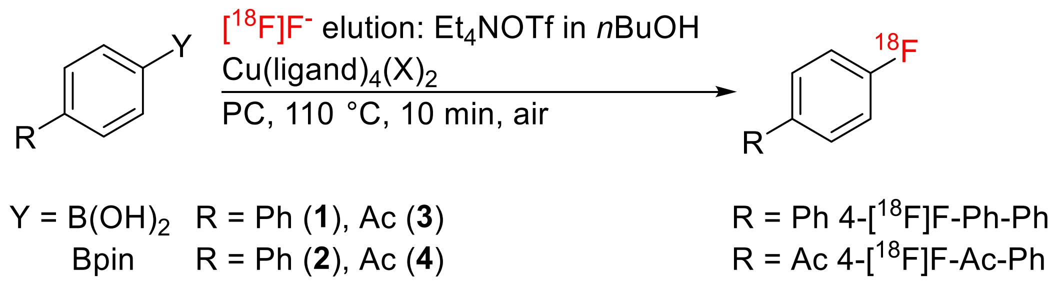

Table S25: Dependency of RCCs in *n*BuOH/PC on the applied copper mediator.

			RCC (%)			
**Entry**	**Ligand**	**X**	**1**	**2**	**3**	**4**
1	Py	OTf	46 ± 5	45 ± 5	66 ± 5	30 ± 7
2		ClO_4	99 ± 1	40 ± 7	76 ± 1	55 ± 9
3	2-MeOPy	OTf	17 ± 8	38 ± 8	16 ± 2	8 ± 3
4		ClO_4	49 ± 16	43 ± 8	39 ± 6	6 ± 1
5	3-MeOPy	OTf	90 ± 1	57 ± 4	72 ± 19	70 ± 16
6		ClO_4	67 ± 10	45 ± 10	73 ± 14	48 ± 15
7	4-MeOPy	OTf	27 ± 5	32 ± 1	25 ± 2	18 ± 2
8		ClO_4	57 ± 15	49 ± 12	50 ± 3	31 ± 6
9	4-PhPy	OTf	86 ± 9	73 ± 5	89 ± 4	68 ± 6
10		ClO_4	71 ± 4	80 ± 4	86 ± 5	80 ± 4
11	3,4-Me_2Py	OTf	68 ± 4	25 ± 3	53 ± 1	32 ± 8
12		ClO_4	53 ± 5	22 ± 6	71 ± 4	37 ± 8
13	Quin	OTf	20 ± 4	11 ± 2	3 ± 0.4	7 ± 3
14		ClO_4	4 ± 6	8 ± 2	3 ± 0.4	9 ± 4
15	Isoq	OTf	86 ± 2	52 ± 6	75 ± 6	51 ± 7
16		ClO_4	83 ± 6	53 ± 4	99 ± 1	67 ± 21
17	Impdz	OTf	41 ± 13	10 ± 1	29 ± 2	14 ± 3
18		ClO_4	55 ± 5	35 ± 1	78 ± 2	62 ± 6

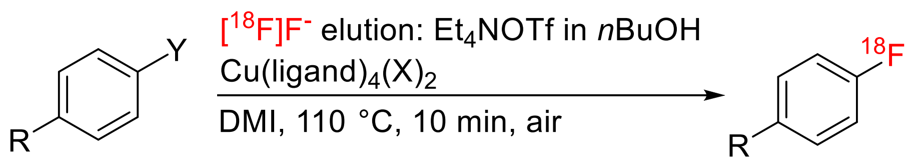

Y = B(OH)$_2$ R = Ph (**1**), Ac (**3**)
Bpin R = Ph (**2**), Ac (**4**)

R = Ph 4-[^{18}F]F-Ph-Ph
R = Ac 4-[^{18}F]F-Ac-Ph

Table S26: Dependency of RCCs in *n*BuOH/DMI on the applied copper mediator.

			RCC (%)			
**Entry**	**Ligand**	**X**	**1**	**2**	**3**	**4**
1	Py	OTf	93 ± 9	94 ± 7	45 ± 5	30 ± 7
2		ClO_4	82 ± 5	82 ± 5	56 ± 10	47 ± 10
3	2-MeOPy	OTf	9 ± 1	5 ± 0.4	2 ± 0.2	0
4		ClO_4	32 ± 8	13 ± 1	5 ± 2	1 ± 2
5	3-MeOPy	OTf	87 ± 2	71 ± 4	51 ± 8	35 ± 2
6		ClO_4	98 ± 1	78 ± 7	53 ± 6	39 ± 3
7	4-MeOPy	OTf	67 ± 1	69 ± 4	67 ± 6	51 ± 4
8		ClO_4	91 ± 3	86 ± 4	77 ± 4	64 ± 5
9	4-PhPy	OTf	86 ± 0.4	81 ± 4	57 ± 5	39 ± 6
10		ClO_4	98 ± 2	95 ± 5	86 ± 8	67 ± 3
11	3,4-Me_2Py	OTf	91 ± 1	93 ± 2	83 ± 6	81 ± 6
12		ClO_4	92 ± 3	89 ± 3	93 ± 2	84 ± 5
13	Quin	OTf	17 ± 3	12 ± 3	8 ± 1	7 ± 1
14		ClO_4	20 ± 2	23 ± 5	6 ± 1	7 ± 2
15	Isoq	OTf	93 ± 2	89 ± 3	79 ± 1	39 ± 1
16		ClO_4	95 ± 1	88 ± 2	80 ± 8	55 ± 4
17	Impdz	OTf	52 ± 2	37 ± 7	41 ± 3	24 ± 2
18		ClO_4	91 ± 2	67 ± 6	88 ± 1	64 ± 5

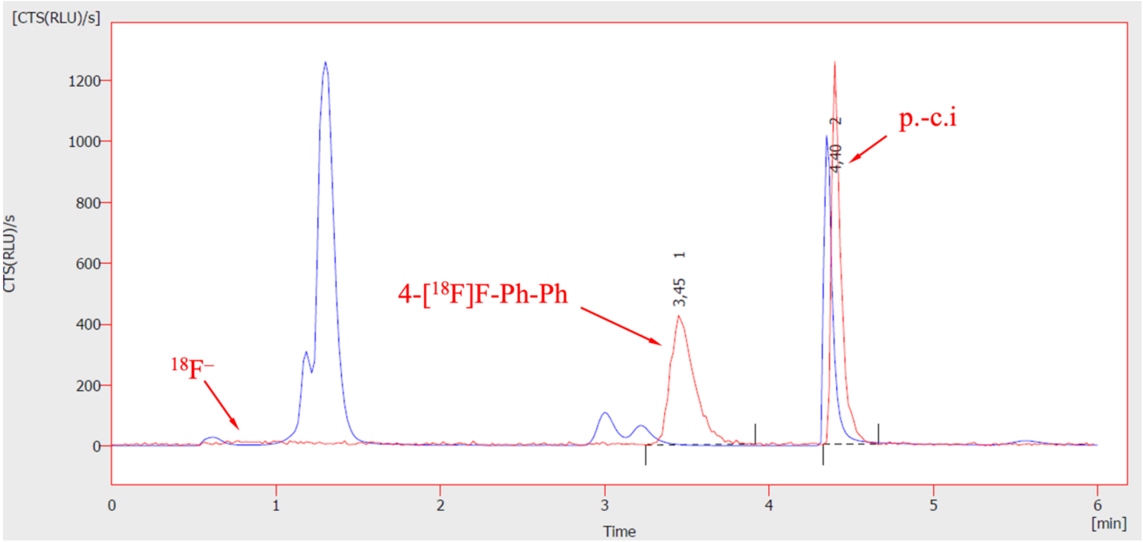

Result Table (Uncal - Data\alc-enhanced_536_21_02_2022_[4 DMI]_biphenyl-B(OH)2 - HERM)

	Reten. Time [min]	Area [CTS(RLU)/s.s]	Height [CTS(RLU)/s]	Area [%]
1	3,450	4338,000	425,100	46,2
2	4,400	5058,000	1257,000	53,8
	Total	9396,000	1682,100	100,0

Figure S19: HPLC traces of crude 4-[^{18}F]F-Ph-Ph prepared in *n*BuOH/DMI from **1** using Cu(4-PhPy)$_4$(ClO$_4$)$_2$ as mediator. Blue trace: UV, λ = 254 nm; red trace: radioactivity. Abbreviation: p.-c.i – post-column injection.

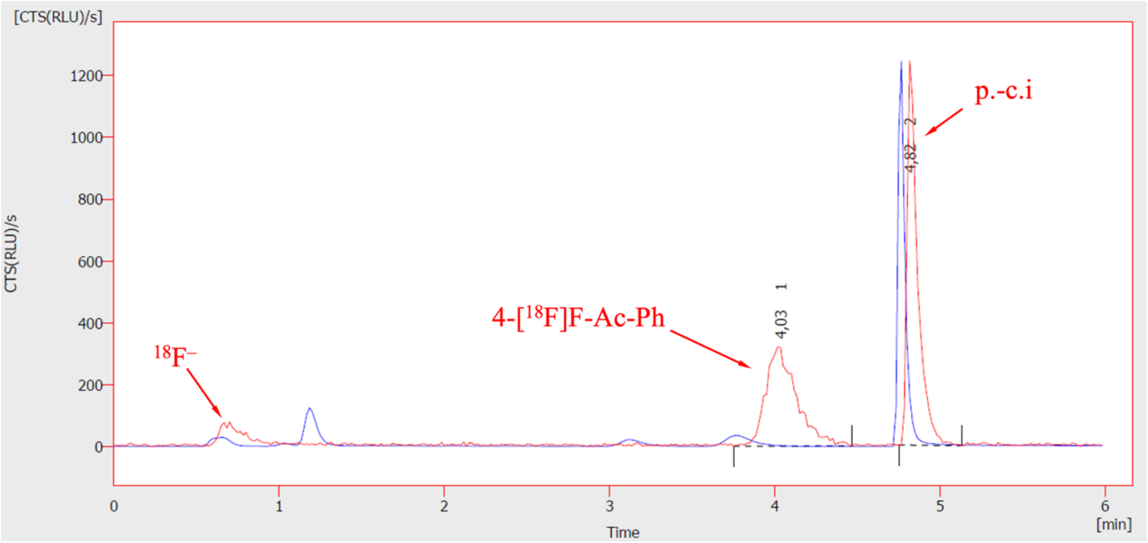

Result Table (Uncal - Data\alc-enhanced_538_21_02_2022_[4 DMI]_4-acetylphenyl-B(OH)2 - HERM)

	Reten. Time [min]	Area [CTS(RLU)/s.s]	Height [CTS(RLU)/s]	Area [%]
1	4,033	4179,500	320,605	42,7
2	4,817	5600,000	1242,348	57,3
	Total	9779,500	1562,952	100,0

Figure S20: HPLC traces of crude 4-[^{18}F]F-Ac-Ph prepared in *n*BuOH/DMI from **3** using Cu(4-PhPy)$_4$(ClO$_4$)$_2$ as mediator. Blue trace: UV, λ = 254 nm; red trace: radioactivity. Abbreviation: p.-c.i – post-column injection.

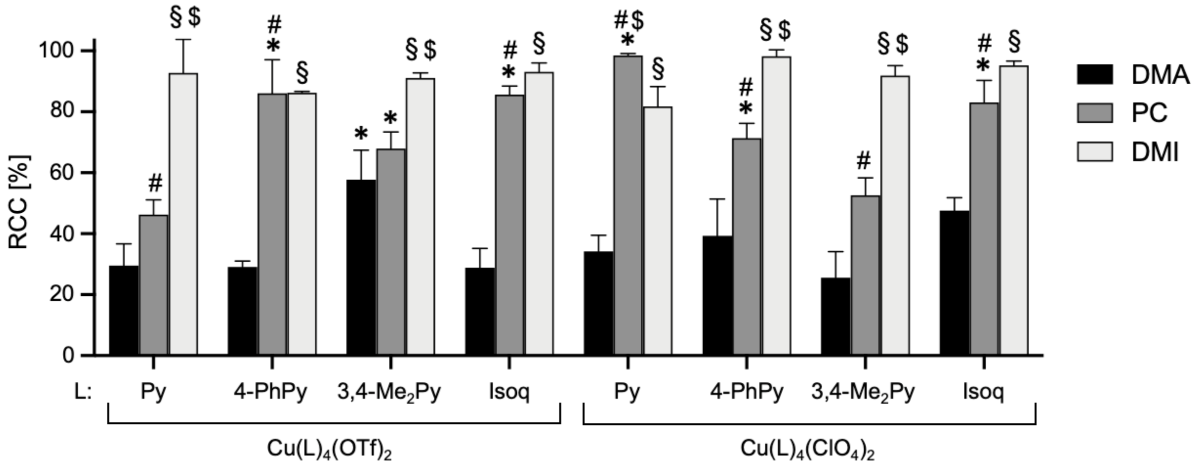

Figure S21: Dependency of RCCs on the aprotic co-solvent. Ph-Ph-B(OH)$_2$ was used as substrate for radiolabeling. Radiosyntheses were carried out according to GP5. Statistics: RCCs were compared using 2-way ANOVA with factors "solvent" and "Cu complex", using selected Cu complexes with the highest RCCs. Using Dunnett's post-hoc test, Cu complexes were compared to Cu(Py)$_4$(OTf)$_2$ (*$p<0.05$). Solvents were compared with Tukey's post-hoc test. Significant differences are labeled with the following symbols: #: $p<0.05$ for DMA vs. PC; §: $p<0.05$ for DMA vs. DMI; $: $p<0.05$ for DMI vs. PC.

Statistical data corresponding to Figure S21:

Table S27: RCCs (mean values ± standard deviation in %) for radiolabeling of 4-Ph-Ph-B$(OH)_2$ in *n*BuOH/DMA, *n*BuOH/PC or *n*BuOH/DMI using different mediators. 2-way ANOVA: Main effect of factor "**solvent**": F(2,48)=482.7, p<0.0001. Tukey's multiple comparisons test with n=3 per group.

	DMA *vs.* DMI	DMA *vs.* PC	DMI *vs.* PC
$Cu(Py)_4(OTf)_2$	DMA: 29.55 ± 7.14<br>DMI: 92.70 ± 11.01<br>p<0.0001§	DMA: 29.55 ± 7.14<br>PC: 46.30 ± 4.79<br>p=0.0053#	DMI: 92.70 ± 11.01<br>PC: 46.30 ± 4.79<br>p<0.0001$
$Cu(4\text{-}PhPy)_4(OTf)_2$	DMA: 29.09 ± 1.98<br>DMI: 86.08 ± 0.50<br>p<0.0001§	DMA: 29.09 ± 1.98<br>PC: 86.06 ± 11.03<br>p<0.0001#	DMI: 86.08 ± 0.50<br>PC: 86.06 ± 11.03<br>p=0.9996
$Cu(3,4\text{-}Me_2Py)_4(OTf)_2$	DMA: 57.76 ± 9.63<br>DMI: 91.04 ± 1.64<br>p<0.0001§	DMA: 57.76 ± 9.63<br>PC: 67.93 ± 5.44<br>p=0.1237	DMI: 91.04 ± 1.64<br>PC: 67.93 ± 5.44<br>p=0.0001$
$Cu(Isoq)_4(OTf)_2$	DMA: 28.80 ± 6.35<br>DMI: 93.05 ± 2.91<br>p<0.0001§	DMA: 28.80 ± 6.35<br>PC: 85.64 ± 2.84<br>p<0.0001#	DMI: 93.05 ± 2.91<br>PC: 85.64 ± 2.84<br>p=0.3216
$Cu(Py)_4(ClO_4)_2$	DMA: 34.21 ± 5.3<br>DMI: 81.68 ± 6.53<br>p<0.0001§	DMA: 34.21 ± 5.3<br>PC: 98.51 ± 0.57<br>p<0.0001#	DMI: 81.68 ± 6.53<br>PC: 98.51 ± 0.57<br>p=0.0050$
$Cu(4\text{-}PhPy)_4(ClO_4)_2$	DMA: 39.32 ± 12.07<br>DMI: 98.12 ± 2.14<br>p<0.0001§	DMA: 39.32 ± 12.07<br>PC: 71.42 ± 4.80<br>p<0.0001#	DMI: 98.12 ± 2.14<br>PC: 71.42 ± 4.80<br>p<0.0001$
$Cu(3,4\text{-}Me_2Py)_4(ClO_4)_2$	DMA: 25.57 ± 8.53<br>DMI: 91.78 ± 3.25<br>p<0.0001§	DMA: 25.57 ± 8.53<br>PC: 52.65 ± 5.65<br>p<0.0001#	DMI: 91.78 ± 3.25<br>PC: 52.65 ± 5.65<br>p<0.0001$
$Cu(Isoq)_4(ClO_4)_2$	DMA: 47.62 ± 4.19<br>DMI: 95.18 ± 1.38<br>p<0.0001§	DMA: 47.62 ± 4.19<br>PC: 83.08 ± 7.22<br>p<0.0001#	DMI: 95.18 ± 1.38<br>PC: 83.08 ± 7.22<br>p=0.0550

§green: RCCs in *n*BuOH/DMI were significantly higher than in *n*BuOH/DMA; # red: RCCs in *n*BuOH/PC were significantly higher than *n*BuOH DMA, $ blue: RCCs in *n*BuOH/DMI were significantly higher than in *n*BuOH/PC, $ pink: RCCs in *n*BuOH/PC were significantly higher than in *n*BuOH/DMI.

Table S28: RCCs (mean values ± standard deviation in %) for radiolabeling of 4-Ph-Ph-B$(OH)_2$ in *n*BuOH/DMA, *n*BuOH/PC or *n*BuOH/DMI using different mediators. 2-way ANOVA: Main effect of factor "**Cu-complex**": F(7,48)=11.60, p<0.0001. Dunnett's multiple comparisons test with $Cu(Py)_4(OTf)_2$ as control with n=3 per group.

	DMA	DMI	PC
$Cu(Py)_4(OTf)_2$	29.55 ± 7.14	92.70 ± 11.01	46.30 ± 4.79
$Cu(4\text{-}PhPy)_4(OTf)_2$	29.09 ± 1.98<br>p=0.9999	86.08 ± 0.50<br>p=0.6720	86.06 ± 11.03<br>p<0.0001*
$Cu(3,4\text{-}Me_2Py)_4(OTf)_2$	57.76 ± 9.63<br>p<0.0001*	91.04 ± 1.64<br>p=0.9995	67.93 ± 5.44<br>p=0.0007*
$Cu(Isoq)_4(OTf)_2$	28.80 ± 6.35<br>p=0.9998	93.05 ± 2.91<br>p=0.9999	85.64 ± 2.84<br>p<0.0001*
$Cu(Py)_4(ClO_4)_2$	34.21 ± 5.3<br>p=0.8988	81.68 ± 6.53<br>p=0.1671	98.51 ± 0.57<br>p<0.0001*
$Cu(4\text{-}PhPy)_4(ClO_4)_2$	39.32 ± 12.07<br>p=0.2659	98.12 ± 2.14<br>p=0.8187	71.42 ± 4.80<br>p<0.0001*
$Cu(3,4\text{-}Me_2Py)_4(ClO_4)_2$	25.57 ± 8.53<br>p=0.9502	91.78 ± 3.25<br>p=0.9997	52.65 ± 5.65<br>p=0.6954
$Cu(Isoq)_4(ClO_4)_2$	47.62 ± 4.19<br>p=0.0054*	95.18 ± 1.38<br>p=0.9952	83.08 ± 7.22<br>p<0.0001*

green: significantly higher RCCs compared to $Cu(Py)_4(OTf)_2$

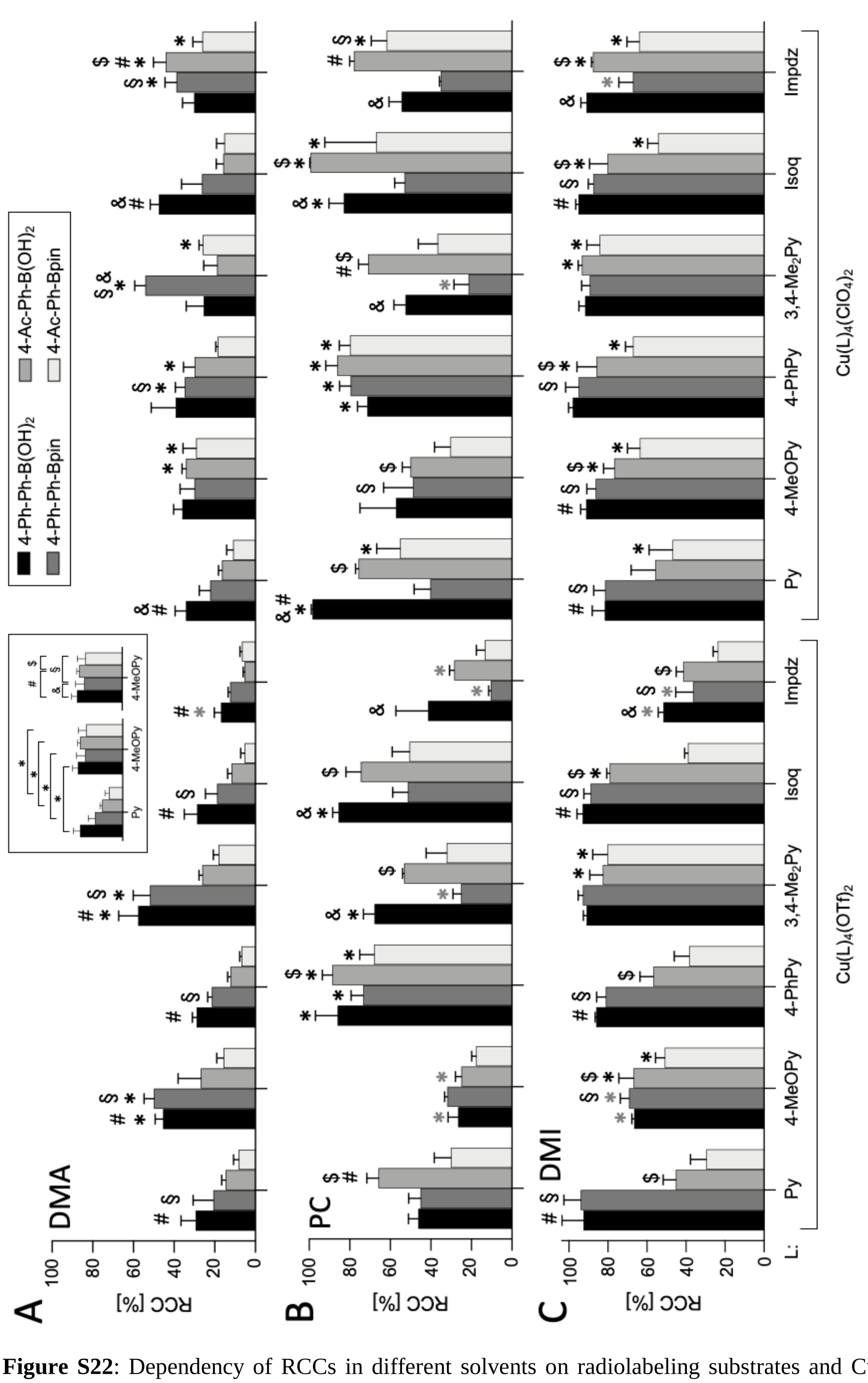

Figure S22: Dependency of RCCs in different solvents on radiolabeling substrates and Cu-complexes. A: Cu complexes in *n*BuOH/DMA. B: Cu complexes in *n*BuOH /PC. C: Cu complexes in *n*BuOH/DMI. Statistics: RCCs were compared using two-way ANOVA with factors "precursor" and "Cu complex". Using Dunnett's post-hoc test, Cu complexes were compared to $Cu(Py)_4(OTf)_2$ (*p<0.05). Precursors were compared with Tukey's post-hoc test. Significant differences are labeled with the following symbols: &: p<0.05 for 4-Ph-Ph-$B(OH)_2$ vs. 4-Ph-Ph-Bpin; #: p<0.05 for 4-Ph-Ph-$B(OH)_2$ vs. 4-Ac-Ph-$B(OH)_2$; §: p<0.05 for 4-Ph-Ph-Bpin vs. 4-Ac-Ph-Bpin; $: p<0.05 for 4-Ac-Ph-$B(OH)_2$ vs. 4-Ac-Ph-Bpin (for example, see insert).

Statistical data corresponding to Figure S22:

Table S29: RCCs (mean values ± standard deviation in %) in *n*BuOH/DMA. 2-way ANOVA: Main effect of factor "**Cu-complex**": $F(11,96)=33.63$, $p<0.0001$. Dunnett's multiple comparisons test with $Cu(Py)_4(OTf)_2$ as control with n=3 per group.

	4-Ph-Ph-B(OH)$_2$	4-Ph-Ph-Bpin	4-Ac-Ph-B(OH)$_2$	4-Ac-Ph-Bpin
$Cu(Py)_4(OTf)_2$	29.55 ± 7.14	20.82 ± 9.91	14.75 ± 1.93	8.29 ± 2.55
$Cu(4\text{-}MeOPy)_4(OTf)_2$	45.53 ± 3.81<br>p=0.0043*	49.96 ± 4.93<br>p<0.0001*	27.01 ± 11.05<br>p=0.0515	15.88 ± 3.17<br>p=0.4609
$Cu(4\text{-}PhPy)_4(OTf)_2$	29.09 ± 1.98<br>p=0.9999	21.48 ± 2.11<br>p=0.9998	12.37 ± 1.42<br>p=0.9991	7.00 ± 0.85<br>p=0.9996
$Cu(3,4\text{-}Me_2Py)_4(OTf)_2$	57.76 ± 9.63<br>p<0.0001*	51.96 ± 8.26<br>p<0.0001*	26.23 ± 1.57<br>p=0.0799	18.24 ± 2.52<br>p=0.1747
$Cu(Isoq)_4(OTf)_2$	28.80 ± 6.35<br>p=0.9998	19.00 ± 5.63<br>p=0.9994	11.80 ± 1.91<br>p=0.9957	5.43 ± 1.96<br>p=0.9960
$Cu(Impdz)_4(OTf)_2$	16.98 ± 3.27<br>p=0.0429*	12.51 ± 1.02<br>p=0.3535	5.37 ± 0.80<br>p=0.2271	6.70 ± 0.96<br>p=0.9995
$Cu(Py)_4(ClO_4)_2$	34.21 ± 5.3<br>p=0.9137	22.33 ± 5.31<br>p=0.9995	16.51 ± 1.73<br>p=0.0004	11.08 ± 3.17<br>p=0.9961
$Cu(4\text{-}MeOPy)_4(ClO_4)_2$	36.03 ± 4.24<br>p=0.6465	30.17 ± 6.96<br>p=0.2310	34.23 ± 1.92<br>p=0.0003*	29.33 ± 6.35<br>p<0.0001*
$Cu(4\text{-}PhPy)_4(ClO_4)_2$	39.32 ± 12.07<br>p=0.1905	34.88 ± 4.59<br>p=0.0167*	30.04 ± 5.39<br>p=0.0071*	18.55 ± 1.09<br>p=0.1508
$Cu(3,4\text{-}Me_2Py)_4(ClO_4)_2$	25.57 ± 8.53<br>p=0.9663	54.26 ± 5.24<br>p<0.0001*	19.00 ± 6.56<br>p=0.9494	25.97 ± 1.82<br>p=0.0112*
$Cu(Isoq)_4(ClO_4)_2$	47.62 ± 4.19<br>p=0.0009*	26.37 ± 10.07<br>p=0.8015	15.97 ± 3.35<br>p=0.9996	15.43 ± 3.77<br>p=0.5330
$Cu(Impdz)_4(ClO_4)_2$	30.21 ± 5.73<br>p=0.9998	39.00 ± 5.60<br>p=0.0008*	44.15 ± 6.10<br>p<0.0001*	26.24 ± 4.58<br>p=0.0009*

green: significantly higher RCCs compared to $Cu(Py)_4(OTf)_2$; red: significantly lower RCCs compared to $Cu(Py)_4(OTf)_2$.

Table S30: RCCs (mean values ± standard deviation in %) in *n*BuOH/DMA. 2-way ANOVA: Main effect of factor "**precursor**": $F(3,96)=100.8$, $p<0.0001$. Tukey's multiple comparisons test, n=3 per group.

	4-Ph-Ph-B(OH)$_2$ vs. 4-Ph-Ph-Bpin	4-Ph-Ph-B(OH)$_2$ vs. 4-Ac-Ph-B(OH)$_2$	4-Ph-Ph-Bpin vs. 4-Ac-Ph-Bpin	4-Ac-Ph-B(OH)$_2$ vs. 4-Ac-Ph-Bpin
Cu(Py)$_4$(OTf)$_2$	29.55 ± 7.14<br>20.82 ± 9.91<br>p=0.2015	29.55 ± 7.14<br>14.75 ± 1.93<br>p=0.0060[#]	20.82 ± 9.91<br>8.29 ± 2.55<br>p=0.0271[§]	14.75 ± 1.93<br>8.29 ± 2.55<br>p=0.4603
Cu(4-MeOPy)$_4$(OTf)$_2$	45.53 ± 3.81<br>49.96 ± 4.93<br>p=0.7463	45.53 ± 3.81<br>27.01 ± 11.05<br>p=0.0003[#]	49.96 ± 4.93<br>15.88 ± 3.17<br>p<0.0001[§]	27.01 ± 11.05<br>15.88 ± 3.17<br>p=0.0617
Cu(4-PhPy)$_4$(OTf)$_2$	29.09 ± 1.98<br>21.48 ± 2.11<br>p=0.3139	29.09 ± 1.98<br>12.37 ± 1.42<br>p=0.0014[#]	21.48 ± 2.11<br>7.00 ± 0.85<br>p=0.0075[§]	12.37 ± 1.42<br>7.00 ± 0.85<br>p=0.6160
Cu(3,4-Me$_2$Py)$_4$(OTf)$_2$	57.76 ± 9.63<br>51.96 ± 8.26<br>p=0.5538	57.76 ± 9.63<br>26.23 ± 1.57<br>p<0.0001[#]	51.96 ± 8.26<br>18.24 ± 2.52<br>p<0.0001[§]	26.23 ± 1.57<br>18.24 ± 2.52<br>p=0.2720
Cu(Isoq)$_4$(OTf)$_2$	28.80 ± 6.35<br>19.00 ± 5.63<br>p=0.1233	28.80 ± 6.35<br>11.80 ± 1.91<br>p=0.0011[#]	19.00 ± 5.63<br>5.43 ± 1.96<br>p=0.0140[§]	11.80 ± 1.91<br>5.43 ± 1.96<br>p=0.4736
Cu(Impdz)$_4$(OTf)$_2$	16.98 ± 3.27<br>12.51 ± 1.02<br>p=0.7397	16.98 ± 3.27<br>5.37 ± 0.80<br>p=0.0469[#]	12.51 ± 1.02<br>6.70 ± 0.96<br>p=0.5528	5.37 ± 0.80<br>6.70 ± 0.96<br>p=0.9903
Cu(Py)$_4$(ClO$_4$)$_2$	34.21 ± 5.32<br>22.33 ± 5.31<br>p=0.0402[&]	34.21 ± 5.32<br>16.51 ± 1.73<br>p=0.0007[#]	22.33 ± 5.31<br>11.08 ± 3.17<br>p=0.0578	16.51 ± 1.73<br>11.08 ± 3.17<br>p=0.6074
Cu(4-MeOPy)$_4$(ClO$_4$)$_2$	36.03 ± 4.24<br>30.17 ± 6.96<br>p=0.5442	36.03 ± 4.24<br>34.23 ± 1.92<br>p=0.9768	30.17 ± 6.96<br>29.33 ± 6.35<br>p=0.9976	34.23 ± 1.92<br>29.33 ± 6.35<br>p=0.6821
Cu(4-PhPy)$_4$(ClO4)2	39.32 ± 12.07<br>34.88 ± 4.59<br>p=0.7450	39.32 ± 12.07<br>30.04 ± 5.39<br>p=0.1576	34.88 ± 4.59<br>18.55 ± 1.09<br>p=0.0019[§]	30.04 ± 5.39<br>18.55 ± 1.09<br>p=0.0503

$Cu(3,4\text{-}Me_2Py)_4(ClO_4)_2$	25.57 ± 8.53	25.57 ± 8.53	54.26 ± 5.24	19.00 ± 6.56
	54.26 ± 5.24	19.00 ± 6.56	25.97 ± 1.82	25.97 ± 1.82
	p<0.0001*	p=0.4461	p<0.0001§	p=0.3930
$Cu(Isoq)_4(ClO_4)_2$	47.62 ± 4.19	47.62 ± 4.19	26.37 ± 10.07	15.97 ± 3.35
	26.37 ± 10.07	15.97 ± 3.35	15.43 ± 3.77	15.43 ± 3.77
	p<0.0001&	p<0.0001#	p=0.0688	p=0.9994
$Cu(Impdz)_4(ClO_4)_2$	30.21 ± 5.73	30.21 ± 5.73	39.00 ± 5.60	44.15 ± 6.10
	39.00 ± 5.60	44.15 ± 6.10	26.24 ± 4.58	26.24 ± 4.58
	p=0.1961	p=0.0109#	p=0.0236§	p=0.0006$

red: significantly lower RCCs for Bpin compared to $B(OH)_2$ precursors; blue: significantly higher RCCs for Bpin compared to $B(OH)_2$ precursors; green: significantly higher RCCs for 4-Ph-Ph compared to 4-Ac-Ph substituted precursors; pink: significantly lower RCCs for 4-Ph-Ph compared to 4-Ac-Ph precursors.

Table S31: RCCs (mean values ± standard deviation in %) in *n*BuOH/PC. 2-way ANOVA: Main effect of factor "**Cu-complex**": $F(11,96)=72.21$, $p<0.0001$. Dunnett's multiple comparisons test with $Cu(Py)_4(OTf)_2$ as control with n=3 per group.

	4-Ph-Ph-B(OH)$_2$	4-Ph-Ph-Bpin	4-Ac-Ph-B(OH)$_2$	4-Ac-Ph-Bpin
$Cu(Py)_4(OTf)_2$	46.30 ± 4.79	45.24 ± 5.77	65.90 ± 5.74	30.13 ± 8.30
$Cu(4\text{-}MeOPy)_4(OTf)_2$	26.69 ± 4.98<br>p=0.0246*	31.98 ± 1.28<br>p=0.2563	25.08 ± 2.94<br>p<0.0001*	17.75 ± 2.19<br>p=0.3289
$Cu(4\text{-}PhPy)_4(OTf)_2$	86.06 ± 11.03<br>p<0.0001*	73.44 ± 6.00<br>p=0.0003*	88.80 ± 4.88<br>p=0.0052*	68.20 ± 6.98<br>p<0.0001*
$Cu(3,4\text{-}Me_2Py)_4(OTf)_2$	67.93 ± 5.44<br>p=0.0097*	25.14 ± 4.00<br>p=0.0198*	53.37 ± 0.74<br>p=0.3162	32.40 ± 10.02<br>p=0.9995
$Cu(Isoq)_4(OTf)_2$	85.64 ± 2.84<br>p<0.0001*	51.55 ± 7.39<br>p=0.9432	74.71 ± 7.32<br>p=0.7209	50.67 ± 8.55<br>p=0.0163*
$Cu(Impdz)_4(OTf)_2$	41.41 ± 15.93<br>p=0.9897	10.44 ± 1.13<br>p<0.0001*	28.61 ± 2.32<br>p<0.0001*	13.51 ± 4.16<br>p=0.0832
$Cu(Py)_4(ClO_4)_2$	98.51 ± 0.57<br>p<0.0001*	40.30 ± 8.05<br>p=0.9892	75.83 ± 1.46<br>p=0.5886	55.39 ± 11.40<br>p=0.0015*
$Cu(4\text{-}MeOPy)_4(ClO_4)_2$	57.31 ± 17.79<br>p=0.4666	48.98 ± 14.43<br>p=0.9991	50.21 ± 3.95<br>p=0.1165	30.55 ± 7.72<br>p=0.9999
$Cu(4\text{-}PhPy)_4(ClO_4)_2$	71.42 ± 4.80<br>p=0.0016*	79.66 ± 5.46<br>p<0.0001*	86.39 ± 5.56<br>p=0.0166*	80.06 ± 5.20<br>p<0.0001*
$Cu(3,4\text{-}Me_2Py)_4(ClO_4)_2$	52.65 ± 5.65<br>p=0.9411	21.52 ± 7.15<br>p=0.0034*	71.01 ± 4.78<br>p=0.9868	36.87 ± 9.40<br>p=0.9173
$Cu(Isoq)_4(ClO_4)_2$	83.08 ± 7.22<br>p<0.0001*	53.08 ± 4.86<br>p=0.8250	99.39 ± 0.55<br>p<0.0001*	67.13 ± 25.26<br>p<0.0001*
$Cu(Impdz)_4(ClO_4)_2$	54.58 ± 6.19<br>p=0.7800	35.24 ± 0.59<br>p=0.5808	78.09 ± 2.05<br>p=0.3462	61.96 ± 7.53<br>p<0.0001*

green: significantly higher RCCs compared to $Cu(Py)_4(OTf)_2$; red: significantly lower RCCs compared to $Cu(Py)_4(OTf)_2$.

Table S32: RCCs (mean values ± standard deviation in %) in *n*BuOH/PC: 2-way ANOVA: Main effect of factor "**precursor**": $F(3,96)=88.15$, $p<0.0001$. Tukey's multiple comparisons test, n=3 per group.

	4-Ph-Ph-B(OH)$_2$ vs. 4-Ph-Ph-Bpin	4-Ph-Ph-B(OH)$_2$ vs. 4-Ac-Ph-B(OH)$_2$	4-Ph-Ph-Bpin vs. 4-Ac-Ph-Bpin	4-Ac-Ph-B(OH)$_2$ vs. 4-Ac-Ph-Bpin
Cu(Py)$_4$(OTf)$_2$	46.30 ± 4.79<br>45.24 ± 5.7<br>p=0.9984	46.30 ± 4.79<br>65.90 ± 5.74<br>p=0.0151[#]	45.24 ± 5.77<br>30.13 ± 8.30<br>p=0.0926	65.90 ± 5.74<br>30.13 ± 8.30<br>p<0.0001[$]
Cu(4-MeOPy)$_4$(OTf)$_2$	26.69 ± 4.98<br>31.98 ± 1.28<br>p=0.8426	26.69 ± 4.98<br>25.08 ± 2.94<br>p=0.9944	31.98 ± 1.28<br>17.75 ± 2.19<br>p=0.1253	25.08 ± 2.94<br>17.75 ± 2.19<br>p=0.6636
Cu(4-PhPy)$_4$(OTf)$_2$	86.06 ± 11.03<br>73.44 ± 6.00<br>p=0.1070	86.06 ± 11.03<br>88.80 ± 4.88<br>p=0.9735	73.44 ± 6.00<br>68.20 ± 6.98<br>p=0.8465	88.80 ± 4.88<br>68.20 ± 6.98<br>p=0.0095[$]
Cu(3,4-Me$_2$Py)$_4$(OTf)$_2$	67.93 ± 5.44<br>25.14 ± 4.00<br>p<0.0001[&]	67.93 ± 5.44<br>53.37 ± 0.74<br>p=0.1120	25.14 ± 4.00<br>32.40 ± 10.02<br>p=0.6706	53.37 ± 0.74<br>32.40 ± 10.02<br>p=0.0079[$]
Cu(Isoq)$_4$(OTf)$_2$	85.64 ± 2.84<br>51.55 ± 7.39<br>p<0.0001[&]	85.64 ± 2.84<br>74.71 ± 7.32<br>p=0.3263	51.55 ± 7.39<br>50.67 ± 8.55<br>p=0.9991	74.71 ± 7.32<br>50.67 ± 8.55<br>p=0.0017[$]
Cu(Impdz)$_4$(OTf)$_2$	41.41 ± 15.93<br>10.44 ± 1.13<br>p<0.0001[&]	41.41 ± 15.93<br>28.61 ± 2.32<br>p=0.1965	10.44 ± 1.13<br>13.51 ± 4.16<br>p=0.9636	28.61 ± 2.32<br>13.51 ± 4.16<br>p=0.0929
Cu(Py)$_4$(ClO$_4$)$_2$	98.51 ± 0.57<br>40.30 ± 8.05<br>p<0.0001[&]	98.51 ± 0.57<br>75.83 ± 1.46<br>p=0.0034[#]	40.30 ± 8.05<br>55.39 ± 11.40<br>p=0.0931	75.83 ± 1.46<br>55.39 ± 11.40<br>p=0.0102[$]
Cu(4-MeOPy)$_4$(ClO$_4$)$_2$	57.31 ± 17.79<br>48.98 ± 14.43<br>p=0.5654	57.31 ± 17.79<br>50.21 ± 3.95<br>p=0.6857	48.98 ± 14.43<br>30.55 ± 7.72<br>p=0.0252[§]	50.21 ± 3.95<br>30.55 ± 7.72<br>p=0.0147[$]
Cu(4-PhPy)$_4$(ClO$_4$)2	71.42 ± 4.80<br>79.66 ± 5.46<br>p=0.5740	71.42 ± 4.80<br>86.39 ± 5.56<br>p=0.0970	79.66 ± 5.46<br>80.06 ± 5.20<br>p=0.9999	86.39 ± 5.56<br>80.06 ± 5.20<br>p=0.7565

$Cu(3,4\text{-}Me_2Py)_4(ClO_4)_2$	52.65 ± 5.65<br>21.52 ± 7.15<br>p<0.0001[&]	52.65 ± 5.65<br>71.01 ± 4.78<br>p=0.0260[#]	21.52 ± 7.15<br>36.87 ± 9.40<br>p=0.0849	71.01 ± 4.78<br>36.87 ± 9.40<br>p<0.0001[$]
$Cu(Isoq)_4(ClO_4)_2$	83.08 ± 7.22<br>53.08 ± 4.86<br>p<0.0001[&]	83.08 ± 7.22<br>99.39 ± 0.55<br>p=0.0593	53.08 ± 4.86<br>67.13 ± 25.26<br>p=0.1328	99.39 ± 0.55<br>67.13 ± 25.26<br>p<0.0001[$]
$Cu(Impdz)_4(ClO_4)_2$	54.58 ± 6.19<br>35.24 ± 0.59<br>p=0.0169[&]	54.58 ± 6.19<br>78.09 ± 2.05<br>p=0.0022[#]	35.24 ± 0.59<br>61.96 ± 7.53<br>p=0.0004[§]	78.09 ± 2.05<br>61.96 ± 7.53<br>p=0.0636

red: significantly lower RCCs for Bpin compared to $B(OH)_2$ precursors; green: significantly higher RCCs for 4-Ph-Ph compared to 4-Ac-Ph substituted precursors; pink: significantly lower RCCs for 4-Ph-Ph compared to 4-Ac-Ph precursors.

Table S33: RCCs (mean values ± standard deviation in %) in *n*BuOH/DMI. 2-way ANOVA: Main effect of factor "**Cu-complex**": $F(11,96)=68.60$, $p<0.0001$. Dunnett's multiple comparisons test with $Cu(Py)_4(OTf)_2$ as control with n=3 per group.

	4-Ph-Ph-B(OH)$_2$	4-Ph-Ph-Bpin	4-Ac-Ph-B(OH)$_2$	4-Ac-Ph-Bpin
$Cu(Py)_4(OTf)_2$	92.70 ± 11.01	94.05 ± 8.57	45.39 ± 6.48	29.74 ± 8.00
$Cu(4\text{-}MeOPy)_4(OTf)_2$	66.74 ± 1.09<br>p<0.0001*	69.40 ± 4.34<br>p<0.0001*	67.00 ± 7.53<br>p=0.0002*	51.06 ± 4.71<br>p=0.0003*
$Cu(4\text{-}PhPy)_4(OTf)_2$	86.08 ± 0.50<br>p=0.7251	81.23 ± 4.60<br>p=0.0724	56.91 ± 6.73<br>p=0.1350	38.57 ± 7.56<br>p=0.3933
$Cu(3,4\text{-}Me_2Py)_4(OTf)_2$	91.04 ± 1.64<br>p=0.9995	92.93 ± 2.43<br>p=0.9997	82.70 ± 6.77<br>p<0.0001*	80.51 ± 7.39<br>p<0.0001*
$Cu(Isoq)_4(OTf)_2$	93.05 ± 2.91<br>p=0.9999	89.07 ± 3.38<br>p=0.9268	79.21 ± 1.50<br>p<0.0001*	39.23 ± 1.62<br>p=0.3125
$Cu(Impdz)_4(OTf)_2$	51.67 ± 2.71<br>p<0.0001*	36.58 ± 8.83<br>p<0.0001*	41.44 ± 3.65<br>p=0.9839	23.86 ± 2.27<br>p=0.8307
$Cu(Py)_4(ClO_4)_2$	81.68 ± 6.53<br>p=0.1688	81.63 ± 5.73<br>p=0.0885	55.82 ± 12.36<br>p=0.2165	46.98 ± 12.00<br>p=0.0054*
$Cu(4\text{-}MeOPy)_4(ClO_4)_2$	91.27 ± 2.82<br>p=0.9996	86.41 ± 4.57<br>p=0.5669	76.95 ± 5.34<br>p<0.0001*	63.81 ± 6.33<br>p<0.0001*
$Cu(4\text{-}PhPy)_4(ClO_4)_2$	98.12 ± 2.14<br>p=0.8851	95.15 ± 6.67<br>p=0.9997	86.04 ± 9.90<br>p<0.0001*	67.29 ± 3.82<br>p<0.0001*
$Cu(3,4\text{-}Me_2Py)_4(ClO_4)_2$	91.78 ± 3.25<br>p=0.9997	89.44 ± 4.16<br>p=0.9523	93.47 ± 1.89<br>p<0.0001*	84.41 ± 6.50<br>p<0.0001*
$Cu(Isoq)_4(ClO_4)_2$	95.18 ± 1.38<br>p=0.9992	87.62 ± 2.54<br>p=0.7535	80.31 ± 9.19<br>p<0.0001*	54.51 ± 5.24<br>p<0.0001*
$Cu(Impdz)_4(ClO_4)_2$	91.16 ± 2.84<br>p=0.9996	67.31 ± 7.17<br>p<0.0001*	87.79 ± 0.79<br>p<0.0001*	64.15 ± 6.21<br>p<0.0001*

green: significantly higher RCCs compared to $Cu(Py)_4(OTf)_2$; red: significantly lower RCCs compared to $Cu(Py)_4(OTf)_2$.

Table S34: RCCs (mean values ± standard deviation in %) in *n*BuOH/DMI. 2-way ANOVA: Main effect of factor "**precursor**": $F(3,96)=155.4$, $p<0.0001$. Tukey's multiple comparisons test, n=3 per group.

	4-Ph-Ph-B(OH)$_2$ *vs.* 4-Ph-Ph-Bpin	4-Ph-Ph-B(OH)$_2$ *vs.* 4-Ac-Ph-B(OH)$_2$	4-Ph-Ph-Bpin *vs.* 4-Ac-Ph-Bpin	4-Ac-Ph-B(OH)$_2$ *vs.* 4-Ac-Ph-Bpin
Cu(Py)$_4$(OTf)$_2$	92.70 ± 11.01<br>94.05 ± 8.57<br>p=0.9924	92.70 ± 11.01<br>45.39 ± 6.48<br>p<0.0001[#]	94.05 ± 8.57<br>29.74 ± 8.00<br>p<0.0001[§]	45.39 ± 6.48<br>29.74 ± 8.00<br>p=0.0089[$]
Cu(4-MeOPy)$_4$(OTf)$_2$	66.74 ± 1.09<br>69.40 ± 4.34<br>p=0.9464	66.74 ± 1.09<br>67.00 ± 7.53<br>p=0.9999	69.40 ± 4.34<br>51.06 ± 4.71<br>p=0.0015[§]	67.00 ± 7.53<br>51.06 ± 4.71<br>p=0.0074[$]
Cu(4-PhPy)$_4$(OTf)$_2$	86.08 ± 0.50<br>81.23 ± 4.60<br>p=0.7475	86.08 ± 0.50<br>56.91 ± 6.73<br>p<0.0001[#]	81.23 ± 4.60<br>38.57 ± 7.56<br>p<0.0001[§]	56.91 ± 6.73<br>38.57 ± 7.56<br>p=0.0015[$]
Cu(3,4-Me$_2$Py)$_4$(OTf)$_2$	91.04 ± 1.64<br>92.93 ± 2.43<br>p=0.9795	91.04 ± 1.64<br>82.70 ± 6.77<br>p=0.3174	92.93 ± 2.43<br>80.51 ± 7.39<br>p=0.0562	82.70 ± 6.77<br>80.51 ± 7.39<br>p=0.9688
Cu(Isoq)$_4$(OTf)$_2$	93.05 ± 2.91<br>89.07 ± 3.38<br>p=0.8439	93.05 ± 2.91<br>79.21 ± 1.50<br>p=0.0263[#]	89.07 ± 3.38<br>39.23 ± 1.62<br>p<0.0001[§]	79.21 ± 1.50<br>39.23 ± 1.62<br>p<0.0001[$]
Cu(Impdz)$_4$(OTf)$_2$	51.67 ± 2.71<br>36.58 ± 8.83<br>p=0.0126[&]	51.67 ± 2.71<br>41.44 ± 3.65<br>p=0.1554	36.58 ± 8.83<br>23.86 ± 2.27<br>p=0.0483[§]	41.44 ± 3.65<br>23.86 ± 2.27<br>p=0.0025[$]
Cu(Py)$_4$(ClO$_4$)$_2$	81.68 ± 6.53<br>81.63 ± 5.73<br>p=0.9999	81.68 ± 6.53<br>55.82 ± 12.36<br>p<0.0001[#]	81.63 ± 5.73<br>46.98 ± 12.00<br>p<0.0001[§]	55.82 ± 12.36<br>46.98 ± 12.00<br>p=0.2667
Cu(4-MeOPy)$_4$(ClO$_4$)$_2$	91.27 ± 2.82<br>86.41 ± 4.57<br>p=0.7475	91.27 ± 2.82<br>76.95 ± 5.34<br>p=0.0200[#]	86.41 ± 4.57<br>63.81 ± 6.33<br>p<0.0001[§]	76.95 ± 5.34<br>63.81 ± 6.33<br>p=0.0386[$]
Cu(4-PhPy)$_4$(ClO$_4$)2	98.12 ± 2.14<br>95.15 ± 6.67<br>p=0.9272	98.12 ± 2.14<br>86.04 ± 9.90<br>p=0.0669	95.15 ± 6.67<br>67.29 ± 3.82<br>p<0.0001[§]	86.04 ± 9.90<br>67.29 ± 3.82<br>p=0.0011[$]

$Cu(3,4\text{-}Me_2Py)_4(ClO_4)_2$	91.78 ± 3.25<br>89.44 ± 4.16<br>p=0.9624	91.78 ± 3.25<br>93.47 ± 1.89<br>p=0.9854	89.44 ± 4.16<br>84.41 ± 6.50<br>p=0.7262	93.47 ± 1.89<br>84.41 ± 6.50<br>p=0.2463
$Cu(Isoq)_4(ClO_4)_2$	95.18 ± 1.38<br>87.62 ± 2.54<br>p=0.4044	95.18 ± 1.38<br>80.31 ± 9.19<br>p=0.0143[#]	87.62 ± 2.54<br>54.51 ± 5.24<br>p<0.0001[§]	80.31 ± 9.19<br>54.51 ± 5.24<br>p<0.0001[$]
$Cu(Impdz)_4(ClO_4)_2$	91.16 ± 2.84<br>67.31 ± 7.17<br>p<0.0001[&]	91.16 ± 2.84<br>87.79 ± 0.79<br>p=0.8982	67.31 ± 7.17<br>64.15 ± 6.21<br>p=0.9137	87.79 ± 0.79<br>64.15 ± 6.21<br>p<0.0001[$]

red: significantly lower RCCs for Bpin compared to $B(OH)_2$ precursors; green: significantly higher RCCs for 4-Ph-Ph compared to 4-Ac-Ph substituted precursors.

Cu(Impdz)$_4$(OTf)$_2$ *vs.* Cu(Impdz)$_4$(ClO$_4$)$_2$

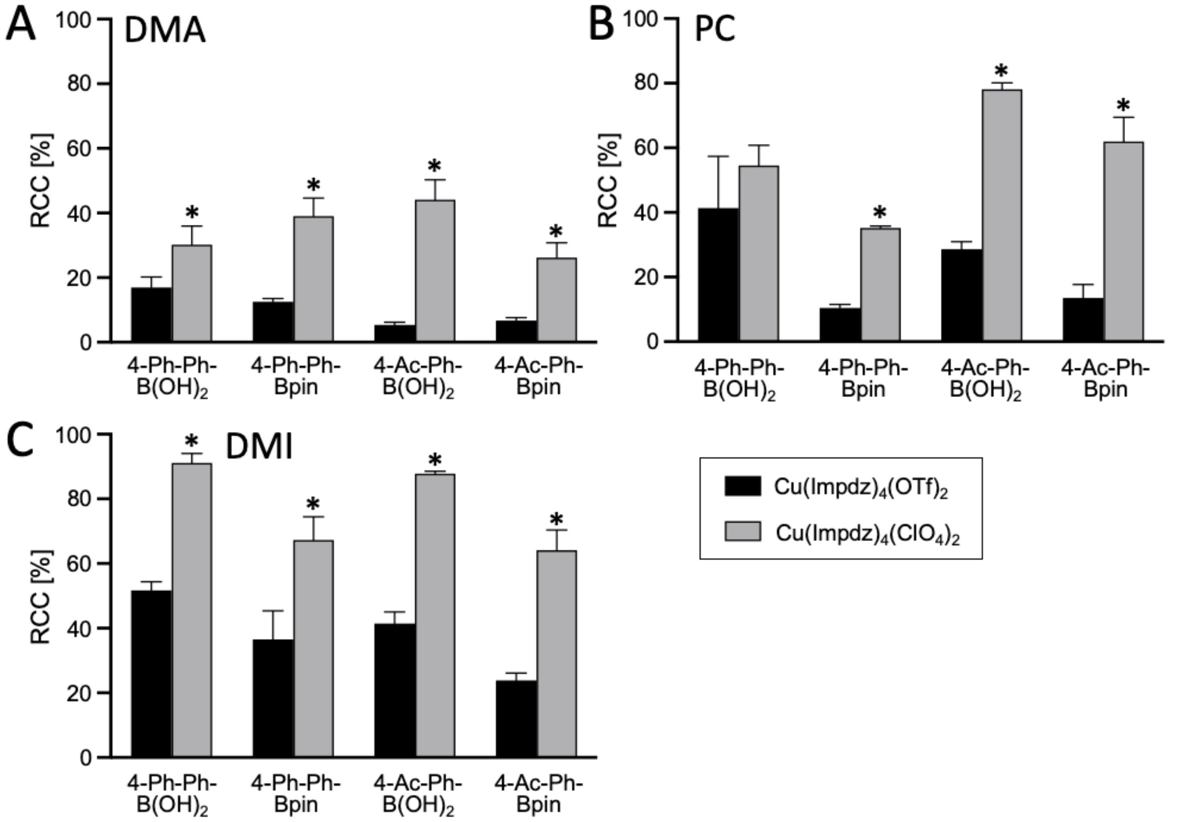

Figure S23: Cu(Impdz)$_4$(OTf)$_2$ *vs.* Cu(Impdz)$_4$(ClO$_4$)$_2$. * $p<0.05$. For full statistics, see Table S35.

Table S35: RCCs (mean values ± standard deviation in %) obtained using $Cu(Impdz)_4(OTf)_2$ and $Cu(Impdz)_4(ClO_4)_2$ as radiofluorination mediators. Comparison was carried out using 2-way ANOVA followed by Sidak's multiple comparison test (n=3 per group). Main effects (F-values) of the factor "**counter ion**" are given.

		$Cu(Impdz)_4(OTf)_2$	$Cu(Impdz)_4(ClO_4)_2$	
DMA	4-Ph-Ph-$B(OH)_2$	16.98 ± 3.27	30.21 ± 5.73	p=0.0047*
F(1,16)=212.6	4-Ph-Ph-Bpin	12.51 ± 1.02	39.00 ± 5.60	p<0.0001*
p<0.0001	4-Ac-Ph-$B(OH)_2$	5.37 ± 0.80	44.15 ± 6.10	p<0.0001*
	4-Ac-Ph-Bpin	6.70 ± 0.96	26.24 ± 4.58	p=0.0001*
PC	4-Ph-Ph-$B(OH)_2$	41.41 ± 15.93	54.58 ± 6.19	p=0.1219
F(1,16)=146.8	4-Ph-Ph-Bpin	10.44 ± 1.13	35.24 ± 0.59	p=0.0017*
p<0.0001	4-Ac-Ph-$B(OH)_2$	28.61 ± 2.32	78.09 ± 2.05	p<0.0001*
	4-Ac-Ph-Bpin	13.51 ± 4.16	61.96 ± 7.53	p<0.0001*
DMI	4-Ph-Ph-$B(OH)_2$	51.65 ± 2.71	91.16 ± 2.84	p<0.0001*
F(1,16)=364.4	4-Ph-Ph-Bpin	36.58 ± 8.83	67.31 ± 7.17	p<0.0001*
p<0.0001	4-Ac-Ph-$B(OH)_2$	41.44 ± 3.65	87.79 ± 0.79	p<0.0001*
	4-Ac-Ph-Bpin	23.86 ± 2.27	64.15 ± 6.21	p<0.0001*

Screening of reaction temperatures

The screening of reaction temperatures was performed in *n*BuOH/DMI according to GP5 using **1** as model substrate and Cu(4-PhPy)$_4$(ClO$_4$)$_2$ as mediator (n=3). RCCs were determined by radio-HPLC as described above. Representative HPLC chromatograms of the crude radiolabeled products are shown in Figure **S25–S27**.

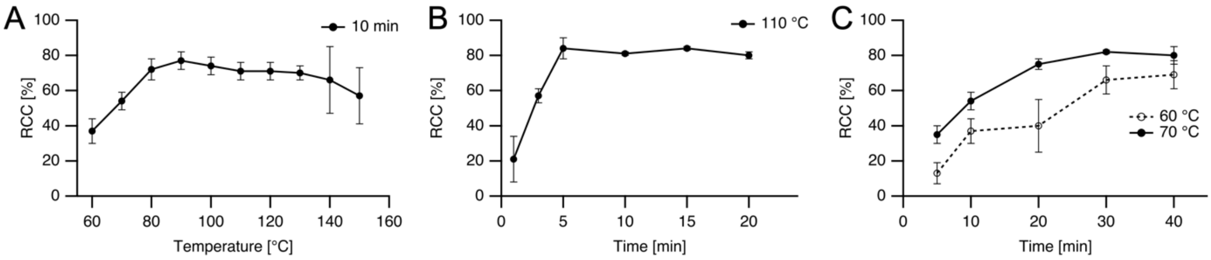

Figure S24: Temperature (A) and reaction time studies (B and C) for Cu(4-PhPy)$_4$(ClO$_4$)$_2$-mediated radiofluorination of 4-Ph-Ph-B(OH)$_2$. Conditions: [^{18}F]F$^-$ (10–50 MBq) was eluted with Et$_4$NOTf (1 mg, 4 µmol) in *n*BuOH (400 µL) into a solution of 4-Ph-Ph-B(OH)$_2$ (10 µmol, 1 eq.) and Cu(4-PhPy)$_4$(ClO$_4$)$_2$ (10 µmol, 1 eq.) in DMI (800 µL),; (A) 60–150 °C as indicated for 10 min. (B) 1–20 min as indicated at 110 °C (C) 10–40 min at 60 or 70 °C as indicated. All radiosyntheses were carried out in triplicate.

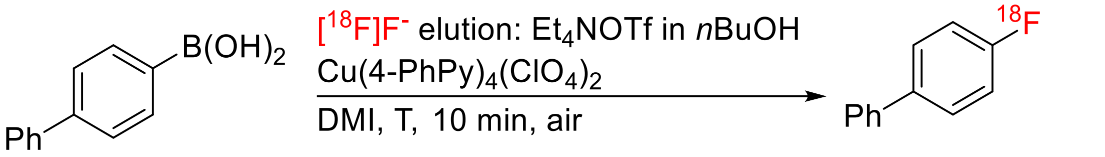

1

4-[^{18}F]F-Ph-Ph

Table S36: Screening of temperature.

Entry	T [°C]	RCC [%]
1	rt	0
2	60	37 ± 7
3	70	54 ± 5
4	80	72 ± 6
5	90	77 ± 5
6	100	74 ± 5
7	110	71 ± 5
8	120	71 ± 5
9	130	70 ± 4
10	140	66 ± 19
11	150	57 ± 16

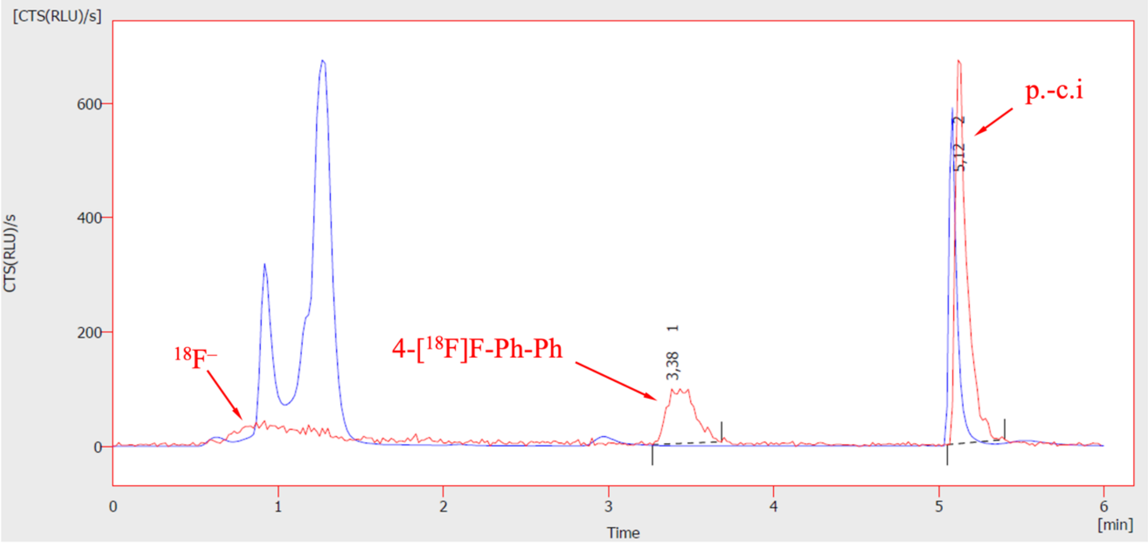

Result Table (Uncal - Data|alc-enhanced_328_03_11_2021_[4 DMI]_biphenyl-B(OH)2_60 - HERM)

	Reten. Time [min]	Area [CTS(RLU)/s.s]	Height [CTS(RLU)/s]	Area [%]
1	3,383	1164,500	96,600	25,6
2	5,117	3377,500	671,286	74,4
	Total	4542,000	767,886	100,0

Figure S25: HPLC traces of crude 4-[^{18}F]F-Ph-Ph prepared from **1** using Cu(4-PhPy)$_4$(ClO$_4$)$_2$ as mediator in *n*BuOH/DMI at 60 °C for 10 min. Blue trace: UV, λ = 254 nm; red trace: radioactivity. Abbreviation: p.-c.i – post-column injection.

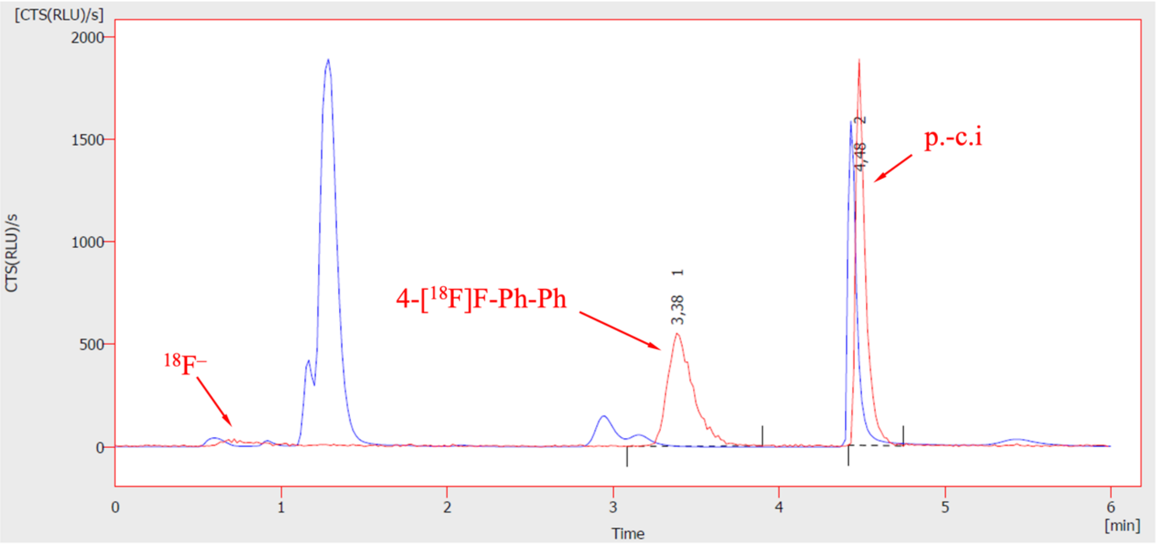

Result Table (Uncal - Data|alc-enhanced_678_08_04_2022_[4 DMI]biphenyl-B(OH)2_90°C - HERM)

	Reten. Time [min]	Area [CTS(RLU)/s.s]	Height [CTS(RLU)/s]	Area [%]
1	3,383	5980,000	551,265	43,5
2	4,483	7767,000	1884,600	56,5
	Total	13747,000	2435,865	100,0

Figure S26: HPLC traces of crude 4-[^{18}F]F-Ph-Ph prepared from **1** using Cu(4-PhPy)$_4$(ClO$_4$)$_2$ as mediator in *n*BuOH/DMI at 90 °C for 10 min. Blue trace: UV, λ = 254 nm; red trace: radioactivity. Abbreviation: p.-c.i – post-column injection.

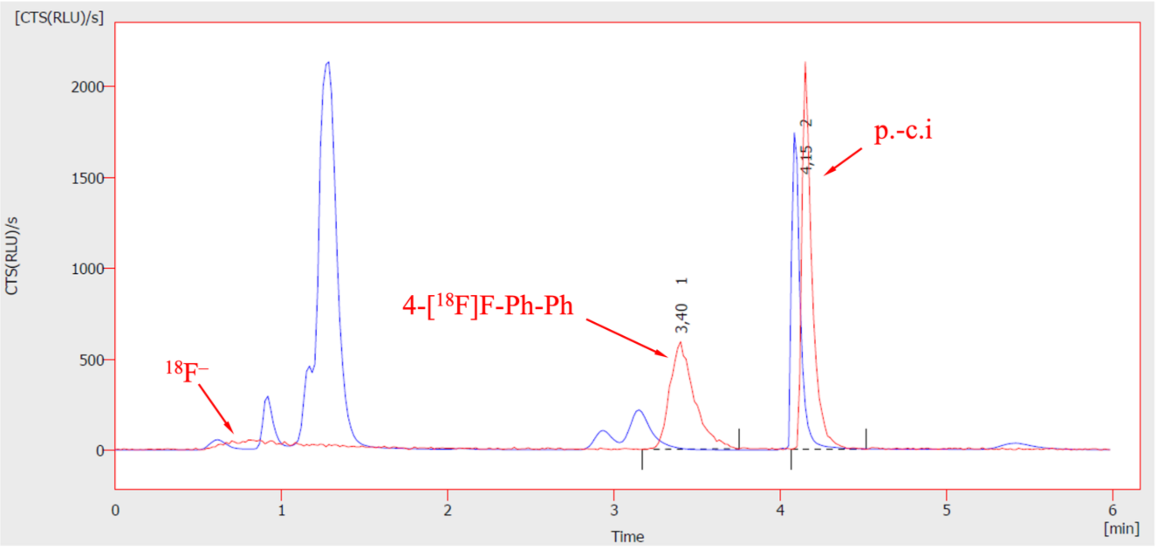

Result Table (Uncal - Data|alc-enhanced_698_11_04_2022_[4 DMI]biphenyl-B(OH)2_150°C - HERM)

	Reten. Time [min]	Area [CTS(RLU)/s.s]	Height [CTS(RLU)/s]	Area [%]
1	3,400	6051,500	591,800	40,1
2	4,150	9030,000	2134,000	59,9
	Total	15081,500	2725,800	100,0

Figure S27: HPLC traces of crude 4-[^{18}F]F-Ph-Ph prepared from **1** using Cu(4-PhPy)$_4$(ClO$_4$)$_2$ as mediator in *n*BuOH/DMI at 150 °C for 10 min. Blue trace: UV, λ = 254 nm; red trace: radioactivity. Abbreviation: p.-c.i – post-column injection.

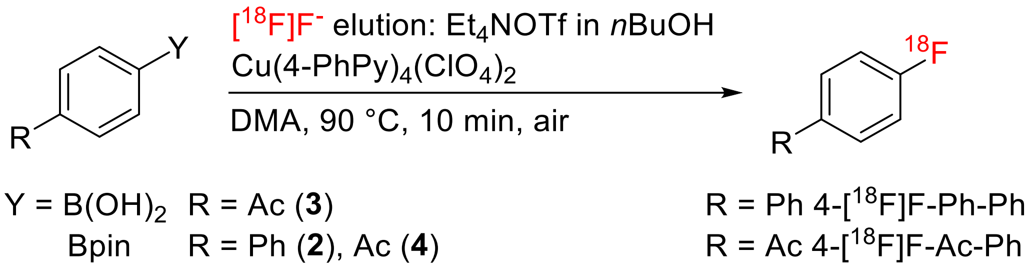

Table S37: Radiolabeling of precursors **2**–**4** at 90 °C for 10 min.

Entry	Precursor	RCC [%]
1	**2**	39 ± 10
2	**3**	44 ± 2
3	**4**	21 ± 2

Screening of reaction times

The screening of various reaction times at different temperatures was performed according to GP5 using **1** as model radiolabeling substrate and $Cu(4\text{-}PhPy)_4(ClO_4)_2$ as mediator in *n*BuOH/DMI. RCCs were determined by radio-HPLC as described above. Representative HPLC chromatograms of the crude radiolabeled products are shown in Figure **S28–S36**.

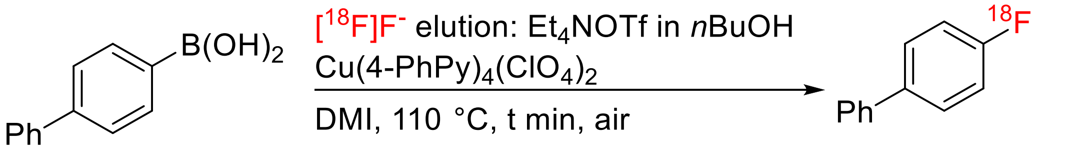

1

4-[^{18}F]F-Ph-Ph

Table S38: Screening of reaction time at 110 °C.

Entry	t [min]	RCC [%]
1	1	21 ± 13
2	3	57 ± 4
3	5	84 ± 6
4	10	81 ± 1
5	15	84 ± 1
6	20	80 ± 2
7	30	59 ± 5
8	40	63 ± 13

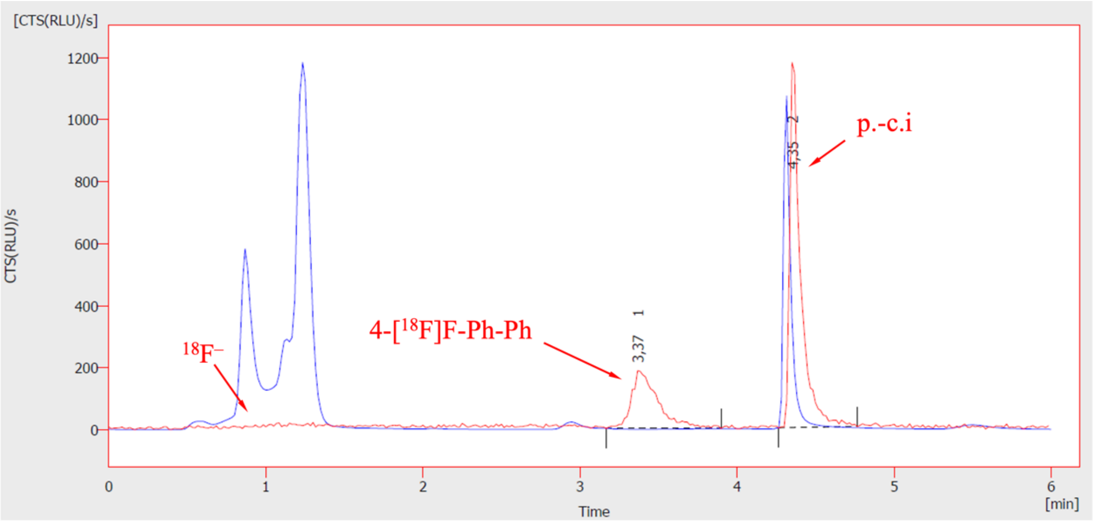

Result Table (Uncal - Data\alc-enhanced_480_04_02_2022_[4 DMI]_biphenyl_B(OH)2_rkt_1min - HERM)

	Reten. Time [min]	Area [CTS(RLU)/s.s]	Height [CTS(RLU)/s]	Area [%]
1	3,367	2204,000	186,000	27,7
2	4,350	5744,000	1177,333	72,3
	Total	7948,000	1363,333	100,0

Figure S28: HPLC traces of crude 4-[^{18}F]F-Ph-Ph prepared from **1** using Cu(4-PhPy)$_4$(ClO$_4$)$_2$ as mediator in *n*BuOH/DMI at 110 °C for 1 min. Blue trace: UV, λ = 254 nm; red trace: radioactivity. Abbreviation: p.-c.i – post-column injection.

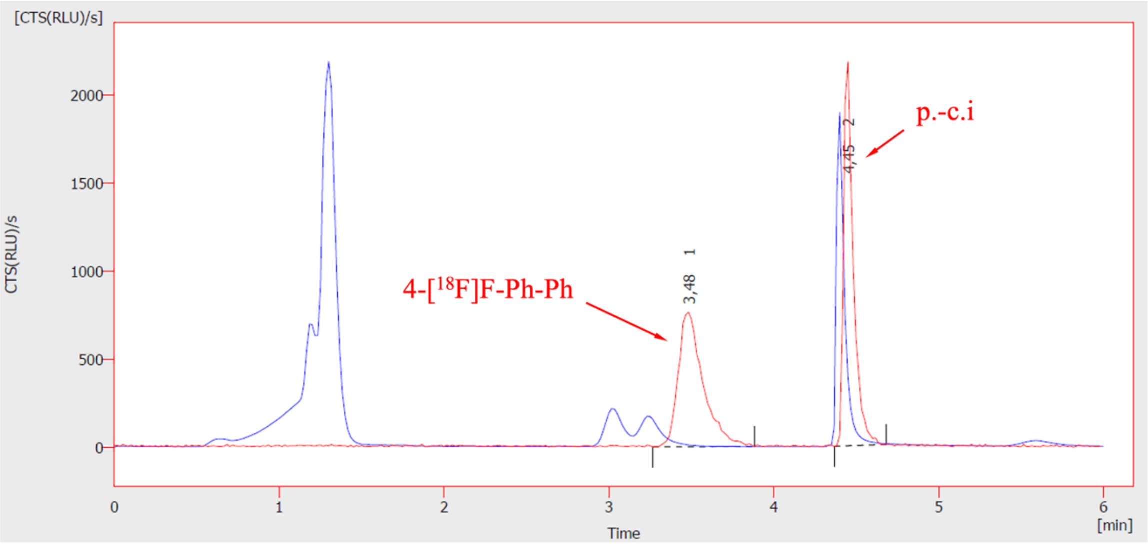

Result Table (Uncal - Data\alc-enhanced_474_04_02_2022_[4 DMI]_biphenyl_B(OH)2_rkt_15min - HERM)

	Reten. Time [min]	Area [CTS(RLU)/s.s]	Height [CTS(RLU)/s]	Area [%]
1	3,483	8086,000	765,297	47,2
2	4,450	9039,500	2181,105	52,8
	Total	17125,500	2946,403	100,0

Figure S29: HPLC traces of crude 4-[^{18}F]F-Ph-Ph prepared from **1** using Cu(4-PhPy)$_4$(ClO$_4$)$_2$ as mediator in *n*BuOH/DMI at 110 °C for 15 min. Blue trace: UV, λ = 254 nm; red trace: radioactivity. Abbreviation: p.-c.i – post-column injection.

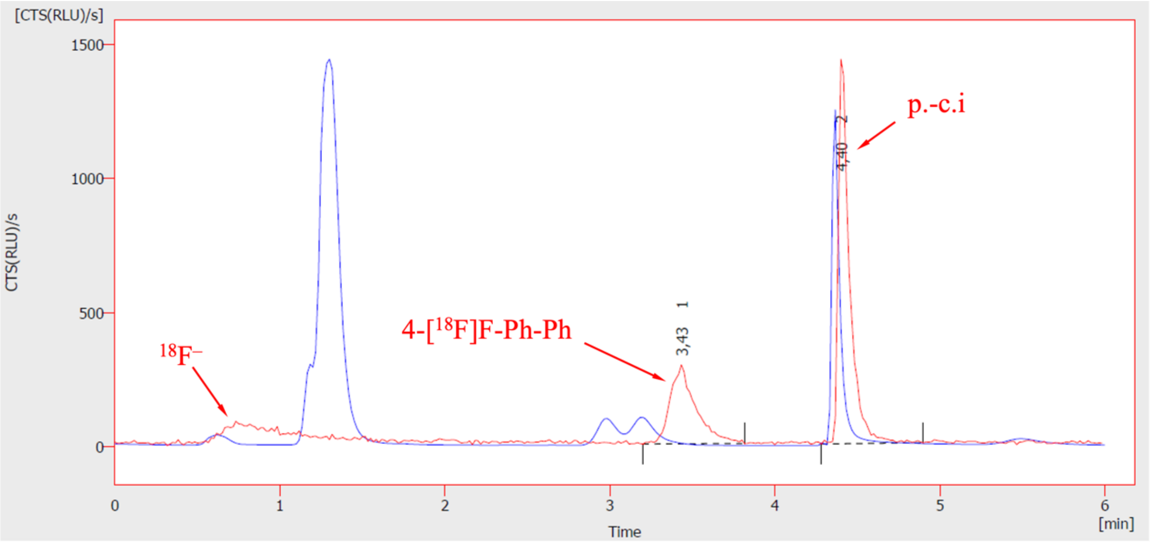

Result Table (Uncal - Data\alc-enhanced_756_12_05_2022_[4 DMI]biphenyl-B(OH)2_110°C_40min - HERM)

	Reten. Time [min]	Area [CTS(RLU)/s.s]	Height [CTS(RLU)/s]	Area [%]
1	3,433	3195,500	295,622	31,6
2	4,400	6913,000	1435,243	68,4
	Total	10108,500	1730,865	100,0

Figure S30: HPLC traces of crude 4-[^{18}F]F-Ph-Ph prepared from **1** using Cu(4-PhPy)$_4$(ClO$_4$)$_2$ as mediator in *n*BuOH/DMI at 110 °C for 40 min. Blue trace: UV, λ = 254 nm; red trace: radioactivity. Abbreviation: p.-c.i – post-column injection.

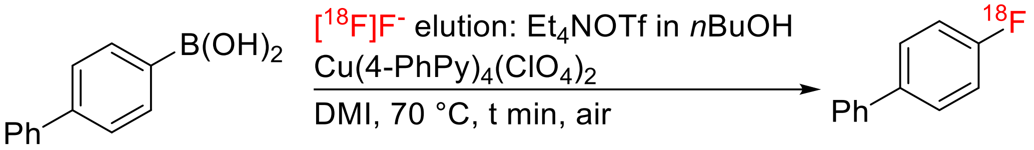

1

4-[^{18}F]F-Ph-Ph

Table S39: Screening of reaction times at 70 °C.

Entry	t [min]	RCC [%]
1	5	35 ± 5
2	10	54 ± 5
3	20	75 ± 3
4	30	82 ± 1
5	40	80 ± 5

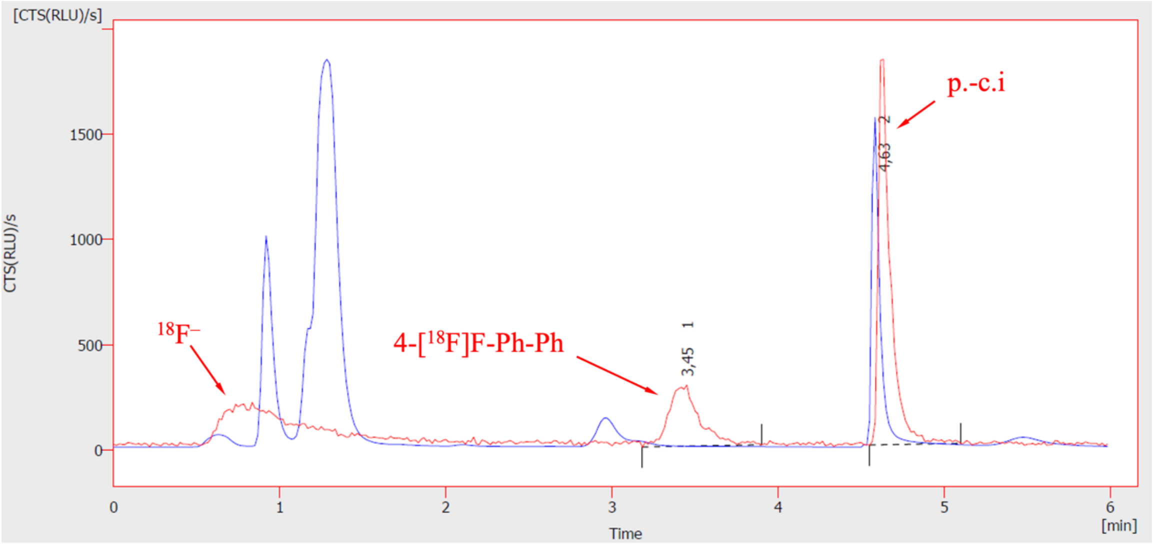

Result Table (Uncal - Data|alc-enhanced_762_12_05_2022_[4 DMI]biphenyl-B(OH)2_70°C_5min - HERM)

	Reten. Time [min]	Area [CTS(RLU)/s.s]	Height [CTS(RLU)/s]	Area [%]
1	3,450	3775,500	290,907	28,7
2	4,633	9400,500	1829,939	71,3
	Total	13176,000	2120,846	100,0

Figure S31: HPLC traces of crude 4-[^{18}F]F-Ph-Ph prepared from **1** using Cu(4-PhPy)$_4$(ClO$_4$)$_2$ as mediator in *n*BuOH/DMI at 70 °C for 5 min. Blue trace: UV, λ = 254 nm; red trace: radioactivity. Abbreviation: p.-c.i – post-column injection.

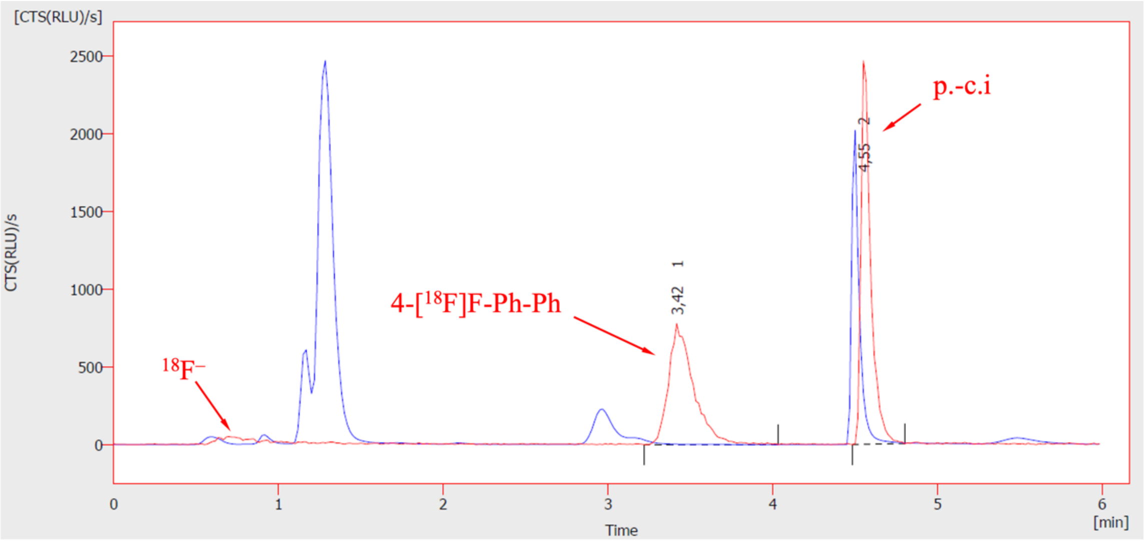

Result Table (Uncal - Data|alc-enhanced_675_08_04_2022_[4 DMI]biphenyl-B(OH)2_70_20min - HERM)

	Reten. Time [min]	Area [CTS(RLU)/s.s]	Height [CTS(RLU)/s]	Area [%]
1	3,417	8122,000	778,000	44,2
2	4,550	10250,500	2469,947	55,8
	Total	18372,500	3247,947	100,0

Figure S32: HPLC traces of crude 4-[^{18}F]F-Ph-Ph prepared from **1** using Cu(4-PhPy)$_4$(ClO$_4$)$_2$ as mediator in *n*BuOH/DMI at 70 °C for 20 min. Blue trace: UV, λ = 254 nm; red trace: radioactivity. Abbreviation: p.-c.i – post-column injection.

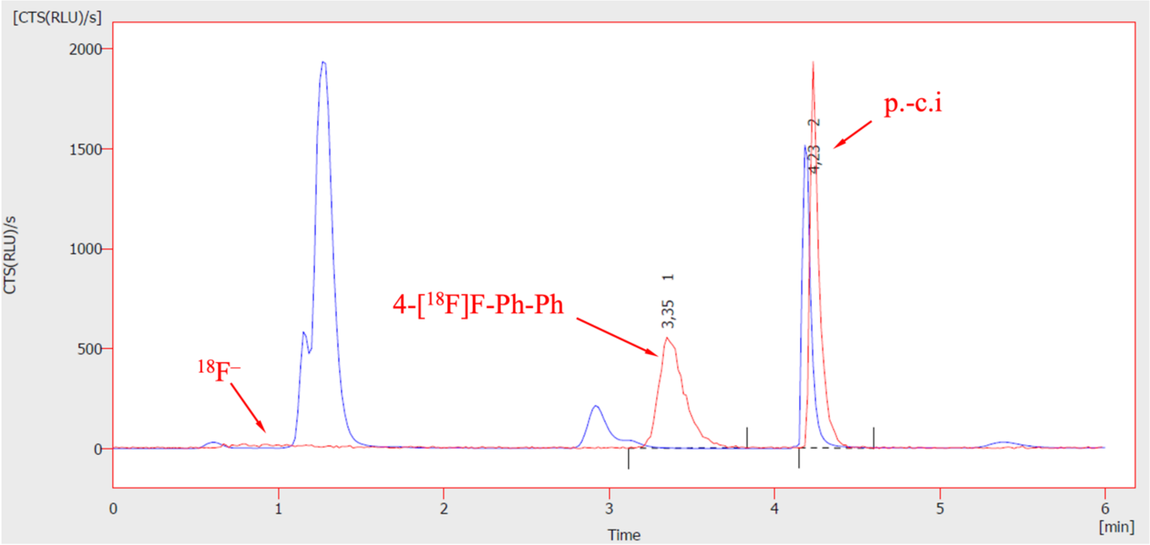

Result Table (Uncal - Data|alc-enhanced_712_14_04_2022_[4 DMI]biphenyl-B(OH)2_70°C_40min - HERM)

	Reten. Time [min]	Area [CTS(RLU)/s.s]	Height [CTS(RLU)/s]	Area [%]
1	3,350	6062,000	553,349	44,4
2	4,233	7591,000	1931,370	55,6
	Total	13653,000	2484,719	100,0

Figure S33: HPLC traces of crude 4-[^{18}F]F-Ph-Ph prepared from **1** using Cu(4-PhPy)$_4$(ClO$_4$)$_2$ as mediator in *n*BuOH/DMI at 70 °C for 30 min. Blue trace: UV, λ = 254 nm; red trace: radioactivity. Abbreviation: p.-c.i – post-column injection.

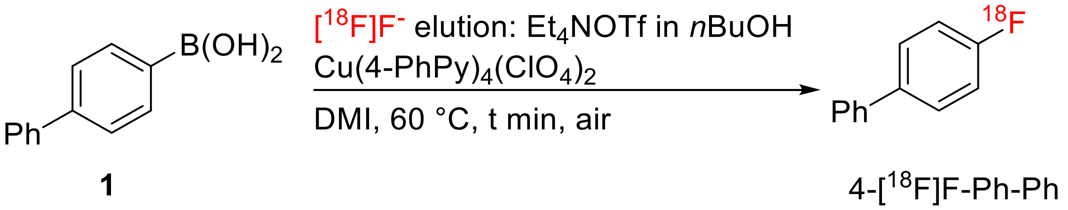

Table S40: Screening of reaction times at 60 °C.

Entry	t [min]	RCC [%]
1	5	13 ± 6
2	10	37 ± 7
3	20	40 ± 15
4	30	66 ± 8
5	40	69 ± 8

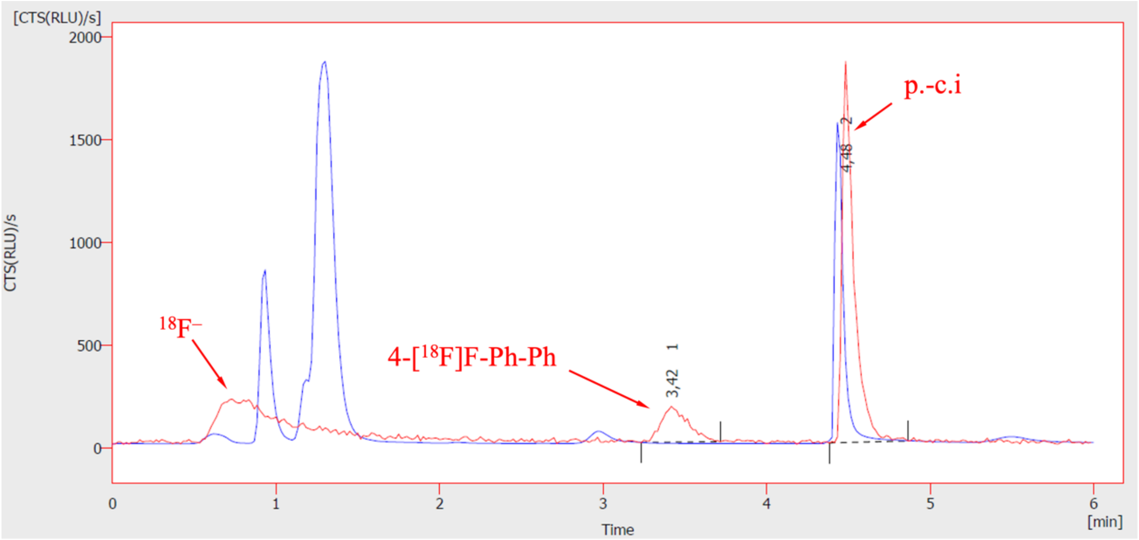

Result Table (Uncal - Data|alc-enhanced_760_12_05_2022_[4 DMI]biphenyl-B(OH)2_60°C_5min - HERM)

	Reten. Time [min]	Area [CTS(RLU)/s.s]	Height [CTS(RLU)/s]	Area [%]
1	3,417	1993,000	174,483	17,4
2	4,483	9441,500	1854,138	82,6
	Total	11434,500	2028,621	100,0

Figure S34: HPLC traces of crude 4-[^{18}F]F-Ph-Ph prepared from **1** using Cu(4-PhPy)$_4$(ClO$_4$)$_2$ as mediator in *n*BuOH/DMI at 60 °C for 5 min. Blue trace: UV, λ = 254 nm; red trace: radioactivity. Abbreviation: p.-c.i – post-column injection.

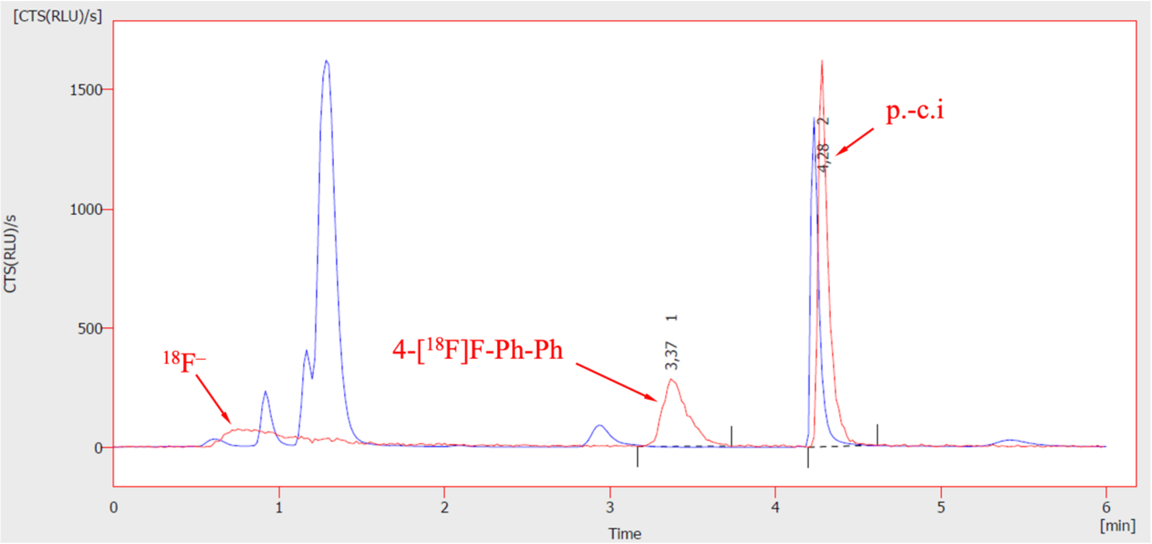

Result Table (Uncal - Data|alc-enhanced_687_08_04_2022_[4 DMI]biphenyl-B(OH)2_60°C_20min - HERM)

	Reten. Time [min]	Area [CTS(RLU)/s.s]	Height [CTS(RLU)/s]	Area [%]
1	3,367	3005,000	283,647	29,9
2	4,283	7052,000	1621,400	70,1
	Total	10057,000	1905,047	100,0

Figure S35: HPLC traces of crude 4-[^{18}F]F-Ph-Ph prepared from **1** using Cu(4-PhPy)$_4$(ClO$_4$)$_2$ as mediator in *n*BuOH/DMI at 60 °C for 20 min. Blue trace: UV, λ = 254 nm; red trace: radioactivity. Abbreviation: p.-c.i – post-column injection.

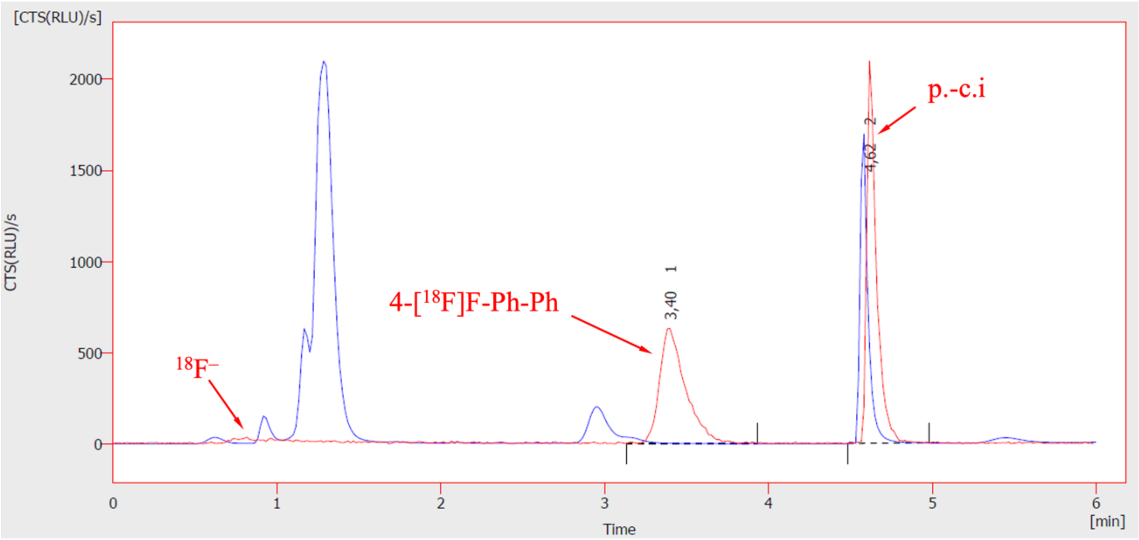

Result Table (Uncal - Data|alc-enhanced_710_14_04_2022_[4 DMI]biphenyl-B(OH)2_60°C_40min - HERM)

	Reten. Time [min]	Area [CTS(RLU)/s.s]	Height [CTS(RLU)/s]	Area [%]
1	3,400	7010,000	630,333	44,6
2	4,617	8693,000	2096,200	55,4
	Total	15703,000	2726,533	100,0

Figure S36: HPLC trace of crude 4-[^{18}F]F-Ph-Ph prepared from **1** using Cu(4-PhPy)$_4$(ClO$_4$)$_2$ as mediator in *n*BuOH/DMI at 60 °C for 40 min. Blue trace: UV, λ = 254 nm; red trace: radioactivity. Abbreviation: p.-c.i – post-column injection.

3.8 Radiolabeling of model stannyl precursors 5 and 6

Screening of radiofluorination mediator

Radiolabeling of stannyl precursors **5** and **6** was performed according to GP6-A in *n*BuOH/DMI (n = 3). RCCs were determined by radio-HPLC as described above.

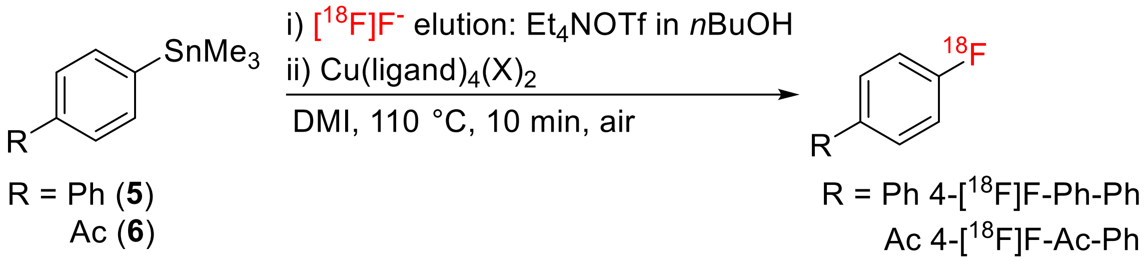

Table S41: Screening of radiofluorination mediators.

Entry	Ligand	X	RCC [%]	
			**5**	**6**
1	Py	OTf	27 ± 8	22 ± 5
2	4-PhPy	ClO_4	26 ± 8	44 ± 3
3	3,4-Me_2Py	OTf	25 ± 6	31 ± 9
4	3,4-Me_2Py	ClO_4	24 ± 1	41 ± 5
5	Impdz	OTf	16 ± 2	26 ± 3
6	Impdz	ClO_4	28 ± 6	41 ± 3

Optimization of radiolabeling of stannyl precursors 5 and 6 with regard to the presence/absence of *n*BuOH and reaction atmosphere

Radiosyntheses were performed according to GP6-B, GP7-A and GP7-B with Cu(4-PhPy)$_4$(ClO_4)$_2$ in DMI (n=3). RCCs were determined by radio-HPLC as described above. Representative HPLC chromatograms of the crude radiolabeled products are shown in Figure **S37–S42**.

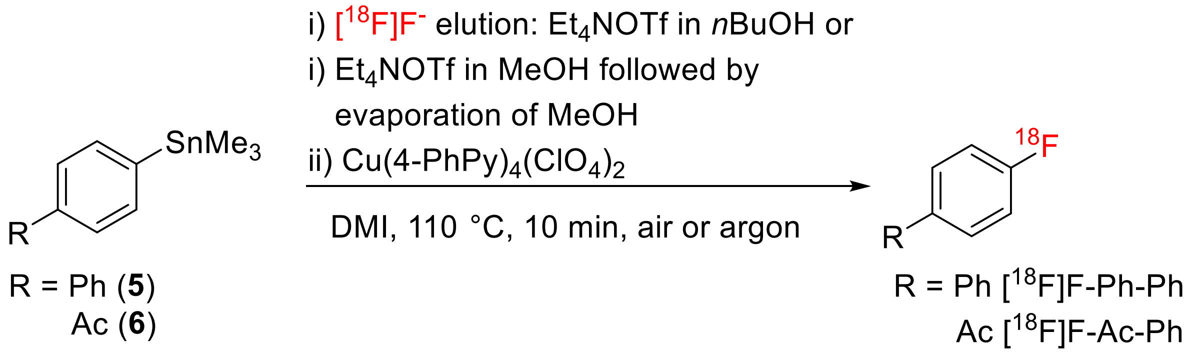

Table S42: Influence of *n*BuOH and reaction atmosphere on radiolabeling of **5** and **6**.

Entry	Precursor	Elution	Conditions	RCC [%]
1	**5**	*n*BuOH	argon	22 ± 6
2		MeOH	air	38 ± 2
3		MeOH	argon	57 ± 3
4	**6**	*n*BuOH	argon	33 ± 11
5		MeOH	air	45 ± 3
6		MeOH	argon	47 ± 6

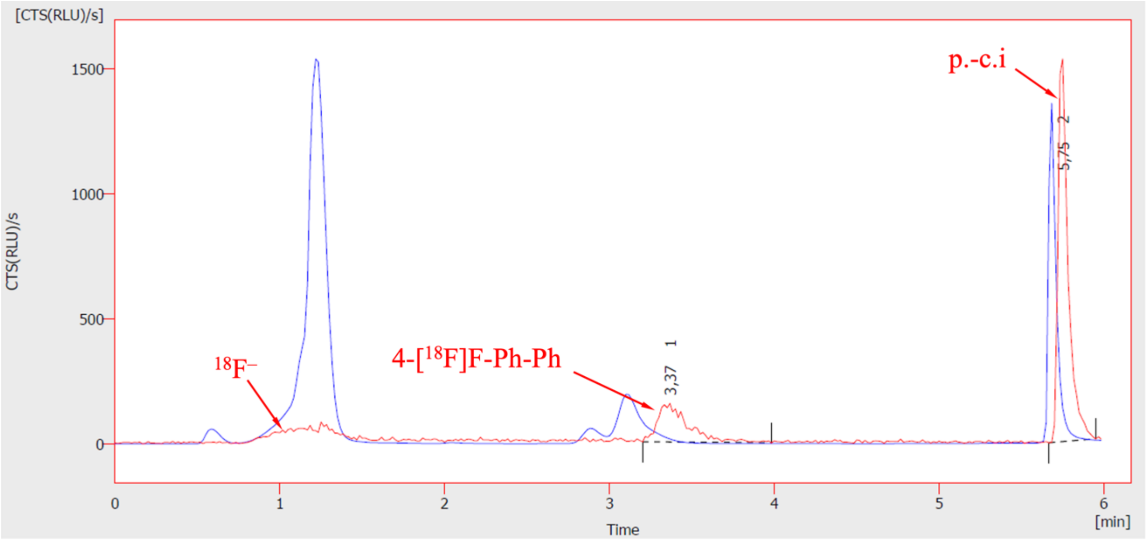

Result Table (Uncal - Data|alc-enhanced_372_24_11_2021_[4 DMI]_biphenyl-SnMe3_nBuOH_argon_110 - HERM)

	Reten. Time [min]	Area [CTS(RLU)/s.s]	Height [CTS(RLU)/s]	Area [%]
1	3,367	2017,000	154,851	23,4
2	5,750	6604,000	1529,294	76,6
	Total	8621,000	1684,145	100,0

Figure S37: HPLC traces of crude 4-[^{18}F]F-Ph-Ph prepared from **5** using Cu(4-PhPy)$_4$(ClO$_4$)$_2$ as mediator in *n*BuOH/DMI at 110 °C for 10 min under Ar. Blue trace: UV, λ = 254 nm; red trace: radioactivity. Abbreviation: p.-c.i – post-column injection.

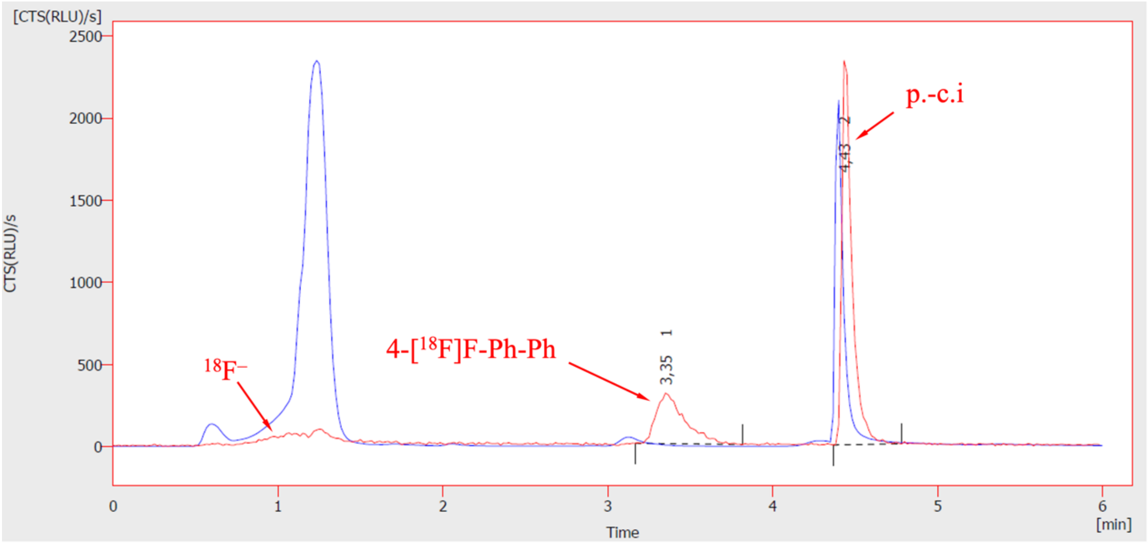

Result Table (Uncal - Data|alc-enhanced_369_24_11_2021_[4 DMI]_biphenyl-SnMe3_MeOH_air_110 - HERM)

	Reten. Time [min]	Area [CTS(RLU)/s.s]	Height [CTS(RLU)/s]	Area [%]
1	3,350	3746,000	308,256	26,9
2	4,433	10203,500	2340,880	73,1
	Total	13949,500	2649,136	100,0

Figure S38: HPLC traces of crude 4-[^{18}F]F-Ph-Ph prepared from **5** using Cu(4-PhPy)$_4$(ClO$_4$)$_2$ as mediator in DMI at 110 °C for 10 min under air. Blue trace: UV, λ = 254 nm; red trace: radioactivity. Abbreviation: p.-c.i – post-column injection.

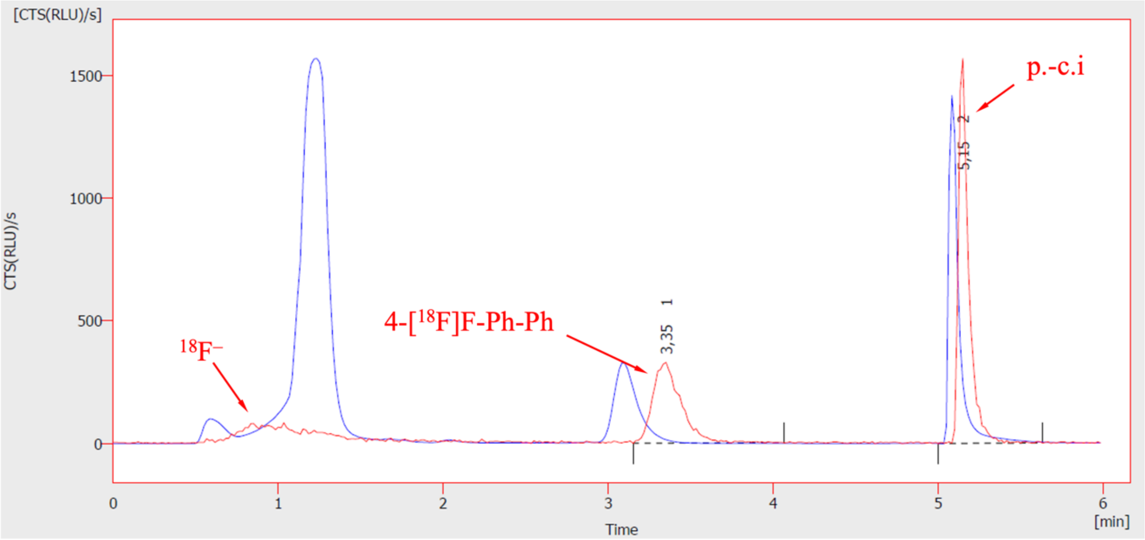

Result Table (Uncal - Data\alc-enhanced_332_03_11_2021_[4 DMI]_biphenyl-Sn(Me)3_argon - HERM)

	Reten. Time [min]	Area [CTS(RLU)/s.s]	Height [CTS(RLU)/s]	Area [%]
1	3,350	3970,000	329,564	36,5
2	5,150	6914,000	1569,526	63,5
	Total	10884,000	1899,090	100,0

Figure S39: HPLC traces of crude 4-[^{18}F]F-Ph-Ph prepared from **5** using Cu(4-PhPy)$_4$(ClO$_4$)$_2$ as mediator in DMI at 110 °C for 10 min under Ar. Blue trace: UV, λ = 254 nm; red trace: radioactivity. Abbreviation: p.-c.i – post-column injection.

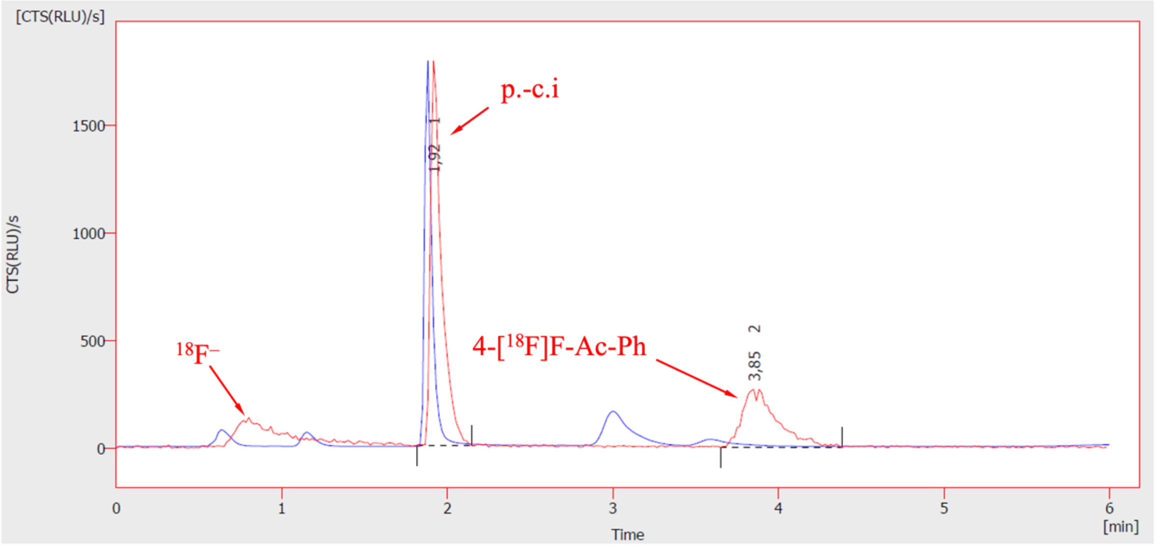

Result Table (Uncal - Data\alc-enhanced_351_16_11_2021_[4 DMI]_4-acetylphenyl-SnMe3_argon_nBuOH - HERM)

	Reten. Time [min]	Area [CTS(RLU)/s.s]	Height [CTS(RLU)/s]	Area [%]
1	1,917	8384,000	1788,300	67,7
2	3,850	4005,000	269,273	32,3
	Total	12389,000	2057,573	100,0

Figure S40: HPLC traces of crude 4-[^{18}F]F-Ac-Ph prepared from **6** using Cu(4-PhPy)$_4$(ClO$_4$)$_2$ as mediator in nBuOH/DMI at 110 °C for 10 min under argon. Blue trace: UV, λ = 254 nm; red trace: radioactivity. Abbreviation: p.-c.i – post-column injection.

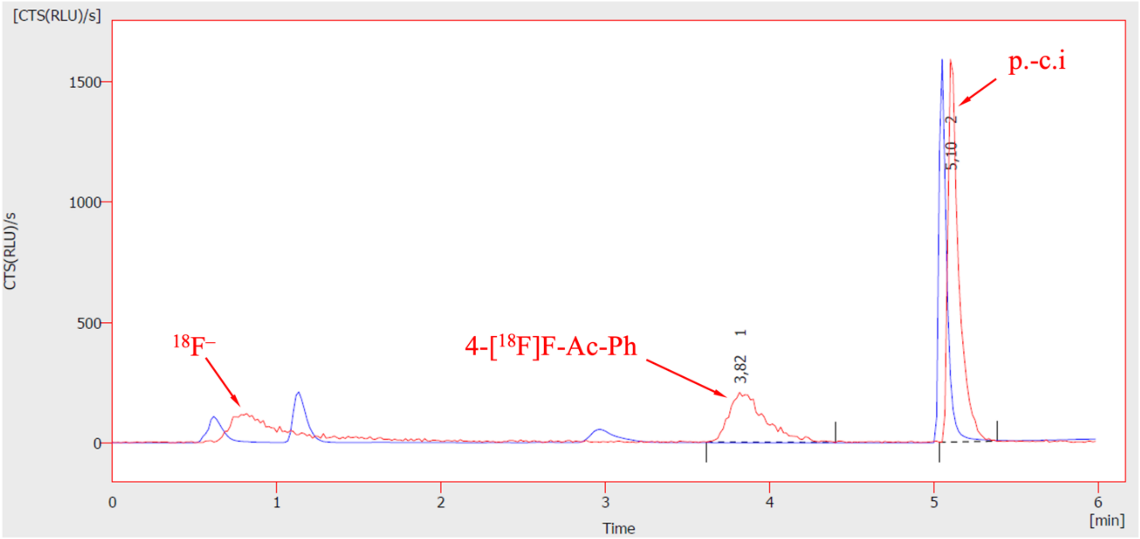

Result Table (Uncal - Data|alc-enhanced_344_15_11_2021_[4 DMI]_4-acetylphenyl-SnMe3_air_MeOH_evap - HERM)

	Reten. Time [min]	Area [CTS(RLU)/s.s]	Height [CTS(RLU)/s]	Area [%]
1	3,817	3147,000	207,511	29,9
2	5,100	7390,000	1590,238	70,1
	Total	10537,000	1797,749	100,0

Figure S41: HPLC traces of crude 4-[^{18}F]F-Ac-Ph prepared from **6** using Cu(4-PhPy)$_4$(ClO$_4$)$_2$ as mediator in DMI at 110 °C for 10 min under air. Blue trace: UV, λ = 254 nm; red trace: radioactivity. Abbreviation: p.-c.i – post-column injection.

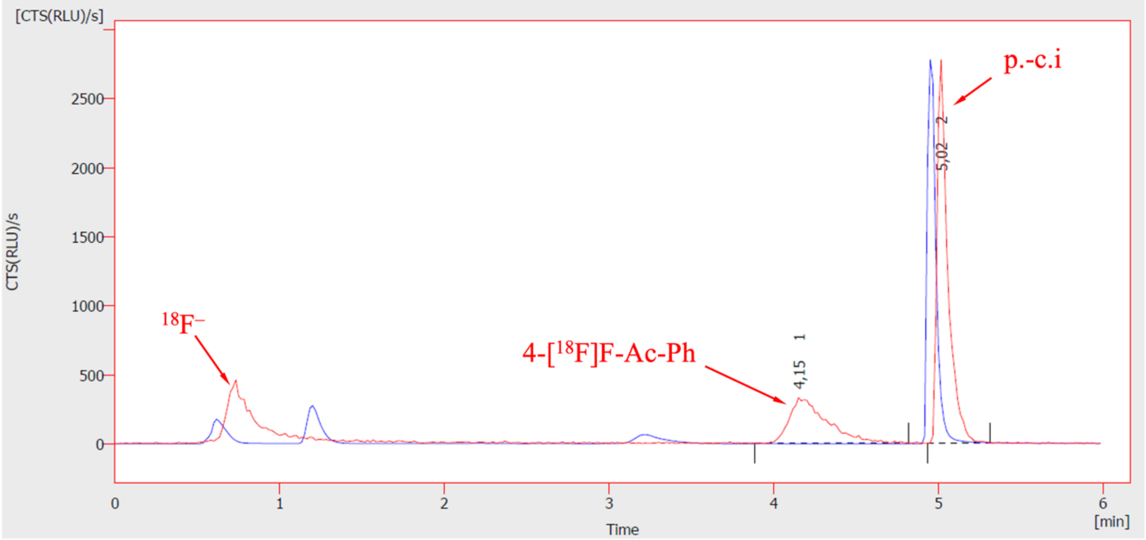

Result Table (Uncal - Data|alc-enhanced_336_05_11_2021_[4 DMI]_4-acetylphenyl-Sn(Me)3_argon - HERM)

	Reten. Time [min]	Area [CTS(RLU)/s.s]	Height [CTS(RLU)/s]	Area [%]
1	4,150	5188,000	331,143	29,0
2	5,017	12719,500	2778,783	71,0
	Total	17907,500	3109,925	100,0

Figure S42: HPLC traces of crude 4-[^{18}F]F-Ac-Ph prepared from **6** using Cu(4-PhPy)$_4$(ClO$_4$)$_2$ as mediator in DMI at 110 °C for 10 min under argon. Blue trace: UV, λ = 254 nm; red trace: radioactivity. Abbreviation: p.-c.i. – post-column injection.

Radiolabeling of stannanes 5 and 6 under optimized conditions

Radiolabeling of stannyl precursors **5** and **6** was performed according to GP7-B with Cu(4-PhPy)$_4$(ClO$_4$)$_2$ in DMI at 90 °C for 10 min (n=3). RCCs were determined by radio-HPLC as described above. Representative radio-HPLC chromatograms of radiolabeled products are shown in Figure **S43** and Figure **S44**.

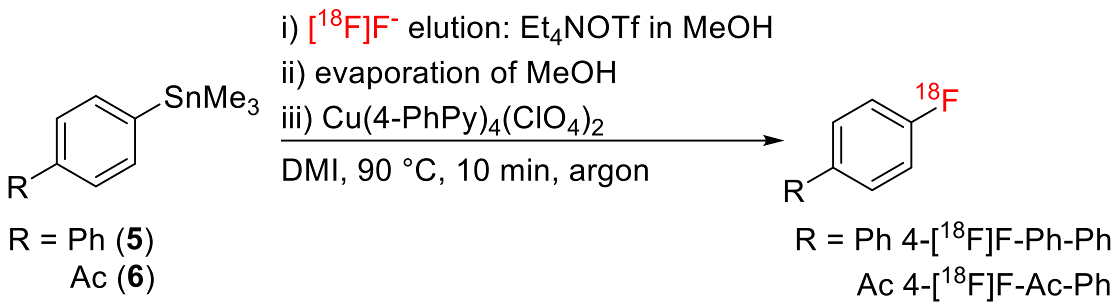

Table S43: Radiolabeling of stannyl precursors under optimized conditions.

Entry	Precursor	RCC [%]
1	**5**	60 ± 6
2	**6**	63 ± 10

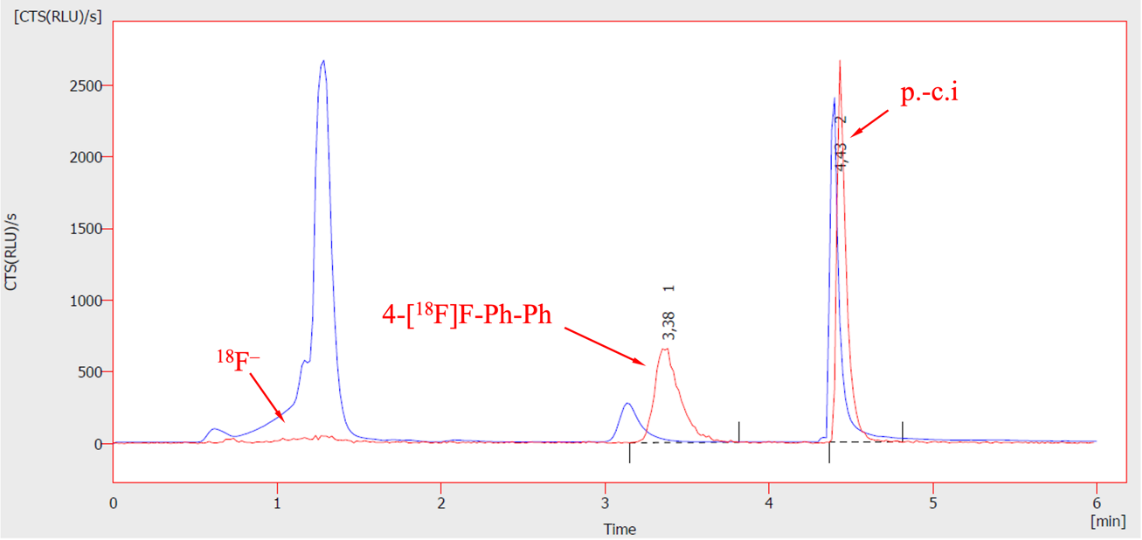

Result Table (Uncal - Data|alc-enhanced_576_02_03_2022_[4 DMI]biphenyl-SnMe3_90°C_argon_MeOH - HERM)

	Reten. Time [min]	Area [CTS(RLU)/s.s]	Height [CTS(RLU)/s]	Area [%]
1	3,383	7179,000	656,950	40,4
2	4,433	10597,000	2665,000	59,6
	Total	17776,000	3321,950	100,0

Figure S43: HPLC traces of crude 4-[^{18}F]F-Ph-Ph prepared from **5** using Cu(4-PhPy)$_4$(ClO$_4$)$_2$ as mediator in DMI at 90 °C for 10 min under argon. Blue trace: UV, λ = 254 nm; red trace: radioactivity. Abbreviation: p.-c.i. – post-column injection.

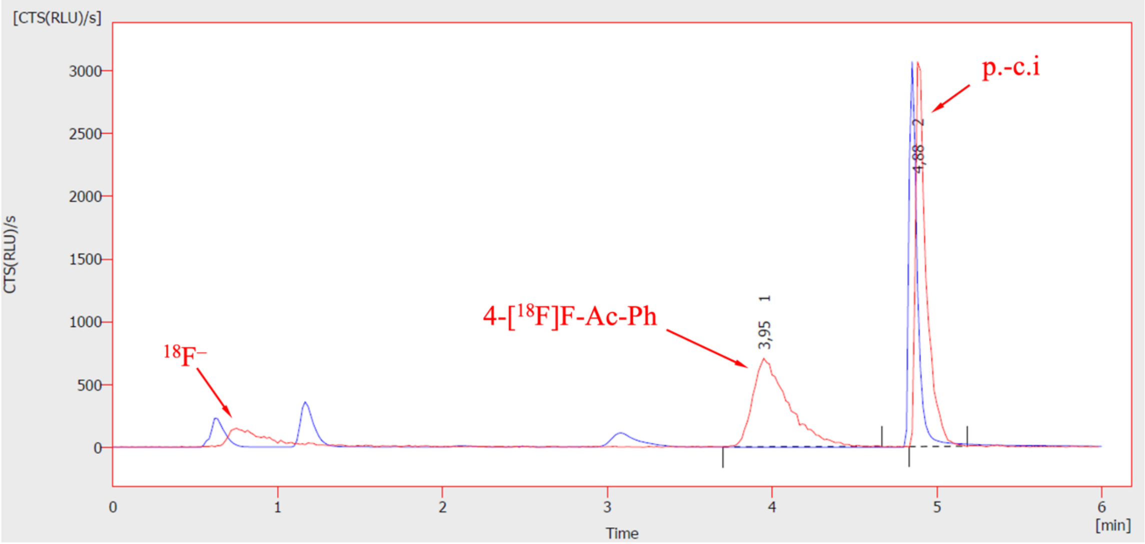

Result Table (Uncal - Data\alc-enhanced_353_19_11_2021_[4 DMI]_4-acetylphenyl-SnMe3_90_MeOH_argon - HERM)

	Reten. Time [min]	Area [CTS(RLU)/s.s]	Height [CTS(RLU)/s]	Area [%]
1	3,950	10640,000	705,224	43,2
2	4,883	14008,500	3067,286	56,8
	Total	24648,500	3772,510	100,0

Figure S44: HPLC traces of crude 4-[^{18}F]F-Ac-Ph prepared from **6** using Cu(4-PhPy)$_4$(ClO$_4$)$_2$ as mediator in DMI at 90 °C for 10 min under argon. Blue trace: UV, λ = 254 nm; red trace: radioactivity. Abbreviation: p.-c.i. – post-column injection.

Statistical evaluation of the influence of *n*BuOH, reaction atmosphere and temperature on radiolabeling of stannyl precursors 5 and 6

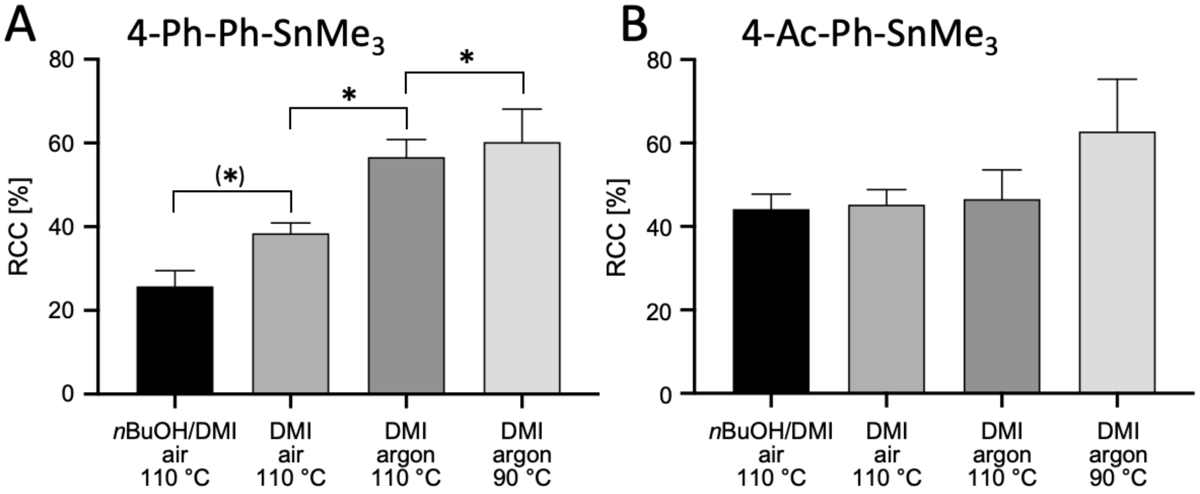

Figure S45: Statistical evaluation of the influence of *n*BuOH, reaction atmosphere and temperature on radiolabeling of stannyl precursors **5** and **6**. Solvent: *n*BuOH/DMI or DMI; radiolabeling mediator: Cu(4-PhPy)$_4$(ClO$_4$)$_2$. Statistics: 1-way ANOVA followed by Tukey's multiple comparison test. * $p<0.05$; (*) $p=0.0552$

Table S44: Comparison of RCCs (mean ± standard deviation in %) under different reaction conditions using 1-way ANOVA followed by Tukey's multiple comparison test (n=3 per group). Solvent: DMI; radiolabeling mediator: $Cu(4\text{-}PhPy)_4(ClO_4)_2$. p-values refer to the respective previous condition.

		atmosph.	temp.	RCCs [%]	
4-Ph-Ph-$SnMe_3$	nBuOH	air	110 °C	25.7 ± 3.69	
F(3,8)=31.43	MeOH	air	110 °C	38.46 ± 2.45	p=0.0552
p<0.0001	MeOH	argon	110 °C	56.60 ± 4.25	p=0.0091*
	MeOH	argon	90 °C	60.22 ± 7.85	p=0.0031*
4-Ac-Ph-$SnMe_3$	nBuOH	air	110 °C	44.15 ± 3.60	
F(3,8)=31.43	MeOH	air	110 °C	45.25 ± 3.58	p=0.9979
p<0.0001	MeOH	argon	110 °C	46.54 ± 7.01	p=0.9966
	MeOH	argon	90 °C	62.80 ± 12.53	p=0.1145

3.9 Radiosynthesis of [^{18}F]R91150

[^{18}F]F^- (30–500 MBq) was eluted from the QMA cartridge with a solution of Et_4NOTf (1 mg, 3.6 µmol) in anhydrous MeOH (500 µL). MeOH was evaporated at 60 °C under reduced pressure in a stream of argon. The reactor was opened, a solution of **7** (6.2 mg, 10 µmol) and $Cu(4\text{-}PhPy)_4(ClO_4)_2$ (8.8 mg, 10 µmol) in anhydrous DMI/*n*BuOH (1.2 mL, 2:1) was added and the reaction mixture was heated at 110 °C for 10 min. That followed, the reaction mixture was diluted with H_2O (18 mL) and loaded onto a preconditioned (2 mL EtOH, 10 mL H_2O) HLB short cartridge (300 mg). The cartridge was washed with H_2O (5 mL) and the radiolabeled intermediate was eluted with acetone (2 mL). The acetone was evaporated at 80 °C under reduced pressure in a stream of argon, 6 M HCl (500 µL) was added to the residue and the mixture was heated at 110 °C for 10 min. Thereafter, 6 M NaOH (350 µL) followed by 0.1% TFA (600 µL) were added to the mixture, and the resulting solution was loaded onto a preparative HPLC column. The product fraction was eluted at 7.5–9.0 min.

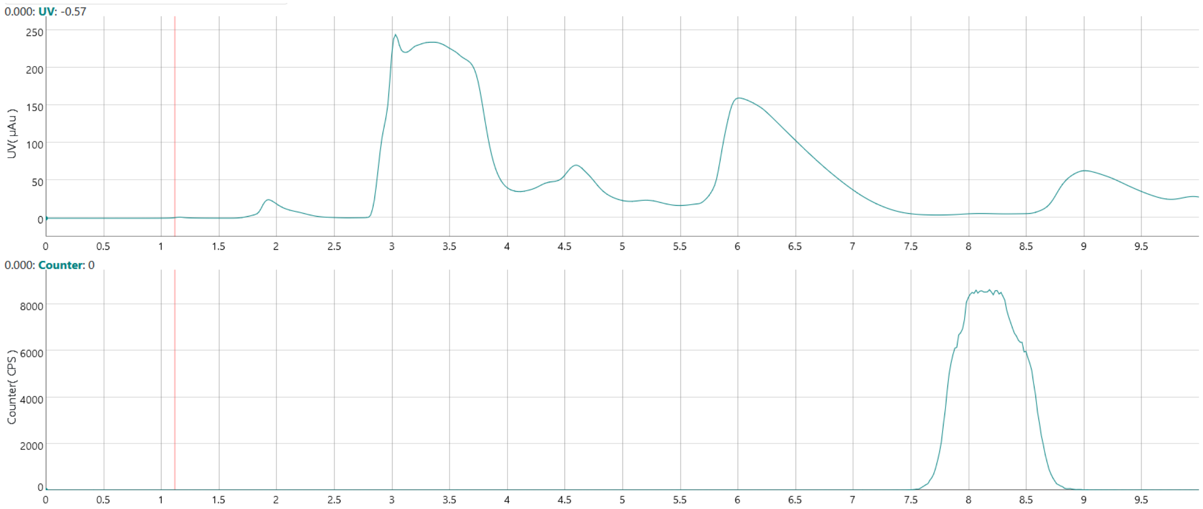

Figure S46: Purification of [^{18}F]R91150 by preparative HPLC (top: UV trace, λ=254 nm; bottom: radioactivity trace).

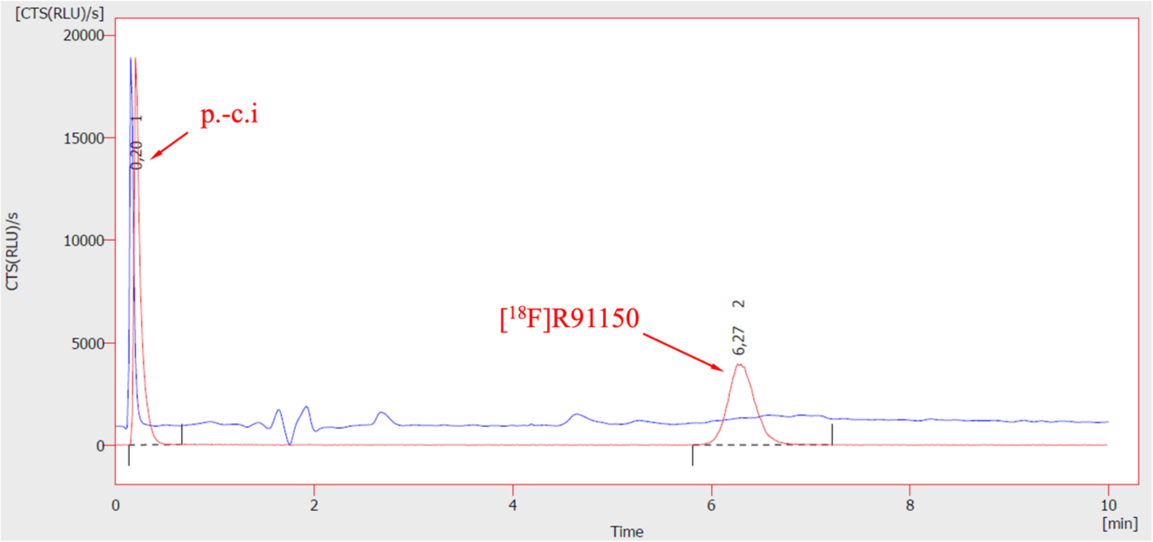

Result Table (Uncal - Data\alc-enhanced_890_08_08_2022_[4 DMI][18F]R91150-Bpin_MeOH_elution_hplc - HERM)

	Reten. Time [min]	Area [CTS(RLU)/s.s]	Height [CTS(RLU)/s]	Area [%]
1	0,200	84242,000	18896,875	52,9
2	6,267	74967,000	3942,786	47,1
	Total	159209,000	22839,661	100,0

Figure S47: HPLC chromatogram of purified [^{18}F]R91150 for quality control. Blue trace: UV channel, λ=254 nm; red trace: radioactivity channel. Abbreviation: p.-c.i. – post-column injection.

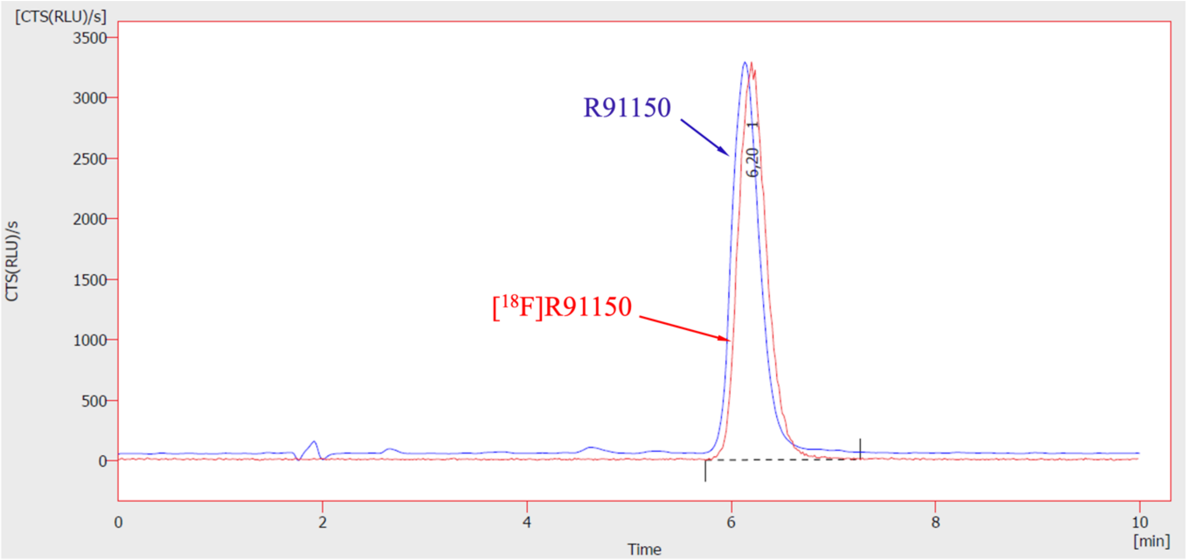

Result Table (Uncal - Data\alc-enhanced_891_08_08_2022_[4 DMI][18F]R91150-Bpin_MeOH_elution_spiked - HERM)

	Reten. Time [min]	Area [CTS(RLU)/s.s]	Height [CTS(RLU)/s]	Area [%]
1	6,200	63849,500	3291,110	100,0
	Total	63849,500	3291,110	100,0

Figure S48: HPLC chromatogram of purified [^{18}F]R91150 spiked with the non-radioactive reference compound. Blue trace: UV channel, λ=254 nm; red trace: radioactivity channel.

3.10 Radiosynthesis of [^{18}F]ALX5407

[^{18}F]F^- was eluted from the QMA cartridge directly into a solution of **8** (8.8 mg, 10 µmol) and Cu(4-PhPy)$_4$(ClO_4)$_2$ (8.8 mg, 10 µmol) in anhydrous DMI (800 µL) with a solution of Et_4NOTf (1 mg) in anhydrous *n*BuOH (400 µL), and the reaction mixture was stirred for 10 min at 110 °C. That followed, 6 M NaOH (250 µL) was added and the mixture was stirred for another 10 min at 110 °C. The reaction mixture was cooled to ambient temperature, diluted with 6 M HCl (400 µL) followed by H_2O (20 mL) and loaded onto a Strata-X cartridge. The cartridge was washed with H_2O (5 mL) and the crude [^{18}F]ALX5407 was eluted with MeCN (500 µL). The resulting solution was diluted with 0.1% AcOH (1 mL) and loaded onto a preparative HPLC column. The product fraction, which eluted at 20.5–22.5 min, was taken up into H_2O (100 mL) and loaded onto a Strata-X cartridge. The cartridge was washed with H_2O (5 mL) and dried for 2 min in a flow of argon. [^{18}F]ALX5407 was eluted with EtOH (800 µL), and the solvent was removed under reduced pressure at 70 °C. The residue was taken up into a sterile filtered 1% Tween 80 solution (500 µL) to afford [^{18}F]ALX5407 in a ready-to-use form.

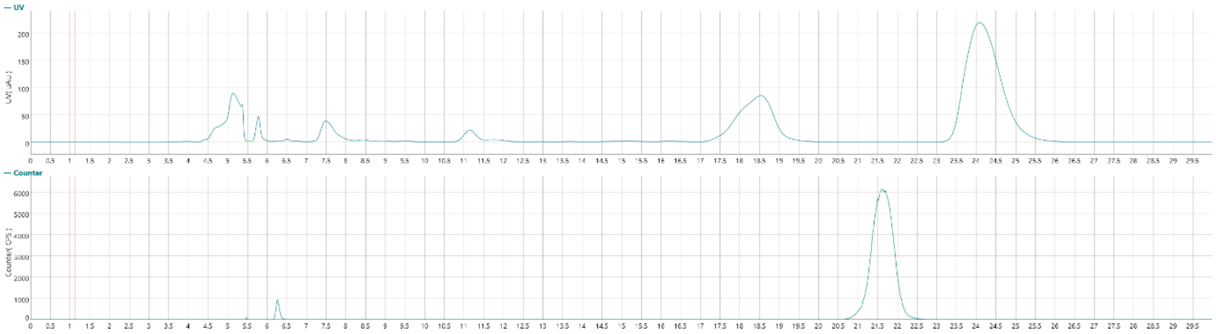

Figure S49: Purification of [^{18}F]ALX5407 by preparative HPLC (top: UV trace, λ=254 nm; bottom: radioactivity trace).

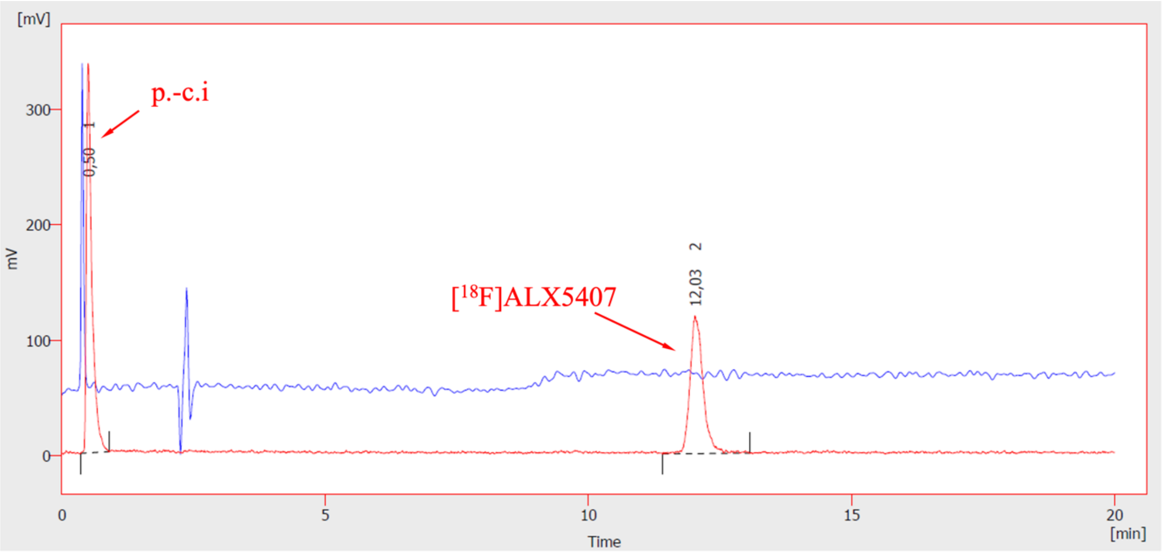

Result Table (Uncal - Data|alc-enhanced_205_23_06_2021_[4 DMI]_[18F]ALX5407_radio_purity - IF2/IFU2.1:Channel 1)

	Reten. Time [min]	Area [mV.s]	Height [mV]	Area [%]
1	0,498	2455,282	336,988	54,1
2	12,025	2081,980	119,362	45,9
	Total	4537,262	456,350	100,0

Figure S50: HPLC chromatogram of purified [^{18}F]ALX5407 for quality control. Blue trace: UV channel, λ=254 nm; red trace: radioactivity channel. Abbreviation: p.-c.i. – post-column injection.

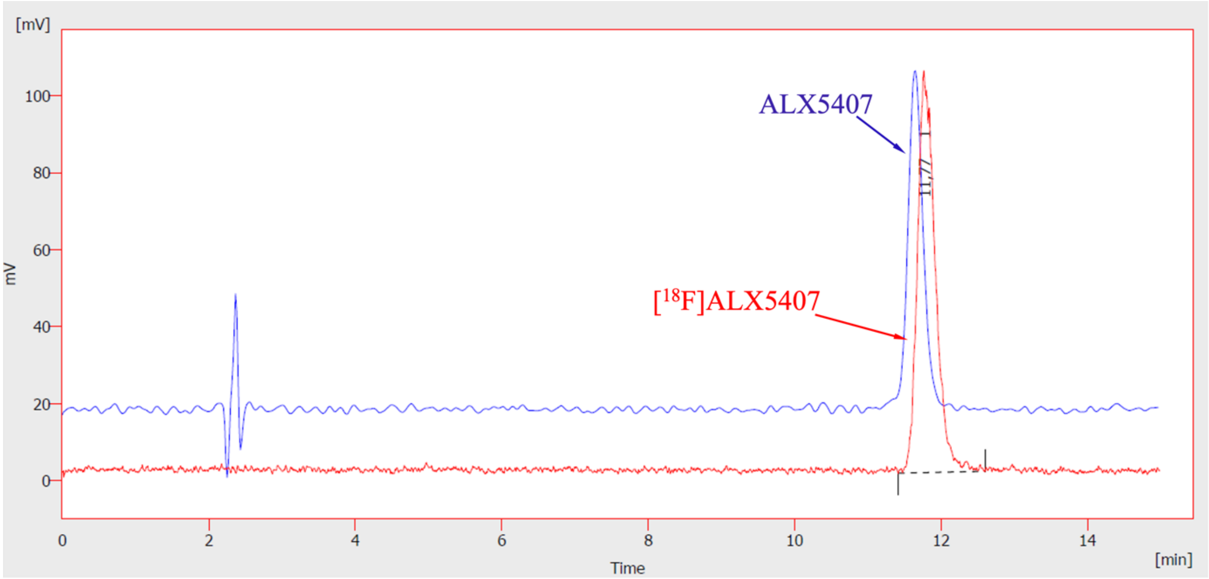

Result Table (Uncal - Data|alc-enhanced_206_23_06_2021_[4 DMI]_[18F]ALX5407_gespiked - IF2/IFU2.1:Channel 1)

	Reten. Time [min]	Area [mV.s]	Height [mV]	Area [%]
1	11,767	1766,430	104,502	100,0
	Total	1766,430	104,502	100,0

Figure S51: HPLC chromatogram of purified [^{18}F]ALX5407 spiked with the non-radioactive reference compound. Blue trace: UV channel, λ=254 nm; red trace: radioactivity channel.

3.11 Radiosynthesis of [^{18}F]MNI1126

[^{18}F]F^- was eluted from the QMA cartridge into a reaction vial with a solution of Et_4NOTf (1 mg, 3.6 µmol) in anhydrous MeOH (500 µL) and all volatiles were removed at 80 °C. Thereafter, a solution of **9** (10 µmol) and copper complex (10 µmol) in anhydrous DMI (800 µL) was added, and the reaction mixture was stirred for 10 min at 110 °C (n=3). RCCs were determined by radio-HPLC.

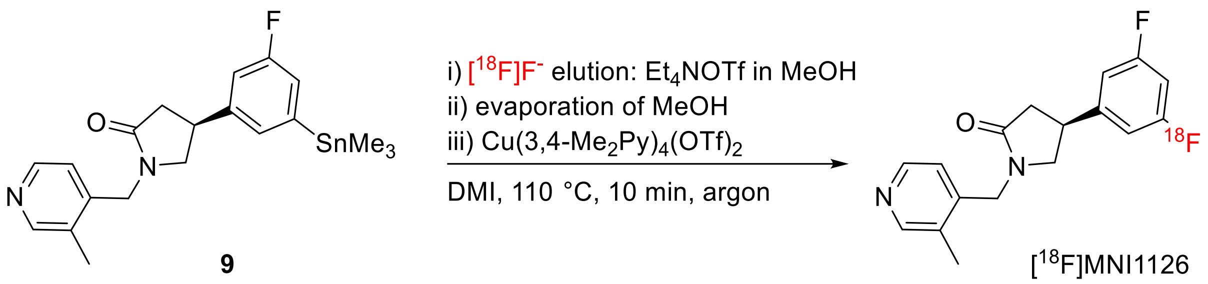

Table S45: Screening of radiolabeling mediator

entry	Cu(II) complex	RCC [%]
1	$Cu(Py)_4(OTf)_2$	26 ± 15
2	$Cu(4\text{-}PhPy)_4(ClO_4)_2$	34 ± 10
3	$Cu(3,4\text{-}Me_2Py)_4(OTf)_2$	47 ± 5
4	$Cu(3,4\text{-}Me_2Py)_4(ClO_4)_2$	37 ± 7

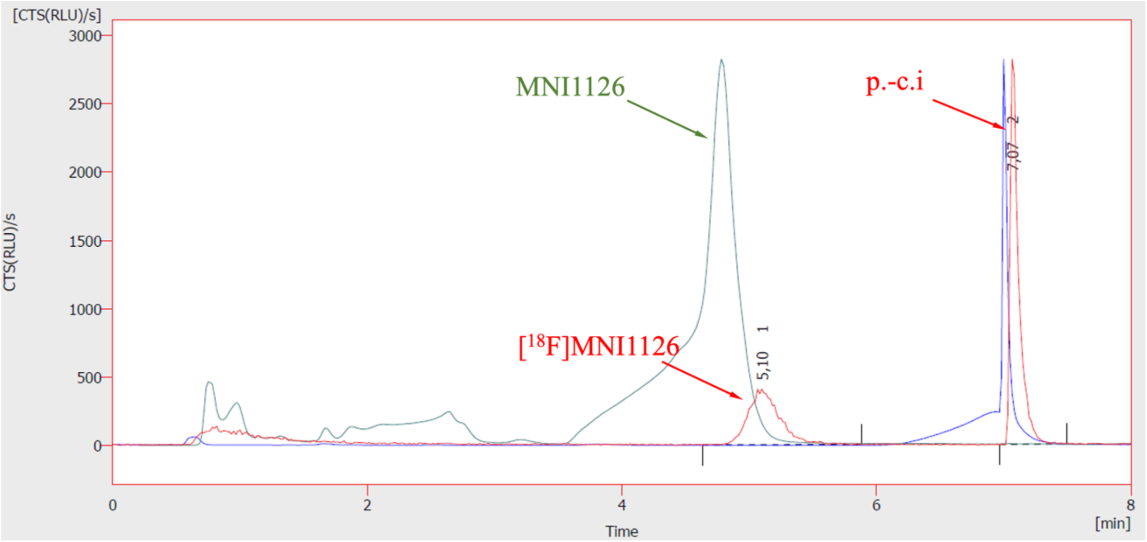

Result Table (Uncal - Data|alc-enhanced_844_14_07_2022_[4 DMI]MNI1126-SnMe3 - HERM)

	Reten. Time [min]	Area [CTS(RLU)/s.s]	Height [CTS(RLU)/s]	Area [%]	RCC [%]
1	5,100	7116,500	408,627	34,6	100,000
2	7,067	13465,000	2823,500	65,4	189,208
	Total	20581,500	3232,127	100,0	289,208

Figure S52: Overlay of HPLC traces of crude [^{18}F]MNI1126 prepared from **9** using Cu(4-PhPy)$_4$(ClO$_4$)$_2$ as mediator in DMI at 110 °C for 10 min under argon and the corresponding non-radioactive reference compound. Blue trace: UV, λ = 254 nm; green trace: MNI1126; red trace: radioactivity. Abbreviation: p.-c.i. – post-column injection.

3.12 Preparation of 3-[^{18}F]FPhes and (*S*)-αMe-3-[^{18}F]FPhe – General Procedure (GP8)

[^{18}F]F^{-} was eluted from the QMA cartridge with a solution of Et_4NOTf (1 mg, 3.6 µmol) in anhydrous *n*BuOH (400 µL) into a solution of $B(OH)_2$ or Bpin substituted [Ni(II)-BPB] or [Ni(II)-BPA] complex (10 µmol of each) in the corresponding anhydrous solvent (800 µL). The reaction mixture was heated at 110 °C for 10 min under air. After cooling to ambient temperature, the reaction mixture was diluted with 5 ml H_2O, loaded onto a ChromaFix C18 ec cartridge (preconditioned with 1 mL EtOH and 5 mL H_2O), and washed with H_2O (5 mL). The labeled intermediate was eluted with EtOH (1 mL) and the resulting solution was concentrated under reduced pressure at 80 °C for 5 min in a stream of argon. Thereafter, 2 M HCl (500 µl) was added and the resulting mixture was stirred for another 10 min at 110 °C. After cooling to ambient temperature, the mixture was purified by preparative HPLC. The fraction containing the radiolabeled amino acid was collected and neutralized with $NaHCO_3$ to afford the desired PET tracer in a ready-to-use form.

Radiosynthesis of 3-[^{18}F]FPhes and (*S*)-αMe-3-[^{18}F]FPhe under optimized conditions

Radiosynthesis of 3-[^{18}F]FPhes was performed according to GP8 in *n*BuOH/DMI using Cu(3,4-$Me_2Py)_4(OTf)_2$ as radiolabeling mediator (n=3).

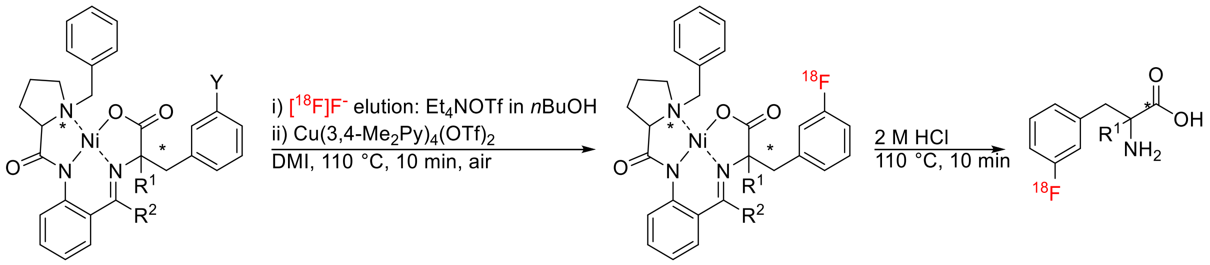

R^1 = H, R^2 = Ph, Y = $B(OH)_2$ [(*S*,*S*)-**10**]
R^1 = H, R^2 = Ph, Y = Bpin [(*S*,*S*)-**11**] — (*S*)-3-[^{18}F]FPhe

R^1 = H, R^2 = Ph, Y = $B(OH)_2$ [(*R*,*R*)-**10**]
R^1 = H, R^2 = Ph, Y = Bpin [(*R*,*R*)-**11**] — (*R*)-3-[^{18}F]FPhe

R^1 = Me, R^2 = H, Y = $B(OH)_2$ [(*S*,*S*)-**12**]
R^1 = Me, R^2 = H, Y = Bpin [(*R*,*R*)-**13**] — (*S*)-αMe-3-[^{18}F]FPhe

Table S46: Radiosynthesis of [^{18}F]FPhes and (*S*)-αMe-3-[^{18}F]FPhe under optimized conditions.

Entry	PET tracer	Y	RCC [%]	AY [%]
1	(*S*)-3-[^{18}F]FPhes	$B(OH)_2$	91 ± 4	41 ± 2
2		Bpin	74 ± 1	
3	(*R*)-3-[^{18}F]FPhes	$B(OH)_2$	78 ± 3	33 ± 0.5
4		Bpin	70 ± 7	
5	(*S*)-αMe-3-[^{18}F]FPhe	$B(OH)_2$	69 ± 11	
6		Bpin	76 ± 2	

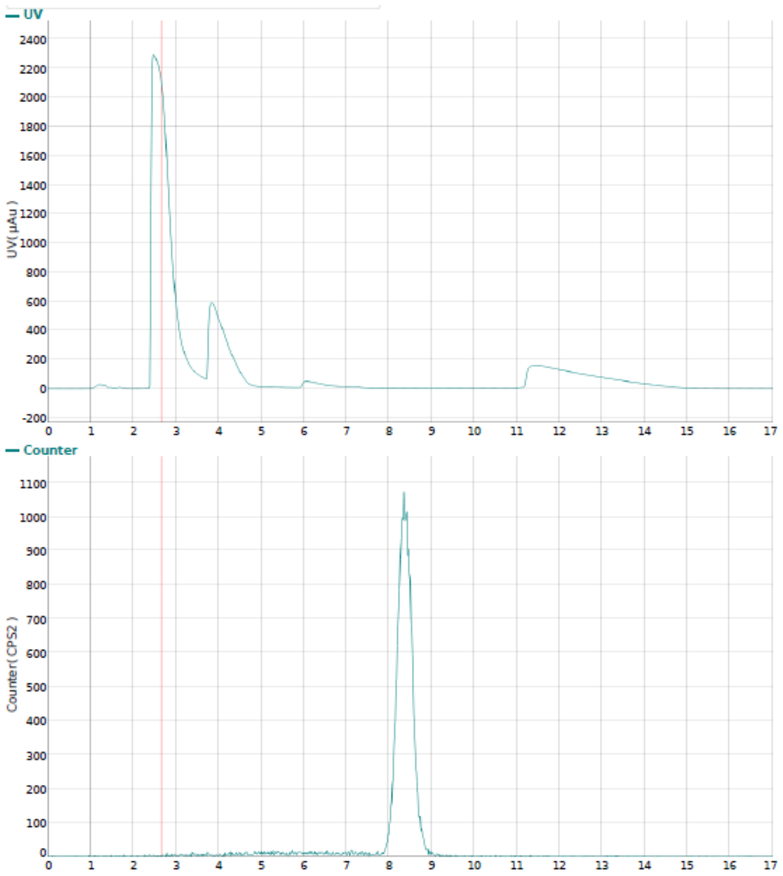

Figure S53: Purification of (*S*)-3-[^{18}F]FPhe by preparative HPLC (top: UV chromatogram, λ=254 nm, bottom: radio-chromatogram).

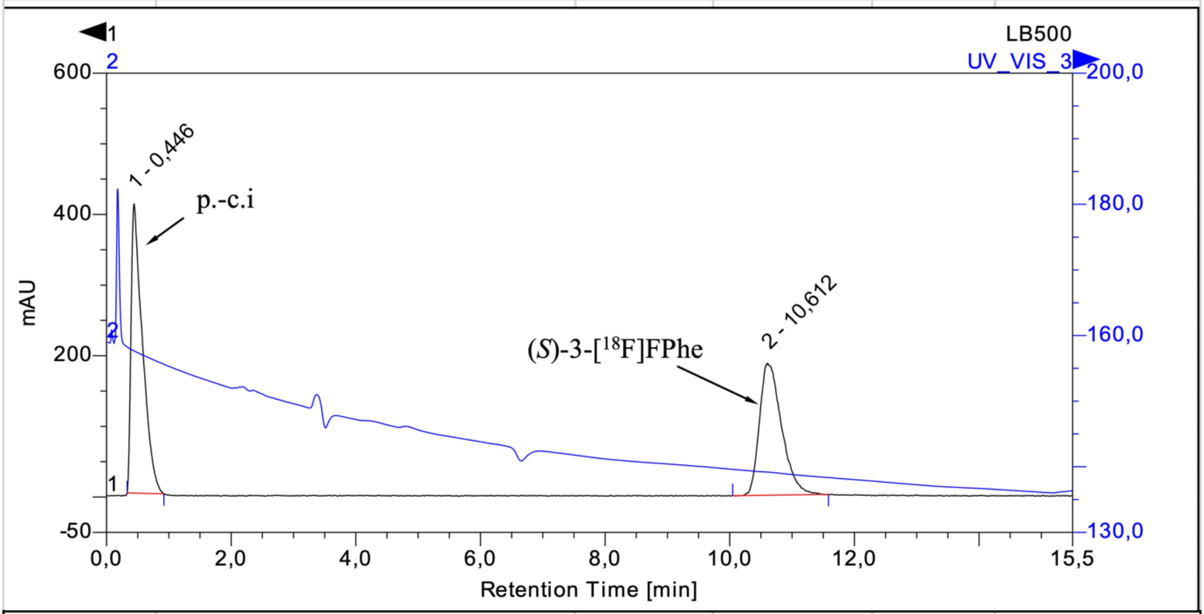

Nr.	R(t)	Intensity	Plates(EP)	Area	Resolution(EP)
	min	mAU		mV*min	
1	0,45	409,415	29	86,525	21,06
2	10,61	186,633	4456	78,819	n.a.

Figure S54: HPLC chromatogram of purified (*S*)-3-[^{18}F]FPhe for quality control. Blue trace: UV channel, λ=254 nm; red trace: radioactivity channel.

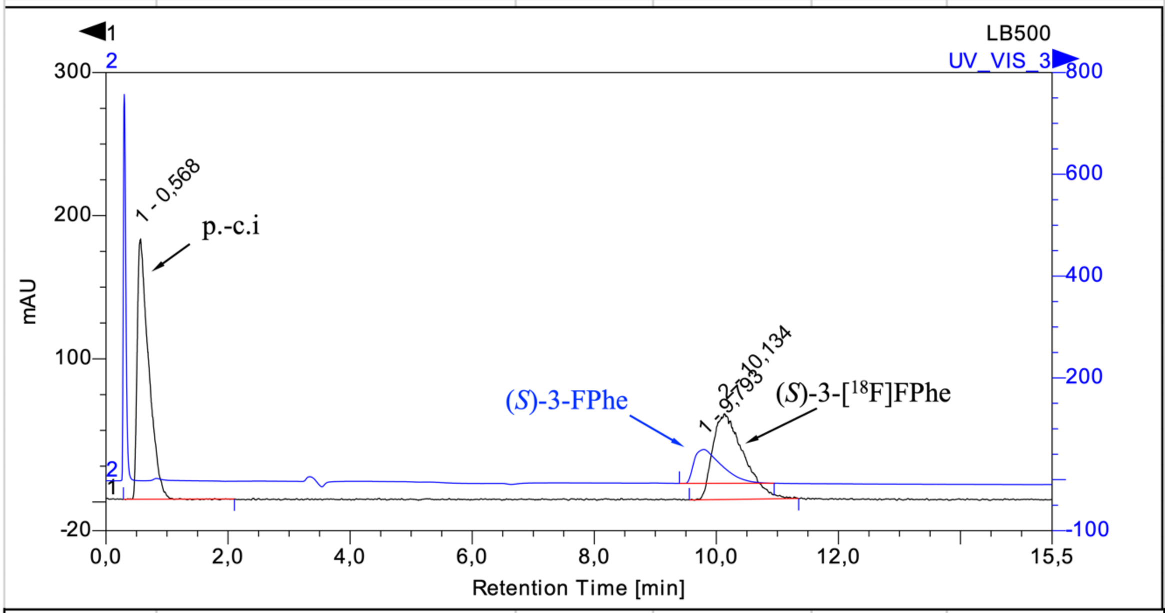

Nr.	R(t)	Intensity	Plates(EP)	Area	Resolution(EP)
	min	mAU		mV*min	
1	0,57	181,798	43	39,482	14,89
2	10,13	59,951	1846	34,966	n.a.

Figure S55: Co-injection of (*S*)-3-[^{18}F]FPhe with the non-radioactive reference compound. Blue trace: UV channel, λ=254 nm; black trace: radioactivity channel.

Determination of carrier amount and molar activity for (*S*)-3-[^{18}F]FPhe

An aliquot of the tracer solution (20 µL) was analyzed by analytical HPLC as described above. The carrier amount was determined from the peak area and the molar activity was calculated according to a calibration curve (Figure **S56**), which was obtained using different concentrations of 3-FPhe (Table **S47**).

$$A_M = \frac{A\left[\frac{\text{GBq}}{\text{mL}}\right]}{c\left[\frac{\mu\text{mol}}{\text{mL}}\right]}$$

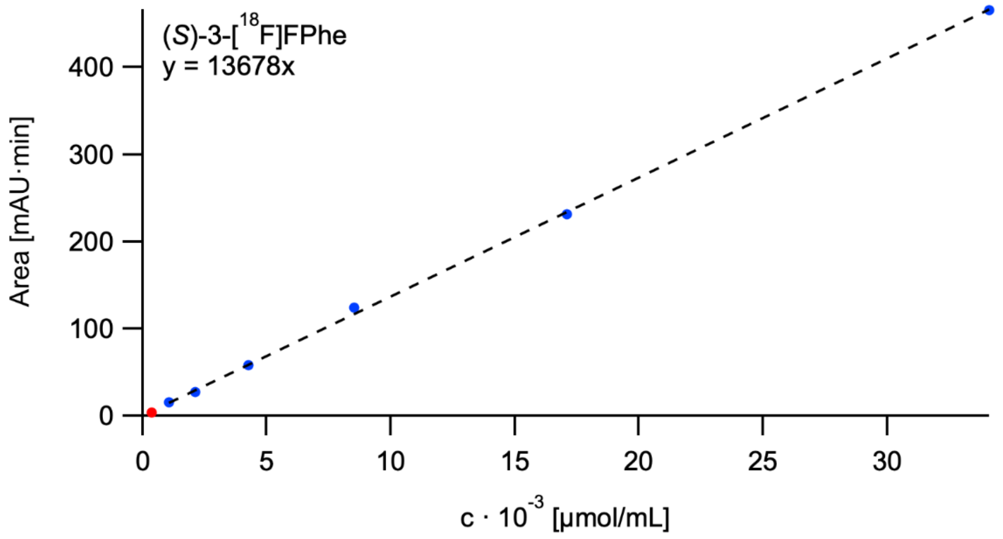

Figure S56: Calibration curve of 3-fluorophenylalanine for calculation of the molar activity of (*S*)-3-[^{18}F]FPhe. Raw data are shown in Table **S47**.

Table S47: Calibration data (measured at 254 nm) for determination of molar activity of (*S*)-3-[^{18}F]FPhe.

**Concentration [µmol/mL]**	**Amount [µg]**	**Peak area [mAU·min]**
0.0683	12.5	1178.8
0.0341	6.25	685.3
0.0171	3.13	347.9
0.00853	1.56	192.2
0.00427	0.781	130.1
0.00213	0.390	58.9
Measured sample:		
0.000249	0.046	3.4

Volume [mL]	13.4
Activity [GBq]	1.8
Carrier amount (µg)	0.62
Carrier concentration [µmol/mL]	$2.49 \cdot 10^{-4}$
Molar activity [GBq/µmol]	538

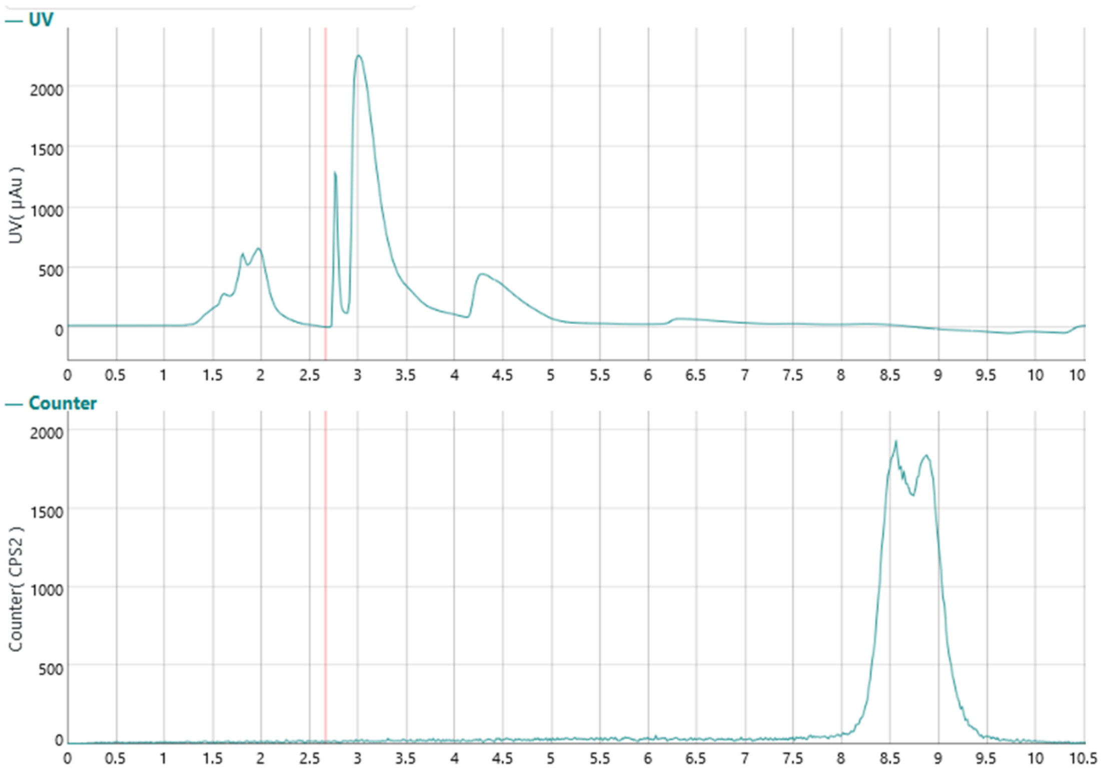

Figure S57: Purification of (*R*)-3-[^{18}F]FPhe by preparative HPLC (top: UV trace, λ=254 nm; bottom: radioactivity trace).

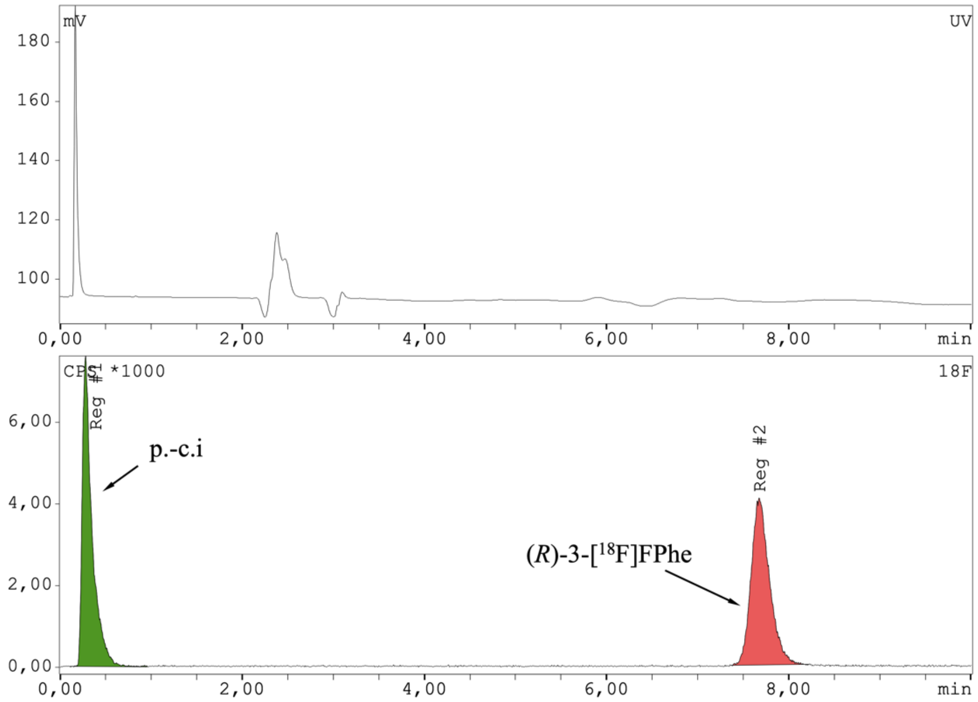

Name	Channel	Start, min	End, min	Area
Reference Injection	^{18}F	0.1	1.0	57223,19
3-(*R*)-[^{18}F]FPhe	^{18}F	7.4	8.2	57433,21

Figure S58: HPLC chromatogram of purified (*R*)-3-[^{18}F]FPhe for quality control. Top: UV trace, λ=254 nm; bottom: radioactivity trace.

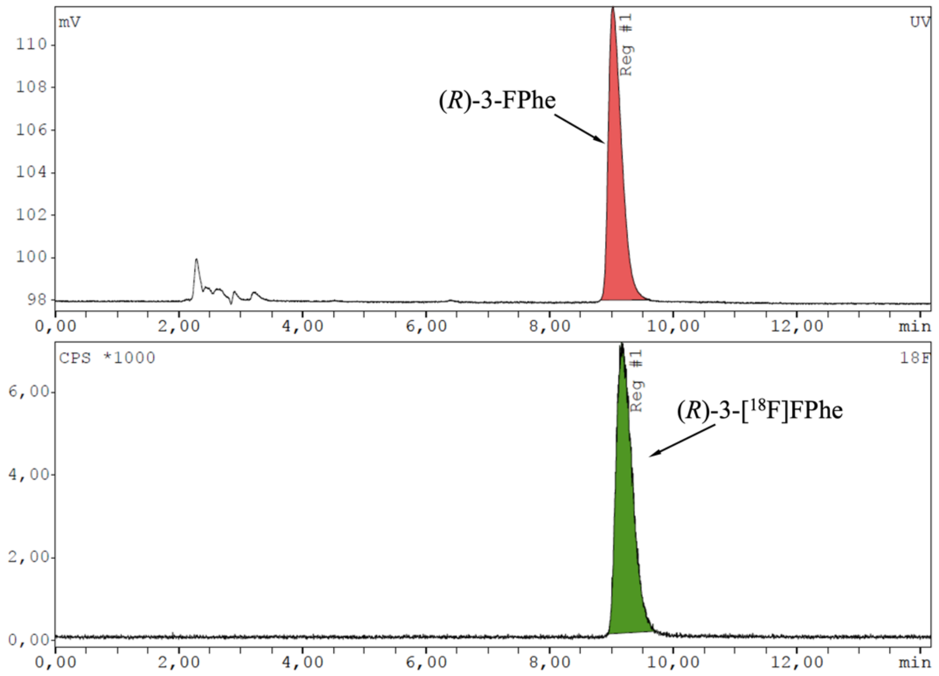

Name	Channel	t_R, min	Area
Non-reference compound	UV	0.1	203.1 mV·min
3-(*R*)-[^{18}F]FPhe	^{18}F	9.2	125296.0 counts·min

Figure S59: Co-injection of (*R*)-3-[^{18}F]FPhe with the non-radioactive reference compound. Top trace: UV channel, λ=254 nm; bottom trace: radioactivity channel.

Radiosynthesis of 3-[^{18}F]FPhes and (*S*)-αMe-3-[^{18}F]FPhe under standard conditions

Radiosynthesis of 3-[^{18}F]FPhes and (*S*)-αMe-3-[^{18}F]FPhe was performed according to GP5 in *n*BuOH/DMA using $Cu(Py)_4(OTf)_2$ as mediator (n=3).

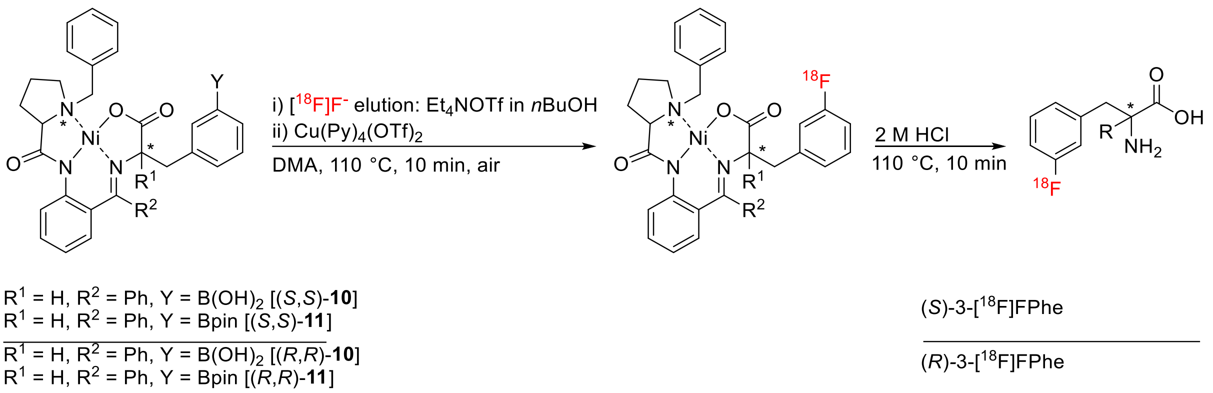

Table S48: Radiosynthesis of [^{18}F]FPhes under standard conditions.

Entry	PET tracer	Y	RCC [%]	AY [%]
1	(*S*)-3-[^{18}F]FPhe	$B(OH)_2$	9 ± 4	17 ± 6
2		Bpin	8 ± 1	
3	(*R*)-3-[^{18}F]FPhe	$B(OH)_2$	21 ± 2	14 ± 4
4		Bpin	11 ± 2	
5	(*S*)-αMe-3-[^{18}F]FPhe	$B(OH)_2$	14 ± 3	
6		Bpin	9 ± 2	

3.13 Automated radiosynthesis of 3-(*S*)-[^{18}F]FPhe in an AllInOne synthesis module (Trasis)

Cassette layout is depicted in Figure **S60**. Aqueous [^{18}F]F^{-} was loaded (from the male side) onto an anion-exchange resin (QMA-cartridge), washed with MeCN (1 mL, Vial 2) and subsequently eluted (from the female side) into reactor 1 using Bu_4NOH solution (25 mg $Bu_4NOH \cdot 30\ H_2O$ in 0.5 mL MeCN, Vial 1). All volatiles were removed under reduced pressure in a stream of N_2 at 95 °C for 5 min. That followed, a solution of the precursor and catalyst (10 µmol precursor and 15.8 mg catalyst in 0.25 mL *n*BuOH and 0.5 mL DMI, Vial 3) was added and the reaction mixture was heated at 110 °C for 15 min. The reaction mixture was diluted with H_2O (5 mL) and loaded onto a Chromafix C_{18} SPE cartridge which was subsequently washed with H_2O (5 mL) and dried in a stream of N_2 for 1 min. The radiolabeled intermediate was eluted with EtOH (1 mL) into reactor 2 and all volatiles were removed under reduced pressure in a stream of N_2 at 100 °C for 10 min. Afterwards, 1 M HCl (0.5 mL, Vial 4) was added and deprotection was carried out at 110 °C for 10 min. The resulting mixture was diluted with H_2O (5 mL) and 3-(*S*)-[^{18}F]FPhe was isolated by HPLC [column: Hydro RP, 250×10 mm, 5 µm/100 Å, Phenomenex; eluent: 10% EtOH (0.1% H_3PO_4), flow rate: 7.4 mL/min]. The product was collected through a sterile filter directly into the product vial.

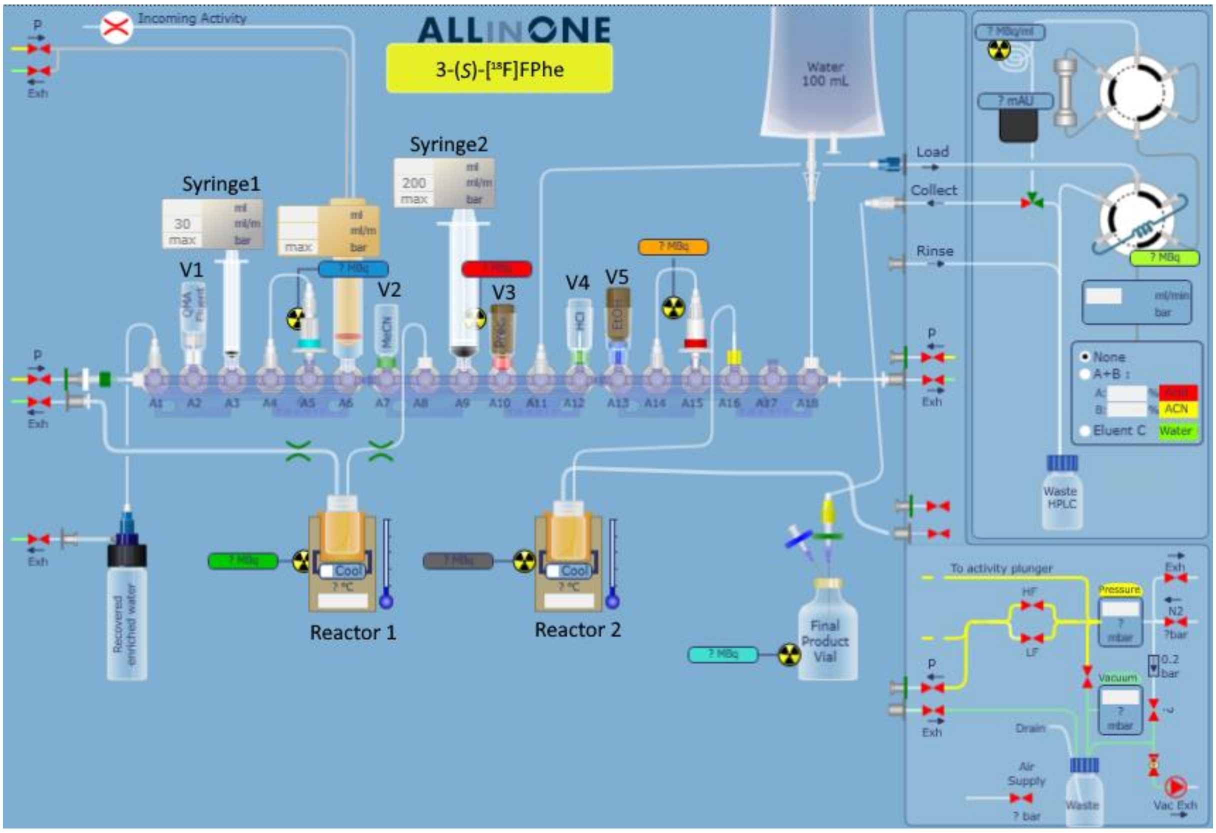

Figure S60: Schematic diagram of the Trasis AIO module with the custom made cassette for preparation of 3-(S)-$[^{18}F]$FPhe. A2: V1 QMA eluent (25 mg $Bu_4NOH{\cdot}30\ H_2O$ in 0.5 mL MeCN) (4 mL Vial); A5: Sep-Pak Acell Plus QMA Carbonate Plus Light cartridge (130 mg); A7: V2 0.5 mL MeCN (4 mL Vial); A10: V3 Precursor solution: 10 µmol precursor and 15.8 mg $Cu(3,4\text{-}Me_2Py)_4(OTf)_2$ in 0.25 mL nBuOH and 0.5 mL DMI (4 mL Vial); A12: V4 0.5 mL 0.5 M HCl (4 mL Vial); A13: V5 1 mL EtOH (4 ml Vial); A15: Macherey-Nagel Chromafix C18 ec (s) cartridge (270 mg).

Table S49: Process sequence for the automated synthesis of 3-(S)-[^{18}F]FPhe using the Trasis AIO module.

Entry	Process	Activated Path/Function
1	Loading of [^{18}F]fluoride onto the QMA cartridge	A6-A5-QMA-A4-A1
2	Washing of QMA with MeCN (1 mL)	V2-A7-A5-QMA-A4-A3-syringe 1
3	Elution of [^{18}F]fluoride from the cartridge into reactor 1	A2-A3-syringe 1-A4-QMA-A5-A8-reactor 1
4	Drying, reactor 1, 95 °C, 5 min, N_2 flow, vacuum	P-A8-R1-Exh
5	Addition of precursor and $Cu(3,4\text{-}Me_2Py)_4(OTf)_2$	V3-A10-A8-reactor1
6	Radiofluorination, 110 °C, 15 min	-
7	Dilution with H_2O (5 mL)	A18-A9-syringe 2, then R1-A8-A9-syringe 2
8	Loading onto SPE cartridge	Syringe 2-A9-A14-cartridge-A15-exh
9	Washing of SPE cartridge with H_2O (5 mL)	A18-A9-syringe 2, then syringe 2-A9- A14-cartridge-A15-exh
10	Drying of SPE cartridge with N_2 (1 min)	P- A14-cartridge-A15-exh
11	Elution of intermediate compound from SPE cartridge into reactor 2	V5-A13-A9-syringe 2, then syringe 2-A9-A14-SPE-A15-A16-reactor 2
12	Drying, reactor 2, 100 °C, 10 min, N_2 flow, vacuum	P-A16-exh
13	Addition of 0.5 M HCl (0.5 mL)	V4-A12-A16-reactor 2
14	Deprotection, 110 °C, 10 min	-
15	Dilution with H_2O (5 mL)	A18-A9-syringe 2, then reactor2-A16-A9-syringe 2
16	Transfer to HPLC system	Syringe-A9-A11-Load
17	Collection of 3-(*S*)-[^{18}F]FPhe into the product vial	Collect-sterile filter-final product vial

3.14 Radiolabeling at 2.5 µmol precursor loading. Initial experiments

The initial experiments were performed according to GP5 in *n*BuOH/DMI using **1** (2.5 µmol) as model substrate with four different radiolabeling mediators. RCCs were determined by radio-HPLC as described above. Representative HPLC chromatograms of the corresponding reaction mixtures are shown in Figure **S61** and Figure **S62**.

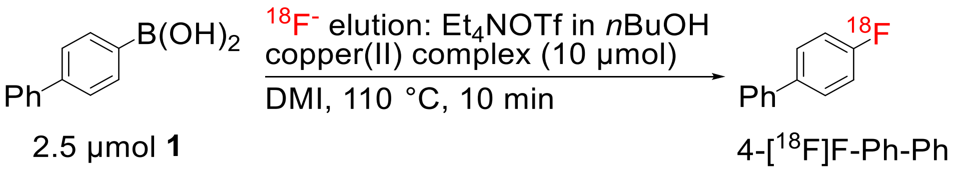

Table S50: Dependency of RCCs for ^{18}F-fluorination of **1** (2.5 µmol) on the applied copper complex.

Entry	Cu(II) complex	RCC [%]
1	$Cu(Py)_4(OTf)_2$	37 ± 2
2	$Cu(4\text{-}PhPy)_4(ClO_4)_2$	56 ± 2
3	$Cu(3,4\text{-}Me_2Py)_4(OTf)_2$	40 ± 2
4	$Cu(3,4\text{-} Me_2Py)_4(ClO_4)_2$	33 ± 2

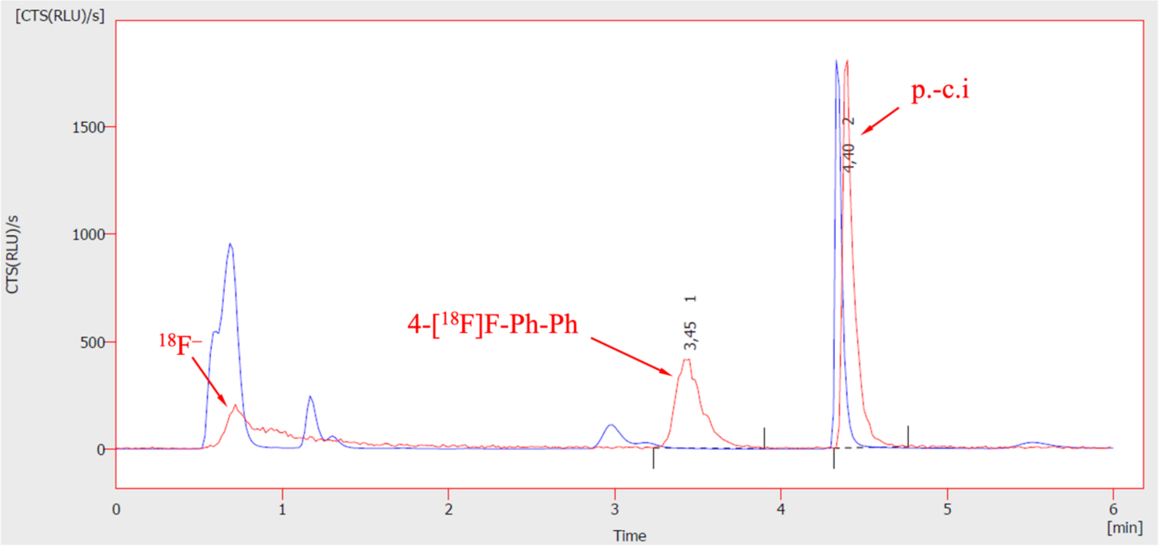

Result Table (Uncal - Data|alc-enhanced_667_07_04_2022_[1 DMI]biphenyl-B(OH)2_2,5umol - HERM)

	Reten. Time [min]	Area [CTS(RLU)/s.s]	Height [CTS(RLU)/s]	Area [%]
1	3,450	4621,000	413,300	35,1
2	4,400	8550,500	1803,074	64,9
	Total	13171,500	2216,374	100,0

Figure S61: HPLC traces of crude 4-[^{18}F]F-Ph-Ph prepared from **1** (2.5 µmol) using $Cu(Py)_4(OTf)_2$ as mediator. Blue trace: UV, λ = 254 nm; red trace: radioactivity. Abbreviation: p.-c.i – post-column injection.

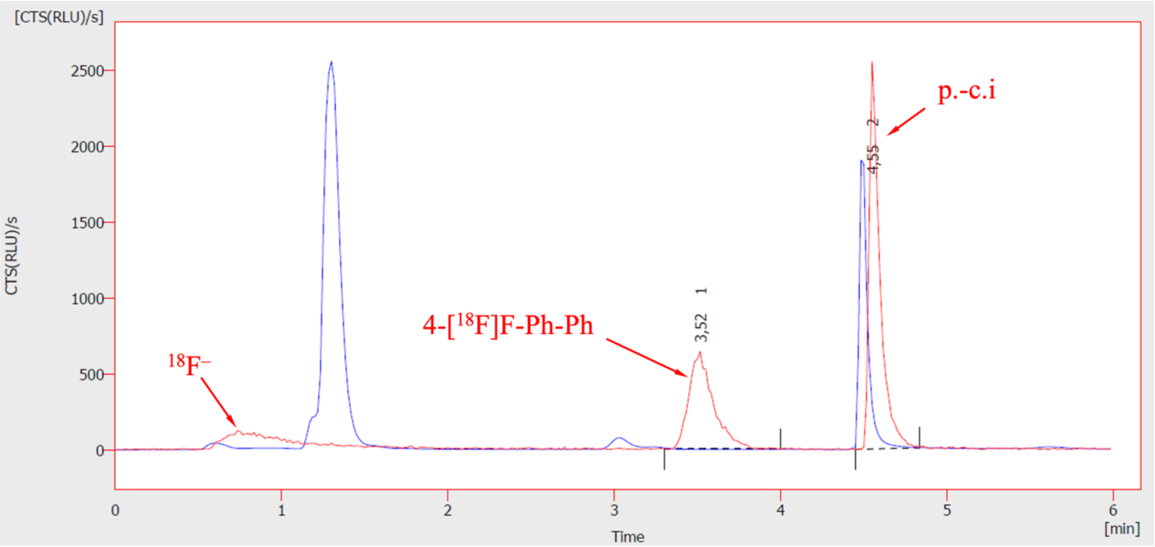

Result Table (Uncal - Data\alc-enhanced_527_18_02_2022_[4 DMI]_biphenyl-B(OH)2_10umol-2.5umol - HERM)

	Reten. Time [min]	Area [CTS(RLU)/s.s]	Height [CTS(RLU)/s]	Area [%]
1	3,517	6550,000	644,000	36,2
2	4,550	11555,000	2554,391	63,8
	Total	18105,000	3198,391	100,0

Figure S62: HPLC traces of crude 4-[^{18}F]F-Ph-Ph prepared from **1** (2.5 µmol) using Cu(4-PhPy)$_4$(ClO$_4$)$_2$ as mediator. Blue trace: UV, λ = 254 nm; red trace: radioactivity. Abbreviation: p.-c.i – post-column injection.

Statistical evaluation of the dependency of RCCs for radiolabeling of 1 (2.5 µmol) on the applied Cu-complex

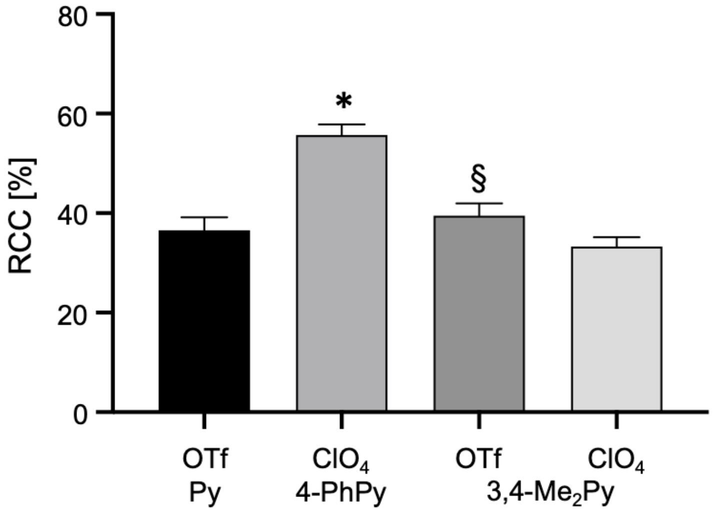

Figure S63: Dependency of RCCs for radiolabeling of **1** (2.5 µmol) on the applied Cu-complex (10 µmol). Solvent: *n*BuOH/DMI; conditions: 110 °C for 10 min. Statistics: 1-way ANOVA followed by Tukey's multiple comparison test. * $p<0.05$ for comparison of Cu(4-PhPy)$_4$(ClO$_4$)$_2$ with all other Cu-complexes. § $p<0.05$ for Cu(3,4-Me$_2$Py)$_4$(OTf)$_2$ *vs.* Cu(3,4-Me$_2$Py)$_4$(ClO$_4$)$_2$.

Table S51: Screening of Cu mediators for radiolabeling of **1** (2.5 µmol); n=3 per group. 1-way ANOVA followed by Tukey's multiple comparison test. Main effect: $F(3,8)=56.99$, $p<0.0001$.

	RCC (%)	comparison	
Cu(Py)$_4$(OTf)$_2$	36.55 ± 2.61	vs. Cu(4-PhPy)$_4$(ClO$_4$)$_2$	p<0.0001*
		vs. Cu(3,4-Me$_2$Py)$_4$(OTf)$_2$	p=0.4367
		vs. Cu(3,4-Me$_2$Py)$_4$(ClO$_4$)$_2$	p=0.3661
Cu(4-PhPy)$_4$(ClO$_4$)$_2$	55.73 ± 2.13	vs. Cu(3,4-Me$_2$Py)$_4$(OTf)$_2$	p=0.0001*
		vs. Cu(3,4-Me$_2$Py)$_4$(ClO$_4$)$_2$	p<0.0001*
Cu(3,4-Me$_2$Py)$_4$(OTf)$_2$	39.51 ± 2.46	vs. Cu(3,4-Me$_2$Py)$_4$(ClO$_4$)$_2$	p=0.0421§
Cu(3,4-Me$_2$Py)$_4$(ClO$_4$)$_2$	33.31 ± 1.88		

* p<0.05

Scope of the novel radiolabeling protocol at ≤2.5 µmol precursor loading (Tables S52, S53)

Radiolabeling experiments were performed according to GP5 (boronate precursors) in *n*BuOH/DMI or GP7-B at 110 ºC (stannane precursors) using Cu(4-PhPy)$_4$(ClO$_4$)$_2$ (10 µmol) as radiolabeling mediator (n=3). RCCs were determined by radio-HPLC as described above. Representative HPLC chromatograms of radiolabeled products are shown in Figure **S64–S87**.

Table S52: Radiolabeled compounds prepared using the novel ^{18}F-fluorination protocol at ≤2.5 µmol precursor loading.

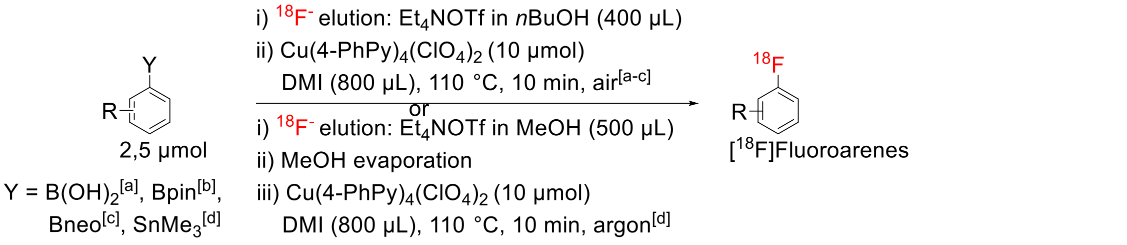

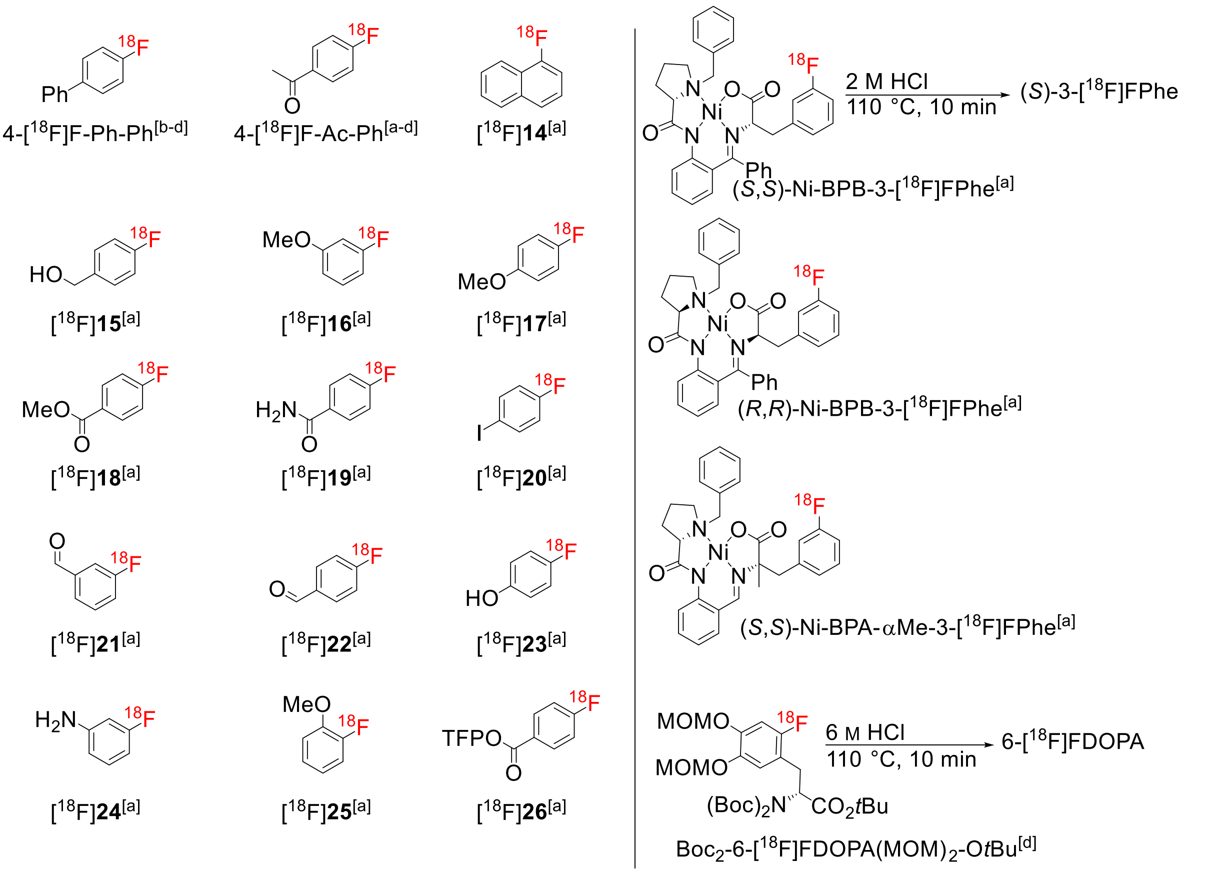

Table S53: Scope of the novel radiolabeling protocol at ≤2.5 µmol precursor loading.

Entry	Radiolabeled product	RCC [%]
1	[^{18}F]F-Ph-Ph	56 ± 2[a]
2		48 ± 6[b]
3		63 ± 12[c]
4		30 ± 0.2[d]
5	[^{18}F]F-Ac-Ph	53 ± 3[a]
6		25 ± 2[b]
7		28 ± 4[c]
8		37 ± 3[d]
9	[^{18}F]**14**	76 ±5[a]
10		62 ± 17[e]
11		29 ± 8[f]
12	[^{18}F]**15**	76 ± 8[a]
13	[^{18}F]**16**	44 ± 2[a]
14	[^{18}F]**17**	28 ± 3[a]
15	[^{18}F]**18**	51 ± 5[a]
16	[^{18}F]**19**	54 ± 14[a]
17	[^{18}F]**20**	29 ± 5[a]
18	[^{18}F]**21**	30 ± 6[a]
19	[^{18}F]**22**	34 ± 6[a]
20	[^{18}F]**23**	39 ± 2[a]
21	[^{18}F]**24**	19 ± 2[a]
22	[^{18}F]**25**	18 ± 3[a]
23	[^{18}F]**26**	6 ± 1[a]
24		28 ± 3[g] (n=2)
25		63 ± 2[g, h]
26	(*S*,*S*)-Ni-BPB-3-[^{18}F]FPhe	62 ± 5[a] (AY = 23 ± 1)
27	(*R*,*R*)-Ni-BPB-3-[^{18}F]FPhe	46 ± 9[a]
28	(*S*,*S*)-Ni-BPB-αMe-3-[^{18}F]FPhe	53 ± 2[a]
29	Boc_2-6-[^{18}F]FDOPA$(MOM)_2$-O*t*Bu	60 ± 5[d]
30		10 ± 3[d,e]

Radiolabeled products were prepared from the respective [a]aryl boronic acid, [b]pinacol boronate, [c]neopentyl glycol boronate or [d]trimethyl stannyl precursor. [e]Prepared from 1 µmol precursor. [f]Prepared from 0.5 µmol precursor. [g]Radiosyntheses were performed according to GP7A. [h]Prepared from 10 µmol precursor.

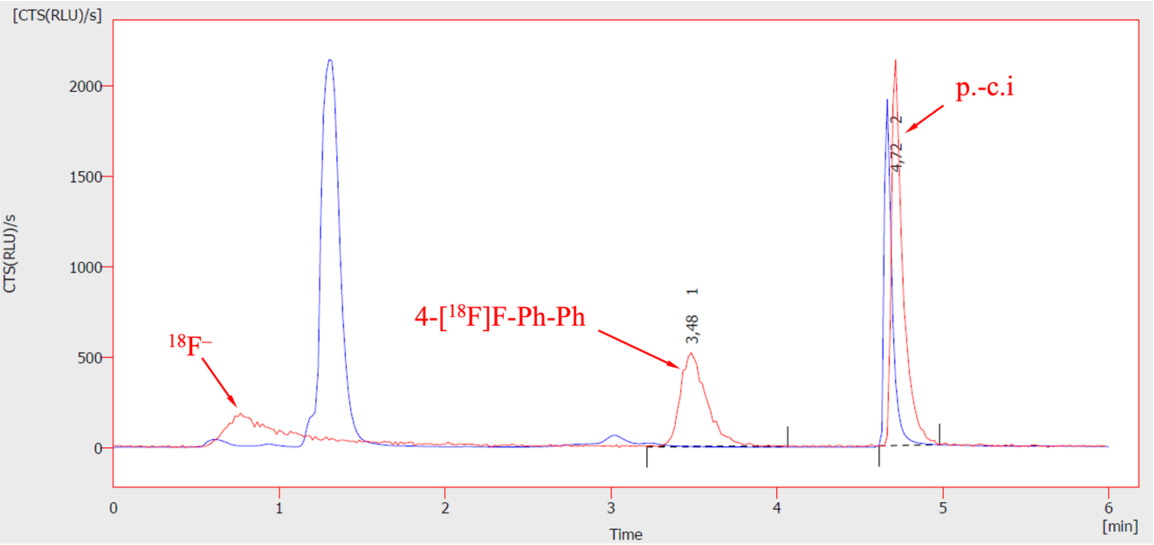

Result Table (Uncal - Data|alc-enhanced_529_18_02_2022_[4 DMI]_biphenyl-Bpin_10umol-2.5umol - HERM)

	Reten. Time [min]	Area [CTS(RLU)/s.s]	Height [CTS(RLU)/s]	Area [%]
1	3,483	5801,000	520,000	36,3
2	4,717	10177,000	2133,091	63,7
	Total	15978,000	2653,091	100,0

Figure S64: HPLC traces of crude 4-[^{18}F]F-Ph-Ph prepared from **2** (2.5 µmol). Blue trace: UV, λ = 254 nm; red trace: radioactivity. Abbreviation: p.-c.i – post-column injection.

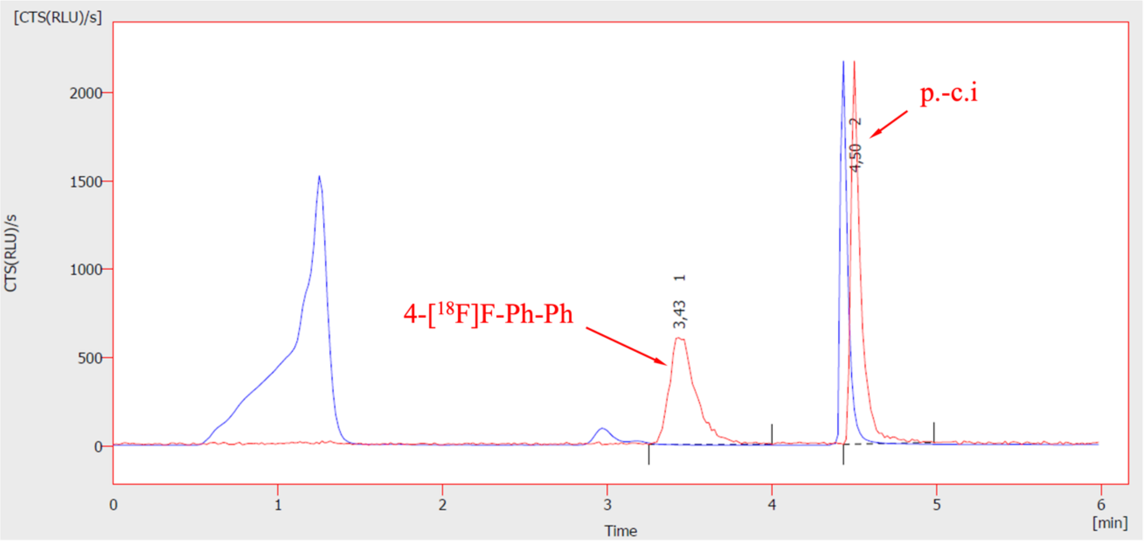

Result Table (Uncal - Data|alc-enhanced_561_01_03_2022_[4 DMI]biphenyl-Bneo_2.5umol - HERM)

	Reten. Time [min]	Area [CTS(RLU)/s.s]	Height [CTS(RLU)/s]	Area [%]
1	3,433	6861,500	603,756	42,9
2	4,500	9120,500	2167,909	57,1
	Total	15982,000	2771,665	100,0

Figure S65: HPLC traces of crude 4-[^{18}F]F-Ph-Ph prepared from 4-Ph-Ph-Bneo (2.5 µmol). Blue trace: UV, λ = 254 nm; red trace: radioactivity. Abbreviation: p.-c.i – post-column injection.

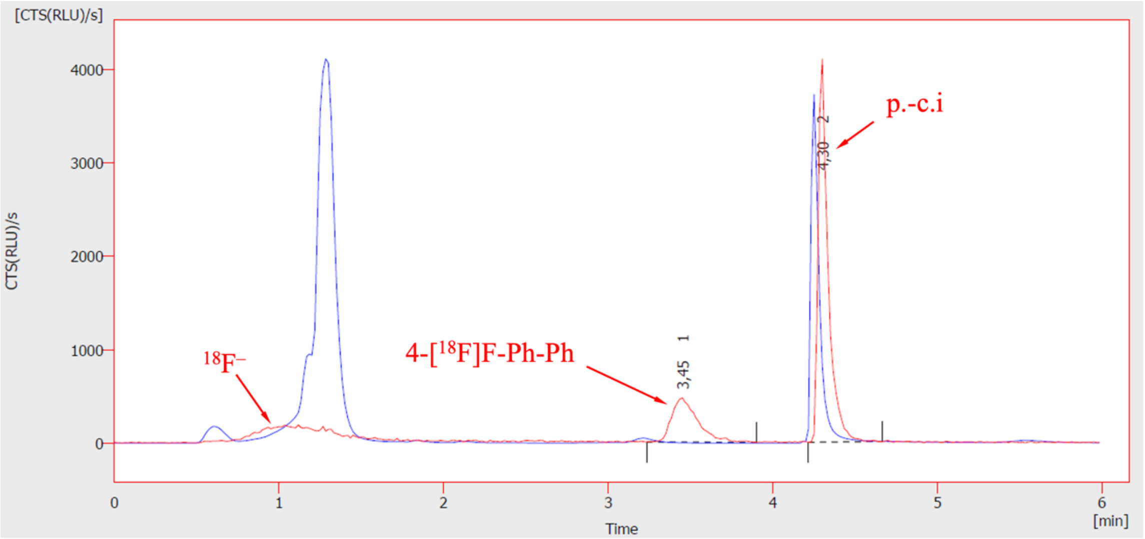

Result Table (Uncal - Data|alc-enhanced_661_04_04_2022_[4 DMI]biphenyl-SnMe3_2,5umol - HERM)

	Reten. Time [min]	Area [CTS(RLU)/s.s]	Height [CTS(RLU)/s]	Area [%]
1	3,450	5255,000	474,650	23,2
2	4,300	17404,000	4102,519	76,8
	Total	22659,000	4577,169	100,0

Figure S66: HPLC traces of crude 4-[^{18}F]F-Ph-Ph prepared from **5** (2.5 µmol). Blue trace: UV, λ = 254 nm; red trace: radioactivity. Abbreviation: p.-c.i – post-column injection.

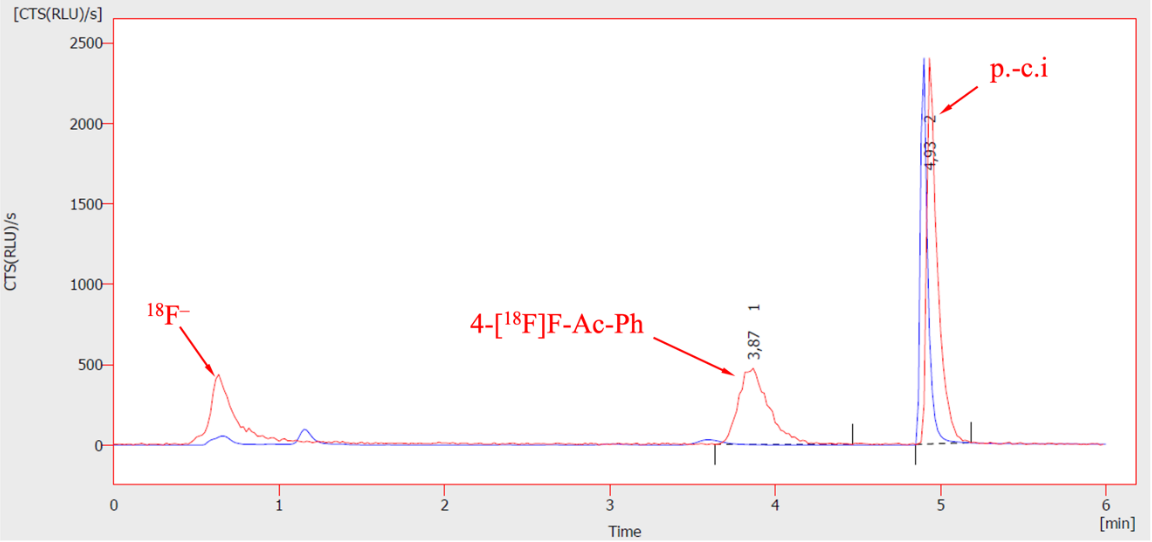

Result Table (Uncal - Data|alc-enhanced_530_18_02_2022_[4 DMI]_4-acetylphenyl-B(OH)2_10umol-2.5umol - HERM)

	Reten. Time [min]	Area [CTS(RLU)/s.s]	Height [CTS(RLU)/s]	Area [%]
1	3,867	6089,000	474,720	36,3
2	4,933	10699,000	2400,000	63,7
	Total	16788,000	2874,720	100,0

Figure S67: HPLC traces of crude 4-[^{18}F]F-Ac-Ph prepared from **3** (2.5 µmol). Blue trace: UV, λ = 254 nm; red trace: radioactivity. Abbreviation: p.-c.i – post-column injection.

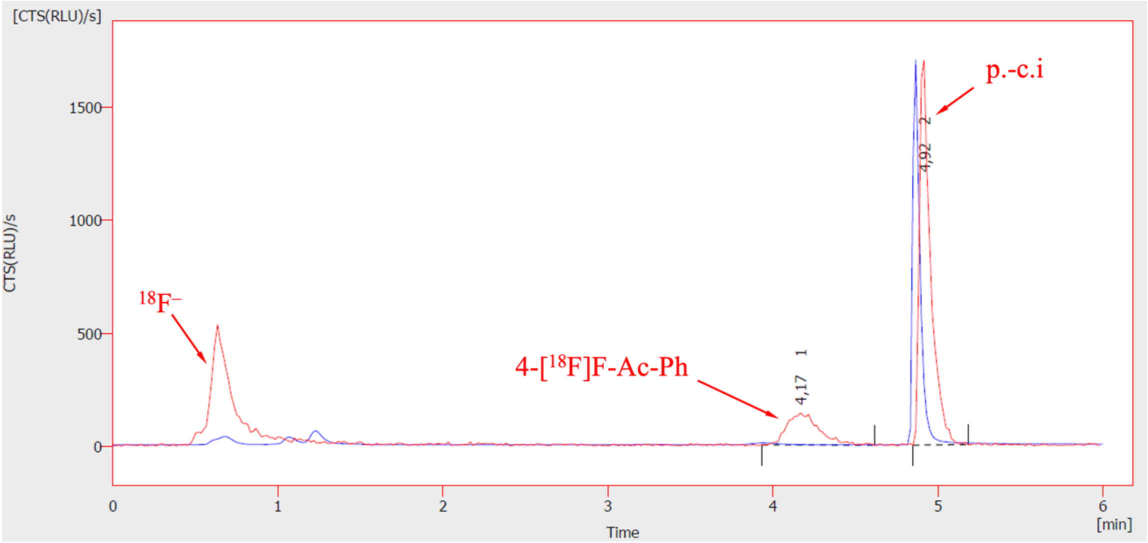

Result Table (Uncal - Data|alc-enhanced_533_18_02_2022_[4 DMI]_4-acetylphenyl-Bpin_10umol-2.5umol - HERM)

	Reten. Time [min]	Area [CTS(RLU)/s.s]	Height [CTS(RLU)/s]	Area [%]
1	4,167	1944,000	141,683	19,4
2	4,917	8087,000	1701,600	80,6
	Total	10031,000	1843,283	100,0

Figure S68: HPLC traces of crude 4-[^{18}F]F-Ac-Ph prepared from **4** (2.5 µmol). Blue trace: UV, λ = 254 nm; red trace: radioactivity. Abbreviation: p.-c.i – post-column injection.

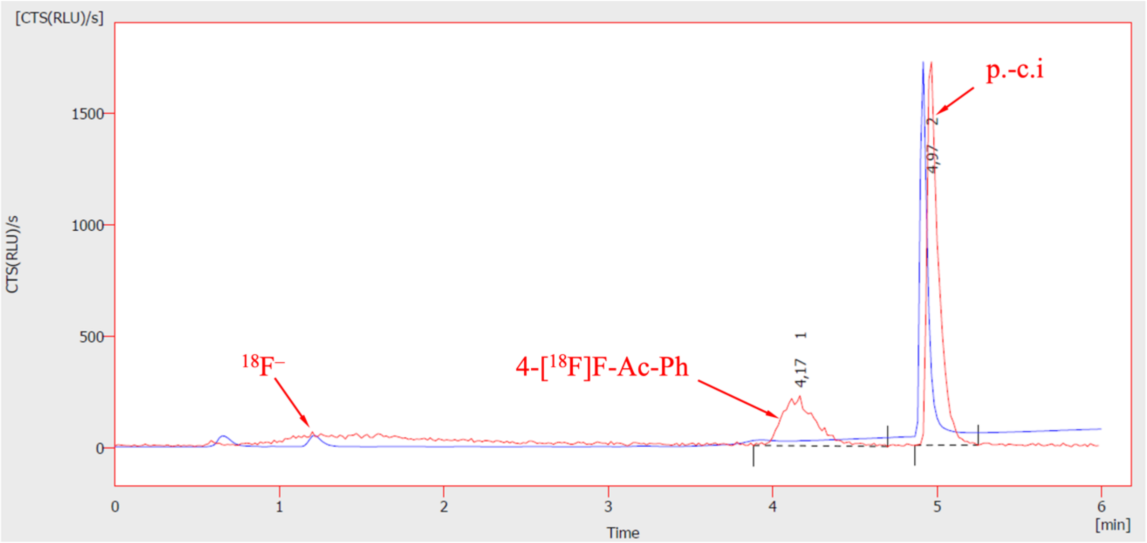

Result Table (Uncal - Data|alc-enhanced_562_01_03_2022_[4 DMI]4-acetylphenyl-Bneo_2.5umol - HERM)

	Reten. Time [min]	Area [CTS(RLU)/s.s]	Height [CTS(RLU)/s]	Area [%]
1	4,167	3191,500	225,735	27,5
2	4,967	8423,500	1720,739	72,5
	Total	11615,000	1946,474	100,0

Figure S69: HPLC traces of crude 4-[^{18}F]F-Ac-Ph prepared from 4-Ac-Ph-Bneo (2.5 µmol). Blue trace: UV, λ = 254 nm; red trace: radioactivity. Abbreviation: p.-c.i – post-column injection.

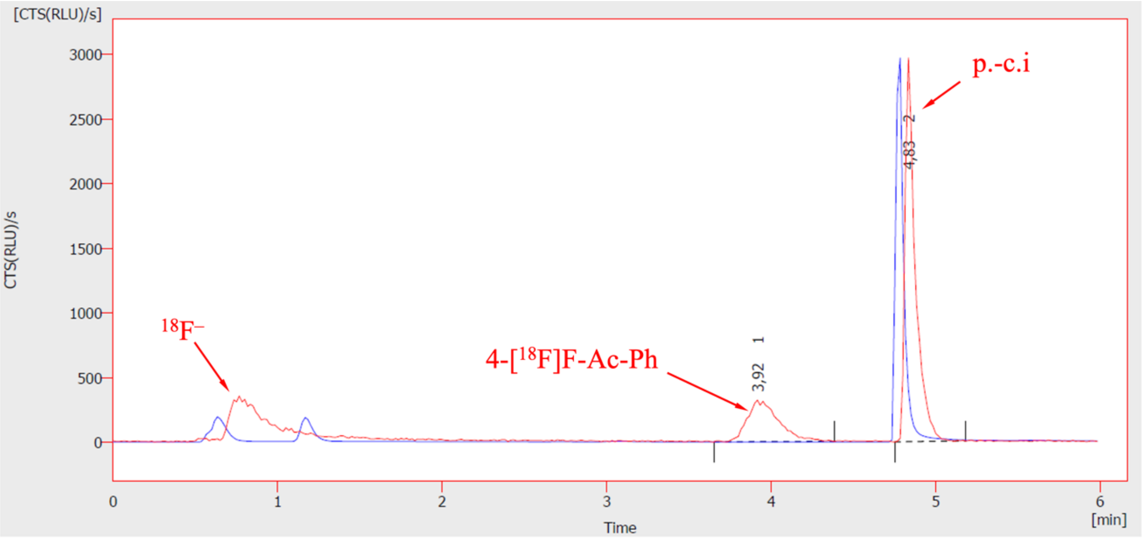

Result Table (Uncal - Data|alc-enhanced_662_04_04_2022_[4 DMI]4-acetylphenyl-SnMe3_2,5umol - HERM)

	Reten. Time [min]	Area [CTS(RLU)/s.s]	Height [CTS(RLU)/s]	Area [%]
1	3,917	4278,000	322,909	24,6
2	4,833	13090,000	2971,846	75,4
	Total	17368,000	3294,755	100,0

Figure S70: HPLC traces of crude 4-[^{18}F]F-Ac-Ph prepared from **6** (2.5 µmol). Blue trace: UV, λ = 254 nm; red trace: radioactivity. Abbreviation: p.-c.i – post-column injection.

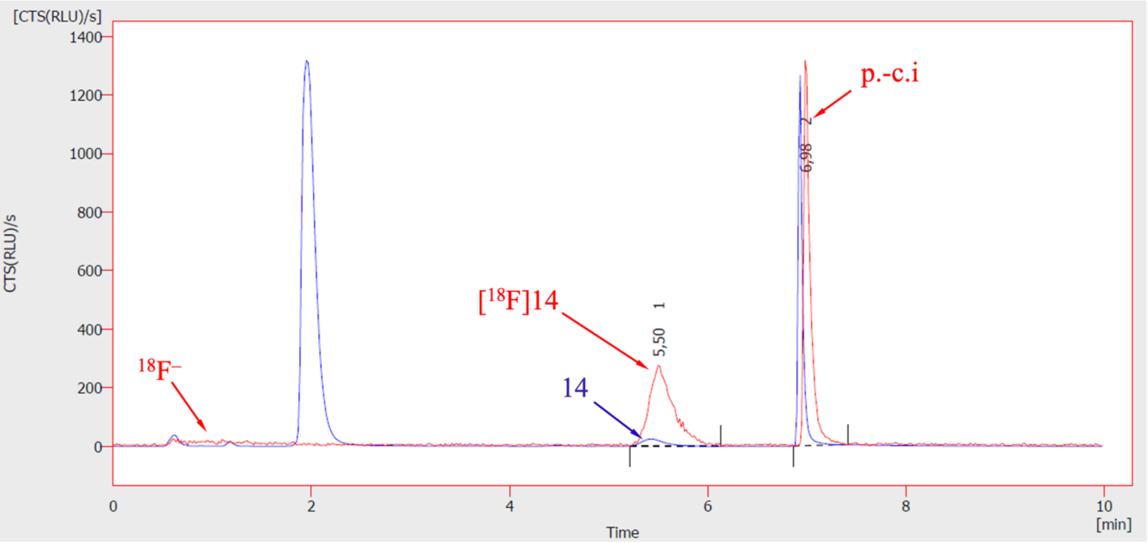

Result Table (Uncal - Data|alc-enhanced_820_27_06_2022_[4 DMI][18F]14_spike - HERM)

	Reten. Time [min]	Area [CTS(RLU)/s.s]	Height [CTS(RLU)/s]	Area [%]
1	5,500	4664,000	275,000	42,8
2	6,983	6230,500	1315,939	57,2
	Total	10894,500	1590,939	100,0

Figure S71: HPLC traces of crude 1-[^{18}F]fluoronaphthalene ([^{18}F]**14**) prepared from the corresponding boronic acid precursor (2.5 µmol) and spiked with 1-fluoronaphthalene (**14**). Blue trace: UV, λ = 254 nm; red trace: radioactivity. Abbreviation: p.-c.i – post-column injection.

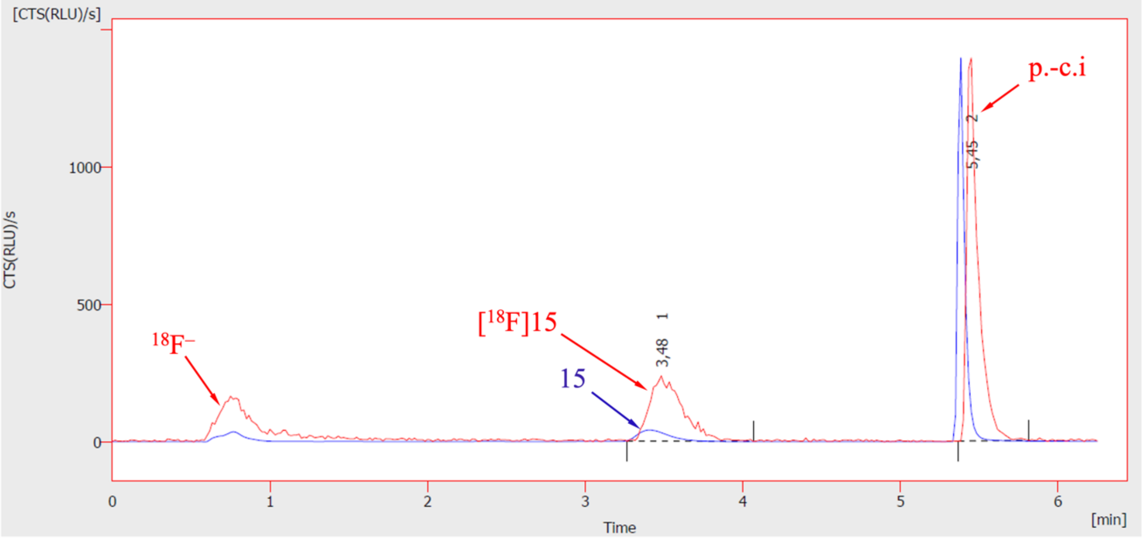

Result Table (Uncal - Data\alc-enhanced_810_27_06_2022_[4 DMI][18F]15_spike - HERM)

	Reten. Time [min]	Area [CTS(RLU)/s.s]	Height [CTS(RLU)/s]	Area [%]
1	3,483	3426,000	237,271	32,4
2	5,450	7132,000	1393,630	67,6
	Total	10558,000	1630,900	100,0

Figure S72: HPLC trace of crude 4-[^{18}F]fluorobenzyl alcohol ([^{18}F]**15**) prepared from the respective boronic acid precursor (2.5 µmol) and spiked with 4-fluorobenzyl alcohol (**15**). Blue trace: UV, λ = 254 nm; red trace: radioactivity. Abbreviation: p.-c.i – post-column injection.

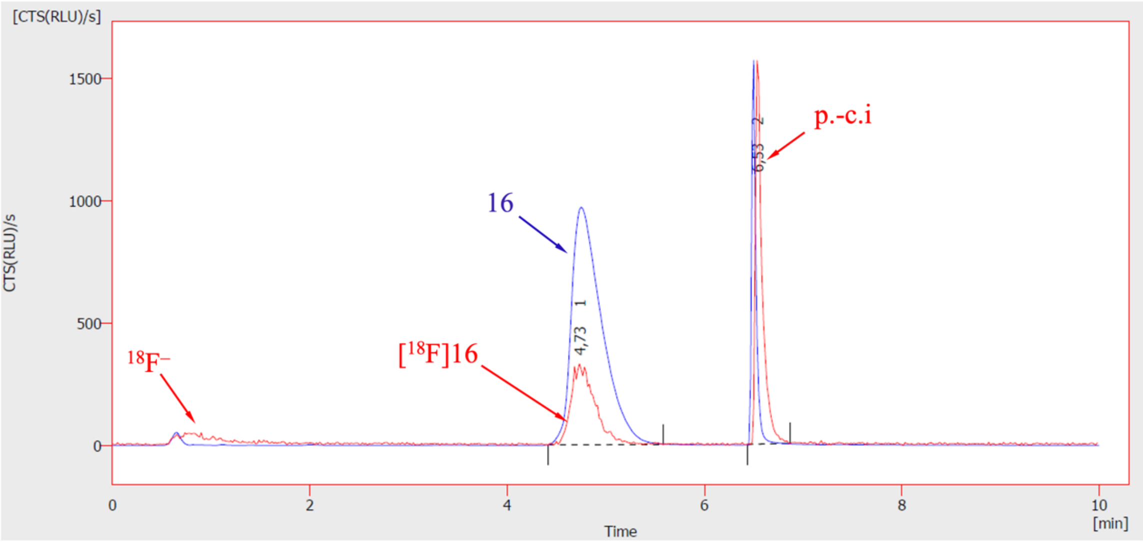

Result Table (Uncal - Data\alc-enhanced_816_27_06_2022_[4 DMI][18F]16_spike - HERM)

	Reten. Time [min]	Area [CTS(RLU)/s.s]	Height [CTS(RLU)/s]	Area [%]
1	4,733	5671,000	331,729	42,9
2	6,533	7543,000	1568,077	57,1
	Total	13214,000	1899,805	100,0

Figure S73: HPLC traces of crude 3-[^{18}F]fluoroanisole ([^{18}F]**16**) prepared from the corresponding boronic acid precursor (2.5 µmol) and spiked with 3-fluoroanisole (**16**). Blue trace: UV, λ = 254 nm; red trace: radioactivity. Abbreviation: p.-c.i – post-column injection.

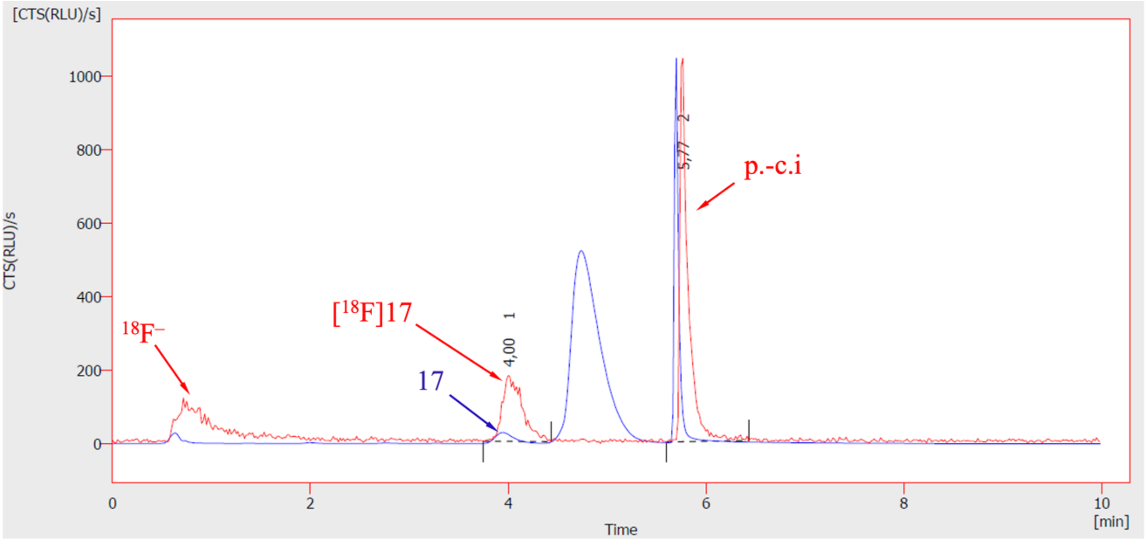

Result Table (Uncal - Data|alc-enhanced_817_27_06_2022_[4 DMI][18F]17_spike - HERM)

	Reten. Time [min]	Area [CTS(RLU)/s.s]	Height [CTS(RLU)/s]	Area [%]
1	4,000	2535,500	179,366	29,3
2	5,767	6110,000	1045,200	70,7
	Total	8645,500	1224,566	100,0

Figure S74: HPLC traces of crude 4-[^{18}F]fluoroanisole ([^{18}F]**17**) prepared from the corresponding boronic acid precursor (2.5 µmol) and spiked with 4-fluoroanisole (**17**). Blue: UV chromatogram, λ = 254 nm; red: radio chromatogram. Abbreviations: p.-c.i – post-column injection.

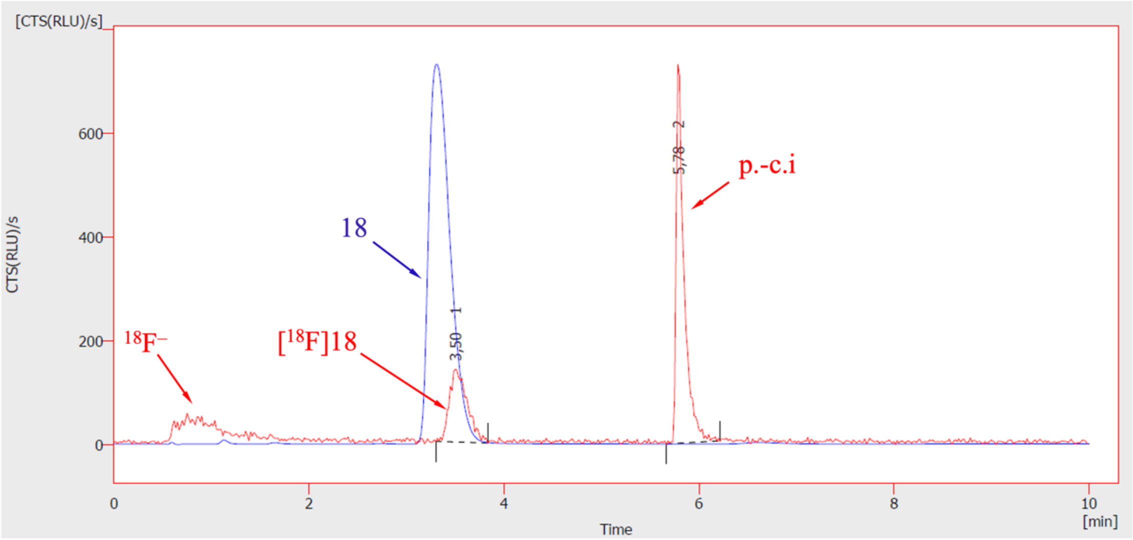

Result Table (Uncal - Data|alc-enhanced_818_27_06_2022_[4 DMI][18F]18_spike - HERM)

	Reten. Time [min]	Area [CTS(RLU)/s.s]	Height [CTS(RLU)/s]	Area [%]
1	3,500	1807,000	140,125	30,3
2	5,783	4162,000	729,727	69,7
	Total	5969,000	869,852	100,0

Figure S75: HPLC traces of crude methyl 4-[^{18}F]fluorobenzoate ([^{18}F]**18**) prepared from the corresponding boronic acid precursor (2.5 µmol) and spiked with methyl 4-flourobenzoate (**18**). Blue trace: UV, λ = 254 nm; red trace: radioactivity. Abbreviation: p.-c.i – post-column injection.

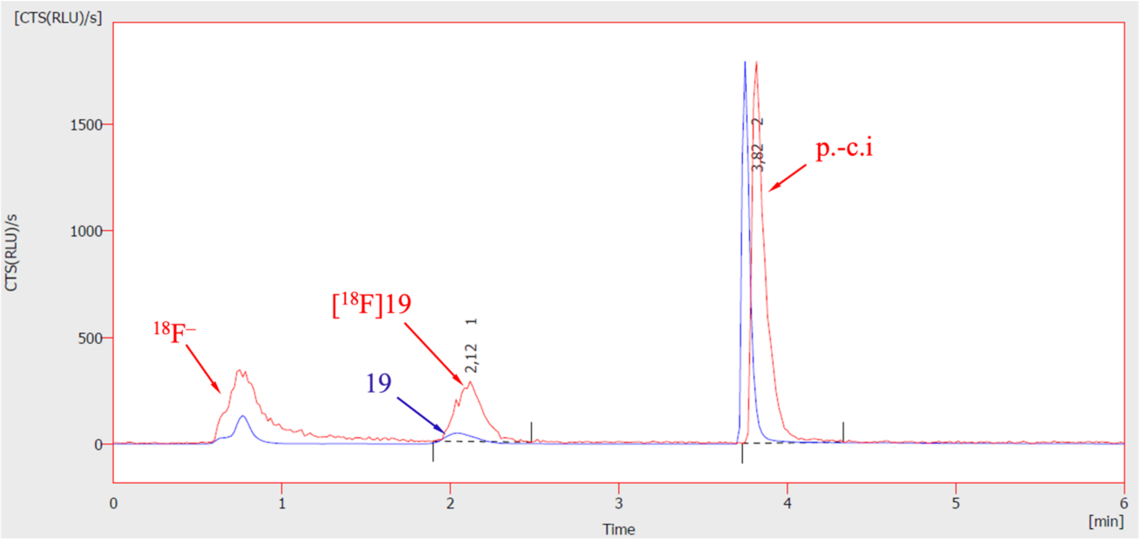

Result Table (Uncal - Data|alc-enhanced_811_27_06_2022_[4 DMI][18F]19_spike - HERM)

	Reten. Time [min]	Area [CTS(RLU)/s.s]	Height [CTS(RLU)/s]	Area [%]
1	2,117	3164,500	282,857	24,1
2	3,817	9988,000	1792,167	75,9
	Total	13152,500	2075,024	100,0

Figure S76: HPLC traces of crude 4-[^{18}F]fluorobenzamide ([^{18}F]**19**) prepared from the corresponding boronic acid precursor (2.5 µmol) and spiked with 4-fluorobenzamide (**19**). Blue trace: UV, λ = 254 nm; red trace: radioactivity. Abbreviation: p.-c.i – post-column injection.

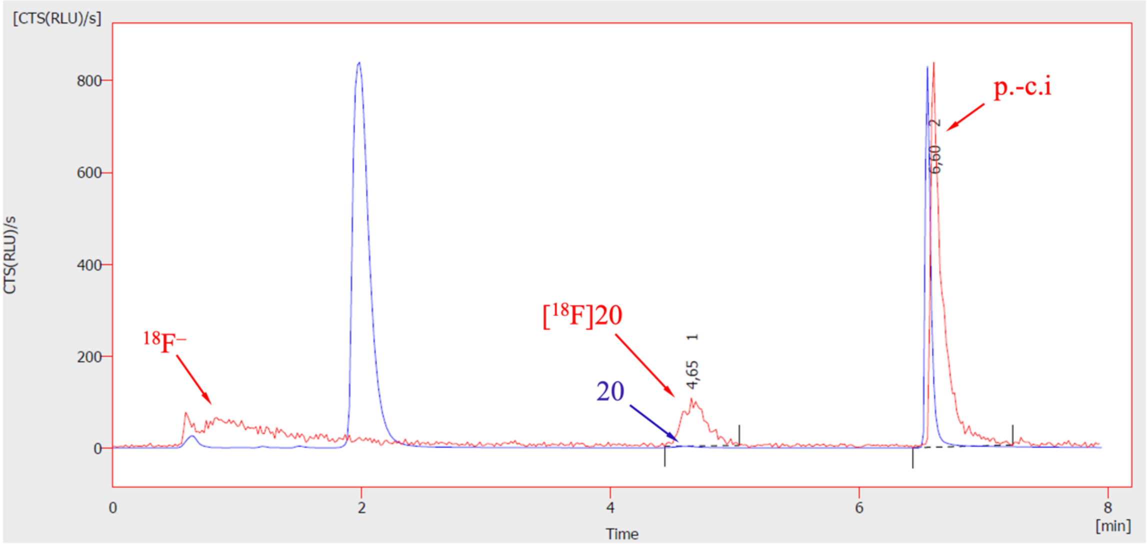

Result Table (Uncal - Data|alc-enhanced_821_27_06_2022_[4 DMI][18F]20_spike - HERM)

	Reten. Time [min]	Area [CTS(RLU)/s.s]	Height [CTS(RLU)/s]	Area [%]
1	4,650	1391,000	105,278	20,0
2	6,600	5570,000	837,750	80,0
	Total	6961,000	943,028	100,0

Figure S77: HPLC traces of crude 4-[^{18}F]fluoroiodobenzene ([^{18}F]**20**) prepared from the corresponding boronic acid precursor (2.5 µmol) and spiked with 4-fluoroiodobenzene (**20**). Blue trace: UV, λ = 254 nm; red trace: radioactivity. Abbreviation: p.-c.i – post-column injection.

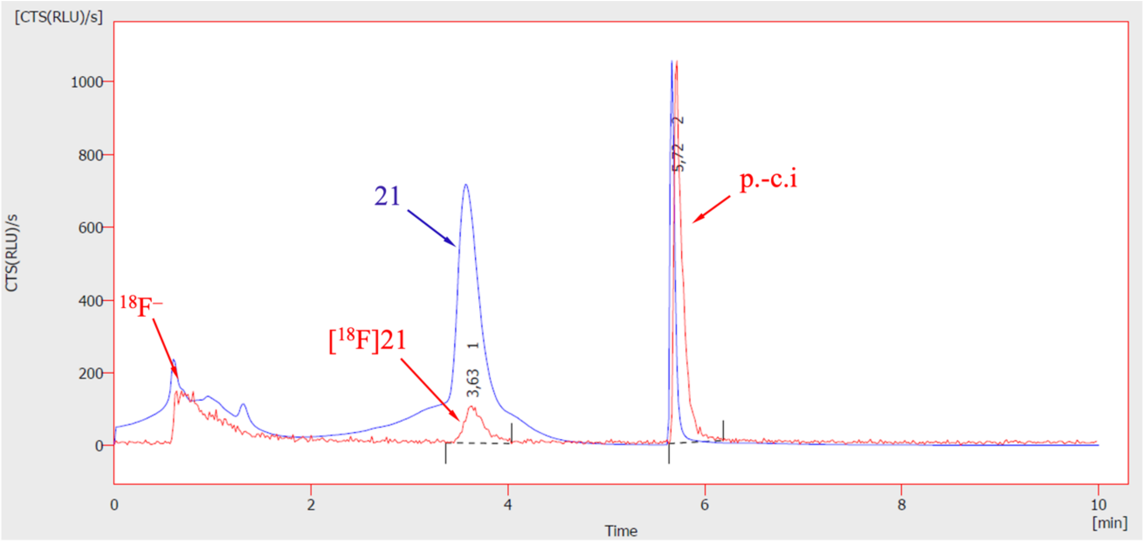

Result Table (Uncal - Data|alc-enhanced_814_27_06_2022_[4 DMI][18F]21_spike - HERM)

	Reten. Time [min]	Area [CTS(RLU)/s.s]	Height [CTS(RLU)/s]	Area [%]
1	3,633	1471,000	101,800	18,7
2	5,717	6395,000	1051,788	81,3
	Total	7866,000	1153,588	100,0

Figure S78: HPLC traces of crude 3-[^{18}F]fluorobenzaldehyde ([^{18}F]**21**) prepared from the corresponding boronic acid precursor (2.5 µmol) and spiked with 3-fluorobenzaldehyde (**21**). Blue trace: UV, λ = 254 nm; red trace: radioactivity. Abbreviation: p.-c.i – post-column injection.

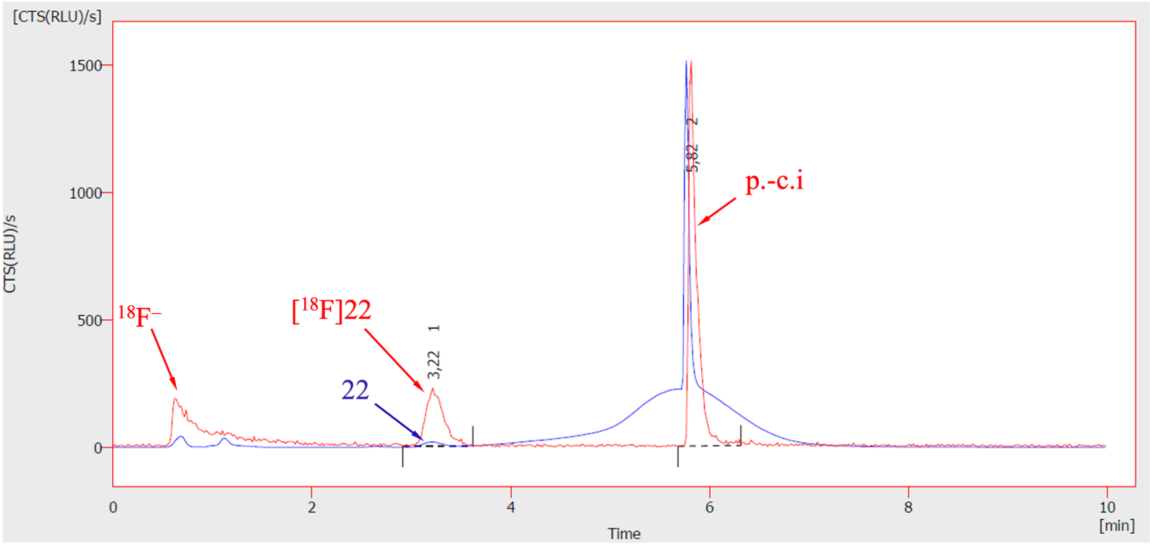

Result Table (Uncal - Data|alc-enhanced_815_27_06_2022_[4 DMI][18F]22_spike - HERM)

	Reten. Time [min]	Area [CTS(RLU)/s.s]	Height [CTS(RLU)/s]	Area [%]
1	3,217	2867,000	228,000	25,0
2	5,817	8623,000	1512,368	75,0
	Total	11490,000	1740,368	100,0

Figure S79: HPLC traces of crude 4-[^{18}F]fluorobenzaldehyde ([^{18}F]**22**) prepared from the corresponding boronic acid precursor (2.5 µmol) and spiked with 4-fluorobenzaldehyde (**22**). Blue trace: UV, λ = 254 nm; red trace: radioactivity. Abbreviation: p.-c.i – post-column injection.

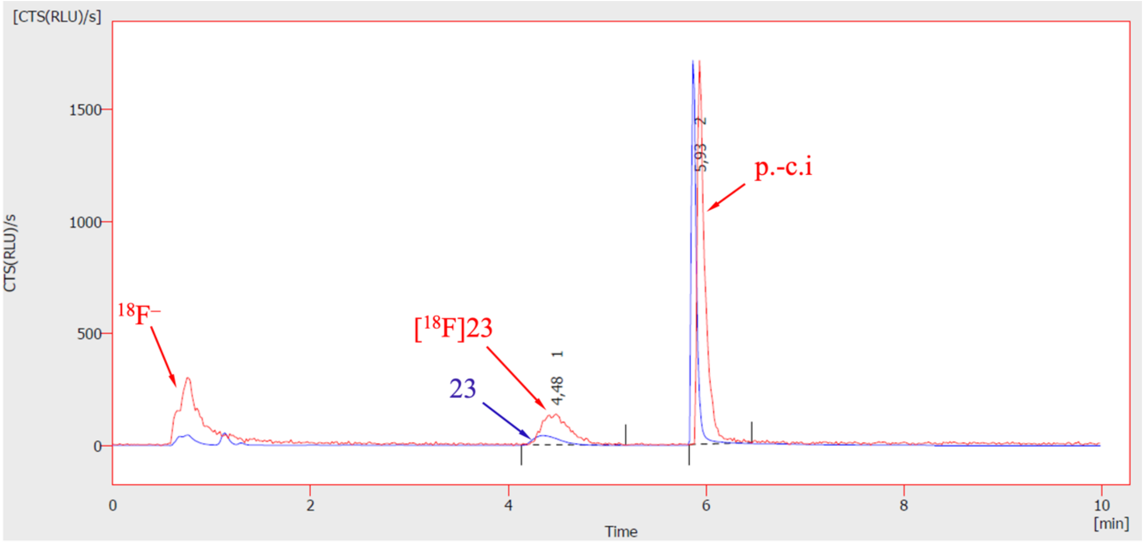

Result Table (Uncal - Data|alc-enhanced_812_27_06_2022_[4 DMI][18F]23_spike - HERM)

	Reten. Time [min]	Area [CTS(RLU)/s.s]	Height [CTS(RLU)/s]	Area [%]
1	4,483	2820,500	138,333	23,3
2	5,933	9288,000	1712,579	76,7
	Total	12108,500	1850,912	100,0

Figure S80: HPLC traces of crude 4-[^{18}F]fluorophenol ([^{18}F]**23**) prepared from the corresponding boronic acid precursor (2.5 µmol) and spiked with 4-fluorophenol (**23**). Blue trace: UV, λ = 254 nm; red trace: radioactivity. Abbreviation: p.-c.i – post-column injection.

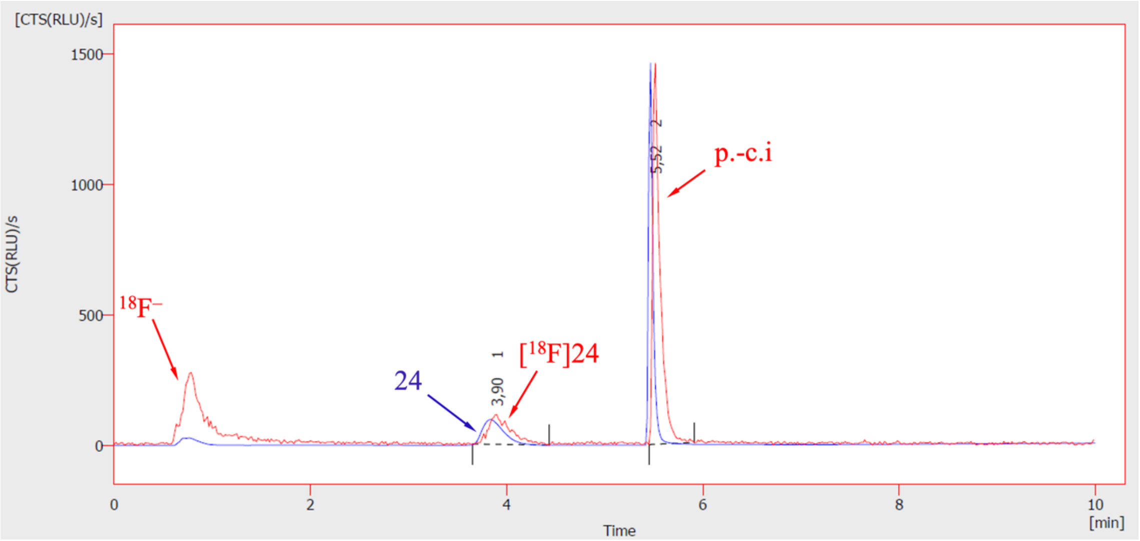

Result Table (Uncal - Data|alc-enhanced_813_27_06_2022_[4 DMI][18F]24_spike - HERM)

	Reten. Time [min]	Area [CTS(RLU)/s.s]	Height [CTS(RLU)/s]	Area [%]
1	3,900	1801,000	114,277	18,9
2	5,517	7704,000	1461,000	81,1
	Total	9505,000	1575,277	100,0

Figure S81: HPLC traces of crude 3-[^{18}F]fluoroaniline ([^{18}F]**24**) prepared from the corresponding boronic acid precursor (2.5 µmol) and spiked with 3-fluoroaniline (**24**). Blue trace: UV, λ = 254 nm; red trace: radioactivity. Abbreviation: p.-c.i – post-column injection.

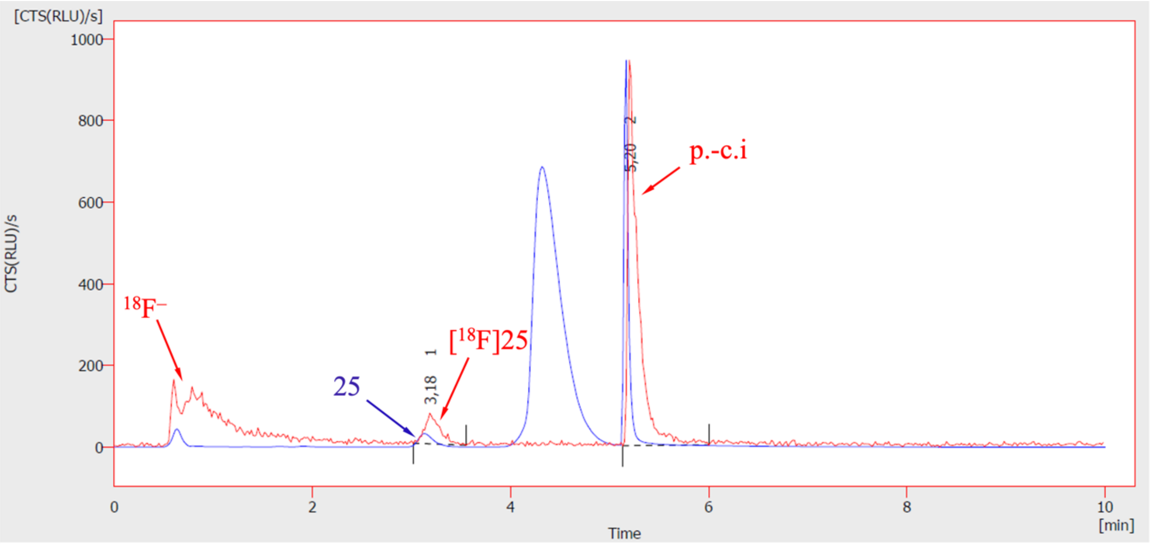

Result Table (Uncal - Data\alc-enhanced_819_27_06_2022_[4 DMI][18F]25_spike - HERM)

	Reten. Time [min]	Area [CTS(RLU)/s.s]	Height [CTS(RLU)/s]	Area [%]
1	3,183	807,000	75,563	10,5
2	5,200	6865,000	943,846	89,5
	Total	7672,000	1019,409	100,0

Figure S82: HPLC traces of crude 2-[^{18}F]fluoroanisole ([^{18}F]**25**) prepared from the corresponding boronic acid precursor (2.5 µmol) and spiked with 2-[^{18}F]fluoroanisole (**25**). Blue trace: UV, λ = 254 nm; red trace: radioactivity. Abbreviation: p.-c.i – post-column injection.

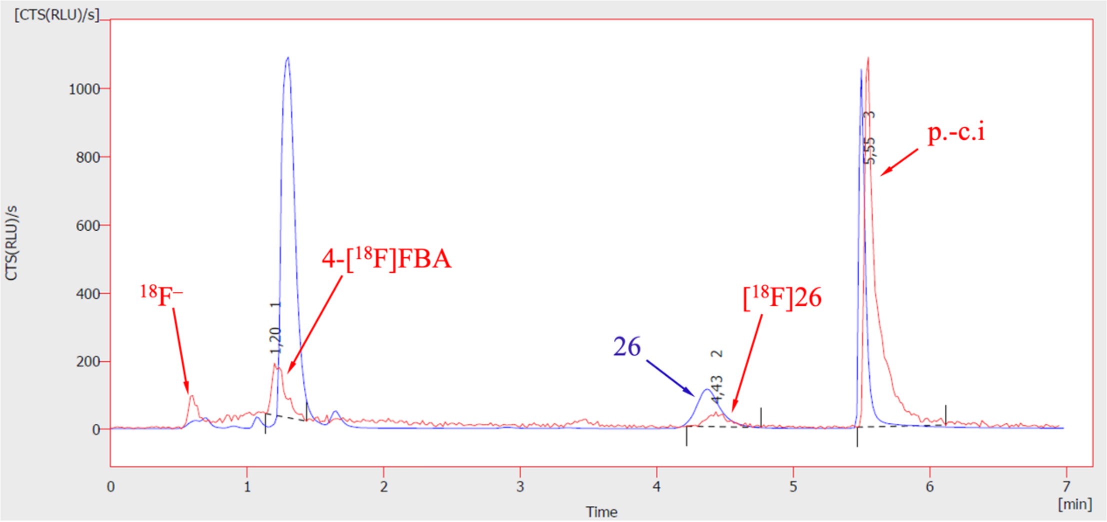

Result Table (Uncal - Data\alc-enhanced_822_27_06_2022_[4 DMI][18F]26_spike - HERM)

	Reten. Time [min]	Area [CTS(RLU)/s.s]	Height [CTS(RLU)/s]	Area [%]	RCC [%]
1	1,200	1085,000	152,889	13,0	100,000
2	4,433	478,000	43,576	5,7	44,055
3	5,550	6804,000	1086,231	81,3	627,097
	Total	8367,000	1282,695	100,0	771,152

Figure S83: HPLC traces of crude 2,3,5,6-tetrafluorophenyl 4-[^{18}F]fluorobenzoate ([^{18}F]**26**) prepared from the corresponding boronic acid precursor (2.5 µmol) in *n*BuOH/DMI and spiked with 2,3,5,6-tretrafluorophenyl 4-fluorobenzoate (**26**). Blue trace: UV, λ = 254 nm; red trace: radioactivity. Abbreviations: 4-[^{18}F]FBA – 4-[^{18}F]fluorobenzoic acid; p.-c.i – post-column injection.

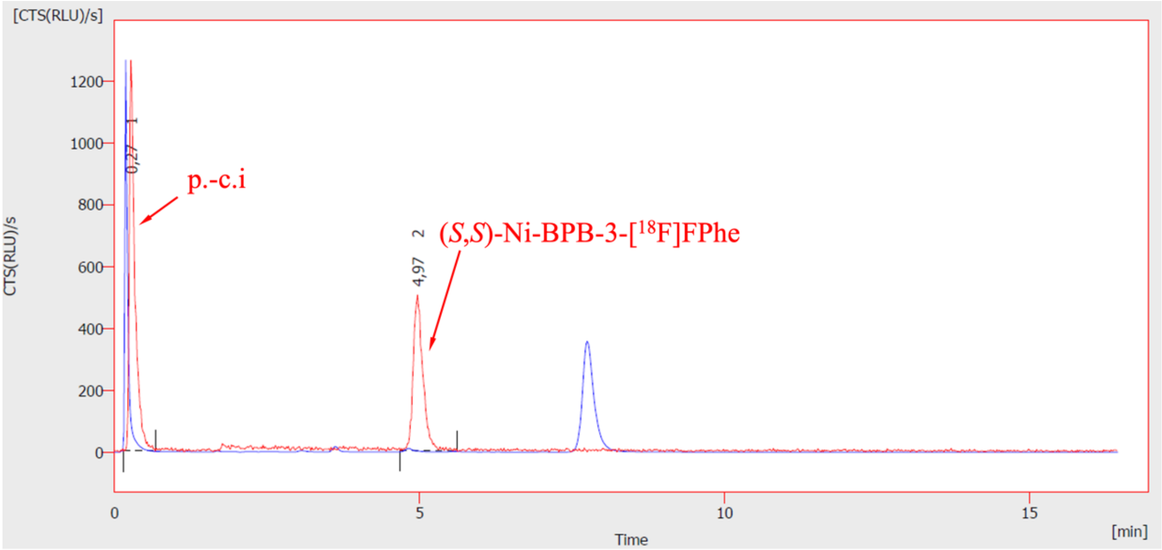

Result Table (Uncal - Data|alc-enhanced_718_20_04_2022_[4 DMI]L-[18F]FPhe - HERM)

	Reten. Time [min]	Area [CTS(RLU)/s.s]	Height [CTS(RLU)/s]	Area [%]
1	0,267	8140,000	1263,000	59,3
2	4,967	5583,000	502,911	40,7
	Total	13723,000	1765,911	100,0

Figure S84: HPLC traces of crude (*S*,*S*)-Ni-BPB-3-[^{18}F]FPhe prepared from (*S*,*S*)-**10** (2.5 µmol). Blue trace: UV, λ = 254 nm; red trace: radioactivity. Abbreviation: p.-c.i – post-column injection.

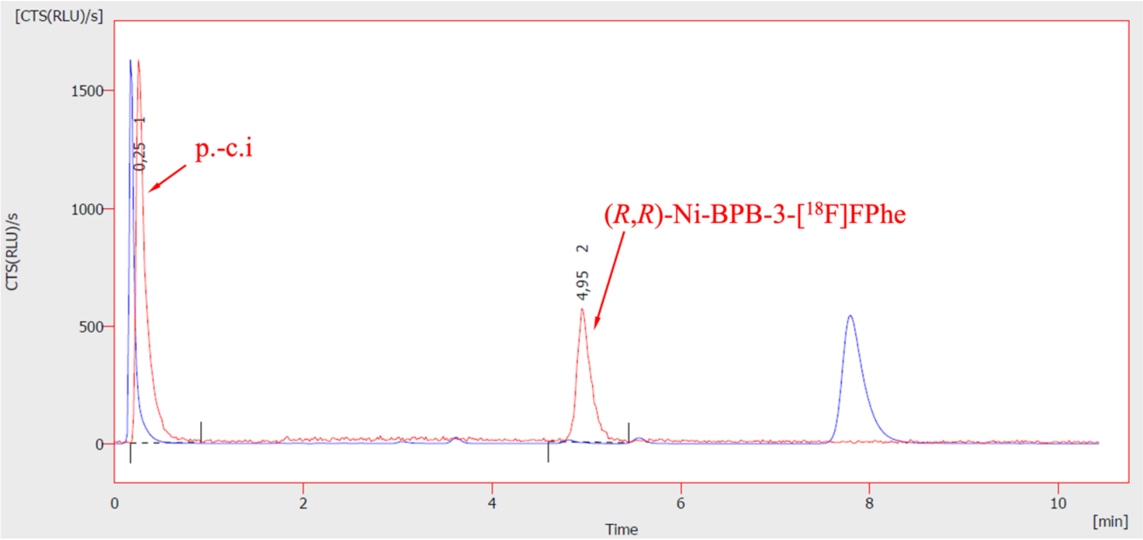

Result Table (Uncal - Data|alc-enhanced_721_20_04_2022_[4 DMI]D-[18F]FPhe - HERM)

	Reten. Time [min]	Area [CTS(RLU)/s.s]	Height [CTS(RLU)/s]	Area [%]
1	0,250	11845,500	1626,667	66,3
2	4,950	6025,000	566,471	33,7
	Total	17870,500	2193,137	100,0

Figure S85: HPLC traces of crude (*R*,*R*)-Ni-BPB-3-[^{18}F]FPhe prepared from (*R*,*R*)-**10** (2.5 µmol). Blue trace: UV, λ = 254 nm; red trace: radioactivity. Abbreviation: p.-c.i – post-column injection.

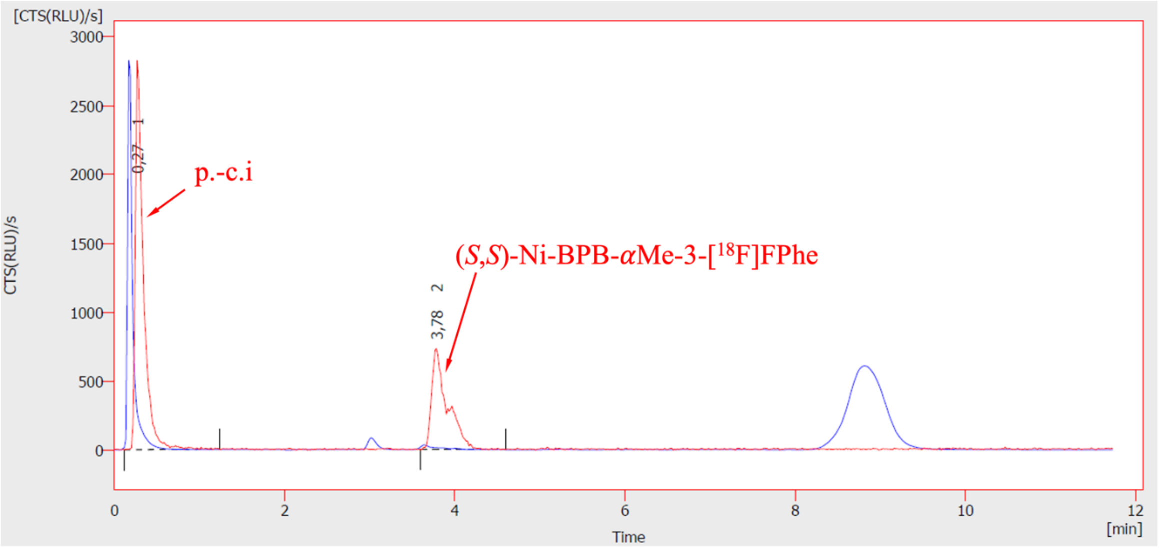

Result Table (Uncal - Data\alc-enhanced_731_21_04_2022_[4 DMI]L-alpha_Me_[18F]FPhe - HERM)

	Reten. Time [min]	Area [CTS(RLU)/s.s]	Height [CTS(RLU)/s]	Area [%]
1	0,267	18735,500	2825,597	66,3
2	3,783	9534,000	733,817	33,7
	Total	28269,500	3559,414	100,0

Figure S86: HPLC traces of crude (*S*,*S*)-Ni-BPB-αMe-3-[^{18}F]FPhe prepared from (*S*,*S*)-**12** (2.5 µmol). Blue trace: UV, λ = 254 nm; red trace: radioactivity. Abbreviation: p.-c.i – post-column injection.

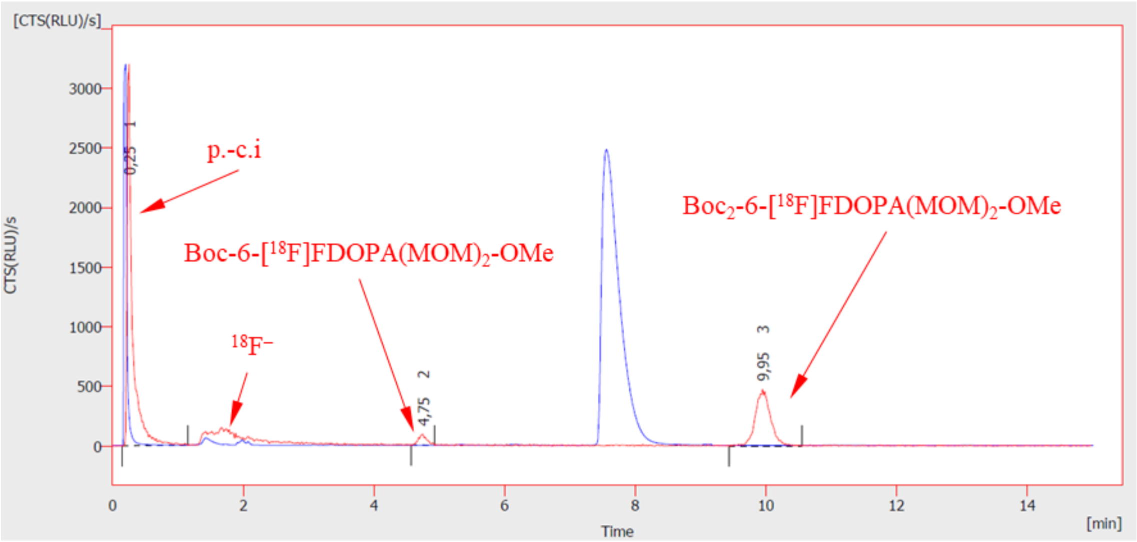

Result Table (Uncal - Data\alc-enhanced_825_28_06_2022_[4 DMI]FDOPA-SnMe3_90°C_MeOH - HERM)

	Reten. Time [min]	Area [CTS(RLU)/s.s]	Height [CTS(RLU)/s]	Area [%]
1	0,250	17861,000	3199,400	68,1
2	4,750	829,000	94,500	3,2
3	9,950	7530,500	476,537	28,7
	Total	26220,500	3770,437	100,0

Figure S87: HPLC traces of crude Boc$_2$-6-[^{18}F]FDOPA(MOM)$_2$-OMe prepared from **S6** (2.5 µmol). Partial deprotection of the radiolabeled product to Boc-6-[^{18}F]FDOPA(MOM)$_2$-OMe was observed.[25] Blue trace: UV, λ = 254 nm; red trace: radioactivity. Abbreviation: p.-c.i – post-column injection.

3.15 Preparation of 6-$[^{18}F]$FDOPA

$[^{18}F]F^-$ (0.2–3 GBq) was eluted from the QMA cartridge with a solution of Et_4NOTf (1 mg, 3.6 µmol) in anhydrous MeOH (500 µL). MeOH was evaporated at 60 °C under reduced pressure in a stream of argon. The reactor was filled with argon and sealed with a silicone septum. Thereafter, a solution of **S6** (1.7 mg, 2.5 µmol) and $Cu(4\text{-}PhPy)_4(ClO_4)_2$ (8.8 mg, 10 µmol) in anhydrous DMI (800 µL) was added via a cannula through the septum and the reaction mixture was heated at 90 °C for 10 min. That followed, the reaction mixture was diluted with H_2O (15 mL) and loaded onto a C18 cartridge. The cartridge was washed with H_2O (5 mL) and the radiolabeled intermediate was eluted with MeOH (500 µL). The MeOH was evaporated at 60 °C under reduced pressure in a stream of argon, 6 M HCl (500 µL) was added to the residue and the mixture was heated at 110 °C for 10 min. Thereafter, 6 M NaOH (400 µL) followed by H_2O (600 µL) were added to the mixture, and the resulting solution was loaded onto a preparative HPLC column. The product fraction eluting at 9–11 min was collected.

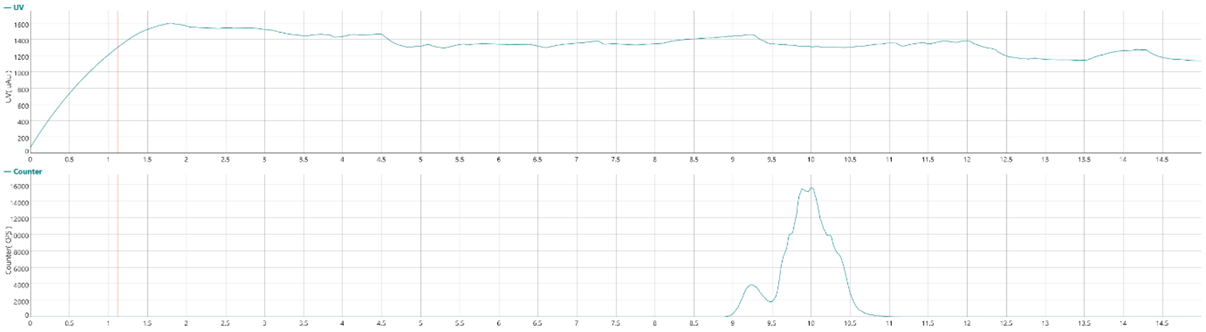

Figure S88: Purification of 6-$[^{18}F]$FDOPA by preparative HPLC (top: UV trace, λ=254 nm; bottom: radioactivity trace).

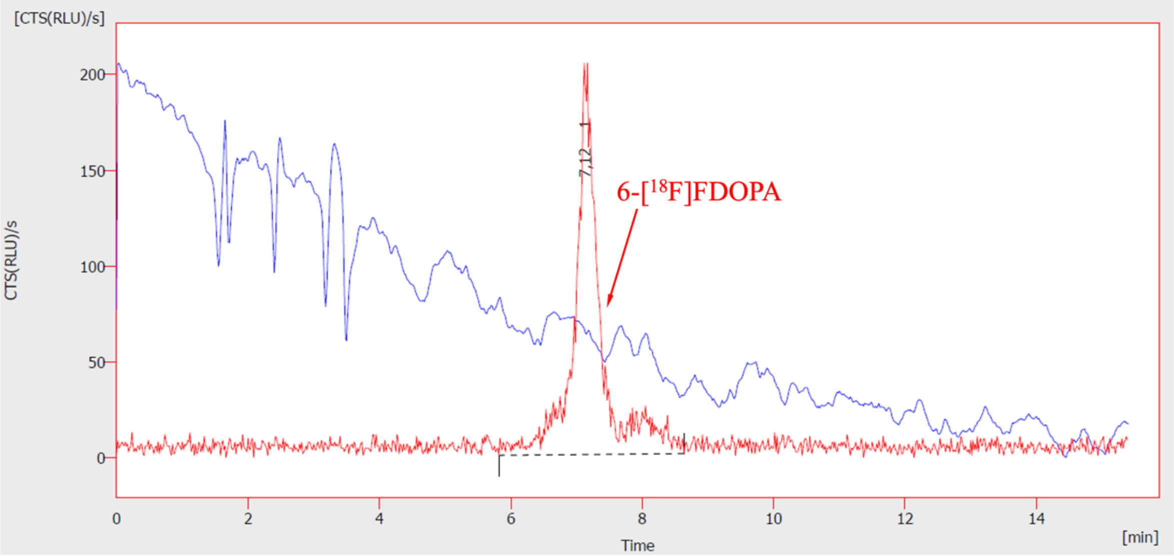

Result Table (Uncal - Data|alc-enhanced_600_09_03_2022_[4 DMI][18F]FDOPA_hplc - HERM)

	Reten. Time [min]	Area [CTS(RLU)/s.s]	Height [CTS(RLU)/s]	Area [%]
1	7,117	5387,500	204,538	100,0
	Total	5387,500	204,538	100,0

Figure S89: HPLC traces of purified 6-[^{18}F]FDOPA for quality control. Blue trace: UV channel, λ=254 nm; red trace: radioactivity channel

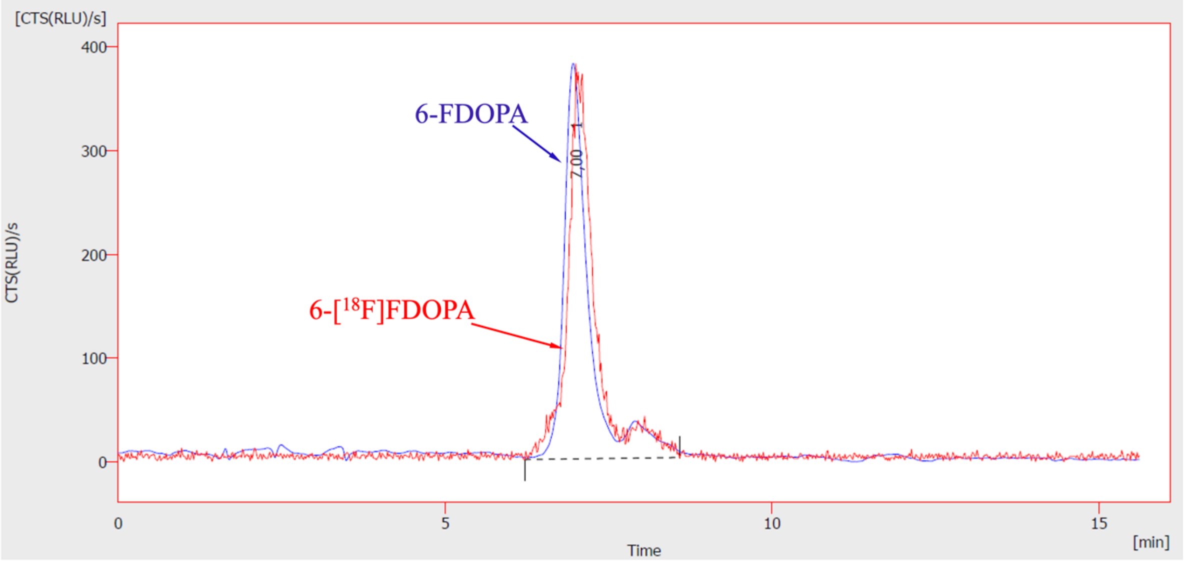

Result Table (Uncal - Data|alc-enhanced_601_09_03_2022_[4 DMI][18F]FDOPA_hplc_spike - HERM)

	Reten. Time [min]	Area [CTS(RLU)/s.s]	Height [CTS(RLU)/s]	Area [%]
1	7,000	10902,000	381,338	100,0
	Total	10902,000	381,338	100,0

Figure S90: HPLC traces of purified 6-[^{18}F]FDOPA spiked with an authentic sample of 6-FDOPA. Blue trace: UV channel, λ=254 nm; red trace: radioactivity channel.

4 References

[1] M. Willmann, J. Hegger, B. Neumaier, J. Ermert, *ACS Med. Chem. Lett.* **2021**, *12*, 738–744.

[2] C. Hoffmann, S. Evcüman, F. Neumaier, B. D. Zlatopolskiy, S. Humpert, D. Bier, M. Holschbach, A. Schulze, H. Endepols, B. Neumaier, *ACS Chem. Neurosci.* **2021**, *12*, 3335–3346.

[3] S. Li, Z. Cai, X. Wu, D. Holden, R. Pracitto, M. Kapinos, H. Gao, D. Labaree, N. Nabulsi, R. E. Carson, Y. Huang, *ACS Chem. Neurosci.* **2019**, *10*, 1544–1554.

[4] A. Craig, N. Kolks, E. A. Urusova, J. Zischler, M. Brugger, H. Endepols, B. Neumaier, B. D. Zlatopolskiy, *Chem. Commun.* **2020**, *56*, 9505–9508.

[5] A. Gaucher, L. Dutot, O. Barbeau, W. Hamchaoui, M. Wakselman, J.-P. Mazaleyrat, *Tetrahedron: Asymmetry* **2005**, *16*, 857–864.

[6] A. R. Byington, W. E. Bull, *Inorganica Chim. Acta* **1977**, *21*, 239–244.

[7] J. S. Haynes, S. J. Rettig, J. R. Sams, J. Trotter, R. C. Thompson, *Inorg. Chem.* **1988**, *27*, 1237–1241.

[8] Y. Agnus, M. Labarelle, R. Louis, B. Metz, *Acta Crystallogr. Sect. C Cryst. Struct. Commun.* **1994**, *50*, 536–538.

[9] W. Libug, I. Uruska, *Inorg. Chem.* **1966**, *5*, 256–264.

[10] G. A. Bowmaker, C. Di Nicola, C. Pettinari, B. W. Skelton, N. Somers, A. H. White, *Dalt. Trans.* **2011**, *40*, 5102.

[11] M. H. H. Wurzenberger, N. Szimhardt, J. Stierstorfer, *J. Am. Chem. Soc.* **2018**, *140*, 3206–3209.

[12] M. Roy, D. N. K. Pham, A. Kreider-Mueller, J. A. Golen, D. R. Manke, *Acta Crystallogr. Sect. C Struct. Chem.* **2018**, *74*, 263–268.

[13] G. A. Barclay, C. H. L. Kennard, *J. Chem. Soc.* **1961**, 5244.

[14] F. Hanic, D. Štempelová, K. Hanicová, *Acta Crystallogr.* **1964**, *17*, 633–639.

[15] A. M. Kałuża, S. Mukherjee, S.-Q. Wang, D. J. O'Hearn, M. J. Zaworotko, *Chem. Commun.* **2020**, *56*, 1940–1943.

[16] K. Al Sarraj, J. Gouteron, S. Jeannin, Y. Jeannin, *Acta Crystallogr. Sect. C Cryst. Struct. Commun.* **1987**, *43*, 1261–1264.

[17] V. Raab, M. Merz, J. Sundermeyer, *J. Mol. Catal. A Chem.* **2001**, *175*, 51–63.

[18] N. J. Taylor, E. Emer, S. Preshlock, M. Schedler, M. Tredwell, S. Verhoog, J. Mercier, C. Genicot, V. Gouverneur, *J. Am. Chem. Soc.* **2017**, *139*, 8267–8276.

[19] P. Luo, J. P. Dinnocenzo, *J. Org. Chem.* **2015**, *80*, 9240–9246.

[20] K. Komeyama, R. Asakura, K. Takaki, *Org. Biomol. Chem.* **2015**, *13*, 8713–8716.

[21] A. Hofer, G. Kovacs, A. Zappatini, M. Leuenberger, M. A. Hediger, M. Lochner, *Bioorg. Med. Chem.* **2013**, *21*, 3202–3213.

[22] B. Shi, R. W. Boyle, *J. Chem. Soc. Perkin Trans. 1* **2002**, 1397–1400.

[23] S. P. A. Hinkes, C. D. P. Klein, *Org. Lett.* **2019**, *21*, 3048–3052.

[24] N. Walter, J. Bertram, B. Drewes, V. Bahutski, M. Timmer, M. B. Schütz, F. Krämer, F. Neumaier, H. Endepols, B. Neumaier, B. D. Zlatopolskiy, *Eur. J. Med. Chem.* **2022**, *237*, 114383.

[25] J. Zischler, N. Kolks, D. Modemann, B. Neumaier, B. D. Zlatopolskiy, *Chem. - Eur. J.* **2017**, *23*, 3251–3256.
